# Control of stereogenic oxygen in a helically chiral oxonium ion

**DOI:** 10.1038/s41586-023-05719-z

**Published:** 2023-03-15

**Authors:** Owen Smith, Mihai V. Popescu, Madeleine J. Hindson, Robert S. Paton, Jonathan W. Burton, Martin D. Smith

**Affiliations:** 1 Chemistry Research Laboratory, University of Oxford, Oxford, UK; 2 Department of Chemistry, Colorado State University, Ft. Collins, CO USA

**Keywords:** Stereochemistry, Reaction mechanisms, Synthetic chemistry methodology

## Abstract

The control of tetrahedral carbon stereocentres remains a focus of modern synthetic chemistry and is enabled by their configurational stability. By contrast, trisubstituted nitrogen^1^, phosphorus^2^ and sulfur compounds^3^ undergo pyramidal inversion, a fundamental and well-recognized stereochemical phenomenon that is widely exploited^4^. However, the stereochemistry of oxonium ions—compounds bearing three substituents on a positively charged oxygen atom—is poorly developed and there are few applications of oxonium ions in synthesis beyond their existence as reactive intermediates^5,6^. There are no examples of configurationally stable oxonium ions in which the oxygen atom is the sole stereogenic centre, probably owing to the low barrier to oxygen pyramidal inversion^7^ and the perception that all oxonium ions are highly reactive. Here we describe the design, synthesis and characterization of a helically chiral triaryloxonium ion in which inversion of the oxygen lone pair is prevented through geometric restriction to enable it to function as a determinant of configuration. A combined synthesis and quantum calculation approach delineates design principles that enable configurationally stable and room-temperature isolable salts to be generated. We show that the barrier to inversion is greater than 110 kJ mol^−1^ and outline processes for resolution. This constitutes, to our knowledge, the only example of a chiral non-racemic and configurationally stable molecule in which the oxygen atom is the sole stereogenic centre.

## Abstract (web summary)

The design, synthesis and characterization of a helically chiral triaryloxonium ion is reported, which is an example of a chiral non-racemic and configurationally stable molecule in which the oxygen atom is the sole stereogenic centre.

## Main

**Fig. 1:**
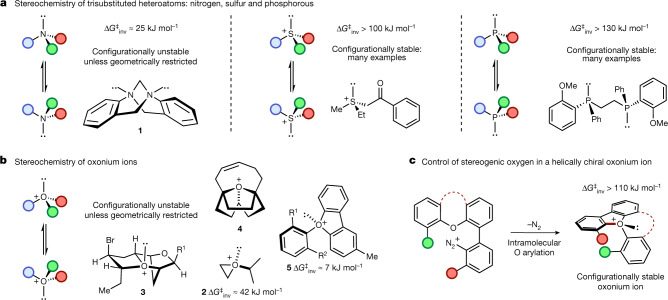
Pyramidal inversion of trisubstituted heteroatoms. **a**, Pyramidal inversion of common trisubstituted heteroatoms occurs at different rates, leading to different configurational stabilities for tertiary amines, sulfonium salts and phosphines. **b**, Inversion of oxonium ions is rapid unless prevented by geometrical restriction: there are no examples of room-temperature configurationally stable oxonium ions bearing oxygen as the sole stereogenic centre. **c**, Synthesis and characterization of configurationally stable oxonium ions bearing a stereogenic oxygen. Counterions are not shown for clarity.Δ*G*^‡^_inv_ = Gibbs free energy of activation for the inversion process.

The pyramidal inversion of trisubstituted amines, phosphines and sulfonium salts is a fundamental stereochemical process that has been intensively studied, leading to an understanding of the factors that affect the magnitude of their inversion barriers^1^. This has enabled the generation of a myriad of configurationally stable chiral phosphines (which possess barriers to inversion of 120–185 kJ mol^−1^)^3^ and sulfonium salts (with barriers of 100–130 kJ mol^−1^)^4^. The inversion of the nitrogen atom in a tertiary amine has a relatively low barrier (20–25 kJ mol^−1^) and there is a substantial contribution from quantum tunnelling to the observed rate^2^; this means that the preparation of configurationally stable tertiary amines bearing a stereogenic nitrogen atom is particularly challenging. However, there are numerous examples of bicyclic amines such as Tröger’s base **1**^8–10^, cinchona alkaloids^11^ and others^12,13^ in which nitrogen inversion is restricted by virtue of conformational rigidity, enabling access to compounds bearing a stereogenic nitrogen (Fig. 1a)^14^. By contrast, the study of oxonium ions is much less developed^15,16^. The only empirical measurement of the inversion barrier for a trivalent oxygen compound is for the epoxide-derived oxonium salt **2** that has a barrier to inversion of 42 kJ mol^−1^, which is substantially below that required to maintain configurational stability at ambient temperature^7^. Consequently, there are no examples of the isolation of enantioenriched oxonium ions in which the oxygen atom^17,18^ is the sole stereogenic centre (Fig. 1b).

Oxonium ions are considered to be highly reactive and are invoked as transient intermediates in a range of synthetic transformations including natural product biosynthesis and total synthesis^19–24^. This has led to the characterization of complex trialkyloxonium ions such as **3**, a proposed intermediate in the synthesis of the Laurencia family of natural products. The fused ring system in **3** prevents inversion, and hence the oxygen is stereogenic; in this case the oxonium ion is formed in a diastereoselective manner from a precursor bearing several carbon stereocenters^5^. Tetracyclic oxonium ion **4** possesses a stereogenic oxygen due to its non-symmetrical bridge^25^. The best-known oxonium ions are arguably the trialkyl tetrafluoroborates, first synthesized by Meerwein more than 80 years ago^6,26^. Although these are stable and isolable, they are also some of the most reactive alkylating agents used in a synthetic chemistry laboratory and react readily with weak nucleophiles including water. Other alkyl oxonium ions have been prepared and studied^27–30^, but species stable to nucleophiles were elusive until Mascal reported oxatriquinane oxonium ions^31–34^; this indicates the potential impact upon stability of embedding oxonium ions in ring structures. In contrast to most alkyl-substituted oxonium ions, simple triaryloxonium ions are almost trigonal planar about oxygen and are stable isolable solids resistant to the action of many nucleophiles^35–37^. Dibenzofuran-derived triaryloxonium ions are pyramidal about the oxygen atom^38^, which led to Baldridge and Siegel investigating rotation about the exocyclic C–O bond in oxonium salts such as **5** (R^1 ^= Et, R^2 ^= H); this showed that the barrier (estimated at more than 84 kJ mol^−1^) was probably relatively close to Oki’s definition of atropisomerism^39^. However, oxygen inversion was calculated to require substantially less energy (R^1 ^= R^2 ^= Me ≈ 7 kJ mol^−1^)^40^. We reasoned that joining of the aromatic rings in the triaryloxonium scaffold with linkers of various lengths and flexibilities should increase the barrier to oxygen inversion while also maintaining the stability exhibited by other triaryloxonium ions (Fig. 1c). A sufficiently high enantiomerization barrier (which we have defined as greater than 107 kJ mol^−1^, *t*_1/2 _rac = 7 days at 25 °C; *t*_1/2_ rac = the time during which the enantiomeric purity of a chiral sample has reduced from 100 to 50% enantiomeric excess) would effectively lock the configuration of the stereogenic oxygen lone pair on a laboratory timescale and generate helically chiral molecules^41,42^. We reasoned that an intramolecular O-arylation of a dibenzofuran scaffold with a diazonium salt could offer a viable route to such molecules.

**Fig. 2:**
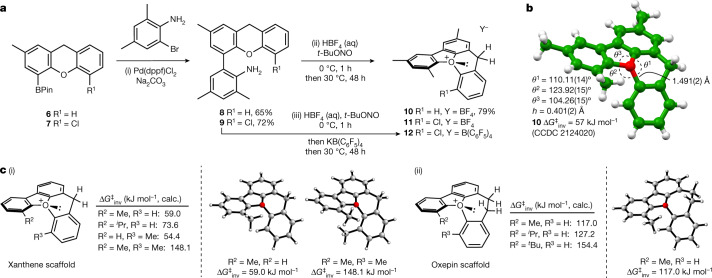
Synthetic route to and quantum chemical calculations of first-generation stereogenic-at-oxygen compounds. **a**, Reagents and conditions: (i) Pd(dppf)Cl_2_·CH_2_Cl_2_ (5 mol%), Na_2_CO_3_ (4 equivalents), PhMe/H_2_O (1:1, v/v), 95 °C, 24 h; (ii) HBF_4_ (48% aq, 5 equivalents), *t*-BuONO (5 equivalents), CH_2_Cl_2_/IPA (1:1), 0 °C, 1 h then 30 °C, 48 h; (iii) HBF_4_ (48% aq, 5 equivalents), *t*-BuONO (5 equivalents), CH_2_Cl_2_/IPA (1:1), 0 °C, 1 h, then KB(C_6_F_5_)_4_, then 30 °C, 48 h. **b**, Crystal and molecular structure of fused pentacyclic oxonium salt **10** from single-crystal X-ray diffraction studies. The tetrafluoroborate counterion is omitted for clarity. Numbers in parentheses indicate standard uncertainties. **c**, B3LYP-D3(BJ)/6.31+G(d,p) computed inversion barriers for a series of oxonium ions based upon a dibenzofuran-xanthene core (i) and a dibenzofuran-benzooxepine core (ii). *h*, Height of conceptualized triangular oblique pyramid with oxygen at its apex and the adjacent carbons of the attached O-aryl rings forming the base; Calc., calculated.

We initially selected a dibenzofuran−xanthene scaffold for our investigations as the CH_2_ protons in the xanthene backbone could be used as a reporter group to measure inversion barriers by variable temperature ^1^H NMR spectroscopy. We prepared the functionalized xanthenes **6** and **7** from commercial materials (see page 3 of the Supplementary Information for details); these were cross-coupled with 4,6-dimethyl-2-bromoaniline to afford biaryls **8** and **9**. Diazotization and intramolecular O-arylation with a loss of nitrogen led to the formation of pentacyclic oxonium tetrafluoroborates **10** and **11** (Fig. 2a). The structure of compound **10** was confirmed by single-crystal X-ray diffraction, which shows that the compound is pyramidal at the oxygen (sum of the angles around the oxygen *θ*^1 ^+ *θ*^2 ^+ *θ*^3 ^= 338.3(3)° versus 345(1)° for the dibenzofuran parent oxonium^43^, and the apex height *h* = 0.401(2) Å, Fig. 2) with the longest C–O bond length of 1.491(2) Å. The dibenzofuran ring is planar (internal C–O–C angle 104.26 (15)°), with the central ring of the xanthene adopting a boat-like conformation that places its two adjoining aromatic rings in different planes (Fig. 2b). The xanthene C–O–C internal angle is 110.11(14)° (versus 119.3° in the parent heterocycle xanthene itself), indicative of some angle strain at the fusion with the benzofuran ring, although that does not seem to compromise the stability of the compound, which can be stored indefinitely at room temperature without special precautions. Low-temperature ^1^H NMR spectroscopy was used to determine the enantiomerization barrier as 58.3 kJ mol^−1^ (see page 23 of the Supplementary Information for further details), which is substantially lower than the estimated value of 107 kJ mol^−1^ required to enable configurationally stable molecules to be isolated. By contrast, oxonium ion **11** was only observed as a transient species, and we initially reasoned that this may have been a consequence of the tetrafluoroborate counterion functioning as a nucleophilic fluoride source (the Balz−Schiemann reaction). Anion exchange of the diazonium tetrafluoroborate salt derived from **9** to a less nucleophilic B(C_6_F_5_)_4_^−^ counterion and ring closure afforded **12**, but this compound slowly decomposed in solution at ambient temperature over 72 hours. We considered that the introduction of a second substituent onto the dibenzofuran−xanthene scaffold may have increased the strain in **11** and **12**, compromising the relative stability observed in **10**.

To enable us to rationalize the magnitude of the inversion barrier in **10** and the reactivity of more substituted compounds such as **11** we applied B3LYP-D3(BJ)/6-31+G(d,p) quantum chemical calculations (see page 33 of the Supplementary Information). We initially calculated barriers to the inversion of the oxonium ions based around the benzofuran−xanthene scaffold (Fig. 2c(i)). This gave excellent agreement with the experimental values for the overall geometry and barrier to pyramidal inversion for a model 2-methyl oxonium ion (R^2 ^= Me, R^3 ^= H: calculated at 59.0 kJ mol^−1^); other levels of theory and basis sets gave comparable quantitative results. These calculations also indicated that increasing the size of the substituent R^2^ would result in an increased inversion barrier (73.6 kJ mol^−1^ for R^2 ^= ^*i*^Pr, R^3 ^= H) and a marginal increase in pyramidalization about oxygen. Switching the (methyl) substitution to the xanthene portion gave predicted enantiomerization barriers similar to that previously calculated (R^2 ^= H, R^3 ^= Me, 54.4 kJ mol^−1^), but below the desired level for configurational stability. To investigate the impact of the addition of substituents on both rings, we examined a doubly alkyl-substituted oxonium (R^2 ^= R^3 ^= Me). This led to an increase in the predicted barrier to enantiomerization to 148.1 kJ mol^−1^ but also led to some changes in geometry that may explain the observed instability of molecules such as **11** and **12** at ambient temperature. The calculated sum of angles around the oxonium oxygen is 345.1° (R^2 ^= R^3 ^= Me), similar to the parent dibenzofuran oxonium ion, but we observe a notable increase in the external C–O–C angle to 129° and a reduction in apex height (*h* = 0.33 Å) consistent with a reduction in pyramidalization around oxygen. We also observe further lengthening of the longest C–O bond to 1.51 Å. This is a result of steric repulsion between the alkyl substituents, which leads to a twist in the xanthene ring and deformation from the boat-like conformation populated in **10**. Calculating a series of hyperhomodesmotic bond separation reaction enthalpies^44^ enabled us to estimate the increase in steric strain in going from a compound such as **10** (R^1 ^= H) to one bearing an extra alkyl group (R^1 ^= Me) as 16.7 kJ mol^−1^ (see page 34 of the Supplementary Information for details). For **11**, the calculated enthalpy difference versus **10** is significantly higher (45.6 kJ mol^−1^), probably indicating both an electronic and a steric contribution. We can conclude that the development of oxonium ions with large enough inversion barriers to attain configurational stability through the addition of multiple substituents on this ring system without compromising stability will probably be a challenge.

The design of configurationally stable molecules requires that we maximize the difference between ground states (GSs) and transition states (TSs). With a six-membered ring, our attempts to destabilize the TS (through installation of a larger R^3^ substituent) also results unavoidably in GS destabilization. Dihydrodibenzooxepines have a non-planar structure and we envisaged that the extra sp^3^ carbon in the seven-membered ring (versus xanthene) may enable the avoidance of steric destabilization in the GS. In contrast, we reasoned that as the molecule moves towards planarity in the TS, the extra sp^3^ carbon would actively lead to TS destabilization and a higher barrier to inversion.

We computed GS and TS structures for oxepin-containing oxonium ions (Fig. 2c(ii)). There is a substantial predicted increase in the barrier to inversion upon changing from the six to the seven-membered ring oxonium, from 59.0 to 117.0 kJ mol^−1^ (for R^2 ^= Me, R^3 ^= H) and this is augmented to 127.2 kJ mol^−1^ and 154.4 kJ mol^−1^ upon introduction of larger *iso*-propyl and *tert*-butyl groups respectively. In the calculated dihydrodibenzooxepine GS structures, the sum of angles about oxygen is generally lower than in the xanthene series and decreases as the size of the substituent on the benzofuran ring increases; this is consistent with a greater apex height in the seven-ring series and greater pyramidalization about oxygen.

**Fig. 3:**
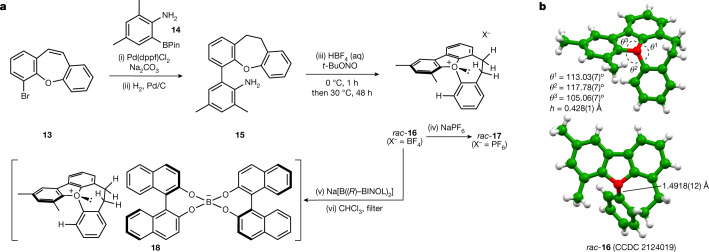
Synthesis and structural study of stereogenic-at-oxygen compounds. **a**, Reagents and conditions: (i) Pd(dppf)Cl_2_·CH_2_Cl_2_ (5 mol%), Na_2_CO_3_ (4 equivalents), PhMe/H_2_O (1:1, v/v), 95 °C, 24 h (67% yield); (ii) H_2_ (g, 1 atmosphere), Pd/C (2.5 mol%), EtOAc, RT, 16 h (quantitative); (iii) HBF_4_ (48% aq, 5 equvialents), *t*-BuONO (5 equivalents), CH_2_Cl_2_/IPA (1:1), 0 °C, 1 h then 30 °C, 48 h (75% yield); (iv) NaPF_6_ (aq), CH_2_Cl_2_ (56% yield); (v) Na[B((*R*)−BINOL)_2_] (1.0 equivalent), MeCN, RT then CH_2_Cl_2_, wash with water; (vi) dissolve in CHCl_3_ and modulate concentration. **b**. Crystal and molecular structure of fused pentacyclic oxonium salt *rac*-**16** from single-crystal X-ray diffraction studies. The tetrafluoroborate counterion is omitted for clarity. Numbers in parentheses indicate standard uncertainties. *h,* height of conceptualized triangular oblique pyramid with oxygen at its apex and the adjacent carbons of the attached O-aryl rings forming the base.

With this information in hand, we focussed on the synthesis of seven-ring-containing compounds, by using a strategically similar approach to that adopted earlier: dibenzooxepine **13** (made in four steps) was cross-coupled with aryl boronic ester **14** to afford a biaryl-containing intermediate in 67% yield. Hydrogenation gave **15**, which was diazotised under standard conditions and gently warmed to 30 °C to enable O-arylation and to afford the oxonium tetrafluoroborate *rac*-**16** in 75% yield (Fig. 3a). Counterion metathesis with sodium hexafluorophosphate enabled the synthesis of oxonium ion *rac*-**17** in 56% yield. Oxonium tetrafluoroborate *rac*-**16** and hexafluorophosphate *rac*-**17** are room-temperature stable solids that can be stored indefinitely without special precautions. The structure of *rac*-**16** was confirmed by single-crystal X-ray diffraction studies (space group *P−*1), which illustrate the expected puckered conformation of the oxepin ring and a pyramidal arrangement of atoms around the oxygen (the sum of the angles around oxygen is 335.9°, Fig. 3b). The apex height *h* = 0.428(1) Å, reflects the increased pyramidalization about oxygen versus **10**. The external C–O–C angle is 117.78(7)° and the internal oxepin C–O–C angle is 113.03(7)°; these constitute relatively minor angular deviations from those observed in the parent heterocycles and are consistent with a ring system that does not experience substantial angle strain. Computed hyperhomodesmotic reaction enthalpies suggest that oxonium ion **16** is less strained than **10** by 13.4 kJ mol^−1^. Examination of the unit cell does not show any short C–H or C–C distances less than normal van der Waals contact distances, which is indicative that the conformation of the oxonium cation is not influenced by crystal packing. The longest C–O bond length is 1.4918(12) Å; this is longer than the corresponding measurement in benzofuran-derived triaryloxonium ions (1.459(6) Å) and the average C–O bond length in triphenyloxonium tetraphenylborate (at 1.47 Å).

Treatment of *rac-***16** with a stoichiometric amount of sodium bis-(*R*)-BINOL borate led to complete conversion to a 1:1 mixture of diastereoisomeric salts (**18**, Fig. 3 and **19** (not shown)). Exploitation of the differential solubility of these two salts in chloroform enabled precipitation of one to yield the enriched diastereoisomeric pair **18** (ratio 94:6–99:1). We were unable to grow crystals suitable for X-ray analysis of diastereoisomerically enriched salt **18**, and hence we attempted to exchange the counterion in a highly diastereoisomerically enriched sample by stirring with an excess of tetrafluoroboric acid. From this mixture we were able to isolate crystals of the desired tetrafluoroborate salt **16**; this crystallized in *P*2_1_2_1_2_1_, with an absolute configuration of *P*-(*R*)_*O*_ (determined by refinement of the Flack parameter *x* against X-ray diffraction datasets collected from three separate crystals; CCDC 21911038-2191040)^45^, which thus also indicates the absolute configuration of the oxonium ion in **18** (see page 19 of the Supplementary Information). We probed the configurational stability of dichloromethane solutions of **18**, and observed a small change in the diastereoisomeric ratio over 7 days at 25 °C. With this data and the equilibrium ratio of 50.5:49.5 (**18**, *P*-(*R*)_*O*_, (*R*); **19**, *M*-(*S*)_*O*_,(*R*)), we can calculate the barriers to helical inversion to be 111.8 ± 0.1 kJ mol^−1^ (for the *P*-(*R*)_*O*_, (*R*) **18** to *M*-(*S*)_*O*_, (*R*) **19** conversion) and 111.7 ± 0.1 kJ mol^−1^ (for the *M*-(*S*)_*O*_, (*R*) **19** to *P*-(*R*)_*O*_, (*R*) **18** conversion); attempts to examine equilibration at higher temperatures (more than 50 °C) led to decomposition at a rate faster than equilibration. We were also able to resolve oxonium hexafluorophosphate *rac*-**17** by chiral stationary phase high-performance liquid chromatography, and use this method to determine its enantiomerization barrier as 111.0 ± 0.1 kJ mol^−1^. This demonstrates that the influence of the counterion on the magnitude of the inversion barrier is probably negligible, consistent with the observed values for diastereoisomeric salts **18** and **19** (which are almost energetically degenerate).

**Fig. 4:**
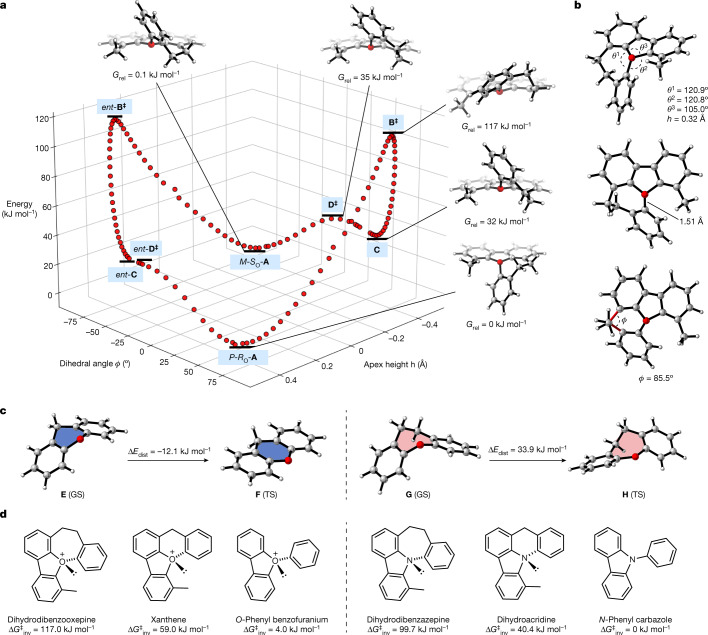
Mechanistic overview of enantiomerization of oxepin-containing oxonium ion. **a**, B3LYP-D3(BJ)/6-31+G(d,p) computed Gibbs energy profile for enantiomerization demonstrates that the process consists of a high barrier oxygen inversion and a low barrier conformational relaxation of the seven-membered ring. **b**, Geometrical measurements of TS **B**. *ϕ* = (C6–C7–C8–C9) dihedral angle. **c**, Computed change in ring strain (Δ*E*_dist_) in highlighted rings in xanthene and dihydrodibenzooxepine oxonium ions on going from GS to TS. The relative energies of truncated xanthene and dihydrodibenzooxepine substructures are computed at geometries taken from the parent oxonium ion. **d**, Comparison of computed barriers to inversion show that triaryloxonium ions have higher barriers to inversion than corresponding triaryl amines (B3LYP-D3(BJ)/6-31+G(d,p)). *G*_rel_ = relative Gibbs free energy. ^‡^ = transition state.

The experimentally determined barriers to inversion in **17** and **18** (111.0–111.8 kJ mol^−1^) are close to that predicted by calculation (117.0 kJ mol^−1^). A calculated reaction energy profile for the reversible enantiomerization of this oxonium ion is shown in Fig. 4a (ref. ^46^); this indicates that the enantiomerization process from *P*-(*R*)_*O*_ **A** consists of a high barrier helical inversion and a much lower barrier conformational relaxation associated with the seven-membered ring. The transition structure (TS) **B** for the helical inversion (*G*_rel _= 117 kJ mol^−1^) possesses inverted pyramidal geometry at the oxygen, albeit reduced from the GS **A** (*h* = 0.32 Å, sum of angles around oxygen 346.7° for **B** versus 335.9° for **A**). A structure in which the oxygen is trigonal planar lies earlier on the reaction coordinate from **A** to **B**; this also demonstrates that O-inversion is directly coupled with overall inversion of the helical chirality of the molecule. In TS **B**, both the internal oxepin and external C–O–C angles are close to 121° (Fig. 4b), which represents a notable change in structure around the oxygen atom versus the GS. Lengthening of the C–O bond in the oxepin ring versus the GS is also observed (1.51 versus 1.49 Å). TS **B** leads directly to intermediate **C** (*G*_rel _= 32 kJ mol^−1^) in which the oxepin ring populates a boat-like conformation; this undergoes a facile ring flip via TS **D** (*G*_rel _= 35 kJ mol^−1^) to the more stable boat-like conformation in GS structure *M*-(*S*)_*O*_ **A**. To rationalize why the inversion barrier of the oxepin-containing oxonium ion is greater than 40 kJ mol^−1^ higher than its xanthene-containing analogue, we calculated how ring strain in the six- and seven-membered rings in oxonium ions changes on going from the GS to the TS (Fig. 4c). In the xanthene-containing system, the boat-like GS **E** changes to an almost perfectly planar TS **F** through movement of the two aromatic rings into the same plane. This leads to a decrease in strain in the six-membered heterocycle of 12.1 kJ mol^−1^. This is owing to the relief of angle strain in the xanthene ring, which can be seen in the increase in internal C–O–C angle from 111.0° to 116.6° (GS to TS). In this system, the TS resembles the GS conformation of xanthene itself. In contrast, there is an increase in strain of 33.9 kJ mol^−1^ in the dihydrodibenzooxepine ring going from GS **G** to TS **H**. To attain the TS geometry demonstrated in **H**, the two aromatic rings twist to enable the inversion of the seven-membered ring. This raises the energy through a combination of torsional strain (between the two sp^3^-carbon atoms in the backbone (H–C–C–H torsion = 38.1°)), an increase in allylic strain between the backbone sp^3^ C–H bonds and the aromatic rings, internal angle strain and a minor deviation from planarity in the two aromatic rings. The seven-membered ring conformation in oxonium ion **G** is boat-like and resembles dihydrodibenzooxepine itself: deviation from this geometry in TS **H** raises the energy substantially. Delocalization of the oxygen lone pair changes along the reaction coordinate for inversion and this is also a prominent source of differentiation between dihydrodibenzooxepine and xanthene-derived oxonium ions. Natural bond orbital^47^ calculations highlight how oxygen lone-pair delocalization into the adjacent π systems stabilizes the inversion TS. For the xanthene oxonium ion, the increasing planarity about oxygen stabilizes the TS by 54.1 kJ mol^−1^ relative to the GS, whereas for the dihydrodibenzooxepine oxonium ion this is lower at 12.0 kJ mol^−1^, a difference of 33.1 kJ mol^−1^. Increased strain in the seven-membered ring oxonium backbone resists planarization in the inversion TS, reducing the extent of lone-pair delocalization, which consequently increases the activation energy barrier.

To examine how the identity of the heteroatom influences the inversion barrier, we studied this class of oxonium ions and their nitrogen analogues computationally (Fig. 4d). The calculated barriers to inversion of triaryloxonium ions are systematically higher than their triarylamine counterparts. This can be rationalized based on the resistance to rehybridization of the heteroatom, which is greater in the oxonium ion (see page 38 of the Supplementary Information). For the nitrogen analogues there is greater lone-pair delocalization in the inversion TS structures. This is consistent with calculations on the dihydrodibenzooxepine oxonium (Δ*G*^‡^_inv _= 117.0 kJ mol^−1^) and the dihydrodibenzazepine (Δ*G*^‡^_inv _= 99.7 kJ mol^−1^) systems, indicating that barrier height depends on both the geometric restraint provided by the ring structure and the identity of the heteroatom.

## Online content

Any methods, additional references, Nature Portfolio reporting summaries, source data, extended data, supplementary information, acknowledgements, peer review information; details of author contributions and competing interests; and statements of data and code availability are available at 10.1038/s41586-023-05719-z.

## Supplementary information

Supplementary Information

Crystallographic data.

## Data Availability

All data (experimental procedures, characterization data and cartesian coordinates for all DFT calculations) supporting the findings of this study are available within the article and its supplementary information. Crystallographic data for compounds **10**, *rac*-**16** and *P*-*(R)*_*O*_*-***16** (three datasets) have been deposited with the Cambridge Crystallographic data Centre under deposition numbers CCDC 2124020, CCDC 2124019 and CCDC 21911038–2191040. These data can be obtained free of charge from www.ccdc.cam.ac.uk/data_request/cif.
