# Supplementary material for: Control of stereogenic oxygen in a helically chiral oxonium ion
Source: Nature. 2023 Mar 15;615(7952):430–5. doi: 10.1038/s41586-023-05719-z (PMC10017494; doi:10.1038/s41586-023-05719-z)
Supplement: Supplementary file 1 — Supplementary Information [file 41586_2023_5719_MOESM1_ESM.pdf]

nature portfolio

https://doi.org/10.1038/s41586-023-05719-z

Supplementary information

Control of stereogenic oxygen in a helically chiral oxonium ion

In the format provided by the authors and unedited

Supporting Information for:

Control of Stereogenic Oxygen in a Helically Chiral Oxonium Ion

Owen Smith[1], Mihai V. Popescu[1,2], Madeleine Hindson[1],
Robert Paton[2]*, Jonathan W. Burton[1]* and Martin D. Smith[1]*

[1] Chemistry Research Laboratory, University of Oxford, 12 Mansfield Road, Oxford, OX1 3TA, UK.
[2] Department of Chemistry, Colorado State University, 1301 Center Ave, Ft. Collins, CO 80523-1872, USA.

Contents

1 General Information 2
2 Experimental Procedures for the synthesis of oxonium ions 3
2.1 Xanthene-derived oxonium ion synthesis 3
2.2 Dibenzodidehydrooxepane derived oxonium ion synthesis 9
3 Experimental data for resolution of *rac*-**16** 14
4 X-ray Crystallography 19
4.1 1,3,6-Trimethyl-8*H*-benzofuro[3,2,1-*de*]xanthen-13-ium tetrafluoroborate, **10** 20
4.2 11,13-Dimethyl-4,5-dihydrobenzo[*b*]benzo[6,7]oxepino[3,2,1-*hi*] benzofuran-10-ium tetrafluoroborate, *rac*-**16** 21
4.3 11,13-Dimethyl-4,5-dihydrobenzo[*b*]benzo[6,7]oxepino[3,2,1-*hi*] benzofuran-10-ium tetrafluoroborate, *enantioenriched*-**16** 22
5 Inversion Barrier Measurements 23
5.1 1,3,6-Trimethyl-8*H*-benzofuro[3,2,1-*de*]xanthen-13-ium tetrafluoroborate, **10** 23
5.2 Equilibration of 11,13-Dimethyl-4,5-dihydrobenzo[*b*]benzo[6,7]oxepino[3,2,1-*hi*]benzofuran-10-ium, [*P*, $(R)_O$]-bis-(*R*)-BINOL borate, **18** 25
5.3 Enantiomeric ratio erosion study of 11,13-dimethyl-4,5-dihydrobenzo[b]benzo[6,7]oxepino[3,2,1-hi]benzofuran-10-ium hexafluorophosphate, enantioenriched-[*M*, $(S)_O$]-**17** 30
6 Computational Studies 33
6.1 Computational details 33
6.2 Hyperhomodesmotic bond separation reactions 35
6.3 NBO analysis on ring size influence 37
6.4 Comparison between triaryl oxonium ions and triaryl amines 38
6.5 Thermochemical data 40
6.6 Cartesian coordinates for stationary points 45
7 NMR Spectra 88
8 References 112

1 General Information

Reagents were purchased from major commercial suppliers and were used without further purification. Anhydrous reaction solvents (CH_2Cl_2, 1,4-dioxane, DMF, THF, PhMe, Et_3N, *i*-Pr_2NH) were obtained from an MBraun SPS5 solvent purification system having been dried over activated alumina columns. All other solvents were used without prior drying. Unless specified, the synthetic reactions were carried out without using dried glassware or an inert atmosphere. The glassware for reactions conducted using an inert atmosphere was flame-dried under vacuum and then placed under N_2 unless H_2O was used as a solvent in the procedure. Solvents were degassed by sparging with Argon for 10 minutes as stated in the procedures. Reactions were heated using a DrySyn® heating block and contact thermometer. Room Temperature (r.t.) is defined as 20-25 °C.

Reactions were monitored by Thin Layer Chromatography (TLC) using silica gel on pre-coated 0.25 mm aluminium sheets (Merck Kieselgel 60 F_{254}) and visualized with UV irradiation (λ = 254 nm) followed by a basic $KMnO_4$ solution. Flash column chromatography was carried out on Silica Gel 60 (0.043-0.063 nm, VWR) under pressure (0.2-0.4 bar) from the house N_2 supply. A UltrawaveU1250D Ultrasonic water bath (50-60 Hz) was used for sonication.

NMR spectra were recorded on Bruker AVIII/AVIIIHD (700, 600, 500 and 400 MHz (^{1}H)) and Bruker NEO (600 MHz (^{1}H)) spectrometers in the deuterated solvent specified. NMR data are reported as chemical shifts (δ) and quoted in parts per million (ppm). ^{1}H NMR were referenced to the non-deuterated residual solvent peak, ^{13}C NMR were referenced to the residual solvent peak and ^{19}F NMR were referenced externally to $CFCl_3$ = 0 ppm. Chemical shifts are quoted in ppm with splitting assigned as singlet (s), doublet (d), triplet (t), quartet (q), pentet (p), septet (sept), and multiplet (m) or combinations of the above. The abbreviation br is used to denote broad signals. Coupling constants, *J*, are measured to the nearest 0.1 Hz and are presented as observed. NMR Spectra were processed using MestReNova 11.0 and where spectra are assigned, this was carried out with the aid of COSY, HSQC and HMBC experiments.

Melting points (M.P.) were determined using a Reichert melting point apparatus and are uncorrected. Unless otherwise stated, the solid material analysed was obtained from *in vacuo* removal of the solvent system used for flash column chromatography. Fourier Transform Infra-red spectra were recorded on a Bruker Tensor 27 FTIR spectrometer equipped with a diamond ATR module. Absorption maxima (ν_{max}) are reported in wavenumbers (cm^{-1}) for the range 3500 – 600 cm^{-1}. Low-resolution mass spectra were recorded on a Micro Mass LCT Premier spectrometer under conditions of electrospray ionization (ESI). High resolution mass spectra using electrospray ionization (ESI) were recorded on a Thermo Exactive orbitrap spectrometer equipped with a Waters Acquity LC system. High resolution mass spectra using atmospheric pressure chemical ionization (APCI) or electron ionization (EI) were recorded on an Agilent 7200 Accurate Mass Q-TOF GC-MS spectrometer connected to a 7890 GC system DIP solid/liquid state thermal probe and a CTC Analytics HTS PAL Sample Manager. Optical rotations were recorded on a Schmidt-Haensch Unipol L2000 polarimeter at 25 °C using 589 nm radiation. Concentrations are quoted in g/100 mL and $[\alpha]_D^{25}$ values are quoted in [° mL g^{-1} dm^{-1}]. The absolute configuration of enantioenriched **16** was confirmed by x-ray crystallography; see **Section 4** for further information. Compound names were

generated using ChemDraw 20.1.1 in accordance with the guidelines stipulated by the International Union of Pure and Applied Chemistry (IUPAC).

2 Experimental Procedures for the synthesis of oxonium ions

2.1 Xanthene-derived oxonium ion synthesis

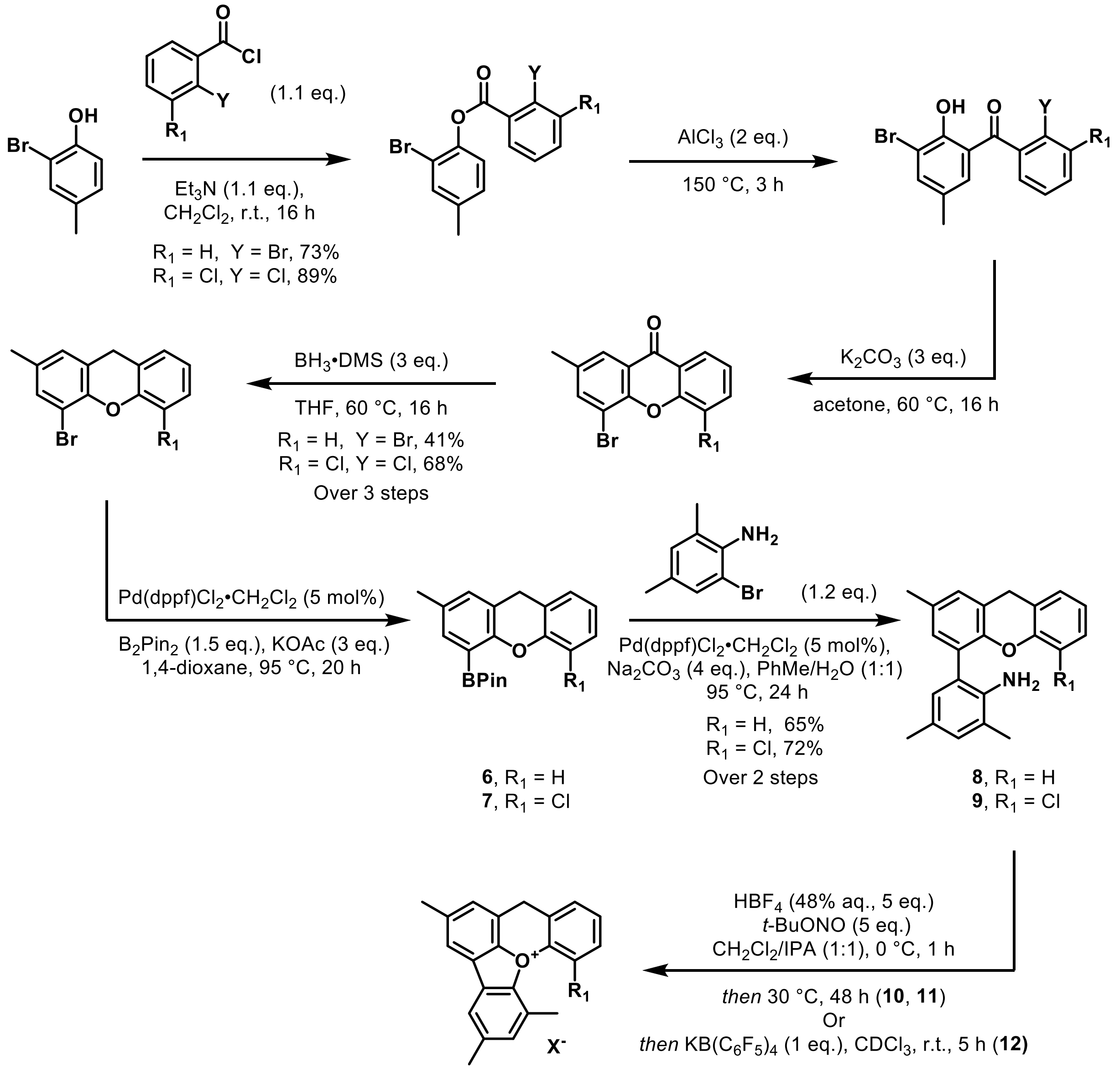

10, R_1 = H, X = BF_4^-, 79%
11, R_1 = Cl, X = BF_4^-
12, R_1 = Cl, X = $B(C_6F_5)_4^-$

Figure S1. Synthetic approach to xanthene-derived oxonium ions

2-Bromo-4-methylphenol, S1:

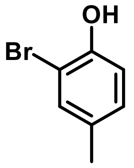

Bromine (2.37 mL, 7.39 g, 46.00 mmol, 1 eq.) in $CHCl_3$ (20 mL) was added *via* pressure equalising dropping funnel to a solution of *p*-cresol (5.00 g, 46.00 mmol, 1 eq.) in $CHCl_3$ (40 mL) at 0 °C. After 1 h, the organic layer was washed (Na_2SO_3, sat. aq., 30 mL), dried ($MgSO_4$) and concentrated *in vacuo* to give the title compound as a pale yellow oil (7.53 g, 87%). **^{1}H NMR** (400 MHz, $CDCl_3$): δ_H = 7.29 – 7.28 (m, 1H), 7.04 – 7.00 (m, 1H), 6.94 (d, *J* = 8.2 Hz, 1H), 5.58 (s, 1H), 2.28 (dd, *J* = 0.8 Hz, 0.8Hz, 3H). **^{13}C NMR** (101 MHz, $CDCl_3$): δ_C = 150.1, 132.3, 131.5, 129.9, 116.0, 110.0, 20.3. **FTIR** (neat) ν_{max}/cm^{-1} = 3503, 2922, 2360, 1609, 1494, 1407, 1330, 1283, 1252,1210, 1180, 1041, 867, 814, 761, 671. **HRMS** (EI^+): m/z calculated for $C_7H_7{}^{79}BrO^+$, $[M]^+$= 185.9680, m/z found = 185.9675. Δ = -2.69 ppm. *Spectra are consistent with literature values.[1]

2-Bromo-4-methylphenyl 2-bromobenzoate, S2:

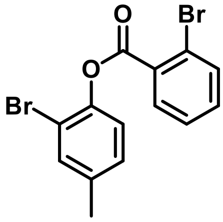

Triethylamine (1.53 mL, 1.11 g, 11.00 mmol, 1.1 eq.) was added to a solution of 2-bromo-4-methylphenol, **S1**, (1.86 g, 10.00 mmol, 1 eq.) in CH_2Cl_2 (20 mL) at 0 °C under N_2. After 5 mins, 2-bromobenzoylchloride (1.40 mL, 2.41 g, 11.00 mmol, 1.1 eq.) was added and the reaction was warmed to r.t.. After 16 h, the reaction mixture was sequentially washed with HCl (3 M aq., 10 mL) and $NaHCO_3$ (sat. aq., 30 mL). The organics were dried ($MgSO_4$), concentrated *in vacuo* and the crude residue was purified by flash column chromatography (Et_2O:pentane, 5:95, R_f = 0.25) to give the title compound as a white solid (3.15 g, 73%). **M.P.** = 52-54 °C. **^{1}H NMR** (500 MHz, $CDCl_3$): δ_H = 8.17 (dd, *J* = 7.6, 1.9 Hz, 1H), 7.75 (dd, *J* = 7.8, 1.4 Hz, 1H), 7.48 (d, *J* = 1.3 Hz, 1H), 7.47 – 7.40 (m, 2H), 7.19 – 7.17 (m, 2H), 2.37 (s, 3H). **^{13}C NMR** (126 MHz, $CDCl_3$): δ_C = 163.6, 145.9, 137.9, 134.8, 133.9, 133.5, 132.3, 130.8, 129.4, 127.5, 123.5, 122.8, 115.8, 20.8. **FTIR** (neat) ν_{max}/cm^{-1} = 2981, 2360, 1754, 1589, 1487, 1432, 1382, 1224, 1199, 1132, 1079, 1021, 954, 867, 806, 741, 683, 643. **HRMS** (ESI^+): *m/z* calculated for $C_{14}H_{10}{}^{79}Br_2NaO_2{}^+$, $[M+Na]^+$ = 390.8940, *m/z* found = 390.8939. Δ = - 0.26 ppm.

4-Bromo-2-methyl-9*H*-xanthene, S3:

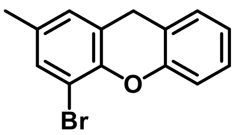

Xanthene **S3** was prepared according to a modified literature procedure.[2] 2-Bromo-4-methylphenyl 2-bromobenzoate, **S2**, (2.00 g, 5.40 mmol, 1 eq.) and $AlCl_3$ (1.44 g, 10.80 mmol, 2.00 eq.) were heated to 150 °C under N_2. After 3 h, the reaction was cooled to r.t. and then 0 °C HCl (6 M, aq., 40 mL) was added and decanted. The remaining residue was sonicated with H_2O/Et_2O (5 × 10 mL/10 mL) until fully dissolved. All fractions were combined, extracted with Et_2O (2 × 50 mL), dried ($MgSO_4$) and concentrated *in vacuo*. The crude solid was dissolved in acetone (20 mL), K_2CO_3 (2.24 g, 16.21 mmol, 3 eq.) was added and the reaction was heated to 60 °C. After 16 h, the acetone was removed *in vacuo*. H_2O (50 mL) was added and the mixture was extracted with Et_2O (3 × 50 mL), dried ($MgSO_4$) and concentrated *in vacuo*. BH_3·DMS (1.54 mL, 1.23 g, 16.20 mmol, 3 eq.) was added to a solution of the crude residue in THF (20 mL) under N_2 and the reaction was heated to 60 °C. After 16 h, MeOH (5 mL) was slowly added to the reaction. The solvent was removed by a steady stream of N_2 and the crude residue was purified by flash column chromatography (pentane, R_f = 0.3) to give the title

compound as a pale yellow solid (605 mg, 41%). **M.P.** = 125-128 °C. **^{1}H NMR** (500 MHz, $CDCl_3$): δ_H = 7.25 (s, 1H), 7.23 – 7.18 (m, 1H), 7.18 – 7.12 (m, 2H), 7.04 (td, *J* = 7.4, 1.4 Hz, 1H), 6.91 (s, 1H), 4.01 (s, 2H), 2.28 (s, 3H). **^{13}C NMR** (126 MHz, $CDCl_3$): δ_C = 152.0, 146.8, 133.5, 131.9, 128.8, 128.6, 127.9, 123.5, 122.2, 120.5, 117.0, 110.4, 28.4, 20.5. **FTIR** (neat) ν_{max}/cm^{-1} = 2898, 1663, 1613, 1596, 1469, 1411, 1314, 1271, 1238, 1196, 1110, 1039, 929, 891, 843, 755, 713, 647. **HRMS (ESI$^+$):** *m/z* calculated for $C_{14}H_{10}{}^{79}BrO^+$, $[M-H]^+$ = 272.9910; *m/z* found = 272.9911. Δ = 0.61 ppm.

2,4-Dimethyl-6-(2-methyl-9*H*-xanthen-4-yl)aniline, 8:

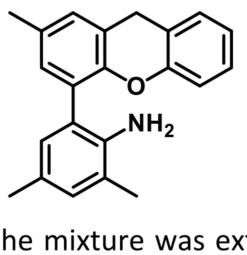

1,4-Dioxane (10 mL) was added to a mixture of 4-bromo-2-methyl-9*H*-xanthene, **S3**, (280 mg, 1.02 mmol, 1 eq.), B_2Pin_2 (387 mg, 1.53 mmol, 1.5 eq.), Pd(dppf)Cl_2·CH_2Cl_2 (24.9 mg, 0.03 mmol, 3 mol%) and KOAc (300 mg, 3.05 mmol, 3 eq.) under N_2. Argon was bubbled through the solution for 10 minutes and the reaction was heated to 95 °C. After 20 h, H_2O (10 mL) was added and the mixture was extracted with CH_2Cl_2 (3 × 10 mL). The organic extracts were dried ($MgSO_4$) and concentrated *in vacuo*. The crude residue was suspended in pentane (15 mL), filtered through Celite® (2 mL), eluted (pentane, 3 × 5 mL) and concentrated *in vacuo*.[3] The crude solid was added as a solution in PhMe (5 mL) to a suspension of 2-bromo-4,6-dimethylaniline (245 mg, 1.22 mmol, 1.2 eq.), Pd(dppf)Cl_2·CH_2Cl_2 (41.6 mg, 0.05 mmol, 5 mol%) and Na_2CO_3 (428 mg, 4.08 mmol, 4 eq.) in H_2O (5 mL) under N_2. Argon was bubbled through the solution for 10 mins and the reaction was heated to 95 °C. After 24 h, the reaction was extracted with CH_2Cl_2 (3 × 10 mL). The organic extracts were dried ($MgSO_4$), concentrated *in vacuo* and the crude residue was purified by flash column chromatography (Et_2O:pentane, 5:95, R_f = 0.2) to give the title compound as a tan solid (210 mg, 65%). **M.P.** = 118-119 °C. **^{1}H NMR** (400 MHz, $CDCl_3$): δ_H = 7.23 – 7.13 (m, 2H), 7.07 – 7.02 (m, 3H), 7.01 – 6.94 (m, 2H), 6.91 (d, *J* = 2.1 Hz, 1H), 4.09 (s, 2H), 3.74 (br s, 2H), 2.37 (s, 3H), 2.33 (s, 3H), 2.29 (s, 3H). **^{13}C NMR** (126 MHz, $CDCl_3$): δ_C = 152.3, 147.3, 139.8, 132.6, 130.7, 130.7, 129.5, 128.8, 128.7, 127.5, 127.5, 127.3, 124.2, 123.1, 122.8, 121.4, 121.2, 117.0, 28.5, 20.7, 20.6, 18.1. **FTIR** (neat) ν_{max}/cm^{-1} = 3384, 2917, 1620, 1584, 1484, 1456, 1292, 1265, 1239, 1209, 1189, 1154, 1098, 1033, 1012, 908, 879, 860, 827, 755, 730, 646. **HRMS** (ESI$^+$): *m/z* calculated for $C_{22}H_{22}NO^+$, $[M+H]^+$ = 316.1696, *m/z* found = 316.1697. Δ = 0.32 ppm.

1,3,6-Trimethyl-8*H*-benzofuro[3,2,1-*de*]xanthen-13-ium tetrafluoroborate, 10:

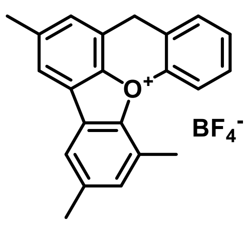

t -BuONO (94 µL, 81 mg, 0.79 mmol, 5 eq.) was added to a solution of 2,4-dimethyl-6-(2-methyl-9*H*-xanthen-4-yl)aniline, **8**, (50 mg, 0.16 mmol, 1 eq.) and HBF_4 (48% aq., 103 µL, 145 mg, 0.79 mmol, 5 eq.) in CH_2Cl_2:IPA (1:1, 2 mL) at 0 °C. After 1 h, the reaction mixture was diluted with CH_2Cl_2 (3 mL), washed with H_2O (1 mL) and heated to 30 °C. After 48 h, the solvent was removed by a steady stream of N_2 and Et_2O (3 mL) was added. The Et_2O layer was passed through a Celite® filter (1 mL). The solid precipitate was washed and the solvent passed through the Celite® (Et_2O, 4 x 1 mL). The solid precipitate was dissolved in MeCN (2 mL), passed through the Celite® and eluted (MeCN, 3 × 2 mL). The solvent was removed by a steady stream of N_2 to give the title compound as a white solid (48 mg, 79%). **M.P.** = 157 °C (MeCN, dec.). **^{1}H NMR** (400 MHz, CD_3CN): δ_H = 7.96 – 7.91 (m, 1H), 7.83 (dd, *J* = 1.7, 0.9 Hz, 1H), 7.82 – 7.78 (m, 1H), 7.64 – 7.58 (m,

2H), 7.58 – 7.52 (m, 3H), 4.40 (br s, 2H), 2.62 (s, 3H), 2.58 (s, 3H), 2.54 (s, 3H). **^{13}C NMR** (126 MHz, CD_3CN): δ_C = 159.6, 156.4, 155.2, 143.4, 142.9, 134.6, 132.7, 131.1, 129.9, 129.9, 127.7, 125.7, 124.4, 123.5, 122.0, 121.7, 115.1, 29.9, 21.6, 21.3, 18.4. One quaternary carbon was not observed. **^{19}F NMR** (377 MHz, CD_3CN): δ_F = -151.8. **FTIR** (neat) ν_{max}/cm^{-1} = 1475, 1452, 1386, 1285, 1171, 1051, 1036, 854, 825, 786, 766, 734, 700, 670, 644. **HRMS** (ESI$^+$): *m/z* calculated for $C_{22}H_{19}O^+$, $[M+H]^+$ = 299.1430, *m/z* found = 299.1432. Δ = 0.67 ppm.

2-Bromo-4-methylphenyl 2,3-dichlorobenzoate, S4:

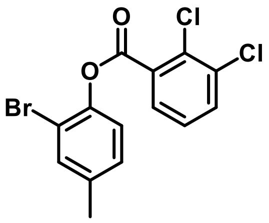

DMF (5 drops) was added to a solution of 2,3-dichlorobenzoic acid (1.50 g, 7.85 mmol, 1 eq.) and oxalyl chloride (0.797 mL, 1.20 g, 9.42 mmol, 1.2 eq.) in CH_2Cl_2 (40 mL) at r.t.. After 2 h, the solvent was removed *in vacuo*. Triethylamine (1.23 mL, 880 mg, 8.64 mmol, 1.1 eq.) was added to a solution of 2-bromo-4-methylphenol, **S1**, (1.46 g, 7.85 mmol, 1 eq.) and the crude residue in CH_2Cl_2 (40 mL) under N_2 at 0 °C and the reaction was allowed to warm to r.t.. After 16 h, the reaction mixture was sequentially washed with HCl (3 M aq., 10 mL) and $NaHCO_3$ (sat. aq., 30 mL). The organics were dried ($MgSO_4$), concentrated *in vacuo* to give the title compound as a white solid (2.52 g, 89%). **M.P.** = 103-105 °C. **^{1}H NMR** (400 MHz, $CDCl_3$): δ_H = 7.95 (dd, *J* = 7.8, 1.6 Hz, 1H), 7.59 (dd, *J* = 8.0, 1.6 Hz, 1H), 7.39 (dt, *J* = 1.4, 0.8 Hz, 1H), 7.27 (t, *J* = 7.9 Hz, 1H), 7.12 – 7.06 (m, 2H), 2.28 (s, 3H). **^{13}C NMR** (101 MHz, $CDCl_3$): δ_C = 162.8, 145.8, 138.1, 135.1, 134.1, 133.9, 132.6, 131.5, 130.0, 129.4, 127.4, 123.4, 115.7, 20.8. **FTIR** (neat) ν_{max}/cm^{-1} = 2922, 1747, 1693, 1579, 1492, 1414, 1311, 1279, 1244, 1197, 1155, 1124, 1097, 1046, 889, 867, 808, 743, 711, 687, 671. **HRMS** (APCI$^+$): *m/z* calculated for $C_{14}H_{10}{}^{79}Br^{35}Cl_2O_2^+$, $[M+H]^+$ = 358.9236, *m/z* found = 358.9235. Δ = -0.28 ppm.

4-Bromo-5-chloro-2-methyl-9*H*-xanthene, S5:

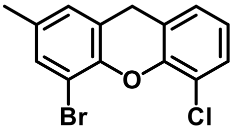

Xanthene **S5** was prepared according to a modified literature procedure.[2] 2-Bromo-4-methylphenyl 2,3-dichlorobenzoate, **S4**, (1.80 g, 5.00 mmol, 1 eq.) and $AlCl_3$ (1.33 g, 10.00 mmol, 2 eq.) were heated to 150 °C under N_2. After 3 h, the reaction was cooled to r.t. and then 0 °C. HCl (6 M, aq., 40 mL) was added and decanted. The remaining residue was sonicated with H_2O/Et_2O (5 × 10 mL/10 mL) until fully dissolved. All fractions were combined, extracted with Et_2O (2 × 50mL), dried ($MgSO_4$) and concentrated *in vacuo*. The crude solid was dissolved in acetone (20 mL), K_2CO_3 (2.07 g, 15.00 mmol, 3 eq.) was added and the reaction was heated to 60 °C. After 16 h, the acetone was removed *in vacuo*. H_2O (50 mL) was added and the mixture was extracted with Et_2O (3 × 50 mL), dried ($MgSO_4$) and concentrated *in vacuo*. $BH_3 \cdot DMS$ (1.42 mL, 1.14 g, 15.00 mmol, 3 eq.) was added to a solution of the crude residue in THF (20 mL) under N_2 and the reaction was heated to 60 °C. After 16 h, MeOH (5 mL) was slowly added to the reaction. The solvent was removed by a steady stream of N_2 and the crude residue was purified by flash column chromatography (pentane, R_f = 0.3) to give the title compound as a pale yellow solid (1.05 g, 68%). **M.P.** = 111-113 °C. **^{1}H NMR** (400 MHz, $CDCl_3$): δ_H = 7.32 – 7.26 (m, 2H), 7.07 (ddt, *J* = 7.6, 1.8, 1.0 Hz, 1H), 6.98 (t, *J* = 7.7 Hz, 1H), 6.94 – 6.89 (m, 1H), 4.03 (s, 2H), 2.31 (s, 3H). **^{13}C NMR** (126 MHz, $CDCl_3$): δ_C = 148.0, 146.5, 134.2, 132.1, 128.7, 128.3, 127.0, 123.6, 122.4, 122.3, 121.8, 110.8, 28.6, 20.5. **FTIR** (neat) ν_{max}/cm^{-1} = 2918, 1589, 1567, 1457,

1377, 1322, 1302, 1265, 1251, 1235, 1216, 1181, 1146, 1128, 1064, 919, 875, 847, 831, 761, 715, 697, 624. **HRMS** (ESI$^-$): *m/z* calculated for $C_{14}H_9{}^{79}Br^{35}ClO^-$, $[M-H]^-$ = 306.9520, *m/z* found = 306.9521. Δ = 0.33 ppm.

2-(5-Chloro-2-methyl-9*H*-xanthen-4-yl)-4,6-dimethylaniline, 9:

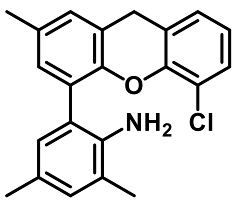

1,4-Dioxane (10 mL) was added to a mixture of 4-bromo-5-chloro-2-methyl-9*H*-xanthene, **S5**, (248 mg, 0.80 mmol, 1 eq.), B_2Pin_2 (305 mg, 1.20 mmol, 1.5 eq.), $Pd(dppf)Cl_2{\cdot}CH_2Cl_2$ (20.0 mg, 0.02 mmol, 3 mol%) and KOAc (235 mg, 2.40 mmol, 3 eq.) under N_2. Argon was bubbled through the solution for 10 minutes and the reaction was heated to 95 °C. After 20 h, H_2O (10 mL) was added and the mixture was extracted with CH_2Cl_2 (3 × 10 mL). The organic extracts were dried ($MgSO_4$) and concentrated *in vacuo*. The crude residue was suspended in pentane (15 mL), filtered through Celite® (2 mL), eluted (pentane, 3 × 5 mL) and concentrated *in vacuo*.[3] The crude solid was added as a solution in PhMe (5 mL) to a suspension of 2-bromo-4,6-dimethylaniline (194 mg, 0.96 mmol, 1.2 eq.), $Pd(dppf)Cl_2{\cdot}CH_2Cl_2$ (32.6 mg, 0.04 mmol, 5 mol%) and Na_2CO_3 (336 mg, 3.20 mmol, 4 eq.) in H_2O (5 mL) under N_2. Argon was bubbled through the solution for 10 mins and the reaction was heated to 95 °C. After 24 h, the reaction was extracted with CH_2Cl_2 (3 × 10 mL). The organic extracts were dried ($MgSO_4$), concentrated *in vacuo* and the crude residue was purified by flash column chromatography (Et_2O:pentane, 10:90, R_f = 0.2) to give the title compound as a viscous yellow oil (202 mg, 72%).

^{1}H NMR (500 MHz, $CDCl_3$): δ_H = 7.20 (dd, *J* = 7.9, 1.5 Hz, 1H), 7.10 – 7.05 (m, 2H), 6.99 (d, *J* = 2.0 Hz, 1H), 6.95 – 6.91 (m, 2H), 6.90 (d, *J* = 2.1 Hz, 1H), 4.05 (s, 2H), 3.53 (s, 2H), 2.34 (s, 3H), 2.28 (s, 3H), 2.23 (s, 3H). **^{13}C NMR** (126 MHz, $CDCl_3$): δ_C = 148.3, 146.8, 140.2, 133.5, 130.8, 130.7, 129.6, 128.5, 128.4, 128.0, 127.1, 127.0, 123.5, 123.2, 123.1, 122.9, 122.2, 121.0, 28.7, 20.8, 20.6, 18.1. **FTIR** (neat) ν_{max}/cm^{-1} = 2917, 1621, 1454, 1250, 1207, 1184, 1146, 909, 859, 767, 737, 714, 635. **HRMS** (ESI$^+$): *m/z* calculated for $C_{22}H_{21}{}^{35}ClNO^+$, $[M+H]^+$ = 350.1306, *m/z* found = 350.1307. Δ = 0.29 ppm.

12-Chloro-1,3,6-trimethyl-8*H*-benzofuro[3,2,1-*de*]xanthen-13-ium tetrakis(perfluorophenyl)borate, 12:

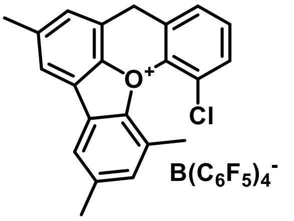

t -BuONO (59 µL, 52 mg, 0.50 mmol, 5 eq.) was added to a solution of 2-(5-chloro-2-methyl-9*H*-xanthen-4-yl)-4,6-dimethylaniline, **9**, (35 mg, 0.10 mmol, 1 eq.) and HBF_4 (48% aq., 65 µL, 91 mg, 0.50 mmol, 5 eq.) in CH_2Cl_2:IPA (1:1, 2 mL) at 0 °C. After 1 h, the reaction mixture was diluted with CH_2Cl_2 (3 mL) and washed with H_2O (1 mL). The organics were removed by a steady stream of N_2, Et_2O (5 mL) was added and the precipitated diazonium salt was collected by suction filtration. $KB(C_6F_5)_4$ (44 mg, 0.06 mmol, 1 eq.) was added to a solution of the precipitated diazonium salt (26 mg, 0.06 mmol, 1 eq.) in $CDCl_3$ (0.5 mL) at r.t.. After 30 mins, the reaction was filtered through a Celite® filter (0.2 mL) and eluted with $CDCl_3$ (3 × 0.5 mL). After 5 h, the reaction mixture was subjected to NMR analysis. **^{1}H NMR** (500 MHz, $CDCl_3$): δ_H = 7.70 (d, *J* = 1.9 Hz, 1H), 7.62 (s, 1H), 7.52 (d, *J* = 7.3 Hz, 1H), 7.43 (t, *J* = 7.5 Hz, 1H), 7.36 (s, 1H), 7.33 (s, 1H), 7.31 – 7.27 (m, 1H), 4.41 (d, *J* = 17.7 Hz, 1H), 4.15 (d, *J* = 17.7 Hz, 1H), 2.55 (s, 3H), 2.54 (s,

3H), 2.52 (s, 3H). **^{13}C NMR** (126 MHz, $CDCl_3$): δ_C = 160.4, 158.0, 156.5, 143.0, 142.5, 134.7, 132.8, 130.5, 130.1, 128.3, 128.3, 126.2, 124.4, 124.0, 123.0, 122.3, 121.0, 120.3, 30.5, 21.7, 21.4, 16.0. **^{19}F NMR** (377 MHz, $CDCl_3$): δ_F = -131.9 – -133.1 (m), -162.8 (t, *J* = 21.3 Hz), -166.5 – -167.0 (m). **FTIR** (neat) ν_{max}/cm^{-1} = 1643, 1513, 1461, 1275, 1086, 978, 909, 853, 775, 756, 734, 684, 661.

2.2 Dibenzodidehydrooxepane derived oxonium ion synthesis

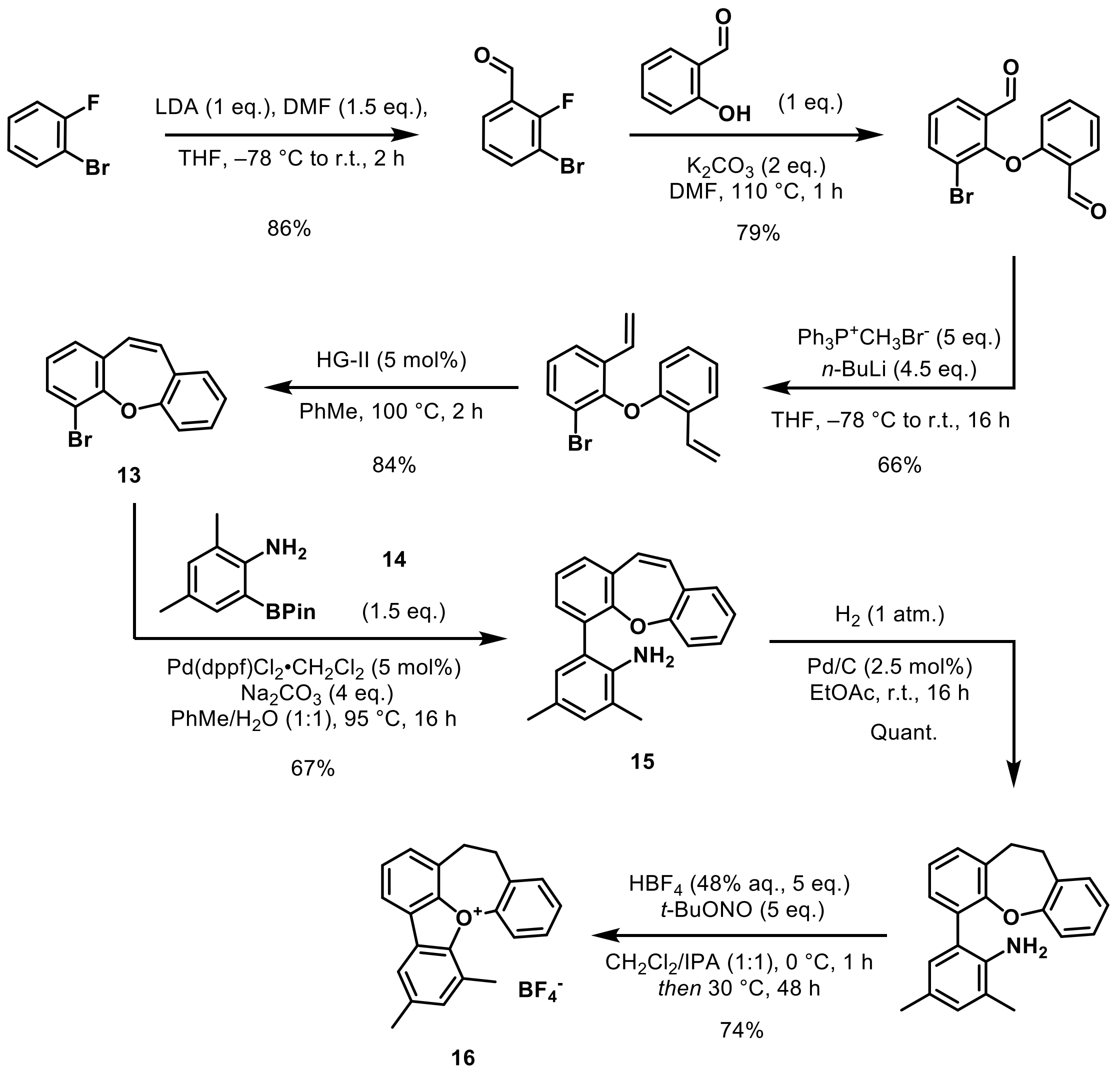

Figure S2. Synthetic approach to dihydrobenzooxepine-derived oxonium ions

3-bromo-2-fluorobenzaldehyde, S6:

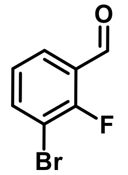

Aldehyde **S6** was prepared according to a literature procedure.[4,5] *n*-BuLi (1.6 M, 7.5 mL, 12.00 mmol, 1.2 eq.) was added to a stirred solution of diisopropylamine (1.82 mL, 1.32 g, 13.00 mmol, 1.3 eq.) in THF (10 mL) at –78 °C under N_2. After 30 mins, 1-fluoro-2-bromobenzene (1.09 mL, 1.75 g, 10.00 mmol, 1 eq.) was added dropwise and the resulting solution was stirred at –78 °C for 1 h. DMF (1.16 mL, 1.10 g, 15.00 mmol, 1.5 eq.) was added and, after 10 mins at –78 °C, the reaction mixture was warmed to r.t.. After 1 h, water (50 mL) was added and the reaction mixture was extracted with EtOAc (3 × 15 mL). The organic extracts were dried ($MgSO_4$), concentrated *in vacuo* and purified by flash column chromatography (Et_2O:Pentane, 5:95, R_f = 0.3) to give the title compound as a white solid (1.75 g, 86%). **M. P.** = 40-41 °C. **^{1}H NMR** (400 MHz, $CDCl_3$): δ_H = 10.30 (d, *J* = 0.8 Hz, 1H), 7.78 (d, *J* = 7.9 Hz, 1H), 7.77 (d, *J* = 7.8 Hz, 1H), 7.15 (tt, *J* = 7.9, 0.9 Hz, 1H). **^{13}C NMR** (101 MHz, $CDCl_3$): δ_C = 186.2 (d, *J* = 6.1 Hz), 161.0 (d, *J* = 259.1 Hz), 139.5 (d, *J* = 1.5 Hz), 127.8 (d, *J* = 1.6 Hz), 125.7 (d, *J* = 4.6 Hz), 125.3 (d, *J* = 9.0 Hz), 110.3 (d, *J* = 20.3 Hz). **^{19}F NMR (377 MHz, $CDCl_3$): δ_F** = -116.1. **FTIR (neat) ν_{max}/cm^{-1}** = 2360, 1715, 1694, 1601, 1575, 1451, 1398, 1249, 1172, 1127, 855, 782, 709, 661. **HRMS:** Mass not found by ESI, APCI or EI.

3-bromo-2-(2-formylphenoxy)benzaldehyde, S7:

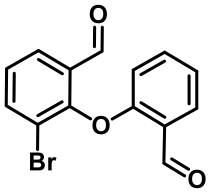

3-Bromo-2-fluorobenzaldehyde, **S6**, (3.00 g, 11.40 mmol, 1 eq.), salicylaldehyde (1.21 mL, 1.39 g, 11.40 mmol, 1 eq.) and K_2CO_3 (3.15 g, 22.80 mmol, 2 eq.) were added to DMF (15 mL) and heated to 110 °C under N_2. After 24 h, H_2O (100 mL) was added and the reaction mixture was extracted with Et_2O (3 × 60 mL). The combined organic layers were washed with H_2O (100 mL), dried ($MgSO_4$) and concentrated *in vacuo*. The crude residue was purified by flash column chromatography (Et_2O:pentane, 15:85. R_f = 0.3) to give the title compound as a white solid (2.72 g, 79%). **M.P.** = 100-102 °C. **^{1}H NMR** (400 MHz, $CDCl_3$): δ_H = 10.76 (s, 1H), 10.20 (s, 1H), 8.01 – 7.92 (m, 3H) 7.44 (ddd, *J* = 8.5, 7.5, 2.0 Hz, 1H), 7.36 (t, *J* = 8.0 Hz, 1H), 7.19 (t, *J* = 7.5 Hz, 1H), 6.45 (d, *J* = 8.5 Hz, 1H). **^{13}C NMR** (101 MHz, $CDCl_3$): δ_C = 189.0, 188.0, 160.2, 153.3, 140.3, 136.1, 131.6, 129.5, 128.8, 127.9, 125.1, 123.5, 118.7, 114.5. **FTIR** (neat) ν_{max}/cm^{-1} = 2862, 1687, 1601, 1585, 1477, 1439, 1391, 1280, 1231, 1205, 1186, 1157, 1122, 1098, 879, 825, 786, 759, 707. **HRMS** (ESI^+): *m/z* calculated for $C_{14}H_{10}{}^{79}BrO_3{}^+$, $[M+H]^+$ = 304.9808; *m/z* found = 304.9810. Δ = 0.66 ppm.

1-Bromo-3-vinyl-2-(2-vinylphenoxy)benzene, S8:

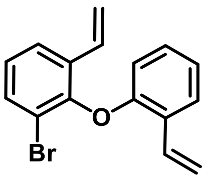

n-BuLi (1.6 M, 14.3 mL, 23.00 mmol, 4 eq.) was added to a suspension of methyltriphenylphosphonium bromide (9.37 g, 26.20 mmol, 4.5 eq.) in THF (200 mL) at –78 °C. Once the addition was complete, the reaction mixture was warmed to 0 °C for 10 mins before being cooled back to –78 °C. 3-bromo-2-(2-formylphenoxy)benzaldehyde, **S7**, (1.75 g, 5.74 mmol, 1 eq.) in THF (20 mL) was added and the reaction was allowed to warm to r.t.. After 16 h, H_2O (150 mL) was added, the mixture was extracted with Et_2O (3 × 50 mL). The organic extracts were dried ($MgSO_4$), concentrated *in vacuo*

and purified by flash column chromatography (Et_2O:pentane, 5:95, R_f = 0.6) to give the title compound as a viscous colourless oil (1.14 g, 66%). **^{1}H NMR** (500 MHz, $CDCl_3$): δ_H = 7.59 (dd, *J* = 7.8, 1.6 Hz, 2H), 7.56 (dd, *J* = 7.9, 1.5 Hz, 1H), 7.30 (dd, *J* = 17.8, 11.2 Hz, 1H), 7.12 (t, *J* = 7.9 Hz, 1H), 7.09 – 7.04 (m, 1H), 7.00 (td, *J* = 7.5, 1.2 Hz, 1H), 6.76 (dd, *J* = 17.6, 11.1 Hz, 1H), 6.29 (dd, *J* = 8.2, 1.2 Hz, 1H), 5.88 (dd, *J* = 17.7, 1.4 Hz, 1H), 5.76 (dd, *J* = 17.7, 1.0 Hz, 1H), 5.39 (dd, *J* = 11.2, 1.4 Hz, 1H), 5.25 (dd, *J* = 11.0, 1.0 Hz, 1H). **^{13}C NMR** (126 MHz, $CDCl_3$): δ_C = 154.8, 149.1, 134.0, 133.3, 131.4, 130.8, 128.9, 127.0, 126.7, 126.7, 125.8, 122.4, 118.4, 117.3, 115.4, 113.6. **FTIR** (neat) ν_{max}/cm^{-1} = 2981, 1626, 1601, 1577, 1557, 1481, 1451, 1436, 1409, 1310, 1259, 1227, 1183, 1123, 1100, 1022, 991, 914, 880, 829, 790, 773, 760, 746, 641. **HRMS** (ESI$^+$): *m/z* calculated for $C_{16}H_{14}{}^{79}BrO^+$, $[M+H]^+$ = 301.0223; *m/z* found = 301.0224. Δ = 0.33 ppm.

4-Bromodibenzo[*b*,*f*]oxepine, 13:

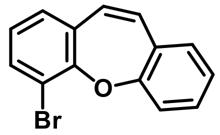

Oxepine **13** was prepared according to a literature procedure for a similar substrate.[6] Hoveyda-Grubbs 2nd Generation Catalyst (52 mg, 0.083 mmol, 2.5 mol%) in degassed PhMe (5 mL) was added to a solution of 1-bromo-3-vinyl-2-(2-vinylphenoxy)benzene, **S7**, (1.00 g, 3.33 mmol, 1 eq.) in degassed PhMe (20 mL) at 100 °C over 5 mins. After 2 h, the reaction was cooled to r.t. and diluted (60 mL, 2:98, EtOAc:Pentane). The reaction mixture was filtered through Celite® (10 mL), eluted (EtOAc:Pentane, 2:98, 3 × 20 mL) and concentrated *in vacuo*. The crude residue was purified by flash column chromatography (Et_2O:pentane, 2:98, R_f = 0.3) to give the title compound as a viscous colourless oil (768 mg, 84%). **^{1}H NMR** (400 MHz, $CDCl_3$): δ_H = 7.56 – 7.49 (m, 2H), 7.33 (ddd, *J* = 8.0, 7.0, 2.0 Hz, 1H), 7.19 (dd, *J* = 8.0, 2.0 Hz, 1H), 7.14 (td, *J* = 7.5, 1.0 Hz, 1H), 7.11 (dd, *J* = 8.0, 1.5 Hz, 1H), 6.97 (t, *J* = 8.0 Hz, 1H), 6.76 (d, *J* = 11.5 Hz, 1H), 6.69 (d, *J* = 11.5 Hz, 1H). **^{13}C NMR** (126 MHz, $CDCl_3$): δ_C = 157.2, 153.3, 133.3, 132.6, 131.1, 130.4, 130.3, 129.6, 129.4, 128.7, 125.9, 125.5, 122.6, 116.5. **FTIR** (neat) ν_{max}/cm^{-1} = 3649, 2981, 2888, 2361, 1482, 1436, 1383, 1229, 1155, 1073, 956, 747. **HRMS** (ESI$^+$): *m/z* calculated for $C_{14}H_{10}{}^{79}BrO^+$, $[M+H]^+$ = 272.9910; *m/z* found = 272.9910. Δ = 0.05 ppm.

2,4-Dimethyl-6-(4,4,5,5-tetramethyl-1,3,2-dioxaborolan-2-yl)aniline, 14:

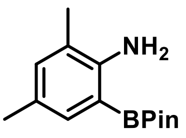

Aniline **14** was prepared according to a modified literature procedure.[3] 1,4-Dioxane (40 mL) was added to a mixture of 2-bromo-4,6-dimethylaniline (1.00 g, 5.00 mmol, 1 eq.), Pd(dppf)Cl_2·CH_2Cl_2 (122 mg, 0.15 mmol, 3 mol%), KOAc (1.47 g, 15.00 mmol, 3 eq.) and B_2Pin_2 (1.90 g, 7.50 mmol, 1.5 eq.) under N_2. Argon was bubbled through the mixture for 10 mins and the reaction was heated to 95 °C. After 20 h, H_2O (30 mL) was added. The resulting mixture was extracted (CH_2Cl_2, 3 × 15 mL), the combined organic extracts were dried ($MgSO_4$) and concentrated *in vacuo*. The crude residue was suspended in pentane (30 mL), filtered through Celite® (8 mL), eluted (pentane, 3 × 10 mL) and concentrated *in vacuo*. The crude product was used without further purification.

2-(Dibenzo[*b*,*f*]oxepin-4-yl)-4,6-dimethylaniline, S9:

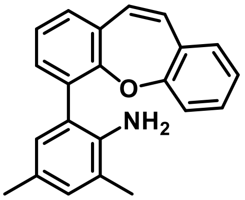

4-Bromodibenzo[*b*,*f*]oxepine, **13**, (600 mg, 2.21 mmol, 1 eq.) as a solution in PhMe (2 mL), 2,4-dimethyl-6-(4,4,5,5-tetramethyl-1,3,2-dioxaborolan-2-yl)aniline, **14**, (3.32 mmol, 1.5 eq.) as a solution in PhMe (20 mL) and water (20 mL) were sequentially added to Pd(dppf)Cl_2·CH_2Cl_2 (90.2 mg, 0.11 mmol, 5 mol%) and Na_2CO_3 (937mg, 8.84 mmol, 4 eq.) under N_2 at r.t.. Argon was bubbled through the solution for 10 mins and the reaction mixture was heated to 95 °C. After 24 h, the reaction mixture was extracted (CH_2Cl_2, 3 × 20 mL), dried ($MgSO_4$) and concentrated *in vacuo*. The crude residue was purified by flash column chromatography (Et_2O:Pentane, 10:90, R_f = 0.3) to give the title compound as a viscous oil (463 mg, 67%). **^{1}H NMR** (500 MHz, $CDCl_3$): δ_H = 7.29 (dd, *J* = 7.1, 2.2 Hz, 1H), 7.22 – 7.15 (m, 2H), 7.15 – 7.08 (m, 2H), 7.04 (td, *J* = 7.4, 1.3 Hz, 1H), 7.00 (d, *J* = 2.1 Hz, 1H, H18), 6.82 (d, *J* = 2.1 Hz, 1H), 6.79 (d, *J* = 11.4 Hz, 1H), 6.73 (d, *J* = 11.4 Hz, 1H), 6.36 (dd, *J* = 8.1, 1.3 Hz, 1H), 2.29 (s, 3H), 2.27 (s, 3H). **^{13}C NMR** (126 MHz, $CDCl_3$): δ_C = 157.0, 154.3, 140.2, 133.1, 132.0, 131.4, 130.8, 130.8, 130.3, 130.2, 129.7, 129.4, 129.0, 128.9, 126.9, 125.1, 124.9, 124.3, 122.6, 122.0, 20.6, 18.1. **FTIR** (neat) ν_{max}/cm^{-1} = 3379, 3022, 2918, 2359, 1621, 1602, 1485, 1443, 1267, 1227, 1196, 1108, 1033, 893, 863, 820, 804, 775, 732. **HRMS** (ESI$^+$): *m/z* calculated for $C_{22}H_{20}NO^+$, $[M+H]^+$ = 314.1539; *m/z* found = 314.1536. Δ = -0.96 ppm.

2-(10,11-Dihydrodibenzo[*b*,*f*]oxepin-4-yl)-4,6-dimethylaniline, 15:

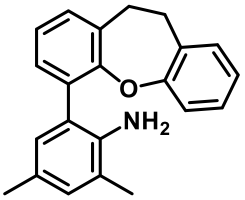

H_2 was passed through a solution of Pd/C (10%, 40 mg, 2.5 mol%) and 2-(dibenzo[*b*,*f*]oxepin-4-yl)-4,6-dimethylaniline, **S9**, (463 mg, 1.48 mmol, 1 eq.) in EtOAc (20 mL) under N_2 and kept under a positive pressure of H_2. After 16 h, N_2 was bubbled through the solution for 10 mins, the reaction was filtered (Celite®, 5 mL), eluted (EtOAc, 4 × 10 mL) and concentrated *in vacuo* to give the title compound as a viscous oil (466 mg, Quant.). **^{1}H NMR** (500 MHz, $CDCl_3$): δ_H = 7.20 (dd, *J* = 7.3, 1.9 Hz, 1H), 7.14 (dd, *J* = 7.5, 2.0 Hz, 1H), 7.10 (t, *J* = 7.5 Hz, 1H), 7.05 – 6.99 (m, 2H), 6.96 – 6.90 (m, 2H), 6.80 (d, *J* = 2.1 Hz, 1H), 6.44 (dd, *J* = 7.5, 1.9 Hz, 1H), 3.39 (ddd, *J* = 13.9, 10.5, 3.7 Hz, 1H), 3.19 – 3.10 (m, 1H), 3.03 – 2.91 (m, 2H), 2.42 (s, 3H), 2.28 (s, 3H). **^{13}C NMR** (126 MHz, $CDCl_3$): δ_C = 156.1, 154.7, 134.8, 133.4, 131.6, 131.0, 130.8, 130.6, 129.8, 129.6, 129.4, 128.1, 127.0, 126.8, 124.8, 124.5, 123.7, 121.6, 31.2, 30.4, 20.6, 18.6. **FTIR** (neat) ν_{max}/cm^{-1} = 2920, 2360, 1757, 1621, 1575, 1487, 1446, 1225, 1204, 1103, 1030, 901, 862, 802, 771, 670. **HRMS** (ESI$^+$): *m/z* calculated for $C_{22}H_{22}NO^+$, $[M+H]^+$ = 316.1696, *m/z* found = 316.1698. Δ = 0.63 ppm.

11,13-Dimethyl-4,5-dihydrobenzo[*b*]benzo[6,7]oxepino[3,2,1-*hi*]benzofuran-10-ium tetrafluoroborate, *rac*-16:

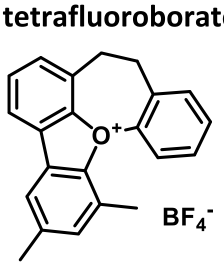

t-BuONO (0.51 mL, 442 mg, 4.28 mmol, 5 eq.) was added to a solution of 2-(10,11-dihydrodibenzo[*b*,*f*]oxepin-4-yl)-4,6-dimethylaniline, **15**, (270 mg, 0.86 mmol, 1 eq.) and HBF_4 (48% aq., 0.56 mL, 783 mg, 4.28 mmol, 5 eq.) in CH_2Cl_2:IPA (1:1, 8 mL) at 0 °C. After 1 h, the reaction mixture was diluted with CH_2Cl_2 (10 mL), washed with H_2O (2 × 5 mL) and heated to 30 °C. After 48 h, the solvent was removed by a steady stream of N_2 and Et_2O (10 mL) was added. The Et_2O layer was passed through

a Celite® filter (5 mL). The solid precipitate was washed and the solvent passed through the Celite® (Et_2O, 4 x 5 mL). The solid precipitate was dissolved in MeCN (10 mL), passed through the Celite® and eluted (MeCN, 3 × 10 mL). The solvent was removed by a steady stream of N_2 to give the title compound as a white solid (244 mg, 74%). **M.P.** = 145-147 °C ($CHCl_3/Et_2O$, dec.) **^{1}H NMR** (500 MHz, CD_3CN): δ_H = 8.01 – 7.94 (m, 2H), 7.76 (dd, *J* = 7.6, 1.8 Hz, 1H), 7.70 – 7.60 (m, 2H), 7.48 (dq, *J* = 7.8, 1.1 Hz, 1H), 7.46 – 7.44 (m, 1H), 7.39 (ddd, *J* = 9.0, 7.4, 1.8 Hz, 1H), 7.17 (dd, *J* = 8.8, 1.0 Hz, 1H), 4.10 (td, *J* = 14.1, 3.9 Hz, 1H), 3.56 – 3.47 (m, 1H), 3.27 (dt, *J* = 14.2, 3.7 Hz, 1H), 3.21 – 3.09 (m, 1H), 2.56 (s, 3H), 2.17 (s, 3H). **^{13}C NMR** (126 MHz, CD_3CN): δ_C = 162.2, 161.0, 153.7, 143.3, 134.7, 133.6, 133.5, 132.9, 132.6, 131.7, 130.5, 127.3, 126.1, 125.2, 123.9, 122.9, 122.3, 115.6, 30.1, 27.9, 21.3, 16.8. **^{19}F NMR** (471 MHz, CD_3CN): δ_F = -151.8. **FTIR** (neat) ν_{max}/cm^{-1} = 1481, 1455, 1407, 1051, 900, 828, 799, 773, 734, 613. **HRMS** (ESI^+): *m/z* calculated for $C_{22}H_{19}O^+$, $[M]^+$ = 299.1430 *m/z* found = 299.1432. Δ = -1.34 ppm.

11,13-Dimethyl-4,5-dihydrobenzo[*b*]benzo[6,7]oxepino[3,2,1-*hi*]benzofuran-10-ium hexafluorophosphate, *rac*-17:

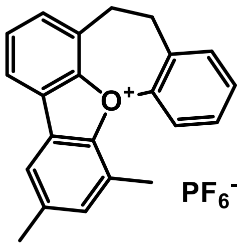

11,13-Dimethyl-4,5-dihydrobenzo[*b*]benzo[6,7]oxepino[3,2,1-*hi*]benzofuran-10-ium, tetrafluoroborate, ***rac*-16**, (15 mg, 0.04 mmol, 1 eq.) and potassium hexafluorophosphate (35 mg, 0.19 mmol, 5 eq.) were dissolved in MeCN (1 mL). After 1 min, the solvent was removed by a steady stream of N_2. CH_2Cl_2 (1 mL) was added and the mixture was washed with H_2O (2 × 1 mL). The organic layers were dried ($MgSO_4$) and the solvent was removed by a steady stream of N_2. Et_2O (4 mL) was added and the solvent was removed by a steady stream of N_2. **M.P.** = 149-151 °C ($CHCl_3/Et_2O$, dec.) **^{1}H NMR** (600 MHz, CD_3CN): δ_H = 8.00 – 7.94 (m, 2H), 7.76 (dd, *J* = 7.5, 2.0 Hz, 1H), 7.68 – 7.61 (m, 2H), 7.50 – 7.45 (m, 2H), 7.39 (ddd, *J* = 9.0, 7.5, 2.0 Hz, 1H), 7.19 – 7.15 (m, 1H), 4.10 (td, *J* = 14.0, 4.0 Hz, 1H), 3.51 (dt, *J* = 18.0, 3.5 Hz, 1H), 3.27 (dt, *J* = 14.0, 3.5 Hz, 1H), 3.21 – 3.09 (m, 1H), 2.57 (s, 3H), 2.17 (s, 3H). **^{13}C NMR** (151 MHz, CD_3CN): δ_c 162.3, 161.0, 153.7, 143.4, 134.8, 133.6, 133.5, 132.9, 132.7, 131.7, 130.5, 127.3, 126.1, 125.2, 123.9, 122.9, 122.3, 115.6, 30.1, 27.9, 21.3, 16.8. **^{19}F NMR** (376 MHz, CD_3CN): δ_F = -71.7 – -72.1 (m), -73.6 – -74.0 (m). **FTIR** (neat) ν_{max}/cm^{-1} = 3057, 2917, 2849, 2360, 2342, 2263, 1423, 1267, 1189, 1017, 896, 846, 792, 736, 704, 668. **HRMS** (ESI^+): *m/z* calculated for $C_{22}H_{19}O^+$, $[M]^+$ = 299.1430 *m/z* found = 299.1427. Δ = -1.15 ppm.

***(M*, (*S)*$_O$*)*-11,13-dimethyl-4,5-dihydrobenzo[b]benzo[6,7]oxepino[3,2,1-hi]benzofuran-10-ium hexafluorophosphate, enantioenriched-17 (97:3):**

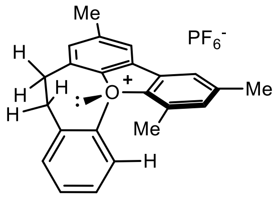

$[\alpha]_D^{25}$ = -2.82 (*c* = 1.0, $CHCl_3$)

Purified by semi-preparative chiral HPLC (2x Chiralpak IC, 90% 250 mM $NaPF_6$ in MeOH, 10% H_2O, 1.0 mL min^{-1}, λ = 270 nm τ_R (major) = 22.2 min, τ_R (minor) = 23.6 min).

Sodium bis-(*R*)-BINOL borate, S10:

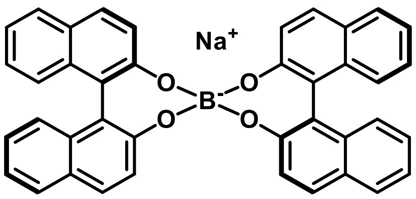

$NaBH_4$ (500 mg, 13.21 mmol, 1 eq.) was slowly added to MeOH (25 mL) at r.t. over 10 mins. After 30 mins the reaction was heated to reflux. After 1 h, the solvent was removed *in vacuo* and the white solid was dried at 80 °C for 16 h to give $NaB(OMe)_4$ as a white solid (2.09 g, Quant.) that was used without further purification. Sodium bis-(*R*)-BINOL borate, **S10**, was prepared according to a modified literature procedure for racemic sodium bis-BINOL borate.[7] (*R*)-BINOL (1.00 g, 3.50 mmol, 2.94 eq.) and $NaB(OMe)_4$ (2.84 g, 1.80 mmol, 1 eq.) were dissolved in THF (30 mL) and heated to reflux. After 20 h, the reaction was cooled to 40 °C and $B(OMe)_3$ (0.25 mL, 235 mg, 2.27 mmol, 1.26 eq.) was added. After 1 h, the reaction was heated to reflux. After 3 h, the reaction was cooled to r.t. and left for 16 h to crystallise. The precipitate was collected by suction filtration and sequentially washed with THF (2 × 20 mL), Et_2O (2 × 20 mL) and pentane (2 × 20 mL). The crude product consisted of sodium bis-(*R*)-BINOL borate·xTHF adduct where x = 2.5. The crude product was dried at 80 °C under vacuum for 7 d to give the title compound as a white solid (501 mg, 46%). **M.P.** = >250 °C. **^{1}H NMR** (400 MHz, DMSO-d$_6$): δ_H = 7.98 (d, *J* = 8.8 Hz, 4H), 7.94 (d, *J* = 8.2 Hz, 4H), 7.36 (d, *J* = 8.7 Hz, 4H), 7.30 (ddd, *J* = 8.0, 5.7, 2.2 Hz, 4H), 7.22 – 7.12 (m, 8H). **^{13}C NMR** (101 MHz, DMSO-d$_6$): δ_C = 156.1, 132.8, 128.9, 128.2, 128.0, 125.8, 124.8, 124.6, 122.4, 121.8. **FTIR** (film) ν_{max}/cm^{-1} = 3053, 2162, 1618, 1590, 1505, 1464, 1428, 1366, 1331, 1266, 1246, 1155, 1071, 986, 948, 910, 828, 812, 744, 720, 665, 633. **LRMS** (ESI$^-$): *m/z* calculated for $C_{20}H_{13}O_2^-$, $[M-C_{20}H_{11}BO_2]^-$ = 285; *m/z* found = 285. $[\alpha]_D^{25}$ = -284 (c = 1.04, DMSO). *Spectra are consistent with literature values.[7]

3 Experimental data for resolution of *rac*-16

11,13-Dimethyl-4,5-dihydrobenzo[*b*]benzo[6,7]oxepino[3,2,1-*hi*]benzofuran-10-ium, bis-(*R*)-BINOL borate, 18 and 19:

11,13-Dimethyl-4,5-dihydrobenzo[*b*]benzo[6,7]oxepino[3,2,1-*hi*]benzofuran-10-ium, tetrafluoroborate, ***rac*-16**, (50 mg, 0.13 mmol, 1 eq.) and sodium [bis-(R)-BINOL borate] (78 mg, 0.13 mmol, 1 eq.) were dissolved in MeCN (8 mL). After 1 min, the solvent was removed by a steady stream of N_2. CH_2Cl_2 (8 mL) was added and the mixture was washed with H_2O (2 × 4 mL). The organic layers were dried ($MgSO_4$) and the solvent was removed by a steady stream of N_2. Et_2O (4 mL) was added and the solvent was removed by a steady stream of N_2. Data for the 1:1 mixture of diastereoisomeric oxonium ion/ bis-(*R*)-BINOL borate ion pairs **18** and **19** was obtained at this point.

Data for 1:1 mixture of diastereoisomers*

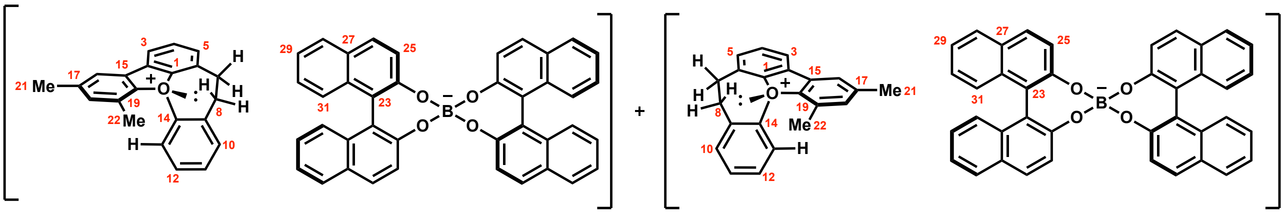

18 ***P*-(*R*)$_O$, (*R*)** **19** ***M*-(*S*)$_O$, (*R*)**

* This compound was found to display particularly concentration-dependant ^{1}H NMR chemical shifts.

M.P. = 180 °C ($CHCl_3/Et_2O$, dec.) **^{1}H NMR** (400 MHz, CD_2Cl_2): δ_H = 7.84 (td, *J* = 7.5, 1.0 Hz, 16H, H_{28}, H_{26}), 7.77 (dd, *J* = 9.0, 1.0 Hz, 1H, H_{Ar}), 7.55 (dt, *J* = 2.0, 1.0 Hz, 1H, H_{16}), 7.48 (ddd, *J* = 7.5, 1.5, 1.0 Hz, 2H, H_{Ar}), 7.42 – 7.33 (m, 9H, H_{30}, H_{Ar}), 7.32 – 7.08 (m, 21H, H_{31}, H_{29}, H_{Ar}), 7.08 – 6.98 (m, 10H, H_{25}, H_{Ar}), 6.98 – 6.96 (m, 1H, H_{Ar}), 6.95 – 6.89 (m, 1H, H_{Ar}), 6.76 (dq, *J* = 7.5, 1.0 Hz, 1H, H_{Ar}), 6.66 – 6.55 (m, 3H, H_{18}, H_{13}, H_3), 3.72 (ddd, *J* = 14.0, 12.0, 6.0 Hz, 1H, H_{8A}), 3.48 (td, *J* = 15.5, 4.0 Hz, 1H, $H_{8A'}$), 3.07 – 2.98 (m, 1H, $H_{8B'}$), 2.74 – 2.52 (m, 3H, H_{8B}, $H_{7B'}$, $H_{7A'}$), 2.49 – 2.40 (m, 2H, H_{7B}, H_{7A}), 2.38 (d, *J* = 0.5 Hz, 3H, H_{22}), 2.31 (d, *J* = 1.0 Hz, 3H, $H_{22'}$), 1.78 (d, *J* = 1.0 Hz, 3H, $H_{21'}$), 1.61 (s, 3H, H_{21}). **^{13}C NMR** (151 MHz, CD_2Cl_2): δ_C = 161.0 (C_{Ar}), 160.7 (C_{Ar}), 160.2 (C_{Ar}), 160.1 (C_{Ar}), 156.8 (C_{24}), 152.6 (C_{20}), 152.4 (C_{20}), 142.3 (C_{Ar}), 142.0 (C_{Ar}), 134.0 (C_{Ar}), 133.6 (C_{Ar}), 133.6 (C_{32}), 132.7 (C_{Ar}), 132.6 (C_{Ar}), 132.4 (C_{Ar}), 132.3 (C_{Ar}), 132.1 (C_{Ar}), 131.9 (C_{Ar}), 131.8 (C_{Ar}), 131.7 (C_{Ar}), 130.6 (C_{Ar}), 130.1 (C_{Ar}), 129.8 (C_{27}), 129.2 (C_{Ar}), 129.1(C_{Ar}), 128.5 ($C_{28/26}$), 128.2 ($C_{28/26}$), 127.2 (C_{Ar}), 127.0 (C_{31}), 125.6 (C_{Ar}), 125.6 (C_{Ar}), 125.3 (C_{30}), 124.8 (C_{25}), 124.6 (C_{Ar}), 124.6 (C_{Ar}), 124.3 (C_{Ar}), 124.3 (C_{Ar}), 123.8 (C_{Ar}), 122.8 (C_{29}), 122.6 (C_{23}), 122.1 (C_{Ar}), 121.6 (C_{Ar}), 121.4(C_{Ar}), 121.1 (C_{Ar}), 113.9 (C_{13}), 113.8 (C_{13}), 29.3 (C_7), 29.1 (C_7), 27.3 (C_8), 27.2 (C_8), 21.3 (C_{21}), 21.3 (C_{21}), 16.5 (C_{22}). **FTIR** (film) ν_{max}/cm^{-1} = 3505, 3058, 2360, 2341, 1619, 1593, 1506, 1464, 1432, 1383, 1333, 1272, 1247, 1212, 1171, 1145, 1127, 1070, 1006, 953, 909, 864, 816, 791, 748, 712, 678, 666, 627. **HRMS** (ESI$^+$): *m/z* calculated for $C_{22}H_{19}O$, $[M]^+$ = 299.1431, *m/z* found = 299.1430. Δ = -0.12 ppm.^{19}F NMR (377 MHz, CD_2Cl_2): δ_F = No peaks were observed, indicating the loss of the BF_4 counterion.

The mixture was dissolved in $CHCl_3$ (3 mL) and concentrated to approximately 1 mL by a steady stream of N_2. After standing for 1 h at r.t, the mixture was filtered through Celite® (0.2 mL) and the solid precipitate was washed with $CHCl_3$ (6 × 0.2 mL). The solid precipitate was eluted from the Celite® with CH_2Cl_2 (4 × 0.5 mL) and the solvent was removed by a steady stream of nitrogen. The solid was dried under vacuum for 2 h to give the title compound as a white solid. Maximum theoretical yield of each diastereoisomer = 56.8 mg. Isolated yield of *P*, $(R)_O$, [B((*R*)-BINOL)$_2$] diastereoisomer **18** = 25.8 mg, 96:4 d.r. (46% of theoretical).

Data for major diastereoisomer from resolution, 18 *P*-(*R*)$_O$, (*R*):

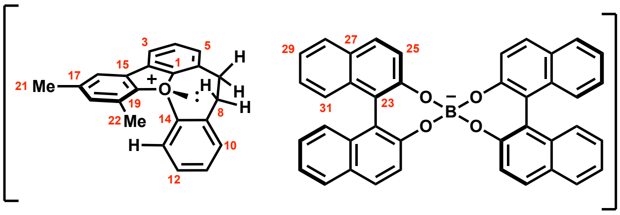

18 *P*-(*R*)$_O$, (*R*)

M.P. = 120-124 °C ($CHCl_3/Et_2O$, dec.) **^{1}H NMR** (400 MHz, CD_2Cl_2) δ 7.87 – 7.81 (m, 8H, H_{28}, H_{26}), 7.55 – 7.50 (m, 1H, H_{16}), 7.45 (dd, *J* = 7.5, 1.5 Hz, 1H, H_3), 7.37 – 7.30 (m, 5H, H_{10}, H_{25}), 7.27 – 7.16 (m, 9H, H_{31}, H_{29}, H_{Ar}), 7.13 – 7.03 (m, 5H, H_{30}, H_{Ar}), 6.98 (ddd, *J* = 9.0, 7.5, 2.0 Hz, 1H, H_4), 6.72 (dd, *J* = 8.0, 1.5 Hz, 1H, H_5), 6.55 (dd, *J* = 5.0, 3.5 Hz, 2H, H_{18}, H_{13}), 3.68 (ddd, *J* = 14.0, 11.5, 6.5 Hz, 1H, H_{8A}), 2.63 (dt, *J* = 14.0, 3.5 Hz, 1H, H_{8B}), 2.41 – 2.38 (m, 2H, H_7), 2.36 (s, 3H, H_{21}), 1.57 (s, 3H, H_{22}). **^{13}C NMR** (101 MHz, CD_2Cl_2): δ_C = 160.7 (C_{14}), 160.2 (C_1), 156.9 (C_{Ar}), 152.6 (C_{20}), 142.0 (C_{Ar}), 133.7 (C_{Ar}), 133.6 (C_{18}), 132.6 (C_{Ar}), 132.4 (C_{Ar}), 132.1 (C_{Ar}), 131.8 (C_{Ar}), 130.1 (C_{Ar}), 129.8 (C_{Ar}), 129.0 (C_4), 128.4 (C_{Ar}), 128.3 (C_{Ar}), 127.1 (C_{Ar}), 125.6 (C_{Ar}), 125.3 (C_{25}), 124.9 (C_{Ar}), 124.8 (C_{Ar}), 124.3 (C_{Ar}), 122.7 (C_{Ar}), 122.6 (C_{Ar}), 121.6 (C_{16}), 121.1 (C_3), 113.9 (C_{13}), 78.0 (C_{Ar}), 29.1 (C_7), 27.3 (C_8), 21.4 (C_{21}), 16.5 (C_{22}). **FTIR** (film) ν_{max}/cm^{-1} = 3056, 2918, 2360, 2341, 1617, 1591, 1505, 1481, 1464, 1429, 1366, 1335, 1272, 1249, 1211, 1142, 1127, 1070, 988, 947, 928, 910, 849, 816, 791, 747, 711, 677, 668, 649, 634, 611. **HRMS** (ESI$^+$): *m/z* calculated for $C_{22}H_{19}O$, $[M]^+$ = 299.1431, *m/z* found = 299.1430. Δ = -0.22 ppm.

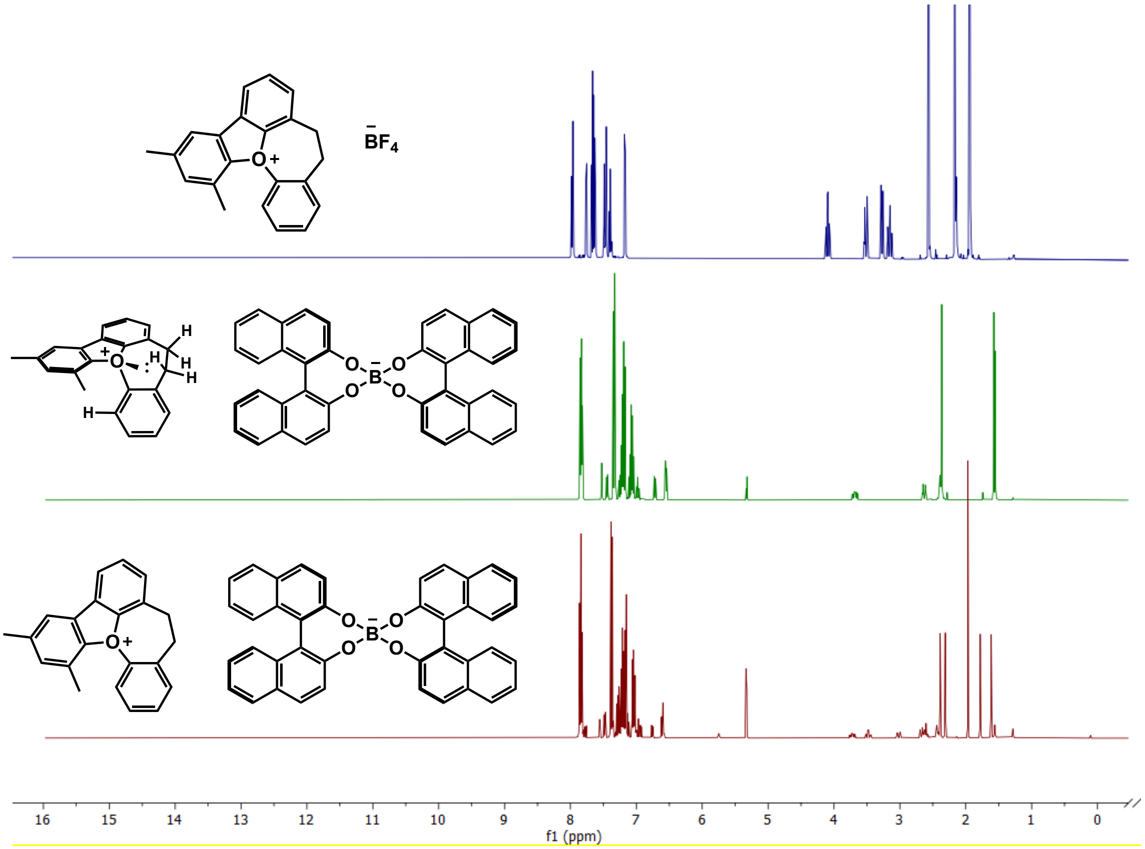

Figure S3: ^{1}H NMR spectra of *rac*-**16** (top), diastereoisomerically enriched **18** (d.r. 94:6, middle) and 1:1 mixture of diastereoisomers **18** & **19** (bottom).

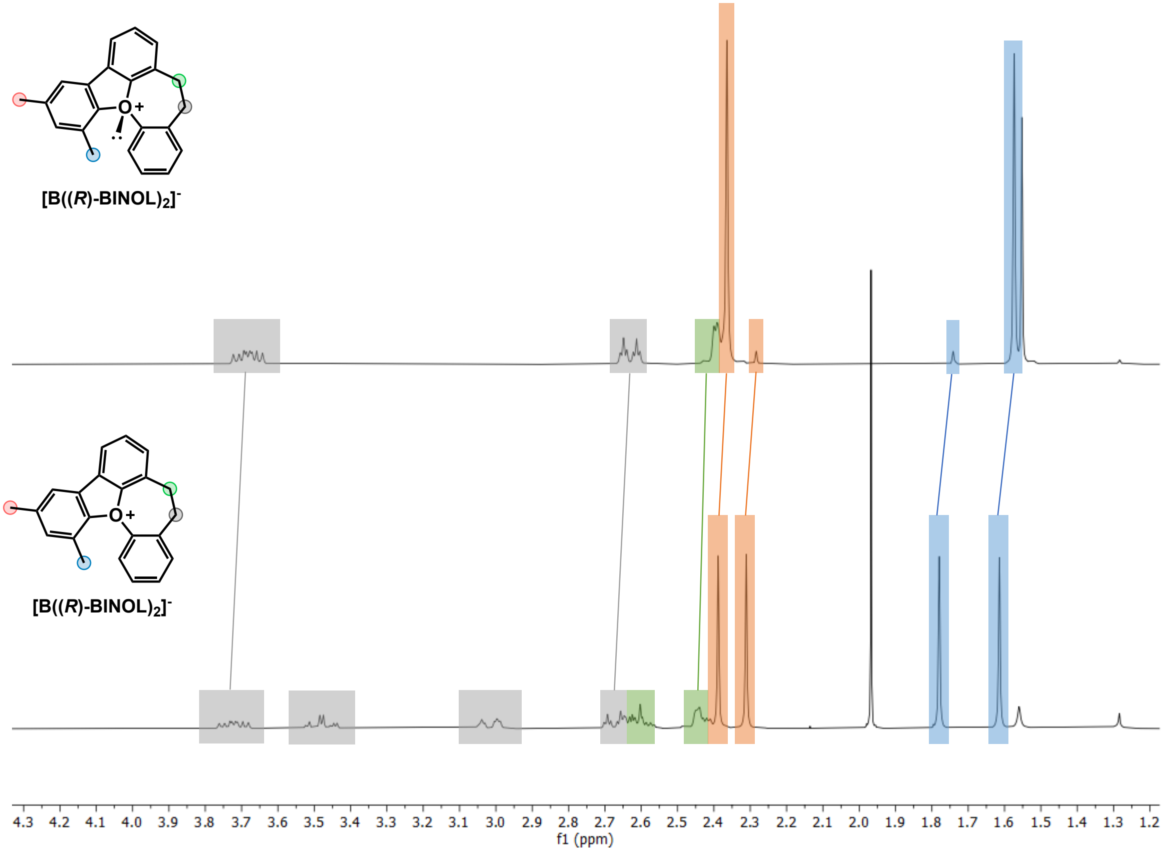

Figure S4: Expansion of ^{1}H NMR spectra of diastereoisomerically enriched **18** (d.r. 94:6, top) and 1:1 mixture of diastereoisomers **18** & **19** (bottom).

The resolution process in pictures (read in context of experimental procedure above):

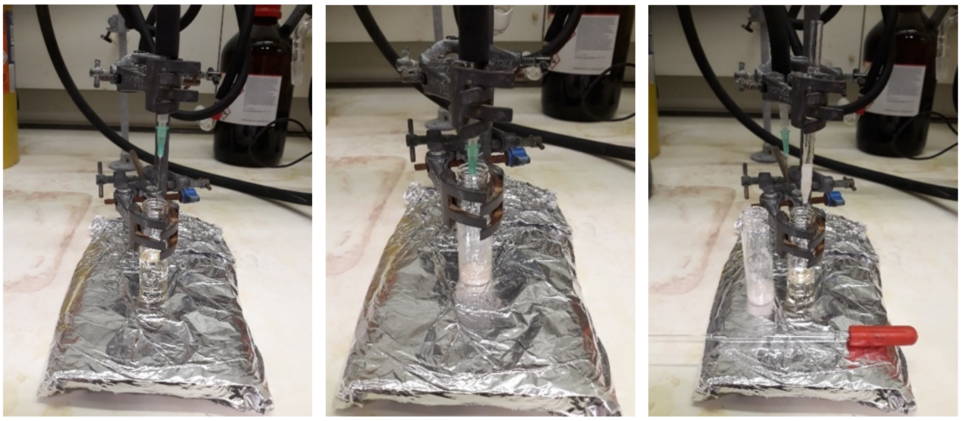

Figure S5: ***Left***: Sodium bis-(*R*)-BINOL borate, **S10**, is poorly soluble in CH_2Cl_2 so the formation of the salt mixture must be carried out in MeCN. ***Centre***: The MeCN is removed by a steady stream of N_2. ***Right***: Addition of CH_2Cl_2 leads to precipitation of some $NaBF_4$, though multiple water washes are necessary to remove it entirely (this can be validated by ^{19}F NMR analysis). The CH_2Cl_2 layers are dried over $MgSO_4$ and filtered using a pipette filter.

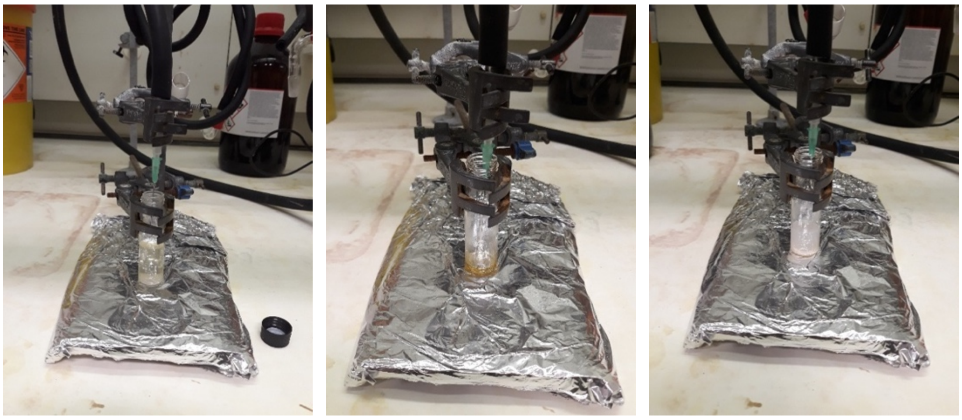

Figure S6. ***Left***: The CH_2Cl_2 is removed by a steady stream of N_2. ***Centre***: After concentration of the oxonium-borate solution, extended periods under vacuum may be required to remove the CH_2Cl_2, which can interfere in the resolution if not removed fully. This can be enabled by the sequential addition and then removal of Et_2O. This provides a convenient solution to the removal of CH_2Cl_2, and yields a white powder (***Right***).

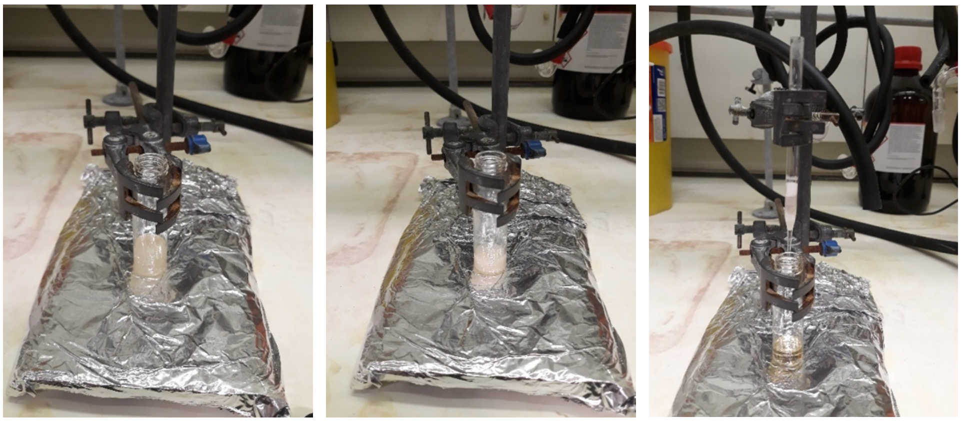

Figure S7. ***Left***: Some precipitation is observed after the addition of $CHCl_3$ (3 mL). ***Centre***: Upon concentration to 1 mL, the less soluble diastereomeric ion-pair precipitates from the mother liquor. ***Right***: Celite® filtration is used to isolate the diastereoenriched oxonium ion. Washing the solid obtained with $CHCl_3$ reliably gives the oxonium ion in high diastereoselectivity. It is then eluted from the Celite® using CH_2Cl_2.

4 X-ray Crystallography

Single crystal X-ray diffraction was carried out by Owen A. Smith using a (Rigaku) Oxford Diffraction/Agilent Supernova A diffractometer (Cu-Ka radiation, λ = 1.54184 Å) within the Department of Chemistry, University of Oxford. Samples were mounted in perfluoropoly-ethyl ether oil and flash-cooled to 150 K by a N_2 Crysostream open-flow cooling device.[8] The raw frame data was integrated and reduced using CrysalisPro. SuperFlip[9] embedded within CRYSTALS[10,11] was used to obtain an ab initio solution using CRYSTALS and for structure refinement.

Molecular graphics of the structure solutions are shown with displacement ellipsoid plots of the best fit model drawn at 50% probability. Raw frame data, unprocessed cif files and completed cif files for all structures are available on request.

4.1 1,3,6-Trimethyl-8*H*-benzofuro[3,2,1-*de*]xanthen-13-ium tetrafluoroborate, **10**:

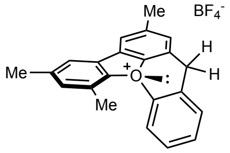

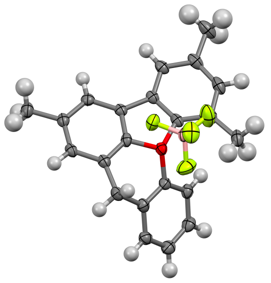

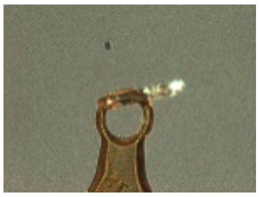

Vapour diffusion of Et_2O into a solution of **10** (5 mg) in CH_2Cl_2 (0.6 mL) over 16 h gave colourless needles suitable for single crystal x-ray analysis.

**Identification Code**	001os20 (CCDC 2124020)	
**Empirical Formula**	C22 H19 B F4 O	
**M_r**	386.20	
**Temperature**	150 K	
**Wavelength**	λ = 1.54180 Å (Cu K_α)	
**Crystal System**	Monoclinic	
**Space Group**	P 2_1/c	
**Unit Cell Dimensions**	a = 8.0432(3) Å	α = 90°
	B = 12.6195(5) Å	β = 96.911(3)°
	c = 18.0018(7) Å	γ = 90°
**Volume**	1813.93(12) ($Å^3$)	
**Z**	4	
**Density (Calculated)**	1.414 Mg m^{-3}	
**Absorption Coefficient**	0.954 mm^{-1}	
**Crystal Size**	0.05 x 0.06 x 0.35 mm^3	
**Theta range for data collection**	4.289 to 75.949°	
**Index Ranges**	-10<=h<=9 , -14<=k<=15 , -15<=l<=22	
**Reflections Collected**	11627	
**Independent Reflections**	3735 [R(int) = 0.046]	
**Completeness to theta = 73.671°**	99.6%	
**Absorption Correction**	multi-scan	
**Refinement method**	Full-matrix least-squares on F^2	
**Goodness-of-fit on F^2**	1.0103	
**Final R indices [I>2sigma(I)]**	R1 = 0.0692, wR2 = 0.1373	
**R indices (all data)**	R1 = 0.0506, wR2 = 0.1213	

4.2 11,13-Dimethyl-4,5-dihydrobenzo[*b*]benzo[6,7]oxepino[3,2,1-*hi*] benzofuran-10-ium tetrafluoroborate, ***rac*-16**:

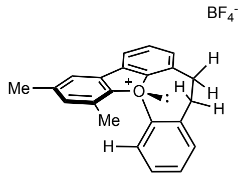

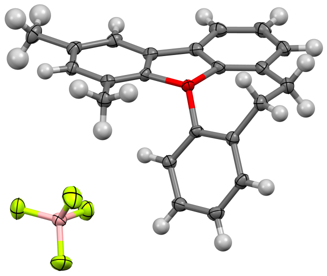

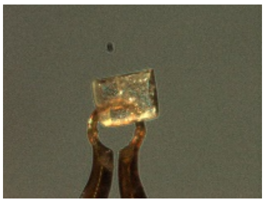

Vapour diffusion of Et_2O into a solution of *rac*-**16** (5 mg) in CH_2Cl_2 (0.6 mL) over 16 h gave colourless blocks suitable for single crystal x-ray analysis.

**Identification Code**	027os21 (CCDC 2124019)	
**Empirical Formula**	C22 H19 B F4 O	
M_r	386.20	
**Temperature**	150 K	
**Wavelength**	λ = 1.54184 Å (Cu K_α)	
**Crystal System**	Triclinic	
**Space Group**	P –1	
**Unit Cell Dimensions**	a = 8.2151(2) Å	α = 75.869(2)°
	b = 9.8320(2) Å	β = 76.543(2)°
	c = 11.9880(3 Å	γ = 88.198(2)°
**Volume**	912.87(4) ($Å^3$)	
**Z**	2	
**Density (Calculated)**	1.405 Mg m^{-3}	
**Absorption Coefficient**	0.948 mm^{-1}	
**Crystal Size**	0.10 x 0.18 x 0.21 mm^3	
**Theta range for data collection**	3.909 to 76.160°	
**Index Ranges**	-10<=h<=8 , -12<=k<=12, -15<=l<=14	
**Reflections Collected**	19078	
**Independent Reflections**	3781 [R(int) = 0.021]	
**Completeness to theta = 73.875°**	99.6%	
**Absorption Correction**	multi-scan	
**Refinement method**	Full-matrix least-squares on F^2	
**Goodness-of-fit on F^2**	1.0033	
**Final R indices [I>2sigma(I)]**	R1 = 0.0361, wR2 = 0.0900	
**R indices (all data)**	R1 = 0.0346, wR2 = 0.0855	

4.3 11,13-Dimethyl-4,5-dihydrobenzo[*b*]benzo[6,7]oxepino[3,2,1-*hi*] benzofuran-10-ium tetrafluoroborate, *enantioenriched*-**16**:

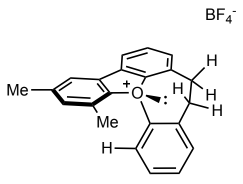

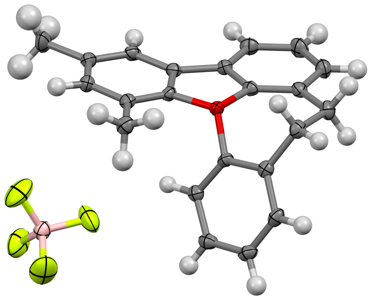

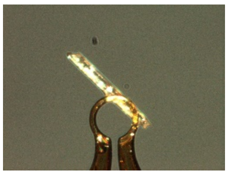

Oxonium ion bis-(*R*)-BINOL borate salt, **18**, 94:6 d.r. (3 mg) was dissolved in CH_2Cl_2 (0.5 mL). The organic layer was washed with 48% aq. HBF_4 (3 × 0.5 mL), dried ($MgSO_4$) and the solvent was removed by a steady stream of N_2. Vapour diffusion of Et_2O into a solution of the crude residue in CH_2Cl_2 (0.6 mL) over 16 h gave a mixture of an amorphous solid and colourless needles. The colourless needles were subjected to single crystal x-ray diffraction analysis. The Flack x parameter[12,13] was refined to -0.04(10), with a Parsons' q of -0.03(3).[14] Bayesian analysis of the Bijvoet pairs was used to give the Hooft y parameter[15] as -0.02(4). Two further crystals were measured and the same absolute structure was found in each case (Flack x parameters: 0.08(12) and -0.06(12), Parsons' q parameters: 0.03(4) and -0.03(3), Hooft y parameters: 0.04(5) and -0.05(4) respectively) allowing us to assign the absolute structure as (*P*, R_O).

**Identification Codes**	037os21, 035os21, 038os21 (CCDC 21911038-2191040)	
**Empirical Formula**	C22 H19 B F4 O	
$\boldsymbol{M_r}$	386.19	
**Temperature**	150 K	
**Wavelength**	λ = 1.54180 Å (Cu K_α)	
**Crystal System**	Orthorhombic	
**Space Group**	P 21 21 21	
**Unit Cell Dimensions**	a = 6.9568(1) Å	α = 90°
	B = 14.0648(1) Å	β = 90°
	c = 18.5135(1) Å	γ = 90°
**Volume**	1811.47(3) ($Å^3$)	
**Z**	4	
**Density (Calculated)**	1.416 Mg m^{-3}	
**Absorption Coefficient**	0.956 mm^{-1}	
**Crystal Size**	0.05 x 0.06 x 0.410 mm^3	
**Theta range for data collection**	3.947 to 76.072°	
**Index Ranges**	-8<=h<=8, -17<=k<=17, -23<=l<=22	
**Reflections Collected**	41603	
**Independent Reflections**	3765 [R(int) = 0.044]	
**Completeness to theta = 74.550°**	99.8%	
**Absorption Correction**	multi-scan	
**Refinement method**	Full-matrix least-squares on F^2	
**Goodness-of-fit on F^2**	0.9966	
**Final R indices [I>2sigma(I)]**	R1 = 0.0297, wR2 = 0.0788	
**R indices (all data)**	R1 = 0.0308, wR2 = 0.0802	
**Flack x parameter**	-0.04(10)	
**Parsons' q parameter**	0.03(4)	
**Hooft y parameter**	-0.02(4)	

5 Inversion Barrier Measurements

5.1 1,3,6-Trimethyl-8*H*-benzofuro[3,2,1-*de*]xanthen-13-ium tetrafluoroborate, **10**:

The barrier to enantiomerization of **10** was estimated using a Bruker AVIIIHD 500 MHz (^{1}H) spectrometer equipped with a BCU-II temperature regulation unit. Calculations were performed and graphs generated in Microsoft Excel.

Using the method presented by Clayden[16], the enantiomerization constant, k_{ent}, for a diastereotopic AB system can be approximated using equation (1) and substitution of k_{ent} into the Eyring equation (2) gives the enantiomerization barrier, $\Delta G^{\ddagger}_{ent}$, at the coalescence temperature, T_c.

$$(1)\ k_{ent} = \pi\sqrt{\frac{(\Delta v)^2 + 6(J_{AB})^2}{2}}$$

$$J_{AB} = (v_1 - v_2) = (v_3 - v_4)\ and\ \Delta v = \sqrt{(v_1 - v_4)(v_2 - v_3)}$$

$$(2)^{\dagger}\ \Delta G^{\ddagger}_{ent}(T_c) = \kappa R T_c ln\left(\frac{k_B T_c}{h k_{ent}}\right)$$

$$R = 8.314\, JK^{-1}mol^{-1}, k_B = 1.3806 \times 10^{-23}\, JK^{-1}\ and\ h = 6.626 \times\ 10^{-34}\, Js$$

Oxonium ion **10** (10 mg) in CD_2Cl_2 (0.5 mL) was cooled to –40 °C (233 K), the sample was allowed to equilibrate for 20 mins and a ^{1}H NMR spectrum was recorded from which values for J_{AB} and Δv were obtained. The sample was subsequently warmed in 10 °C intervals from –40 °C (233 K) to 20 °C (293 K) and then in 1 °C intervals from 20 °C (293 K) to 22 °C (295 K) to obtain the coalescence temperature, T_c. In each case, the sample was equilibrated at the desired temperature for 20 minutes and a ^{1}H NMR spectrum was recorded.

The following values were obtained: $\Delta v = 124.1\, Hz,\ J_{AB} = 17.6\, Hz\ and\ T_c = 295\, K.$

$$(1)\ k_{ent} = \pi\sqrt{\frac{(\Delta v)^2 + 6(J_{AB})^2}{2}} = 2918\, s^{-1}$$

$$(2)\ \Delta G^{\ddagger}_{ent}(T_c) = R T_c ln\left(\frac{k_B T_c}{h k_{ent}}\right)$$

$$\Delta G^{\ddagger}_{ent}(295) = 58302\, Jmol^{-1} = 58.3\, kJmol^{-1} = 13.9\, kcalmol^{-1}$$

† In this equation the transmission coefficient κ = 1 in the absence of other information.

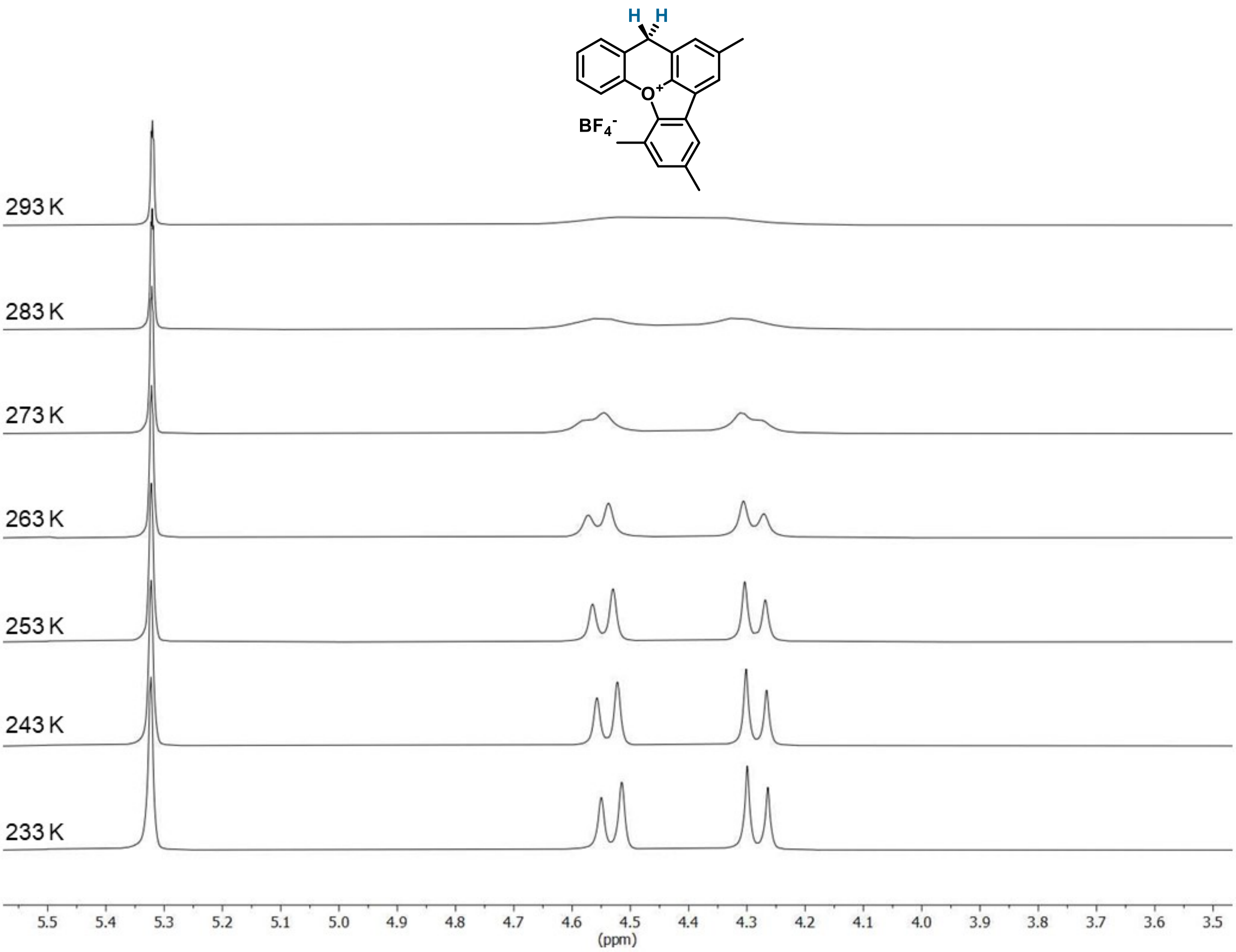

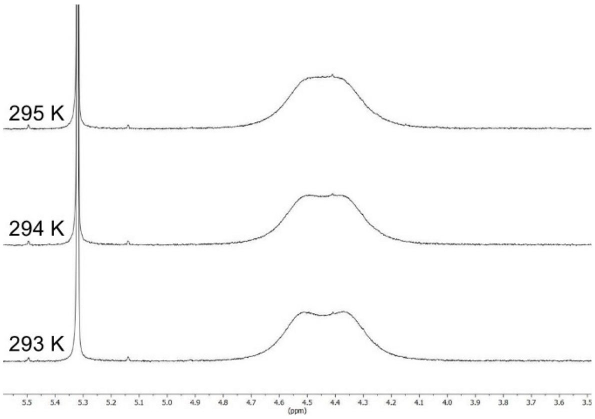

v_1 = 4.5499 ppm

v_2 = 4.5148 ppm

v_3 = 4.2994 ppm

v_4 = 4.2641 ppm

Δv = 0.2481 ppm = 124.058 Hz (500 MHz)

Average J_{AB} = 0.0352 ppm = 17.6 Hz (500 MHz)

Figure S8: Variable temperature ^{1}H NMR spectra of **10** (233 to 295 K) and values for T_c, Δv and J_{AB}.

5.2 Equilibration of 11,13-Dimethyl-4,5-dihydrobenzo[*b*]benzo[6,7]oxepino[3,2,1-*hi*]benzofuran-10-ium, [*P*, $(R)_O$]-bis-(*R*)-BINOL borate, **18**:

The barrier to diastereomerization of **18** is quoted as an average of three experiments:

Experiment 1:
Oxonium ion **18** (8.8 mg, 0.01 mmol) at 94:6 d.r. was dissolved in CD_2Cl_2 (0.45 mL) in an NMR tube fitted with a J. Young valve. CH_2Br_2 (0.005 mmol) in CD_2Cl_2 (0.05 mL) was added as an internal standard and the diastereomeric ratio was monitored by ^{1}H NMR using a Bruker AVII 500 MHz spectrometer. The sample was maintained at 298 K for the duration of the experiment (using a Cambridge Reactor Design Polar Bear Plus). ^{1}H NMR spectra with extended relaxation times were recorded at 24 h intervals

Experiment 2:
Oxonium ion **18** (8.8 mg, 0.01 mmol) at 97:3 d.r. was dissolved in CD_2Cl_2 (0.5 mL) in an NMR tube fitted with a J. Young valve under Argon. 1,2,4,5-Tetramethylbenzene (0.005 mmol) in CD_2Cl_2 (0.01 mL) was added as an internal standard and the diastereomeric ratio was monitored by ^{1}H NMR using a Bruker AVII 400 MHz spectrometer. The sample was maintained at 298 K for the duration of the experiment (using a Cambridge Reactor Design Polar Bear Plus).

Experiment 3:
Oxonium ion **18** (8.8 mg, 0.01 mmol) at 93:7 d.r. was dissolved in CD_2Cl_2 (0.5 mL) in an NMR tube fitted with a J. Young valve under Argon. 1,2,4,5-Tetramethylbenzene (0.005 mmol) in CD_2Cl_2 (0.01 mL) was added as an internal standard and the diastereomeric ratio was monitored by ^{1}H NMR using a Bruker AVII 500 MHz spectrometer. The sample was maintained at 298 K for the duration of the experiment (using a Cambridge Reactor Design Polar Bear Plus).

To prepare the internal standard stock solution **Experiment 1**:
(1) CH_2Br_2 (10.5 µl, 26.1 mg, 0.15 mmol) was dissolved in CD_2Cl_2 (0.3 mL).
(2) 0.1 mL of (1) (0.05 mmol CH_2Br_2) was diluted with CD_2Cl_2 (0.4 mL).

To prepare the internal standard stock solution **Experiment 2 and 3**:
(1) 1,2,4,5-tetramethylbenzene (67.1 mg, 0.5 mmol) was dissolved in CD_2Cl_2 (1 mL).
Integration of the signals at 3.70 and 3.44 ppm, belonging to the H_8 residue in the major and minor diastereoisomeric salt pair respectively, relative to the internal standard were used to calculate the ratio.

The sum of the integrals of the resonances at 3.70 and 3.44 ppm were monitored over the course of the experiment and no change was observed.

A sample of **18** was left to equilibrate for 5 weeks and the ratio of diastereoisomers was found to be 1.02 by integration of the NMR peaks at 3.70 and 3.44. Using the method presented by Schurig,[17] the diastereomerization constants, $k_{A\text{-}to\text{-}B}$ and $k_{B\text{-}to\text{-}A}$, for a diastereotopic AB system can be calculated using the equilibrium constant, K_{eq}, and the rate constant for equilibration, $k_{A\text{-}eq}$, using equation (4). Substitution of $k_{A\text{-}to\text{-}B}$ and $k_{B\text{-}to\text{-}A}$ into the Eyring equation (5) gives the diastereomerization barriers, $\Delta G^{\ddagger}_{A-to-B}$ and $\Delta G^{\ddagger}_{B-to-A}$, respectively. The free energies are given as an average over three experiments (designated 1-3 below).

$$A \leftrightharpoons B$$

$$(3)\ K_{eq} = \frac{k_{A-to-B}}{k_{B-to-A}} = \frac{[B_{eq}]}{[A_{eq}]}$$

$$(4)\ k_{A-to-eq} = k_{A-to-B}\left(1 + \frac{1}{K_{eq}}\right) = k_{B-to-A}(1 + K_{eq})$$

$$(5)\ \Delta G^{\ddagger}_{A-to-B}(T) = RT_c ln\left(\frac{k_B T}{hk_{A-to-B}}\right)$$

$$T\ = 298\ K, R = 8.314\ JK^{-1}mol^{-1},\ k_B = 1.3806 \times 10^{-23}\ JK^{-1}\ and\ h = 6.626 \times\ 10^{-34}\ Js$$

$$\Delta G^{\ddagger}_{A-to-B}(298) = 26.7\ kcalmol^{-1}\ , \Delta G^{\ddagger}_{B-to-A}(298) = 26.7\ kcalmol^{-1}$$

Experiment 1						
**time (s)**	**major (3.70 ppm)**	**minor (3.44 ppm)**	**sum**	**dr**	**de**	**ln(d0/de)**
0	2.06	0.13	2.19	94.06	88.13	0
86400	2.04	0.15	2.19	93.15	86.30	0.020943174
172800	2.02	0.17	2.19	92.24	84.47	0.042334364
259200	2	0.19	2.19	91.32	82.65	0.064193158
354600	1.96	0.22	2.18	89.91	79.82	0.099058223
432000	1.94	0.24	2.18	88.99	77.98	0.122315085
518400	1.9	0.27	2.17	87.56	75.12	0.159765612
604800	1.87	0.29	2.16	86.57	73.15	0.186301834
				**k_{rac}**	3.12697E-07	s-1

Experiment 2						
**time (s)**	**major (3.70 ppm)**	**minor (3.44 ppm)**	**sum**	**dr**	**de**	**ln(d0/de)**
0	1.12	0.03	1.15	97.39	94.78	0
87480	1.17	0.06	1.23	95.12	90.24	0.049069908
168720	1.12	0.06	1.18	94.92	89.83	0.053661284
264720	1.02	0.08	1.1	92.73	85.45	0.103601337
331980	1.01	0.08	1.09	92.66	85.32	0.105164143
419280	1	0.1	1.1	90.91	81.82	0.147086449
530220	0.99	0.12	1.11	89.19	78.38	0.190037837
				**k_{rac}**	3.39823E-07	s-1

Experiment 3						
**time (s)**	**major (3.70 ppm)**	**minor (3.44 ppm)**	**sum**	**dr**	**de**	**ln(d0/de)**
0	0.83	0.06	0.89	93.26	86.52	0
121560	0.86	0.07	0.93	92.47	84.95	0.018320693
192540	0.83	0.08	0.91	91.21	82.42	0.048540445
261540	0.84	0.09	0.93	90.32	80.65	0.070280432
347820	0.74	0.09	0.83	89.16	78.31	0.09962239
434220	0.77	0.1	0.87	88.51	77.01	0.116384551
520440	0.74	0.11	0.85	87.06	74.12	0.154685582
				**k_{rac}**	3.01899E-07	s-1

Table S1: Integration of the peaks at δ3.70 and δ3.44 in the ^{1}H NMR spectrum of **18** over time for experiments 1, 2 and 3, and calculated dr, de and ln(de_0/de_t).

	Experiment 1	Experiment 2	Experiment 3	
K_{eq}	1.02	1.02	1.02	
$k_{a\text{-}eq}$	3.13E-07	3.40E-07	3.02E-07	
$k_{a\text{-}b}$	1.58E-07	1.72E-07	1.52E-07	
**Free energy**	111786.8697	111584.6757	111877.8762	*J*
	26.71770309	26.66937755	26.73945415	*Kcal mol^{-1}*
**Average free energy**		26.70884493		*Kcal mol^{-1}*
**Half-life**	2213160.402	2039728.443	2295960.858	S
	614.7667784	566.5912342	637.7669051	h
	25.61528243	23.60796809	26.57362105	d
K_{eq}	1.02	1.02	1.02	
$k_{a\text{-}eq}$	3.13E-07	3.40E-07	3.02E-07	
$k_{b\text{-}a}$	1.55E-07	1.68E-07	1.49E-07	
**Free energy**	111835.9351	111633.7411	111926.9415	*J*
	26.72943	26.68110446	26.75118106	*Kcal mol^{-1}*
**Average free energy**		26.72057184		*Kcal mol^{-1}*
**Half-life**	2213160.402	2039728.443	2295960.858	s
	614.7667784	566.5912342	637.7669051	h
	25.61528243	23.60796809	26.57362105	d

Table S2: Free energy and half-life calculated from the rate constants, $k_{A\text{-}to\text{-}B}$ and $k_{B\text{-}to\text{-}A}$, and the equilibrium constant, K_{eq} for **18**.

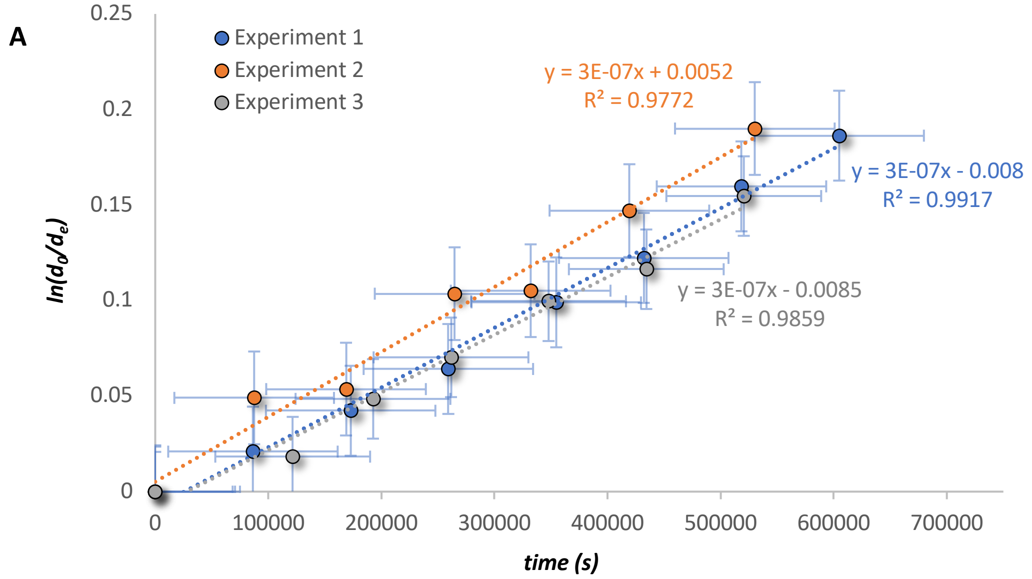

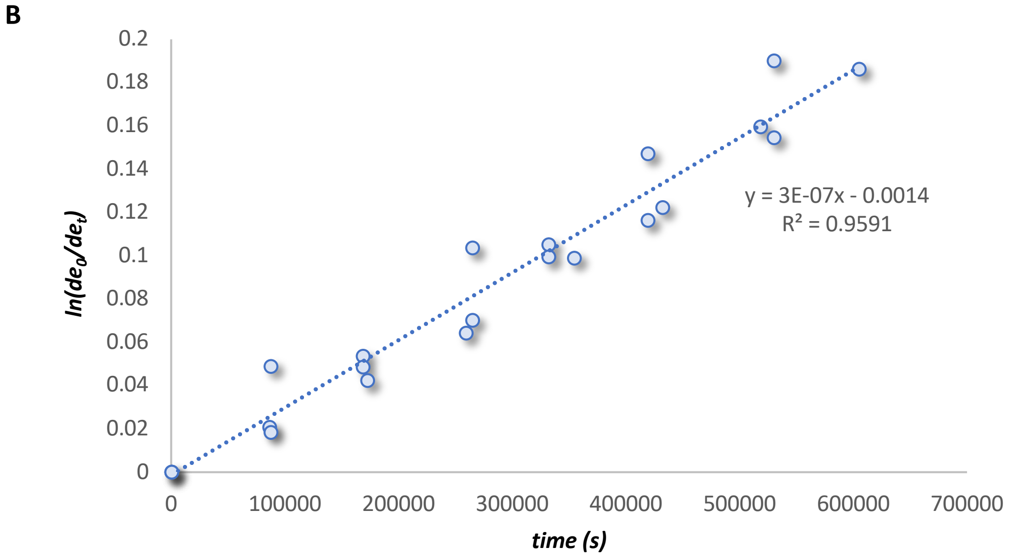

Figure S9: Graphs to show the correlation between $\ln(de_0/de_t)$ and time for inversion barrier experiments 1, 2 and 3 for **18**. **A** linear correlations for each of the three experiments; **B** correlation for all three combined datasets.

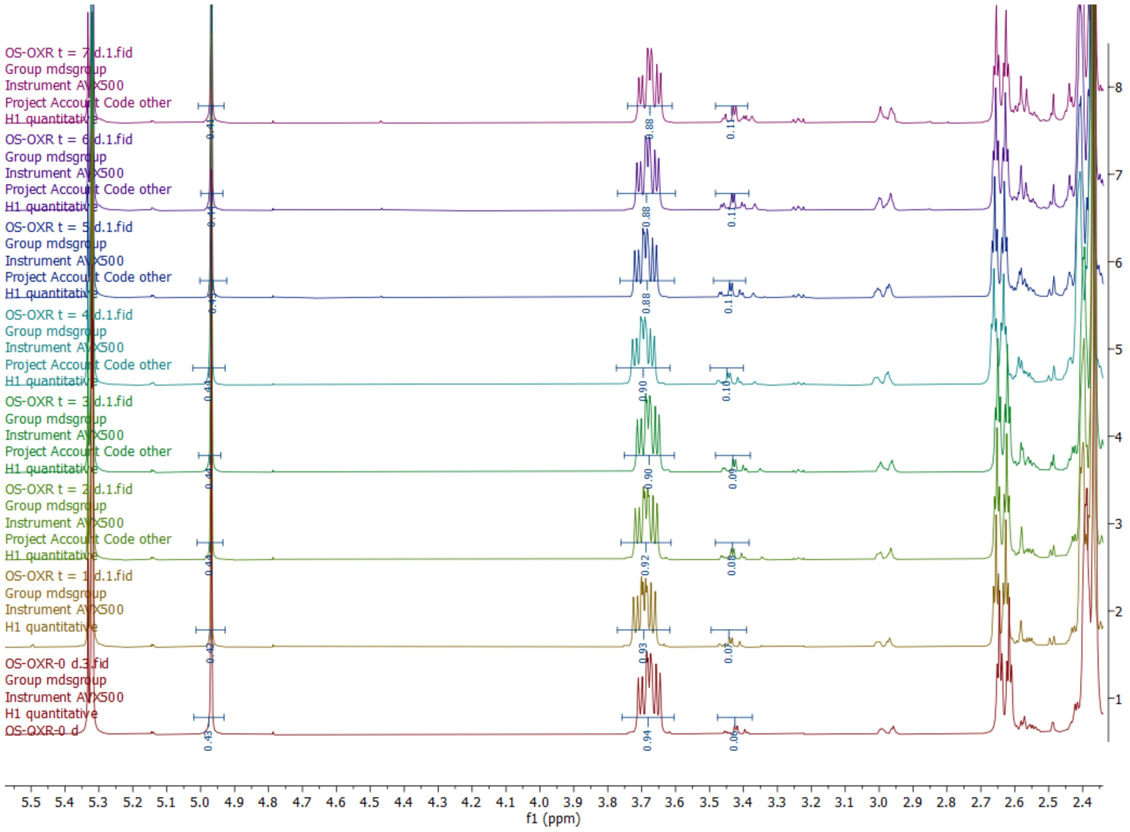

Figure S10: ^{1}H NMR spectra to show the thermal equilibration at 298K of enriched diastereomer **18** over 7 days (starting dr 94:6).

5.3 Enantiomeric ratio erosion study of 11,13-dimethyl-4,5-dihydrobenzo[b]benzo[6,7]oxepino[3,2,1-hi]benzofuran-10-ium hexafluorophosphate, enantioenriched-[*M*, (*S*)*O*]-**17**:‡

Three samples were prepared from the same enantioenriched material **17** at 97:3 e.r., and were dissolved in CH_2Cl_2 in a screw cap vial. The samples were maintained at 298 K for the duration of the experiment (using a Cambridge Reactor Design Polar Bear Plus). Aliquots were taken from each sample over 5 days and dissolved in MeOH. The e.r. was monitored by HPLC.

Chiral HPLC: (2x Chiralpak IC, 95% 100 mM $NaPF_6$ in MeOH, 5% H_2O, 1.0 mL min^{-1}, λ = 270 nm) τ_R (major) = 22.1 min, τ_R (minor) = 23.4 min).

‡ The absolute configuration of the major enantiomer in this 97:3 ratio was correlated with the known absolute configuration of **18** separated in the resolution process. This was achieved by comparing the HPLC retention times (under the above conditions: MeOH/H_2O, 10:1, 100mM $NaPF_6$) of the major/minor enantiomers of **17** with that of a sample of highly diastereoenriched **18** under these conditions. We assume that complete counterion metathesis to generate enantioenriched **17** occurs under these conditions.

A

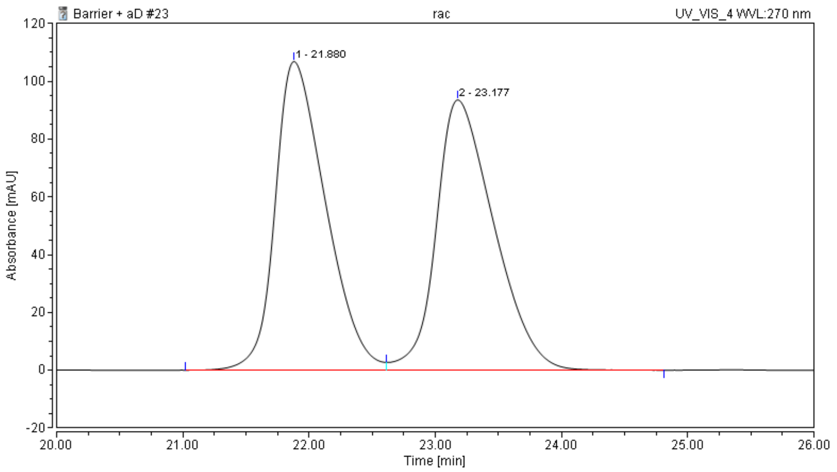

B

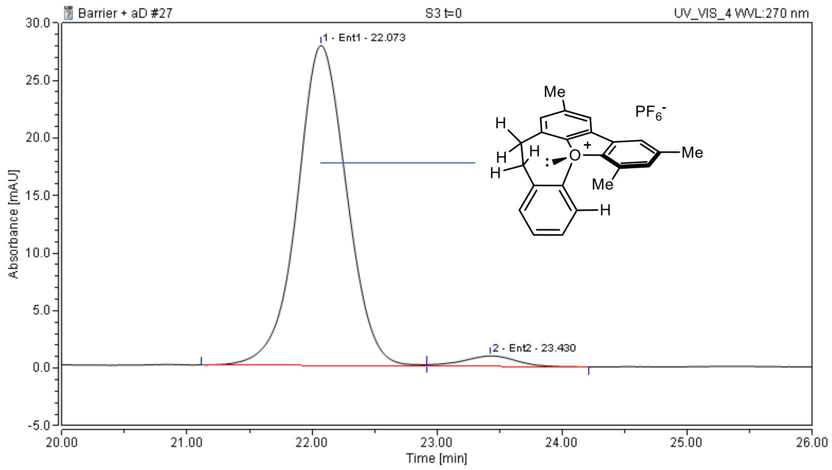

C

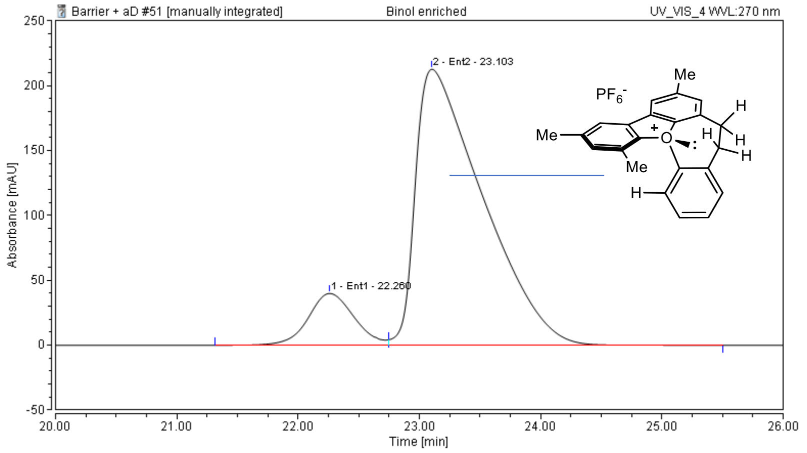

Figure S11: HPLC chromatograms (2x Chiralpak IC, 95% 100 mM $NaPF_6$ in MeOH, 5% H_2O, 1.0 mL min^{-1}, λ = 270 nm). **A** racemic **17**; **B** enantioenriched **17** (major enantiomer [*M*, (*S)*$_O$] after preparative HPLC separation. **C** **18** after *in-situ* counterion metathesis to $[PF_6]^-$ salt (showing absolute configuration: [*P*, (*R)*$_O$]).

	Time (h)	S1	S2	S3	Average
			ee		
**S=0**	0	92.09	93.11	93.03	92.74
**S=1**	12.25	91.43	91.65	90.7	91.26
**S=2**	39.45	86.72	87.52	87.31	87.18
**S=3**	61.25	82.67	85.46	85.27	84.47
**S=4**	85.4	78.7	82.8	82.58	81.36
**S=5**	113.5	73.45	80.14	78.38	77.32

Table S3: Measured erosion of e.r. of enantioenriched **17** over time (3 samples)

	Average ln(ee(0)/ee(t))	Standard deviation	Standard error (SE)
**S=0**	0	0.567215	0.327481976
**S=1**	0.016123246	0.497293	0.287112057
**S=2**	0.06182264	0.414769	0.239467001
**S=3**	0.093478842	1.558856	0.900006173
**S=4**	0.130952069	2.306252	1.331515427
**S=5**	0.181840057	3.467915	2.002201566
**k_{rac}**	4.4259E-07	*s^{-1}*	
**k_{ent}**	2.21295E-07	*s^{-1}*	
**Free energy**	111.0	*kJ mol^{-1}*	
	26.5	*Kcal mol^{-1}*	
**Half life**	1566115.667	s	
	435.0321296	h	
	18.12633873	d	

Table S4: Calculated ln(de_0/d_t) k_{rac}, k_{ent}, standard errors, free energy and half life from the measured e.r. of **17**.

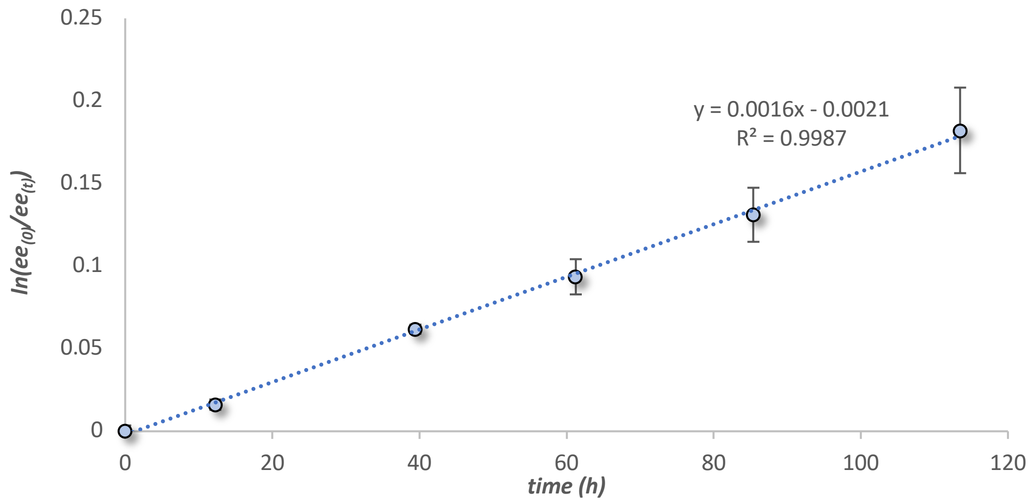

Figure S12: Graph to show the correlation between ln(de_0/de_t) and time for inversion barrier experiments 1, 2 and 3 for compound **17**.

6 Computational Studies

6.1 Computational details

Gaussian 16 version C.01[18] was employed for all density functional theory (DFT) calculations, using an "ultrafine" pruned (99,590) grid for numerical integration of the exchange-correlation functional and its derivatives. Vibrational frequency calculations were used to verify that stationary points were either minima or first-order saddle points on the potential energy surface, and to calculate thermal corrections to Gibbs free energies (G). Intrinsic reaction coordinate (IRC)[19] calculations were performed to ensure that the transition structures connected to their corresponding starting and final geometries and were used to construct **Figure 4** in the main article. The computed thermochemistry data were corrected following Grimme's quasi-harmonic (QHA)[20] model for entropy with a frequency cut-off value of 100.0 cm^{-1} using the GoodVibes[21] program at 298.15 K (25˚C). Molecular graphics were generated using CYLview[22].

The B3LYP[23] functional in conjunction with Grimme's D3 dispersion correction method with Becke–Johnson damping[24] and Pople's double-ζ 6-31+G(d,p) basis set[25] were used in this study, as it performed best in benchmarking optimization against experimentally determined crystal structure of compound **10** (**Table S3**). Inversion barrier height computed for compound **10** also showed little variation with respect to the level of theory and basis set (**Table S4**). The inclusion of solvation effects with implicit solvation models (PCM,[26] CPCM[27] and SMD[28]; solvent=dichloromethane), also did not significantly influence the computed barrier heights (**Table S4**), lowering the barriers by 0.3 to 0.4 $kcal{\cdot}mol^{-1}$ relative to the gas phase barrier. This can be rationalised based on the small change in dipole moment between the ground state (1.08 D) and transition state (1.16 D) structures. Similarly, the influence of the tetrafluoroborate ($BF4^-$) non-coordinating counterion was investigated. Grimme's CREST[29] conformational search workflow was used to sample the conformational space of oxonium ion **10** in the presence of the $BF4^-$ counterion, using an energy window of 12 $kcal{\cdot}mol^{-1}$. The resulting conformational ensembles for the ground state (**10-BF_4-GS**) and transition state (**10-BF_4-TS**) were then filtered using the in-built *CREGEN* function and further optimised at the B3LYP/6-31+G(d,p)-SMD(CH_2Cl_2) level of theory. Further thermochemical corrections and Boltzmann averaging was applied using the *Goodvibes* program. The final conformational space that was studied can be visualised in **Figure S11**. The computed inversion barrier in the presence of the counterion, obtained as an ensemble average over all of the ion-pair conformations, was found to be very similar as the computed barrier in gas phase at the B3LYP/6-31+G(d,p) in the absence of the counterion (13.1 kcal/mol). As such, further computational investigations were performed in the gas phase in the absence of the counterion.

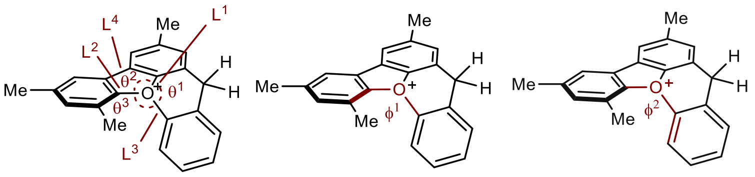

Figure S13: Diagram showing selected bonds, angles and dihedral angles used in benchmarking studies

Theory	Basis Set	L^1 / Å	L^2 / Å	L^3 / Å	L^4 / Å	Θ^1	Θ^2	Θ^3	Φ^1	Φ^2
Experimental		1.459	1.476	1.491	1.458	110.1	104.3	123.9	9.5	-62.3
M06-2X-D3 [30]	6-31+G(d,p)	1.444	1.460	1.471	1.466	111.2	105.2	124.2	5.9	-62.7
M06-2X-D3	Def2-TZVPP [31]	1.440	1.457	1.467	1.462	111.4	105.2	124.6	6.0	-61.7
B3LYP-D3(BJ)	6-31+G(d,p)	1.456	1.477	1.485	1.464	111.0	105.0	124.6	6.8	-63.3
B3LYP-D3(BJ)	Def2-TZVPP	1.452	1.474	1.480	1.460	111.1	105.0	124.9	6.9	-62.9
wb97x-D [32]	6-31+G(d,p)	1.442	1.462	1.472	1.464	111.2	105.2	124.6	5.6	-63.0
wb97x-D	Def2-TZVPP	1.437	1.458	1.467	1.460	111.3	105.2	125.0	5.7	-62.3

Table S5: Compiled selected bond lengths, angles and dihedrals of compound **10** obtained experimentally and computationally at various levels of theory.

Optimization Method	Optimization Basis Set	Single Point Correction Method	Single Point Correction Basis Set	ΔG / kcal·mol^{-1}
Experimental				13.9
B3LYP-D3(BJ)	6-31+G(d,p)	N/A		13.1
**B3LYP-D3(BJ)-SMD(CH_2Cl_2)[(a)]**	6-31+G(d,p)	N/A		13.1[(b)]
**B3LYP-D3(BJ)**	6-31+G(d,p)	B3LYP-D3(BJ)-SMD(CH_2Cl_2)	6-31+G(d,p)	12.7
**B3LYP-D3(BJ)**	6-31+G(d,p)	B3LYP-D3(BJ)-PCM(CH_2Cl_2)	6-31+G(d,p)	12.8
**B3LYP-D3(BJ)**	6-31+G(d,p)	B3LYP-D3(BJ)-CPCM(CH_2Cl_2)	6-31+G(d,p)	12.8
B3LYP-D3(BJ)	6-31+G(d,p)	B3LYP-D3(BJ)	6-311+G(d,p)	12.8
B3LYP-D3(BJ)	6-31+G(d,p)	B3LYP-D3(BJ)	Def2-TZVPP	12.4
B3LYP-D3(BJ)	6-31+G(d,p)	M06-2X-D3	Def2-TZVPP	14.4
B3LYP-D3(BJ)	6-31+G(d,p)	wb97xd	Def2-TZVPP	13.8

Table S6: Compiled free energy of inversion for compound **10** obtained experimentally and computationally at various levels of theory; (a) Calculations include an explicit BF_4^- counterion; (b) Gibbs free energy Boltzmann averaged across conformational ensemble.

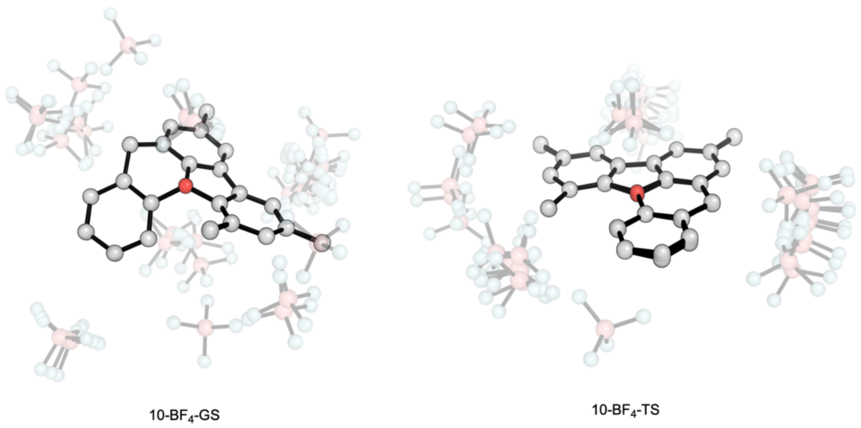

Figure S14: Conformational space sampled for compound **10** when the non-coordinating BF_4^- counterion is included. Graphics show several optimised ion-pair conformations superimposed for ground and transition state structures, generated with PyMol[33].

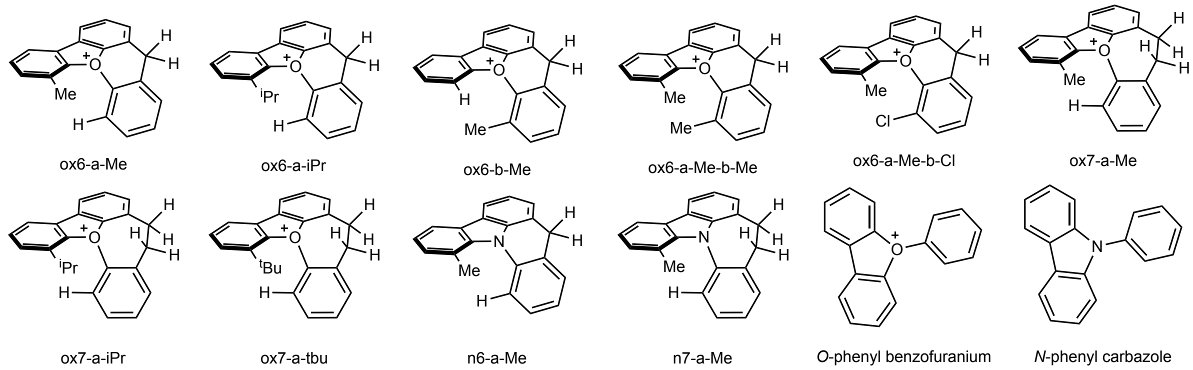

Figure S15: Oxonium ion and triarylamine structures computed in this study.

6.2 Hyperhomodesmotic bond separation reactions

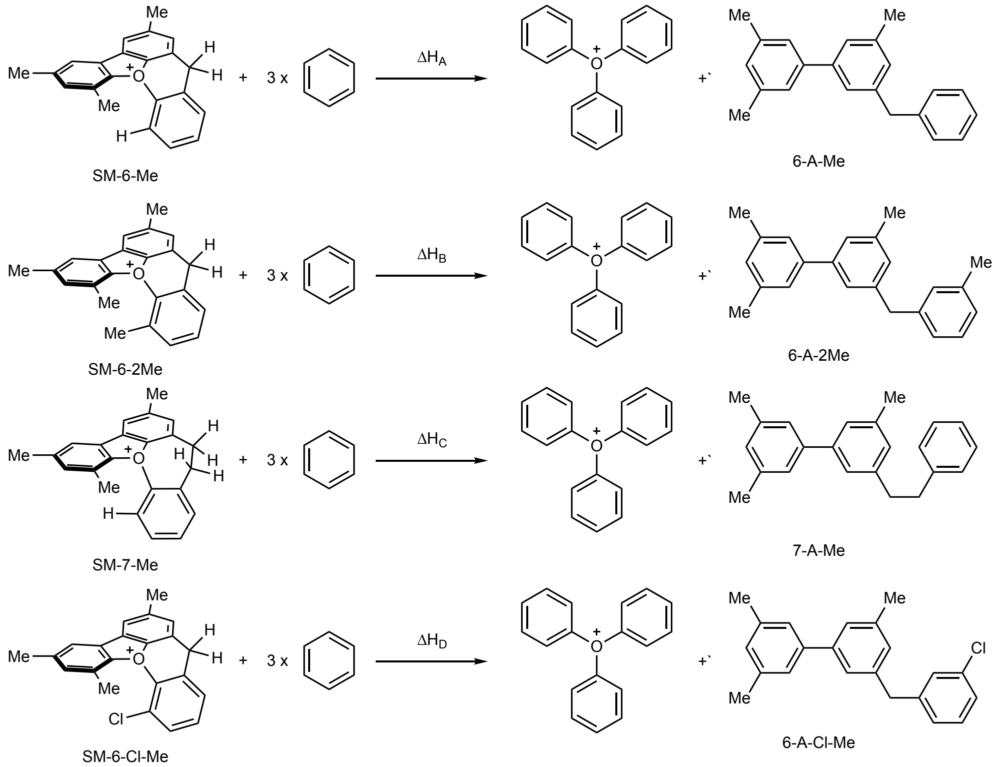

Figure S16: Hyperhomodesmotic transformations used in the estimation of ring strain

Single Point Correction Level of Theory	ΔH_A	ΔH_B	ΔH_C	ΔH_D	$\Delta\Delta(H_A/H_B)$	$\Delta\Delta(H_A/H_C)$	$\Delta\Delta(H_A/H_D)$
N/A	-8.7	-13.0	-4.9	-19.6	4.3	-3.8	10.9
B3LYP-D3(BJ)/6-311+G(d,p)	-8.4	-12.4	-4.7	-18.9	4.0	-3.7	10.5
B3LYP-D3(BJ)/Def2-TZVPP	-6.9	-11.4	-3.4	-17.3	4.5	-3.5	10.4
M06-2X-D3/Def2-TZVPP	-7.1	-11.5	-3.5	-18.1	4.4	-3.6	11.0
wb97x-D[29]/Def2-TZVPP	-5.6	-10.0	-2.5	-17.2	4.4	-3.1	11.6

Table S7: Compiled reaction enthalpies (kcal·mol^{-1}) for hyperhomodesmotic transformations; geometries were optimised at the B3LYP-D3(BJ)/6-31+G(d,p) level of theory

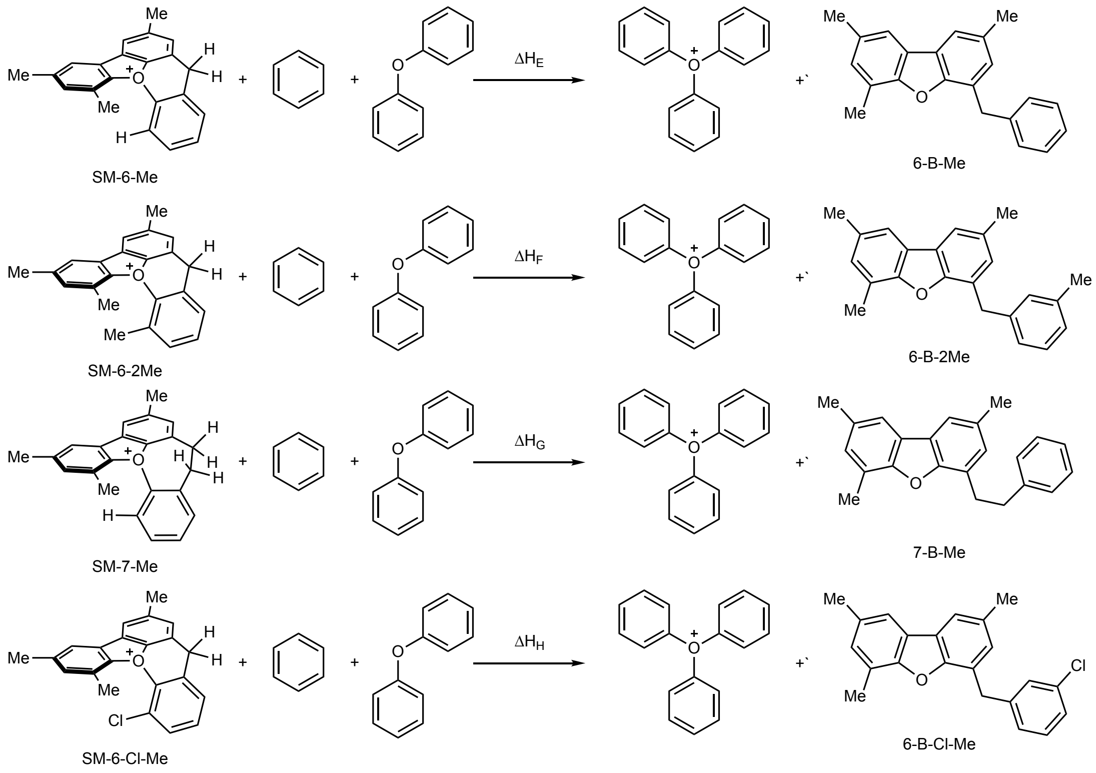

Figure S17: Hyperhomodesmotic transformations used in the estimation of ring strain

Single Point Correction Level of Theory	ΔH_E	ΔH_F	ΔH_G	ΔH_H	$\Delta\Delta(H_E/H_F)$	$\Delta\Delta(H_E/H_G)$	$\Delta\Delta(H_E/H_H)$
N/A	-10.6	-14.9	-7.4	-21.8	4.3	-3.2	11.2
B3LYP-D3(BJ)/6-311+G(d,p)	-10.3	-14.4	-7.3	-21.0	4.1	-3.0	10.7
B3LYP-D3(BJ)/Def2-TZVPP	-9.4	-13.9	-6.4	-20.0	4.5	-3.0	10.6
M06-2X-D3/Def2-TZVPP	-9.4	-13.9	-6.3	-20.6	4.5	-3.1	11.2
wb97xD/Def2-TZVPP	-9.3	-13.9	-6.6	-21.0	4.6	-2.7	11.7

Table S8: Compiled reaction enthalpies (kcal・mol^{-1}) for hyperhomodesmotic transformations; geometries were optimised at the B3LYP-D3(BJ)/6-31+G(d,p) level of theory

6.3 NBO analysis on ring size influence

In order to further deconvolute the influence of ring size on the enantiomerization activation energy barrier, natural bonding orbital (NBO) analysis was performed for the oxonium ions ox6-a-Me and ox7-a-Me. Calculations used NBO7[34] interfaced with Gaussian 16 version C.01. Second-order perturbation theory analysis of the Fock matrices was used to estimate donor-acceptor (i.e. filled to empty) interactions in the NBO basis. Delocalization energies of the oxygen lone pair into adjacent π^* orbitals (for both GS and TS structures) have been compiled in **Table S9**. These results suggest that for ox6-a-Me, the increasing planarity about oxygen in the TS results in a stabilization from increased delocalization by 14.0 kcal mol^{-1} relative to the GS. In contrast, for ox7-a-Me, there is very little change in delocalization between GS and TS, with the stabilization reduced to 2.9 kJ mol^{-1}.

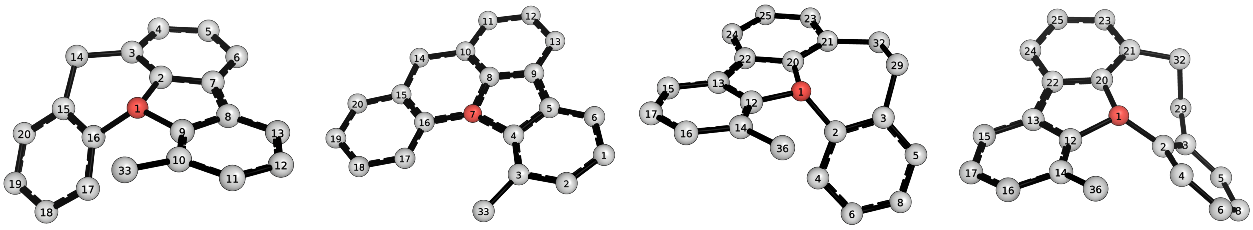

ox6-a-Me ox6-a-Me-TS ox7-a-Me ox7-a-Me-TS

Figure S18: Three-dimensional structures of ox6-a-Me, ox6-a-Me-TS, ox7-a-Me and ox-7-a-Me-TS generated using PyMol, together with their corresponding atom indices.

Entry	Donor	Acceptor	E / kcal mol^{-1}
**ox6-a-Me**	LP(1)O1	BD*(1)C2-C7	0.77
		BD*(2)C2-C7	7.43
		BD*(1)C8-C9	0.77
		BD*(2)C9-C10	6.59
		BD*(2)C16-C17	0.96
		BD*(2)C16-C17	7.65
		RY*(5)C2	0.72
**ox6-a-Me-TS**	LP(1)O7	BD*(2)C3-C4	9.55
		BD*(2)C8-C10	14.61
		BD*(2)C15-C16	11.50
**ox7-a-Me**	LP(1)O1	BD*(1)C2-C3	0.79
		BD*(1)C2-C4	2.59
		BD*(2)C2-C4	3.87
		BD*(1)C12-C13	0.85
		BD*(2)C12-C14	6.82
		BD*(2)C20-C21	6.45
**ox7-a-Me-TS**	LP(1)O1	BD*(1)C2-C3	1.54
		BD*(2)C2-C3	5.80
		BD*(1)C12-C13	0.55
		BD*(2)C12-C14	5.61
		BD*(2)C20-C22	8.51
		RY(3)C2	0.59

Table S9: Second-order perturbative estimates of the oxygen lone pair delocalization energies for compounds ox6-a-Me and ox7-a-Me

6.4 Comparison between triaryl oxonium ions and triaryl amines

To elucidate the influence of the heteroatom on the activation energy barrier of enantiomerization, the corresponding nitrogen-containing triaryl amines were also investigated computationally (**Figure S19**) at the same level of theory.

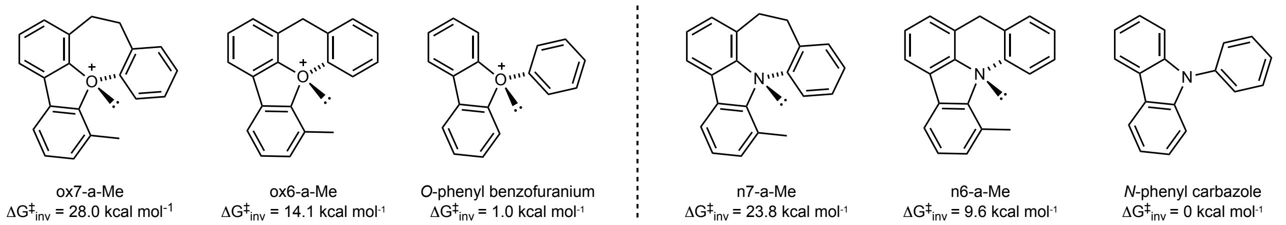

Figure S19: Three-dimensional structures of ox6-a-Me, ox6-a-Me-TS, ox7-a-Me, and ox-7-a-Me-TS, together with their corresponding atom indices.

The computed barriers to enantiomerization for the triaryl amines were found to be consistently higher than their oxonium counterpart. The ground state structure of *N*-phenyl carbazole is completely planar. NBO calculations performed on these structures revealed increased delocalization of the heteroatom lone pair in the triarylamine heterocycles, as evidenced by shortened C-N bond lengths, larger C-N Wiberg bond orders (WBO), and decreased nitrogen lone pair orbital occupancy compared to the corresponding oxonium ions (**Figure S20**). Consequently, due to the increased delocalization of the nitrogen atom lone pair, there is little change in lone pair hybridization between GS and TS structures. For n6-a-Me, the p-character of the nitrogen atom changes from 96%p to 100%p from GS to TS, while for n7-a-Me, the p-character changes from 96%p to 97%p. On the other hand, ox6-a-Me has a much larger change in the hybridization of the oxygen atom from 86%p to 100%p on going from GS to TS, while the ox7-a-Me compound has a more modest change from 86%p to 89%p. Compared to triaryl amines, the ground state structures of oxonium ions are characterized by lower lone-pair p-character, less delocalization, and greater pyramidalization of the central heteroatom. Consequently, there is an increase in enantiomerization activation energy barrier due to the associated increase in structural distortion necessary to achieve planarity (or near planarity) in the TS.

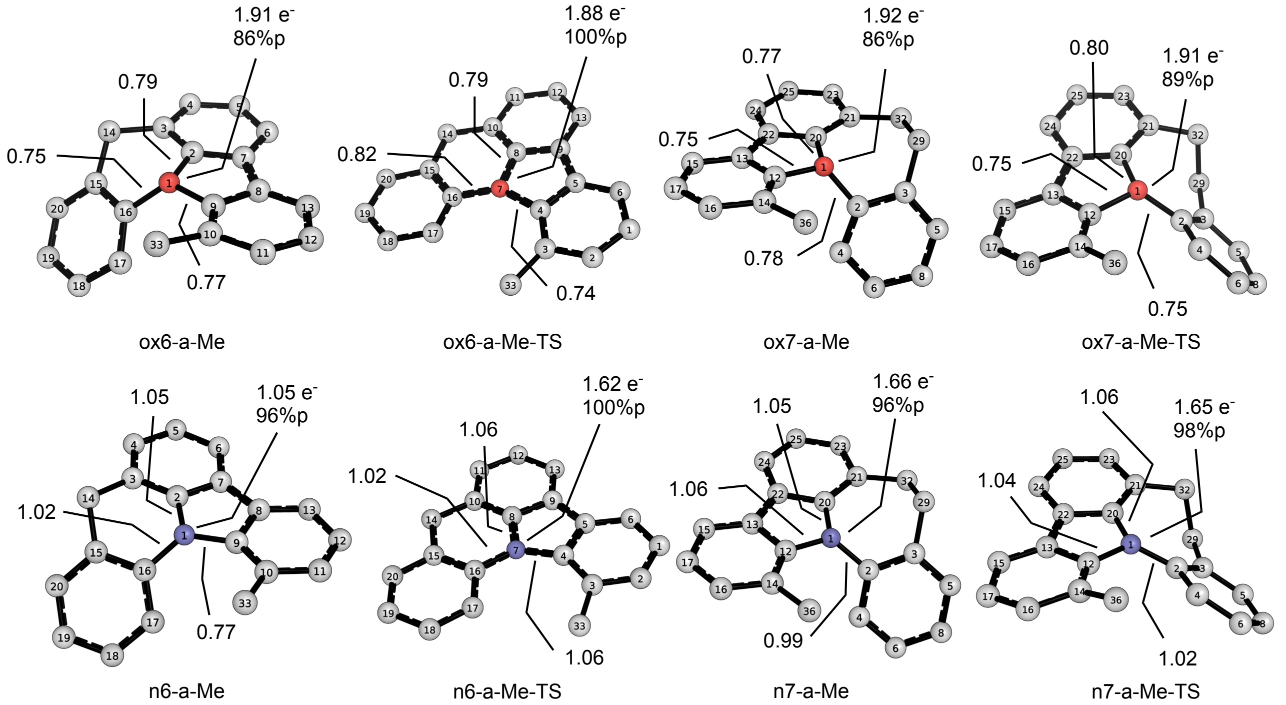

Figure S20: Optimised structures of ox6-a-Me, ox6-a-Me-TS, ox7-a-Me, ox-7-a-Me-TS, n6-a-Me, n6-a-Me-TS, n7-a-Me and n7-a-Me-TS generated using PyMol, together with corresponding Wiberg bond orders, lone pair occupancy and %p character of the heteroatom

6.5 Thermochemical data

Name	E / $a.u.$	ZPE / $a.u.$	H / $a.u.$	$T \cdot S$ / $a.u.$	G / $a.u$	v_{imag} / cm^{-1}
**10-BF4-GS_1**	-1349.647827	0.360068	-1349.261207	0.078266	-1349.339474	
**10-BF4-GS_10**	-1349.645530	0.359786	-1349.259180	0.078147	-1349.337328	
**10-BF4-GS_13**	-1349.645485	0.359716	-1349.259063	0.078851	-1349.337914	
**10-BF4-GS_16**	-1349.645767	0.359804	-1349.259553	0.077781	-1349.337333	
**10-BF4-GS_17**	-1349.644987	0.359797	-1349.258602	0.078106	-1349.336708	
**10-BF4-GS_18**	-1349.644419	0.359666	-1349.258016	0.079540	-1349.337556	
**10-BF4-GS_19**	-1349.644428	0.359845	-1349.257889	0.079429	-1349.337318	
**10-BF4-GS_20**	-1349.644633	0.359890	-1349.258089	0.079022	-1349.337112	
**10-BF4-GS_21**	-1349.644645	0.359891	-1349.258083	0.079001	-1349.337085	
**10-BF4-GS_22**	-1349.643938	0.359979	-1349.257334	0.078844	-1349.336178	
**10-BF4-GS_23**	-1349.644177	0.359459	-1349.257844	0.080491	-1349.338335	
**10-BF4-GS_24**	-1349.644151	0.359679	-1349.257674	0.080862	-1349.338535	
**10-BF4-GS_25**	-1349.644165	0.359672	-1349.257667	0.079862	-1349.337529	
**10-BF4-GS_26**	-1349.644167	0.359713	-1349.257657	0.079620	-1349.337277	
**10-BF4-GS_27**	-1349.644157	0.359763	-1349.257638	0.079574	-1349.337212	
**10-BF4-GS_28**	-1349.644219	0.359753	-1349.257698	0.079539	-1349.337237	
**10-BF4-GS_30**	-1349.644164	0.359601	-1349.257680	0.080204	-1349.337884	
**10-BF4-GS_31**	-1349.644067	0.359633	-1349.257637	0.079305	-1349.336943	
**10-BF4-GS_32**	-1349.644171	0.359809	-1349.257647	0.079216	-1349.336864	
**10-BF4-GS_34**	-1349.644235	0.359781	-1349.257668	0.079521	-1349.337189	
**10-BF4-GS_37**	-1349.644270	0.359976	-1349.257671	0.078788	-1349.336459	
**10-BF4-GS_38**	-1349.644184	0.359697	-1349.257670	0.079568	-1349.337239	
**10-BF4-GS_39**	-1349.644195	0.359916	-1349.257595	0.079117	-1349.336712	
**10-BF4-GS_40**	-1349.644204	0.359955	-1349.257567	0.079188	-1349.336754	
**10-BF4-GS_41**	-1349.644179	0.359716	-1349.257660	0.079621	-1349.337281	
**10-BF4-GS_42**	-1349.644183	0.359901	-1349.257591	0.079010	-1349.336601	
**10-BF4-GS_44**	-1349.644238	0.359950	-1349.257609	0.079234	-1349.336842	
**10-BF4-GS_45**	-1349.644160	0.359785	-1349.257622	0.079295	-1349.336917	
**10-BF4-GS_46**	-1349.643071	0.359835	-1349.256573	0.079092	-1349.335665	
**10-BF4-GS_47**	-1349.643269	0.359666	-1349.256829	0.079707	-1349.336536	
**10-BF4-GS_48**	-1349.643310	0.359620	-1349.256933	0.079236	-1349.336169	
**10-BF4-GS_49**	-1349.642484	0.359874	-1349.255911	0.079493	-1349.335405	
**10-BF4-GS_5**	-1349.647653	0.359938	-1349.261098	0.078359	-1349.339456	
**10-BF4-GS_50**	-1349.642421	0.359892	-1349.255959	0.079114	-1349.335073	
**10-BF4-GS_51**	-1349.642450	0.359855	-1349.255911	0.079470	-1349.335381	
**10-BF4-GS_52**	-1349.645826	0.359770	-1349.259388	0.078956	-1349.338344	
**10-BF4-GS_58**	-1349.644542	0.359660	-1349.258131	0.079434	-1349.337565	
**10-BF4-GS_59**	-1349.644189	0.359582	-1349.257784	0.079694	-1349.337478	
**10-BF4-TS_1**	-1349.627011	0.359991	-1349.241060	0.076973	-1349.318033	-70.95
**10-BF4-TS_10**	-1349.623734	0.359872	-1349.237683	0.078886	-1349.316570	-86.95
**10-BF4-TS_11**	-1349.623805	0.359580	-1349.237970	0.078647	-1349.316618	-70.89

**10-BF4-TS_13**	-1349.623778	0.359739	-1349.237814	0.078542	-1349.316356	-66.59
**10-BF4-TS_14**	-1349.623744	0.359891	-1349.237699	0.078829	-1349.316528	-88.54
**10-BF4-TS_15**	-1349.623648	0.359828	-1349.237692	0.078355	-1349.316047	-93.80
**10-BF4-TS_17**	-1349.623725	0.359713	-1349.237803	0.078495	-1349.316298	-93.99
**10-BF4-TS_18**	-1349.623733	0.360133	-1349.237491	0.078109	-1349.315600	-84.02
**10-BF4-TS_19**	-1349.623262	0.359958	-1349.237208	0.077963	-1349.315172	-69.06
**10-BF4-TS_2**	-1349.626868	0.359745	-1349.241029	0.077417	-1349.318446	-73.54
**10-BF4-TS_20**	-1349.623782	0.359983	-1349.237642	0.078407	-1349.316049	-78.69
**10-BF4-TS_22**	-1349.623729	0.360018	-1349.237637	0.077679	-1349.315316	-83.71
**10-BF4-TS_24**	-1349.623364	0.360135	-1349.237205	0.077640	-1349.314845	-86.02
**10-BF4-TS_26**	-1349.623418	0.359867	-1349.237348	0.078598	-1349.315946	-81.21
**10-BF4-TS_28**	-1349.623732	0.359885	-1349.237696	0.078196	-1349.315892	-87.77
**10-BF4-TS_29**	-1349.623616	0.360006	-1349.237483	0.078083	-1349.315566	-82.48
**10-BF4-TS_3**	-1349.626768	0.359721	-1349.240961	0.077426	-1349.318387	-78.36
**10-BF4-TS_30**	-1349.623646	0.360039	-1349.237523	0.078031	-1349.315554	-84.67
**10-BF4-TS_31**	-1349.623675	0.359973	-1349.237582	0.078248	-1349.315830	-88.51
**10-BF4-TS_32**	-1349.622935	0.359664	-1349.237081	0.078361	-1349.315442	-73.21
**10-BF4-TS_37**	-1349.622948	0.359691	-1349.237084	0.078242	-1349.315326	-82.01
**10-BF4-TS_38**	-1349.626993	0.359879	-1349.241063	0.077388	-1349.318452	-76.15
**10-BF4-TS_39**	-1349.623516	0.359571	-1349.237749	0.078124	-1349.315873	-66.26
**10-BF4-TS_4**	-1349.623999	0.359513	-1349.238216	0.078330	-1349.316545	-95.99
**10-BF4-TS_40**	-1349.622669	0.359702	-1349.236820	0.078024	-1349.314844	-86.99
**10-BF4-TS_41**	-1349.622666	0.359634	-1349.236826	0.078285	-1349.315111	-86.01
**10-BF4-TS_42**	-1349.622513	0.359362	-1349.236877	0.079019	-1349.315897	-94.87
**10-BF4-TS_43**	-1349.622520	0.359527	-1349.236797	0.078641	-1349.315438	-90.38
**10-BF4-TS_45**	-1349.623733	0.359831	-1349.237741	0.078279	-1349.316020	-82.10
**10-BF4-TS_46**	-1349.623827	0.359614	-1349.237942	0.078675	-1349.316617	-66.43
**10-BF4-TS_47**	-1349.622722	0.359740	-1349.236830	0.078068	-1349.314898	-88.32
**10-BF4-TS_50**	-1349.623806	0.359555	-1349.237979	0.078697	-1349.316677	-69.77
**10-BF4-TS_51**	-1349.621643	0.359393	-1349.236005	0.078998	-1349.315004	-87.97
**10-BF4-TS_52**	-1349.622102	0.359545	-1349.236195	0.080104	-1349.316300	-74.20
**10-BF4-TS_53**	-1349.623212	0.359721	-1349.237265	0.078912	-1349.316177	-77.55
**10-BF4-TS_54**	-1349.623726	0.359843	-1349.237756	0.078174	-1349.315930	-94.11
**10-BF4-TS_6**	-1349.623688	0.360018	-1349.237593	0.077816	-1349.315408	-79.56
**10-BF4-TS_7**	-1349.623793	0.360040	-1349.237613	0.078659	-1349.316272	-77.55
**10-BF4-TS_9**	-1349.623619	0.359952	-1349.237532	0.078706	-1349.316237	-54.14
**6-A-2Me-1**	-891.105543	0.399508	-890.682287	0.074518	-890.756805	
**6-A-2Me-10**	-891.104883	0.399443	-890.681639	0.075190	-890.756829	
**6-A-2Me-11**	-891.104877	0.399440	-890.681632	0.075336	-890.756968	
**6-A-2Me-12**	-891.105029	0.399454	-890.681790	0.075060	-890.756850	
**6-A-2Me-13**	-891.105442	0.399496	-890.682178	0.074729	-890.756907	
**6-A-2Me-14**	-891.105032	0.399451	-890.681796	0.074964	-890.756760	
**6-A-2Me-2**	-891.104877	0.399441	-890.681632	0.075353	-890.756985	
**6-A-2Me-3**	-891.105529	0.399501	-890.682266	0.074564	-890.756831	
**6-A-2Me-4**	-891.105430	0.399487	-890.682161	0.074863	-890.757025	

**6-A-2Me-5**	-891.104824	0.399539	-890.681602	0.074319	-890.755921
**6-A-2Me-6**	-891.105033	0.399438	-890.681806	0.075006	-890.756812
**6-A-2Me-7**	-891.104970	0.399476	-890.681727	0.074811	-890.756538
**6-A-2Me-8**	-891.105175	0.399506	-890.681918	0.074771	-890.756689
**6-A-2Me-9**	-891.105123	0.399486	-890.681867	0.074892	-890.756759
**6-A-Me-1**	-851.779186	0.372214	-851.385088	0.070890	-851.455978
**6-A-Me-2**	-851.778684	0.372221	-851.384595	0.070803	-851.455398
**6-A-Me-3**	-851.778795	0.372232	-851.384705	0.070748	-851.455453
**6-A-Me-4**	-851.778835	0.372245	-851.384731	0.070727	-851.455458
**6-A-Me-5**	-851.778796	0.372238	-851.384704	0.070674	-851.455378
**6-A-Me-6**	-851.779186	0.372217	-851.385090	0.070845	-851.455935
**6-A-Me-7**	-851.778833	0.372230	-851.384730	0.071365	-851.456096
**6-A-Me-Cl-1**	-1311.377860	0.362643	-1310.992157	0.073546	-1311.065703
**6-A-Me-Cl-10**	-1311.377387	0.362660	-1310.991648	0.073839	-1311.065487
**6-A-Me-Cl-11**	-1311.377352	0.362656	-1310.991618	0.073952	-1311.065570
**6-A-Me-Cl-12**	-1311.377387	0.362634	-1310.991687	0.073820	-1311.065507
**6-A-Me-Cl-13**	-1311.377784	0.362678	-1310.992038	0.073730	-1311.065768
**6-A-Me-Cl-14**	-1311.377390	0.362600	-1310.991695	0.074353	-1311.066048
**6-A-Me-Cl-2**	-1311.377352	0.362657	-1310.991618	0.073950	-1311.065568
**6-A-Me-Cl-3**	-1311.377847	0.362627	-1310.992136	0.073712	-1311.065847
**6-A-Me-Cl-4**	-1311.377767	0.362649	-1310.992018	0.074180	-1311.066198
**6-A-Me-Cl-5**	-1311.377352	0.362659	-1310.991621	0.073926	-1311.065548
**6-A-Me-Cl-6**	-1311.377401	0.362652	-1310.991707	0.073444	-1311.065151
**6-A-Me-Cl-7**	-1311.377502	0.362674	-1310.991770	0.073649	-1311.065419
**6-A-Me-Cl-8**	-1311.377539	0.362623	-1310.991836	0.073985	-1311.065821
**6-A-Me-Cl-9**	-1311.377514	0.362635	-1310.991792	0.074061	-1311.065853
**6-B-2Me-1**	-965.126357	0.382722	-964.720376	0.072753	-964.793129
**6-B-2Me-2**	-965.126236	0.382638	-964.720305	0.072919	-964.793225
**6-B-2Me-3**	-965.125936	0.382701	-964.719957	0.072625	-964.792583
**6-B-2Me-4**	-965.126074	0.382833	-964.720003	0.072734	-964.792736
**6-B-2Me-5**	-965.126357	0.382844	-964.720268	0.072641	-964.792909
**6-B-Me-1**	-925.799939	0.355459	-925.423132	0.068512	-925.491644
**6-B-Me-2**	-925.799929	0.355610	-925.422998	0.068370	-925.491368
**6-B-Me-Cl-1**	-1385.399415	0.345997	-1385.030925	0.070913	-1385.101838
**6-B-Me-Cl-2**	-1385.398792	0.345852	-1385.030377	0.071409	-1385.101787
**6-B-Me-Cl-3**	-1385.398042	0.345866	-1385.029583	0.071763	-1385.101347
**6-B-Me-Cl-4**	-1385.398686	0.346050	-1385.030103	0.071334	-1385.101437
**6-B-Me-Cl-5**	-1385.399415	0.345997	-1385.030925	0.070912	-1385.101837
**7-A-Me-1**	-812.449082	0.346400	-812.083391	0.065097	-812.148488
**7-A-Me-10**	-812.449076	0.346386	-812.083384	0.065178	-812.148562
**7-A-Me-11**	-812.454213	0.346933	-812.088313	0.063302	-812.151615
**7-A-Me-12**	-812.453528	0.346856	-812.087666	0.063639	-812.151305
**7-A-Me-13**	-812.451793	0.346679	-812.086026	0.064313	-812.150339
**7-A-Me-14**	-812.451712	0.346672	-812.085954	0.064201	-812.150155
**7-A-Me-2**	-812.451027	0.346650	-812.085290	0.064207	-812.149496

**7-A-Me-3**	-812.451793	0.346701	-812.086019	0.064129	-812.150148	
**7-A-Me-4**	-812.451004	0.346655	-812.085258	0.064245	-812.149503	
**7-A-Me-5**	-812.450989	0.346645	-812.085242	0.064316	-812.149558	
**7-A-Me-6**	-812.451022	0.346670	-812.085270	0.064195	-812.149465	
**7-A-Me-7**	-812.451028	0.346650	-812.085289	0.064204	-812.149493	
**7-A-Me-8**	-812.449135	0.346402	-812.083447	0.065045	-812.148492	
**7-A-Me-9**	-812.449125	0.346381	-812.083440	0.065234	-812.148674	
**7-B-Me-1**	-886.472193	0.329645	-886.123715	0.063494	-886.187209	
**7-B-Me-2**	-886.473973	0.329806	-886.125481	0.063038	-886.188520	
**7-B-Me-3**	-886.476271	0.330017	-886.127782	0.061762	-886.189545	
**C6H6**	-232.287286	0.100497	-232.181441	0.032824	-232.214265	
**E**	-576.702089					
**F**	-576.706658					
**G**	-616.021128					
**H**	-616.008193					
**N-phenyl-carbazole**	-748.642493	0.257501	-748.370553	0.053691	-748.424245	
**O-phenyl-benzoduranium-TS**	-768.798989	0.255859	-768.529083	0.052896	-768.581980	-40.70
**O-phenyl-benzoduranium**	-768.800035	0.256076	-768.529167	0.054445	-768.583611	
**Ph2O**	-538.594283	0.185231	-538.398110	0.046503	-538.444612	
**Ph3O+**	-770.000999	0.277311	-769.707535	0.057346	-769.764881	
**SM-6-2Me**	-964.219667	0.373436	-963.824456	0.067934	-963.892390	
**SM-6-Cl-Me**	-1384.480878	0.335461	-1384.123813	0.068005	-1384.191818	
**SM-6-Me**	-924.900062	0.345769	-924.534185	0.064773	-924.598959	
**SM-7-Me**	-885.575527	0.320362	-885.237813	0.059031	-885.296844	
**n6-a-Me-TS**	-826.069407	0.293082	-825.761280	0.054456	-825.815736	-94.65
**n6-a-Me**	-826.083509	0.292610	-825.775061	0.056052	-825.831113	
**n7-a-Me-1**	-865.409317	0.321654	-865.070747	0.058156	-865.128903	
**n7-a-Me-TS**	-865.372893	0.322196	-865.034747	0.056126	-865.090874	-92.16
**ox6-H-TS**	-806.908696	0.263096	-806.631421	0.052936	-806.684357	-79.91
**ox6-H**	-806.915549	0.263659	-806.637337	0.053585	-806.690922	
**ox6-a-Cl-b-Me-TS**	-1305.784390	0.278039	-1305.488644	0.060177	-1305.548821	-26.82
**ox6-a-Cl-b-Me**	-1305.824625	0.281069	-1305.525896	0.059899	-1305.585795	
**ox6-a-Me-TS**	-846.221794	0.291309	-845.914881	0.055553	-845.970434	-93.77
**ox6-a-Me-b-Me-TS**	-885.507343	0.318349	-885.171898	0.058242	-885.230140	-93.64
**ox6-a-Me-b-Me**	-885.563552	0.319020	-885.226689	0.059833	-885.286522	
**ox6-a-Me**	-846.243659	0.291333	-845.936126	0.056759	-845.992885	
**ox6-a-iPr-TS**	-924.862654	0.347859	-924.496413	0.061016	-924.557429	-65.44
**ox6-a-iPr**	-924.890632	0.348172	-924.523553	0.061993	-924.585546	
**ox6-b-Me-TS**	-846.214393	0.291320	-845.907679	0.055022	-845.962701	-86.31
**ox6-b-Me**	-846.234364	0.291359	-845.926865	0.056534	-845.983399	
**ox7-a-Me-1**	-885.575527	0.320362	-885.237813	0.059031	-885.296844	
**ox7-a-Me-2**	-885.562895	0.320097	-885.225276	0.059437	-885.284713	
**ox7-a-Me-TS**	-885.532520	0.320647	-885.195555	0.056739	-885.252294	-73.17
**ox7-a-Me-flip-TS**	-885.562366	0.319888	-885.225635	0.058113	-885.283748	-96.30

**ox7-a-iPr-1**	-964.222406	0.377222	-963.825127	0.064209	-963.889336	
**ox7-a-iPr-2**	-964.210020	0.376933	-963.812859	0.064637	-963.877496	
**ox7-a-iPr-TS**	-964.174714	0.377021	-963.778512	0.062345	-963.840857	-50.55
**ox7-a-tBu-1**	-1003.539358	0.405399	-1003.112789	0.066004	-1003.178793	
**ox7-a-tBu-2**	-1003.528463	0.405092	-1003.102037	0.066417	-1003.168455	
**ox7-a-tBu-TS**	-1003.481295	0.405314	-1003.055651	0.064334	-1003.119985	-46.01

Table S10: Compiled thermochemical data for structures computed at the B3LYP-D3/6-31+G(d,p) level of theory

6.6 Cartesian coordinates for stationary points

47
10-BF4-GS_1 Eopt
-1349.647827
O -0.119303 -0.311896 -0.365234
C -0.373447 1.113682 -0.330469
C -1.653422 1.594955 -0.233440
C -1.730964 2.992730 -0.237821
C -0.574197 3.798614 -0.335379
C -0.718469 5.300533 -0.309345
C 0.698700 3.213811 -0.450242
C 0.806054 1.818261 -0.456951
C 1.890668 0.840013 -0.528086
C 1.343035 -0.447017 -0.436042
C 2.047250 -1.616178 -0.226038
C 3.445314 -1.439151 -0.278547
C 4.065845 -0.193457 -0.455363
C 3.276753 0.964720 -0.550439
C 5.568953 -0.091949 -0.513755
C 1.440670 -2.954337 0.099024
C -2.765896 0.579329 -0.171034
C -2.411097 -0.593851 -1.064330
C -1.092851 -1.021040 -1.219196
C -0.661837 -1.981734 -2.110523
C -1.641447 -2.629813 -2.869979
C -2.985247 -2.281452 -2.725674
C -3.359615 -1.262958 -1.845763
H -2.705282 3.467375 -0.164312
H 0.228767 5.793977 -0.541974
H -1.469610 5.637533 -1.031572
H -1.043028 5.641325 0.680881
H 1.582399 3.837714 -0.531856
H 4.066113 -2.318782 -0.132653
H 3.736973 1.945708 -0.607542
H 5.910269 -0.024621 -1.554185
H 5.924601 0.803081 0.005772
H 6.044293 -0.967488 -0.063397
H 1.432255 -3.613588 -0.775588
H 0.420527 -2.858074 0.472633
H 2.041817 -3.440529 0.872143
H -3.717351 1.013485 -0.483531
H -2.877198 0.237157 0.865669
H 0.385056 -2.220120 -2.231589
H -1.341002 -3.397674 -3.574654
H -3.743064 -2.786755 -3.315507
H -4.400251 -0.964288 -1.764957
B -0.905347 -0.915276 2.934148
F -1.301618 -1.840724 1.941373
F -0.696439 -1.595719 4.154285
F 0.290090 -0.276316 2.539383
F -1.923549 0.053187 3.100927
47
10-BF4-GS_10 Eopt
-1349.645530
O -0.303786 -0.749080 -1.150644
C 0.342867 0.547654 -1.238462
C 1.623330 0.661516 -1.712171
C 2.112768 1.972631 -1.702994
C 1.329372 3.052958 -1.238963
C 1.922100 4.439367 -1.218370
C 0.025407 2.838414 -0.761561
C -0.485849 1.536045 -0.749748
C -1.745317 0.899139 -0.364658
C -1.656998 -0.473760 -0.633039
C -2.702605 -1.373652 -0.621338
C -3.905993 -0.820747 -0.135882
C -4.047902 0.524028 0.239120
C -2.959189 1.400917 0.090099
C -5.361464 1.030652 0.776436
C -2.646446 -2.794383 -1.118713
C 2.322733 -0.609218 -2.125861
C 1.903227 -1.732708 -1.194897
C 0.623762 -1.792971 -0.646169
C 0.207579 -2.680080 0.323968
C 1.131739 -3.646327 0.738142
C 2.414980 -3.669506 0.190546
C 2.799645 -2.712257 -0.751464
H 3.125534 2.159793 -2.051842
H 2.458847 4.657497 -2.148715
H 1.148676 5.199866 -1.080402
H 2.638748 4.536529 -0.394157
H -0.565691 3.668111 -0.392200
H -4.771920 -1.473371 -0.082272
H -3.065751 2.458336 0.307728
H -5.559215 2.054131 0.439447
H -6.192241 0.393161 0.463984
H -5.344197 1.044125 1.873214
H -1.793814 -2.964053 -1.779611
H -3.559436 -3.018764 -1.674289
H -2.579932 -3.506194 -0.289759
H 3.406137 -0.488105 -2.094250
H 2.048894 -0.855928 -3.160589
H -0.771417 -2.618627 0.774788
H 0.840047 -4.358016 1.500479
H 3.130054 -4.415406 0.520776
H 3.813550 -2.699171 -1.134730
B 1.331357 0.649182 2.502461
F 0.178075 -0.022399 2.036441
F 2.420690 0.378801 1.647239
F 1.084806 2.040763 2.532280
F 1.636449 0.201340 3.809719
47
10-BF4-GS_13 Eopt
-1349.645485
O -0.253562 -0.513680 -1.267672
C 0.236102 0.851293 -1.181052
C 1.483858 1.175225 -1.645117
C 1.821550 2.520676 -1.456903
C 0.932032 3.427477 -0.839013
C 1.361170 4.860232 -0.639292
C -0.328198 2.999678 -0.386485
C -0.689725 1.658706 -0.553736
C -1.856538 0.831452 -0.252810
C -1.615340 -0.471585 -0.707181
C -2.547573 -1.484807 -0.806821
C -3.794090 -1.150407 -0.238245
C -4.081595 0.104714 0.318484
C -3.109199 1.117976 0.280499
C -5.430134 0.371720 0.937003
C -2.334159 -2.807204 -1.493670
C 2.313312 0.060677 -2.232299
C 2.050328 -1.215485 -1.454082
C 0.799569 -1.496650 -0.909036
C 0.510016 -2.540983 -0.057114
C 1.547106 -3.435647 0.221259
C 2.812645 -3.239069 -0.333838
C 3.062697 -2.131753 -1.146875
H 2.796567 2.870658 -1.783840
H 2.129223 4.926650 0.140436
H 1.789808 5.276850 -1.556666
H 0.518915 5.487128 -0.335437
H -1.002886 3.699057 0.095548
H -4.575211 -1.905232 -0.264335
H -3.334018 2.115880 0.642364
H -6.190167 -0.309445 0.544525
H -5.385780 0.230675 2.023990
H -5.753871 1.400625 0.753488
H -2.155367 -3.609808 -0.770755
H -1.489688 -2.780560 -2.185345
H -3.232484 -3.068405 -2.059615
H 3.377107 0.302434 -2.197442
H 2.042232 -0.079406 -3.287868
H -0.460415 -2.650314 0.404463
H 1.359435 -4.267917 0.890842
H 3.617132 -3.931008 -0.107260
H 4.060675 -1.950848 -1.534036
B 1.343207 0.055782 2.636098
F 1.219203 1.083374 3.601399
F 0.088960 -0.165005 2.025620
F 2.287935 0.441359 1.660773
F 1.774820 -1.133495 3.268406
47
10-BF4-GS_16 Eopt
-1349.645767
O 0.566560 -0.312099 -1.248351
C 1.096348 0.968292 -0.816219
C 2.449024 1.175817 -0.744856
C 2.798401 2.448898 -0.279361
C 1.817425 3.400605 0.079538
C 2.256107 4.751561 0.591362
C 0.449530 3.092327 -0.017199
C 0.070608 1.825273 -0.474148
C -1.186136 1.122222 -0.722666
C -0.898637 -0.153553 -1.221970
C -1.802933 -1.030635 -1.785545
C -3.139537 -0.599805 -1.668338
C -3.513225 0.624018 -1.092302
C -2.519986 1.510615 -0.645392
C -4.969240 0.987961 -0.957417
C -1.454012 -2.306041 -2.503336
C 3.345468 0.024698 -1.129628
C 2.705726 -1.274498 -0.675141
C 1.323260 -1.445664 -0.665045
C 0.648512 -2.517721 -0.119610
C 1.428650 -3.561237 0.387080
C 2.822214 -3.480010 0.352715
C 3.449643 -2.339844 -0.154086
H 3.849420 2.706213 -0.183145
H 2.808290 4.648340 1.533329
H 2.921224 5.246614 -0.125648
H 1.396395 5.403867 0.770920
H -0.298258 3.822759 0.272118
H -3.912259 -1.246259 -2.073674
H -2.783643 2.488067 -0.256451
H -5.119302 2.069729 -1.056506
H -5.579575 0.476396 -1.710447
H -5.342766 0.690508 0.034346

H	-1.620383	-3.181533	-1.866788
H	-0.413657	-2.319720	-2.832672
H	-2.094209	-2.412488	-3.382913
H	4.333957	0.128163	-0.679256
H	3.483112	0.016692	-2.219601
H	-0.428770	-2.532264	-0.041032
H	0.935092	-4.422019	0.825955
H	3.422411	-4.291875	0.750213
H	4.531484	-2.253475	-0.127536
B	-1.162948	-0.532772	2.630329
F	-0.914131	-1.069865	3.915466
F	0.063376	-0.183673	2.022024
F	-1.827951	-1.500562	1.843057
F	-1.973468	0.618907	2.749825

47

10-BF4-GS_17 Eopt

-1349.644987

O	0.239247	-1.000463	-1.088682
C	0.657465	0.378369	-1.279605
C	1.963456	0.680297	-1.559462
C	2.209417	2.054350	-1.681667
C	1.180761	3.007318	-1.518865
C	1.507335	4.477079	-1.615719
C	-0.132439	2.600675	-1.222350
C	-0.405374	1.236867	-1.086678
C	-1.581792	0.418196	-0.802230
C	-1.211760	-0.932697	-0.831149
C	-2.073483	-2.011916	-0.834077
C	-3.419888	-1.647137	-0.626857
C	-3.853894	-0.319750	-0.494080
C	-2.925424	0.726888	-0.616767
C	-5.307866	-0.014802	-0.239689
C	-1.686749	-3.445228	-1.084195
C	2.933570	-0.470514	-1.656302
C	2.552633	-1.526844	-0.635430
C	1.224450	-1.768287	-0.290051
C	0.797619	-2.591872	0.729972
C	1.781243	-3.302016	1.424475
C	3.128132	-3.143629	1.093329
C	3.506950	-2.251941	0.087661
H	3.219270	2.391961	-1.896194
H	0.622544	5.063358	-1.878419
H	1.877812	4.849042	-0.652545
H	2.284303	4.664447	-2.362816
H	-0.915731	3.338087	-1.083458
H	-4.155716	-2.446083	-0.598099
H	-3.248266	1.762077	-0.583291
H	-5.947372	-0.853778	-0.527057
H	-5.474235	0.183413	0.826246
H	-5.628440	0.874484	-0.790883
H	-0.724757	-3.529698	-1.593807
H	-2.448114	-3.919402	-1.709364
H	-1.627085	-4.013560	-0.150221
H	3.958354	-0.138407	-1.480285
H	2.896038	-0.895792	-2.668719
H	-0.243577	-2.665635	1.008268
H	1.483876	-3.959830	2.233944
H	3.888984	-3.691846	1.639255
H	4.557350	-2.090829	-0.134842
B	-0.033010	1.467526	2.669507
F	-1.430040	1.592771	2.492306
F	0.605850	2.651975	2.238415
F	0.248061	1.253699	4.039980
F	0.443870	0.370846	1.918480

47

10-BF4-GS_18 Eopt

-1349.644419

O	-1.283651	0.584220	0.678406
C	-0.443858	-0.600190	0.569551
C	0.883187	-0.545469	0.906170
C	1.562702	-1.752964	0.710408
C	0.906880	-2.898282	0.204781
C	1.692330	-4.172927	0.024726
C	-0.457133	-2.852338	-0.134516
C	-1.160407	-1.655323	0.042001
C	-2.533182	-1.190906	-0.162496
C	-2.623534	0.144794	0.251231
C	-3.787375	0.864966	0.432687
C	-4.939371	0.179404	-0.004867
C	-4.916365	-1.128968	-0.514425
C	-3.701510	-1.831517	-0.564373
C	-6.189707	-1.784278	-0.985978
C	-3.881608	2.220728	1.078076
C	1.422013	0.785325	1.369612
C	0.748915	1.885658	0.571454
C	-0.579406	1.787570	0.162440
C	-1.228920	2.659349	-0.686788
C	-0.504930	3.775950	-1.119650
C	0.817702	3.957009	-0.709722
C	1.439367	3.014040	0.111849
H	2.623079	-1.800726	0.935914
H	2.583469	-3.996503	-0.586732
H	2.034584	-4.559503	0.991966
H	1.089108	-4.946294	-0.457539
H	-0.950195	-3.731427	-0.535855
H	-5.893417	0.688869	0.092865
H	-3.674612	-2.868204	-0.883655
H	-6.202126	-2.850089	-0.735271
H	-7.069678	-1.309769	-0.541696
H	-6.280295	-1.702292	-2.077208
H	-4.755963	2.248640	1.733304
H	-4.003782	3.012307	0.331209
H	-2.997299	2.453441	1.676533
H	2.502507	0.834372	1.238082
H	1.208857	0.913511	2.439744
H	-2.242216	2.489498	-1.021833
H	-0.981222	4.488364	-1.784452
H	1.377020	4.821055	-1.052714
H	2.483190	3.128220	0.386112
B	4.982416	-0.174486	-0.365282
F	4.737134	-0.172284	1.029400
F	4.014600	0.624860	-1.012927
F	6.270548	0.348098	-0.615900
F	4.905651	-1.498963	-0.851979

47

10-BF4-GS_19 Eopt

-1349.644428

O	1.260516	-0.571243	0.488524
C	0.446167	0.596197	0.183121
C	-0.921254	0.524745	0.244450
C	-1.563228	1.717421	-0.110919
C	-0.829852	2.863761	-0.493700
C	-1.576261	4.127069	-0.844999
C	0.574925	2.835036	-0.548518
C	1.242945	1.652877	-0.207258
C	2.633805	1.207851	-0.114304
C	2.653154	-0.118598	0.337859
C	3.762765	-0.815181	0.775229
C	4.971328	-0.117838	0.573289
C	5.039172	1.179132	0.041396
C	3.852568	1.860993	-0.276770
C	6.375147	1.846220	-0.164694
C	3.735094	-2.157402	1.455946
C	-1.524774	-0.804829	0.622657
C	-0.690268	-1.911575	0.006346
C	0.691900	-1.796235	-0.131111
C	1.510560	-2.672484	-0.813170
C	0.904555	-3.813893	-1.348997
C	-0.469190	-4.014808	-1.202400
C	-1.257396	-3.065056	-0.548071
H	-2.648403	1.754251	-0.101725
H	-0.906408	4.878807	-1.270505
H	-2.373739	3.923520	-1.567056
H	-2.049171	4.557475	0.045490
H	1.128921	3.715539	-0.856232
H	5.890656	-0.608837	0.880225
H	3.879927	2.891176	-0.616225
H	7.138017	1.419806	0.492350
H	6.714441	1.712437	-1.199356
H	6.313525	2.922218	0.023288
H	2.756070	-2.378407	1.886933
H	4.475179	-2.169593	2.260466
H	3.989164	-2.964877	0.761387
H	-2.557531	-0.881986	0.283083
H	-1.531310	-0.904052	1.716760
H	2.567019	-2.487505	-0.946681
H	1.514530	-4.531729	-1.886819
H	-0.934933	-4.900384	-1.622160
H	-2.332338	-3.197823	-0.477031
B	-5.332319	0.173155	0.334923
F	-4.955822	1.367663	-0.323119
F	-4.813334	0.178542	1.649525
F	-6.738969	0.079423	0.378995
F	-4.806022	-0.935216	-0.372211

47

10-BF4-GS_20 Eopt

-1349.644633

O	1.265183	0.572346	-0.635824
C	0.472506	-0.638087	-0.478544
C	-0.869649	-0.630567	-0.753280
C	-1.499817	-1.857808	-0.513454
C	-0.780755	-2.974778	-0.030537
C	-1.514545	-4.271879	0.200928
C	0.594649	-2.881635	0.242715
C	1.248985	-1.663346	0.021710
C	2.613226	-1.152349	0.154385
C	2.637870	0.182969	-0.270441
C	3.768232	0.940248	-0.511214
C	4.961313	0.295106	-0.129250
C	5.008252	-1.008466	0.386482
C	3.821281	-1.751443	0.501925
C	6.325715	-1.618374	0.795540
C	3.785494	2.297757	-1.161870
C	-1.472214	0.676129	-1.205771
C	-0.804096	1.806618	-0.445989
C	0.543977	1.755360	-0.095962
C	1.200419	2.652873	0.720527
C	0.460836	3.749648	1.174946
C	-0.882996	3.884838	0.822254
C	-1.509670	2.915157	0.035389
H	-2.568552	-1.941697	-0.686331
H	-0.866132	-5.017704	0.667716
H	-2.384894	-4.117927	0.848236
H	-1.884169	-4.684682	-0.745022
H	1.135999	-3.739743	0.626530
H	5.892358	0.835607	-0.276748
H	3.844908	-2.785815	0.827497

H 6.337721 -2.695846 0.607915
H 7.159865 -1.160008 0.257289
H 6.499386 -1.468203 1.868297
H 3.914156 3.094860 -0.422018
H 2.868059 2.498897 -1.719334
H 4.627921 2.354185 -1.856250
H -2.548771 0.698729 -1.035213
H -1.304704 0.800133 -2.285261
H 2.232461 2.516455 1.011040
H 0.941579 4.481657 1.814133
H -1.455103 4.733409 1.182146
H -2.567493 2.993153 -0.193557
B -5.091054 -0.152419 0.193706
F -6.427647 -0.178822 0.646124
F -4.857604 1.046677 -0.523472
F -4.212874 -0.215962 1.298954
F -4.856860 -1.258555 -0.658933
47
10-BF4-GS_21 Eopt
-1349.644645
O -1.266146 -0.573204 -0.639191
C -0.470033 0.635994 -0.484861
C 0.872083 0.624954 -0.763638
C 1.505605 1.850773 -0.527176
C 0.790832 2.969683 -0.042167
C 1.528220 4.266000 0.185768
C -0.584581 2.879921 0.234731
C -1.242318 1.663323 0.016772
C -2.607149 1.155188 0.155284
C -2.636525 -0.180261 -0.268915
C -3.768455 -0.935460 -0.505934
C -4.959595 -0.287570 -0.119677
C -5.001511 1.016934 0.396148
C -3.812446 1.757203 0.505980
C -6.315818 1.628779 0.809486
C -3.791199 -2.292411 -1.156630
C 1.469751 -0.683521 -1.217137
C 0.800610 -1.812172 -0.455322
C -0.546062 -1.757150 -0.100472
C -1.202038 -2.652988 0.718102
C -0.462966 -3.751050 1.170948
C 0.879085 -3.890304 0.812728
C 1.505593 -2.922296 0.024214
H 2.573633 1.932297 -0.702994
H 2.400378 4.109896 0.829476
H 1.895070 4.676821 -0.761902
H 0.882498 5.013406 0.653764
H -1.122617 3.739631 0.619838
H -5.892363 -0.825838 -0.263376
H -3.832508 2.791948 0.831774
H -6.484733 1.482685 1.883604
H -6.328046 2.705947 0.619094
H -7.153807 1.170265 0.277376
H -3.919085 -3.088932 -0.416188
H -2.876261 -2.495390 -1.716880
H -4.636277 -2.347228 -1.848083
H 2.546345 -0.708594 -1.048236
H 1.299960 -0.807446 -2.295634
H -2.232642 -2.513562 1.012749
H -0.943436 -4.482302 1.812052
H 1.450051 -4.740597 1.171003
H 2.562185 -3.004063 -0.209225
B 5.080717 0.153759 0.206138
F 4.180752 0.240298 1.291551
F 4.844331 -1.046820 -0.506137
F 4.881997 1.255309 -0.659682
F 6.407447 0.163043 0.685960
47
10-BF4-GS_22 Eopt
-1349.643938
O -1.135548 0.584399 0.315240
C -0.195685 -0.349990 -0.287065
C 1.106893 0.016421 -0.505772
C 1.876518 -0.983079 -1.112053
C 1.323236 -2.237649 -1.454194
C 2.211711 -3.283007 -2.081511
C -0.033769 -2.514125 -1.212418
C -0.830245 -1.530484 -0.614218
C -2.222927 -1.411680 -0.183121
C -2.406942 -0.154259 0.408152
C -3.505498 0.248036 1.142075
C -4.561522 -0.686103 1.118404
C -4.484428 -1.928725 0.471049
C -3.287860 -2.307722 -0.160566
C -5.667480 -2.863078 0.473761
C -3.597941 1.513903 1.951238
C 1.504024 1.416335 -0.110168
C 0.346456 2.352966 -0.399929
C -0.978865 1.945091 -0.255566
C -2.090368 2.668816 -0.635004
C -1.865764 3.952949 -1.142152
C -0.563638 4.440663 -1.266540
C 0.527266 3.641717 -0.915751
H 2.924260 -0.788188 -1.315083
H 2.722923 -2.884967 -2.964595
H 2.987020 -3.602954 -1.376185
H 1.638436 -4.163846 -2.381984
H -0.454287 -3.475758 -1.486704
H -5.466665 -0.432539 1.663402
H -3.181334 -3.294802 -0.598376
H -6.240001 -2.758441 -0.456186
H -5.346718 -3.906643 0.544864
H -6.343210 -2.648001 1.305927
H -4.172523 2.284630 1.426777
H -2.613299 1.925249 2.183692
H -4.112032 1.303355 2.892687
H 2.395123 1.741678 -0.647849
H 1.749272 1.433526 0.959609
H -3.090429 2.266625 -0.558392
H -2.714613 4.556897 -1.444298
H -0.393991 5.436998 -1.661446
H 1.540657 4.005009 -1.055409
B 4.917993 -0.326774 1.051494
F 4.868900 0.513295 -0.086372
F 6.112633 -0.081047 1.764825
F 3.807899 -0.056895 1.884363
F 4.882435 -1.677701 0.639214
47
10-BF4-GS_23 Eopt
-1349.644177
O 1.147054 -0.487219 0.469987
C 0.734495 0.884949 0.221679
C -0.588090 1.238205 0.299729
C -0.827044 2.586921 0.006819
C 0.224531 3.467092 -0.336721
C -0.092189 4.913149 -0.624692
C 1.551178 3.005714 -0.412115
C 1.818522 1.660793 -0.131890
C 3.003393 0.805399 -0.074422
C 2.612444 -0.480749 0.321468
C 3.452745 -1.503602 0.715334
C 4.818472 -1.203896 0.527752
C 5.283438 0.031078 0.049981
C 4.364899 1.058506 -0.223783
C 6.759625 0.263933 -0.146699
C 3.015682 -2.801288 1.339729
C -1.574916 0.148444 0.631465
C -1.125402 -1.137223 -0.036144
C 0.224079 -1.452614 -0.184865
C 0.730632 -2.516892 -0.901786
C -0.201081 -3.392012 -1.469900
C -1.568858 -3.159147 -1.318862
C -2.024801 -2.034870 -0.624907
H -1.846282 2.959710 0.037276
H 0.791790 5.449410 -0.978919
H -0.875994 4.998416 -1.385929
H -0.459179 5.417601 0.276014
H 2.351214 3.684836 -0.687166
H 5.540804 -1.966888 0.802208
H 4.708513 2.043966 -0.520090
H 7.031213 1.298508 0.082585
H 7.354283 -0.401283 0.485519
H 7.041243 0.072125 -1.189313
H 3.047923 -3.623426 0.616927
H 2.002920 -2.745037 1.744111
H 3.696950 -3.055734 2.156016
H -2.580850 0.411252 0.303816
H -1.614583 0.010555 1.720816
H 1.791991 -2.667599 -1.037218
H 0.155839 -4.245966 -2.036777
H -2.287740 -3.839168 -1.764023
H -3.087531 -1.827005 -0.551303
B -5.502953 -0.009307 0.303603
F -5.006723 -0.422255 1.561095
F -5.325931 -1.051577 -0.636637
F -6.874411 0.302520 0.412614
F -4.790126 1.135611 -0.127682
47
10-BF4-GS_24 Eopt
-1349.644151
O -1.884480 0.243170 -0.636050
C -1.061200 -0.957670 -0.673712
C -1.641253 -2.194360 -0.777083
C -0.719359 -3.249728 -0.760251
C 0.668378 -3.016950 -0.641133
C 1.623345 -4.184866 -0.662560
C 1.170759 -1.708493 -0.512793
C 0.272698 -0.636509 -0.518565
C 0.363226 0.821187 -0.424889
C -0.925598 1.361581 -0.531401
C -1.240801 2.692392 -0.715997
C -0.119433 3.543986 -0.625970
C 1.190983 3.082663 -0.432659
C 1.439292 1.700481 -0.361479
C 2.337817 4.055517 -0.330269
C -2.610122 3.229385 -1.035288
C -3.148374 -2.244710 -0.833217
C -3.706945 -1.169599 0.081452
C -3.060704 0.054923 0.246032
C -3.400884 1.025699 1.165365
C -4.536846 0.787517 1.946110
C -5.258322 -0.397584 1.793453
C -4.835446 -1.370199 0.884088
H -1.084686 -4.270215 -0.830332
H 1.179073 -5.068733 -0.195306
H 1.881345 -4.451717 -1.694961
H 2.553521 -3.943726 -0.141734
H 2.236050 -1.540801 -0.399468

H -0.289681 4.610588 -0.744017
H 2.453310 1.324241 -0.280707
H 2.694427 4.119331 0.704696
H 3.184694 3.730557 -0.942537
H 2.039979 5.058126 -0.648287
H -2.521248 4.027281 -1.777431
H -3.091294 3.656196 -0.149154
H -3.269603 2.458394 -1.439505
H -3.523961 -3.223413 -0.528862
H -3.482873 -2.070654 -1.865164
H -2.812508 1.923114 1.295607
H -4.840241 1.529808 2.676375
H -6.137757 -0.579126 2.402543
H -5.369888 -2.311413 0.801095
B 4.794354 -0.348619 0.763019
F 4.700759 0.843990 0.006391
F 6.075236 -0.437645 1.347245
F 3.807951 -0.340016 1.774968
F 4.582515 -1.458339 -0.089153
47
10-BF4-GS_25 Eopt
-1349.644165
O -1.883823 0.230652 -0.638484
C -1.032637 -0.949941 -0.694892
C -1.584363 -2.198605 -0.801593
C -0.637738 -3.232683 -0.802409
C 0.744731 -2.968267 -0.694876
C 1.729974 -4.110296 -0.734393
C 1.217572 -1.648726 -0.560484
C 0.295175 -0.598353 -0.549848
C 0.351636 0.860481 -0.445453
C -0.950657 1.370379 -0.535389
C -1.299924 2.694328 -0.708581
C -0.198986 3.572300 -0.623240
C 1.123998 3.141430 -0.444167
C 1.406783 1.765430 -0.386344
C 2.246341 4.142152 -0.339387
C -2.685289 3.198722 -1.012248
C -3.090270 -2.283963 -0.840766
C -3.662598 -1.230719 0.090852
C -3.044016 0.007842 0.256750
C -3.396466 0.963825 1.186995
C -4.516314 0.692349 1.980070
C -5.209977 -0.509349 1.828349
C -4.775075 -1.464998 0.906770
H -0.980096 -4.260701 -0.876477
H 1.272211 -5.041943 -0.390263
H 2.087585 -4.273763 -1.758585
H 2.604082 -3.898392 -0.112866
H 2.279378 -1.458179 -0.451721
H -0.396024 4.635226 -0.733368
H 2.430412 1.412694 -0.320620
H 3.119793 3.815778 -0.911941
H 1.937996 5.127164 -0.699753
H 2.565947 4.249249 0.704294
H -3.169098 3.608126 -0.119268
H -3.328943 2.413673 -1.415211
H -2.623116 4.002838 -1.750386
H -3.439231 -3.274288 -0.542267
H -3.441055 -2.107671 -1.866917
H -2.829218 1.874914 1.316347
H -4.828880 1.422032 2.719117
H -6.076804 -0.716757 2.447182
H -5.287860 -2.418058 0.823663
B 4.757864 -0.347694 0.797866
F 6.034342 -0.307459 1.397285
F 4.616353 0.746204 -0.089884
F 4.610429 -1.554760 0.075033
F 3.760297 -0.272731 1.795709
47
10-BF4-GS_26 Eopt
-1349.644167
O -1.884157 0.234253 -0.637622
C -1.042241 -0.953157 -0.685021
C -1.603353 -2.197995 -0.790910
C -0.665099 -3.239330 -0.783049
C 0.719082 -2.985524 -0.668948
C 1.693904 -4.136683 -0.700538
C 1.201648 -1.669620 -0.536246
C 0.287363 -0.611820 -0.533714
C 0.355003 0.846799 -0.433481
C -0.942532 1.367046 -0.532561
C -1.279954 2.693272 -0.711396
C -0.172216 3.562459 -0.622474
C 1.146154 3.121299 -0.434915
C 1.417088 1.743143 -0.370925
C 2.276667 4.112565 -0.327928
C -2.659302 3.208567 -1.023824
C -3.109725 -2.271408 -0.838935
C -3.679379 -1.210111 0.085134
C -3.051686 0.023857 0.250958
C -3.401912 0.985615 1.176044
C -4.528950 0.725953 1.962870
C -5.231796 -0.470284 1.810313
C -4.799053 -1.432396 0.894451
H -1.015111 -4.264900 -0.855830
H 1.242257 -5.050218 -0.303320
H 2.010167 -4.343937 -1.730434
H 2.592251 -3.908954 -0.120863
H 2.264560 -1.487236 -0.423849
H -0.359853 4.626646 -0.736554
H 2.437169 1.381969 -0.297450
H 3.143913 3.784674 -0.909072
H 1.973643 5.102910 -0.678028
H 2.604008 4.207697 0.714459
H -2.586268 4.011055 -1.762731
H -3.144732 3.623316 -0.134228
H -3.307172 2.428364 -1.429353
H -3.468495 -3.257676 -0.538627
H -3.452753 -2.096285 -1.867906
H -2.827797 1.892273 1.306138
H -4.840001 1.460503 2.697722
H -6.104244 -0.668496 2.424236
H -5.319125 -2.381503 0.810923
B 4.774315 -0.346986 0.779145
F 4.603373 -1.553354 0.060335
F 6.057110 -0.322729 1.365780
F 4.638416 0.747013 -0.109407
F 3.787880 -0.256794 1.786692
47
10-BF4-GS_27 Eopt
-1349.644157
O -1.886432 0.251120 -0.629417
C -1.080929 -0.961817 -0.652452
C -1.677606 -2.191151 -0.758340
C -0.771662 -3.258990 -0.729188
C 0.618789 -3.045787 -0.597421
C 1.552642 -4.230906 -0.599591
C 1.138670 -1.745031 -0.470108
C 0.255604 -0.659849 -0.487727
C 0.367889 0.796507 -0.401752
C -0.911781 1.356339 -0.520293
C -1.204038 2.691294 -0.712839
C -0.069624 3.525123 -0.618801
C 1.232135 3.044158 -0.416394
C 1.457657 1.658558 -0.336522
C 2.394252 3.998800 -0.315401
C -2.561258 3.250642 -1.045026
C -3.184385 -2.220145 -0.833294
C -3.739541 -1.132235 0.067993
C -3.078038 0.083766 0.235935
C -3.418465 1.063955 1.145096
C -4.568908 0.846058 1.910340
C -5.304747 -0.329582 1.753293
C -4.882450 -1.312590 0.854968
H -1.150516 -4.274588 -0.800875
H 1.667286 -4.630310 -1.614641
H 2.544392 -3.952343 -0.235446
H 1.165627 -5.039959 0.028150
H 2.205456 -1.591064 -0.350794
H -0.222203 4.593665 -0.742573
H 2.465441 1.267259 -0.250321
H 3.231275 3.665537 -0.936745
H 2.109124 5.007923 -0.624234
H 2.760086 4.049431 0.716983
H -2.453236 4.039817 -1.793928
H -3.038983 3.694749 -0.165564
H -3.232768 2.488538 -1.445898
H -3.577648 -3.191676 -0.528492
H -3.503121 -2.047256 -1.870416
H -2.820315 1.954201 1.278675
H -4.872440 1.596651 2.632010
H -6.195450 -0.495526 2.350339
H -5.428623 -2.246809 0.768827
B 4.833137 -0.348057 0.729125
F 3.917019 -0.287177 1.803487
F 4.514063 -1.451664 -0.097729
F 4.745663 0.842959 -0.030096
F 6.144048 -0.495187 1.228763
47
10-BF4-GS_28 Eopt
-1349.644219
O -1.880222 0.264109 -0.629630
C -1.105295 -0.968748 -0.632947
C -1.730689 -2.184071 -0.736985
C -0.852088 -3.273745 -0.689066
C 0.541852 -3.093837 -0.543211
C 1.445781 -4.301731 -0.523333
C 1.092005 -1.805603 -0.420296
C 0.236178 -0.699139 -0.455239
C 0.384724 0.754236 -0.379316
C -0.878980 1.345756 -0.518081
C -1.134101 2.686462 -0.721216
C 0.020646 3.491371 -0.616914
C 1.306561 2.978932 -0.394265
C 1.494857 1.588319 -0.302885
C 2.494155 3.901768 -0.293704
C -2.471844 3.281236 -1.071047
C -3.236563 -2.176369 -0.830184
C -3.775507 -1.067962 0.055723
C -3.086879 0.133287 0.221459
C -3.415637 1.129524 1.117320
C -4.580191 0.945923 1.870133
C -5.341355 -0.213712 1.715004
C -4.931637 -1.214418 0.830539
H -1.254883 -4.280327 -0.758037
H 1.521918 -4.744769 -1.523621
H 2.453613 -4.034400 -0.196679

H 1.056842 -5.074808 0.147451
H 2.160972 -1.673488 -0.295618
H -0.103064 4.562656 -0.748485
H 2.490416 1.172592 -0.196839
H 2.967953 3.814821 0.690356
H 3.254098 3.642648 -1.038566
H 2.202156 4.944204 -0.443999
H -2.335387 4.054247 -1.832121
H -2.939888 3.754695 -0.201612
H -3.164608 2.533879 -1.463238
H -3.657199 -3.135555 -0.523197
H -3.538372 -2.003724 -1.872400
H -2.799248 2.007199 1.249964
H -4.874682 1.710450 2.580844
H -6.242758 -0.353176 2.302692
H -5.498288 -2.136563 0.746150
B 4.843781 -0.348808 0.693210
F 6.133148 -0.734391 1.116703
F 4.425926 -1.183742 -0.371276
F 3.933337 -0.470410 1.767329
F 4.870626 0.993621 0.249357
47
10-BF4-GS_30 Eopt
-1349.644164
O -1.886662 0.229177 -0.637638
C -1.035656 -0.951766 -0.685577
C -1.586883 -2.200386 -0.794059
C -0.640495 -3.234870 -0.785651
C 0.741066 -2.970803 -0.668408
C 1.727043 -4.112481 -0.698295
C 1.213405 -1.650934 -0.533037
C 0.291319 -0.600493 -0.530919
C 0.347346 0.858454 -0.427968
C -0.953712 1.368914 -0.528845
C -1.300709 2.692927 -0.707386
C -0.199786 3.570103 -0.615510
C 1.121590 3.138769 -0.424940
C 1.402685 1.762808 -0.361861
C 2.242896 4.139693 -0.311283
C -2.683332 3.197889 -1.022437
C -3.092525 -2.285292 -0.844560
C -3.671472 -1.230272 0.081062
C -3.053742 0.008437 0.249193
C -3.412506 0.966049 1.175305
C -4.538304 0.696452 1.960591
C -5.231304 -0.505205 1.805865
C -4.790031 -1.462630 0.889138
H -0.982719 -4.262857 -0.860489
H 2.119960 -4.254253 -1.712683
H 2.579723 -3.913931 -0.043257
H 1.258098 -5.050756 -0.389287
H 2.274689 -1.460592 -0.418415
H -0.395130 4.632954 -0.729555
H 2.424975 1.408484 -0.286511
H 2.530183 4.273286 0.738842
H 3.132472 3.797312 -0.848218
H 1.948086 5.115942 -0.705348
H -2.614825 4.002143 -1.759828
H -3.174383 3.607219 -0.133418
H -3.323926 2.413095 -1.430809
H -3.444204 -3.275009 -0.547210
H -3.435341 -2.110349 -1.873633
H -2.845673 1.876957 1.307391
H -4.856019 1.427552 2.696037
H -6.102797 -0.711156 2.418609
H -5.302667 -2.415576 0.803806
B 4.780876 -0.344374 0.772587
F 4.626184 0.743088 -0.120790
F 6.064771 -0.298340 1.355855
F 3.795797 -0.264586 1.782354
F 4.626255 -1.556184 0.059208
47
10-BF4-GS_31 Eopt
-1349.644067
O 1.749979 0.207606 0.551920
C 0.937458 -0.975606 0.310378
C 1.448973 -2.226580 0.530207
C 0.548993 -3.260738 0.237051
C -0.752379 -2.994134 -0.241293
C -1.680115 -4.139974 -0.562301
C -1.184316 -1.671151 -0.451825
C -0.304287 -0.620153 -0.177285
C -0.352544 0.841407 -0.225672
C 0.866149 1.350562 0.243167
C 1.127828 2.664692 0.574630
C 0.075431 3.540606 0.235115
C -1.140323 3.115005 -0.320614
C -1.368365 1.743443 -0.526198
C -2.214454 4.114375 -0.666422
C 2.362512 3.159854 1.279345
C 2.878066 -2.309272 1.007527
C 3.685456 -1.216448 0.330209
C 3.125839 0.026682 0.035646
C 3.726428 1.021643 -0.707992
C 5.036937 0.787244 -1.137579
C 5.672131 -0.417305 -0.831188
C 4.995509 -1.412333 -0.121354
H 0.863182 -4.290356 0.381516
H -1.579942 -4.433237 -1.614770
H -1.453002 -5.017773 0.049212
H -2.723608 -3.859033 -0.396651
H -2.186122 -1.470882 -0.813732
H 0.212571 4.596019 0.454212
H -2.331198 1.382714 -0.870318
H -2.048284 5.069498 -0.161064
H -2.229156 4.303634 -1.746805
H -3.203724 3.738350 -0.388609
H 3.061560 3.632619 0.581337
H 2.891048 2.355625 1.795484
H 2.077522 3.912413 2.019390
H 3.314324 -3.285355 0.787486
H 2.907080 -2.174229 2.097442
H 3.211144 1.936030 -0.966529
H 5.545396 1.548865 -1.718868
H 6.688455 -0.594644 -1.167347
H 5.474773 -2.366369 0.075530
B -4.817178 -0.326616 0.107908
F -3.991472 0.135209 1.156635
F -4.233638 0.018171 -1.136933
F -4.944686 -1.731452 0.189876
F -6.091484 0.273340 0.208962
47
10-BF4-GS_32 Eopt
-1349.644171
O -1.890895 0.234916 -0.631121
C -1.055270 -0.957522 -0.662087
C -1.621403 -2.199987 -0.771648
C -0.688752 -3.245843 -0.747707
C 0.695363 -2.999024 -0.616454
C 1.663064 -4.156548 -0.631595
C 1.183095 -1.685352 -0.482676
C 0.274072 -0.622842 -0.495762
C 0.348866 0.835620 -0.401172
C -0.944346 1.363143 -0.517639
C -1.270843 2.690892 -0.705753
C -0.158598 3.553256 -0.606797
C 1.154802 3.104853 -0.403098
C 1.416040 1.725238 -0.330460
C 2.290398 4.089551 -0.289854
C -2.642294 3.215071 -1.037320
C -3.127203 -2.266430 -0.841962
C -3.706340 -1.196880 0.066559
C -3.074576 0.034299 0.237981
C -3.435530 1.001694 1.153028
C -4.577632 0.751973 1.921061
C -5.284909 -0.440626 1.760854
C -4.841667 -1.408880 0.856629
H -1.043089 -4.269866 -0.821974
H 1.213886 -5.054647 -0.197614
H 1.956987 -4.398043 -1.660643
H 2.573850 -3.917098 -0.076806
H 2.245684 -1.507262 -0.359876
H -0.338394 4.618086 -0.727074
H 2.432952 1.357390 -0.246993
H 1.995363 5.081206 -0.643062
H 2.611901 4.183892 0.754391
H 3.159241 3.755885 -0.865322
H -2.554039 4.014148 -1.778249
H -3.135547 3.636951 -0.155479
H -3.290896 2.438242 -1.448003
H -3.495029 -3.249056 -0.540602
H -3.453734 -2.096564 -1.877159
H -2.858786 1.905620 1.289430
H -4.897130 1.491415 2.647343
H -6.169286 -0.631204 2.359950
H -5.365611 -2.355412 0.767661
B 4.830757 -0.341834 0.732185
F 4.587294 -1.551004 0.039360
F 6.152584 -0.336959 1.224375
F 4.651188 0.746912 -0.154811
F 3.920825 -0.224257 1.807005
47
10-BF4-GS_34 Eopt
-1349.644235
O -1.853356 0.251586 -0.608907
C -1.082408 -0.979187 -0.504445
C -1.692781 -2.196475 -0.660965
C -0.826846 -3.284100 -0.492952
C 0.540506 -3.099817 -0.186555
C 1.433199 -4.307326 -0.038421
C 1.073647 -1.809137 -0.020894
C 0.228886 -0.704060 -0.175631
C 0.367304 0.751130 -0.108681
C -0.872468 1.336698 -0.402163
C -1.103877 2.671516 -0.663163
C 0.031557 3.481907 -0.448348
C 1.284615 2.977642 -0.071684
C 1.461462 1.589972 0.073588
C 2.455524 3.904482 0.133839
C -2.395377 3.252503 -1.173672
C -3.177475 -2.188743 -0.930732
C -3.812818 -1.072215 -0.122068
C -3.146254 0.131440 0.104754
C -3.570800 1.138136 0.947058
C -4.811914 0.962434 1.568106
C -5.551842 -0.199595 1.343420
C -5.047613 -1.210638 0.521556
H -1.219378 -4.292081 -0.593685

H 1.587861 -4.796093 -1.007663
H 2.412408 -4.027596 0.357320
H 0.986260 -5.048174 0.632989
H 2.119568 -1.673874 0.230924
H -0.076311 4.549625 -0.618333
H 2.440157 1.178699 0.293130
H 2.187340 4.940507 -0.088266
H 2.810496 3.855004 1.169362
H 3.296163 3.617614 -0.506609
H -2.176594 4.003594 -1.937456
H -2.954614 3.749834 -0.373999
H -3.042298 2.492710 -1.617337
H -3.633987 -3.145021 -0.669371
H -3.353415 -2.021823 -2.002335
H -2.971920 2.017398 1.137917
H -5.182944 1.734735 2.233214
H -6.512739 -0.332603 1.829593
H -5.602606 -2.133593 0.385403
B 4.954780 -0.330877 0.337003
F 4.795999 0.989160 0.819491
F 6.317720 -0.692223 0.382612
F 4.202808 -1.216648 1.145898
F 4.487268 -0.403239 -0.994693
47
10-BF4-GS_37 Eopt
-1349.644270
O -1.869224 0.222250 -0.644960
C -0.983959 -0.929230 -0.755891
C -1.498800 -2.194128 -0.862350
C -0.520797 -3.196372 -0.917322
C 0.856454 -2.888933 -0.860354
C 1.873258 -3.999268 -0.956799
C 1.290621 -1.557475 -0.723656
C 0.336197 -0.537833 -0.657174
C 0.351893 0.919433 -0.528539
C -0.967849 1.389706 -0.556932
C -1.362731 2.705152 -0.693346
C -0.285863 3.614780 -0.637168
C 1.055872 3.222203 -0.518786
C 1.381302 1.854949 -0.495603
C 2.150599 4.255382 -0.441490
C -2.773688 3.171621 -0.932994
C -3.001516 -2.327202 -0.845587
C -3.569149 -1.310193 0.127755
C -2.985917 -0.054073 0.290597
C -3.332606 0.873605 1.250992
C -4.409784 0.551020 2.083166
C -5.067709 -0.671464 1.938809
C -4.638624 -1.596294 0.983688
H -0.832794 -4.233909 -0.994376
H 2.809082 -3.720103 -0.465936
H 1.499221 -4.920125 -0.499678
H 2.101978 -4.221099 -2.006654
H 2.347165 -1.326900 -0.656706
H -0.519157 4.672727 -0.721022
H 2.414344 1.525450 -0.473967
H 2.503325 4.361065 0.591617
H 3.012472 3.962095 -1.048607
H 1.800599 5.234508 -0.779240
H -3.409992 2.372456 -1.319365
H -2.765997 3.986314 -1.662041
H -3.231469 3.555340 -0.015173
H -3.307135 -3.333686 -0.554399
H -3.397059 -2.142480 -1.853834
H -2.791643 1.801150 1.374771
H -4.716048 1.257363 2.847103
H -5.900671 -0.918672 2.588726
H -5.120651 -2.565686 0.904911
B 4.613213 -0.363968 0.933843
F 5.810670 0.202434 1.421490
F 4.754439 -1.766640 0.839137
F 3.549621 -0.053740 1.810653
F 4.330708 0.165027 -0.350365
47
10-BF4-GS_38 Eopt
-1349.644184
O -1.877993 0.225756 -0.616812
C -1.028707 -0.956987 -0.624319
C -1.575639 -2.204783 -0.753388
C -0.630165 -3.240438 -0.708906
C 0.745326 -2.977553 -0.540196
C 1.734288 -4.117009 -0.520204
C 1.213430 -1.656839 -0.391855
C 0.293223 -0.606377 -0.425767
C 0.348211 0.853107 -0.333335
C -0.947535 1.365742 -0.481178
C -1.283071 2.689536 -0.684640
C -0.182793 3.563918 -0.565143
C 1.130691 3.130615 -0.329626
C 1.404433 1.754737 -0.244335
C 2.251264 4.129349 -0.193382
C -2.651459 3.198194 -1.051391
C -3.077362 -2.290913 -0.869294
C -3.699248 -1.227403 0.017738
C -3.087838 0.010973 0.211125
C -3.492306 0.973421 1.113196
C -4.657863 0.710097 1.840376
C -5.345484 -0.490324 1.655217
C -4.859498 -1.452774 0.767019
H -0.970326 -4.267888 -0.798098
H 1.228096 -5.081729 -0.430754
H 2.327396 -4.129465 -1.442289
H 2.436202 -4.012768 0.312772
H 2.270628 -1.467467 -0.244212
H -0.370904 4.625966 -0.697168
H 2.422665 1.395935 -0.142728
H 1.988463 5.088732 -0.647327
H 2.475670 4.309033 0.865209
H 3.168237 3.758176 -0.660332
H -3.171928 3.613970 -0.182381
H -3.280477 2.414336 -1.478805
H -2.553284 3.998592 -1.789565
H -3.440509 -3.277770 -0.576148
H -3.374494 -2.128655 -1.914522
H -2.932395 1.884270 1.270887
H -5.011034 1.445393 2.555239
H -6.248187 -0.691235 2.222816
H -5.368908 -2.405307 0.659331
B 4.827906 -0.337458 0.654100
F 4.609893 -1.685672 0.286979
F 4.552043 0.497615 -0.455263
F 3.965781 0.003824 1.720955
F 6.169852 -0.164725 1.053530
47
10-BF4-GS_39 Eopt
-1349.644195
O -1.861484 0.221369 -0.609915
C -1.021534 -0.965930 -0.536349
C -1.565634 -2.211377 -0.693598
C -0.633647 -3.252586 -0.564056
C 0.725862 -2.995535 -0.290931
C 1.702608 -4.139586 -0.171340
C 1.190407 -1.675689 -0.121812
C 0.282953 -0.620380 -0.238015
C 0.339950 0.840158 -0.159743
C -0.936957 1.357309 -0.418450
C -1.248126 2.678809 -0.665158
C -0.155365 3.550256 -0.470628
C 1.131712 3.114336 -0.124014
C 1.389505 1.737987 0.004371
C 2.249004 4.105741 0.078041
C -2.581915 3.186820 -1.143221
C -3.054863 -2.286120 -0.924836
C -3.732070 -1.213268 -0.091258
C -3.128670 0.023926 0.132242
C -3.589127 0.997994 0.993977
C -4.803550 0.747048 1.641475
C -5.482399 -0.452678 1.422277
C -4.941681 -1.426667 0.579168
H -0.972223 -4.278910 -0.670282
H 2.229620 -4.104871 0.787923
H 1.196777 -5.105060 -0.253279
H 2.463550 -4.082963 -0.957728
H 2.235959 -1.491391 0.097843
H -0.326594 4.611108 -0.631477
H 2.392172 1.375905 0.202213
H 2.527189 4.160053 1.137223
H 3.144533 3.804548 -0.474864
H 1.957282 5.107524 -0.247797
H -3.159304 3.629699 -0.324785
H -3.185177 2.395588 -1.593541
H -2.424450 3.966285 -1.893472
H -3.448921 -3.268537 -0.658665
H -3.268625 -2.123785 -1.990242
H -3.037375 1.908567 1.180252
H -5.201490 1.491334 2.322796
H -6.422602 -0.643572 1.929261
H -5.447096 -2.378198 0.446556
B 4.901638 -0.325662 0.415297
F 4.539097 0.183535 -0.852818
F 6.286807 -0.152794 0.618503
F 4.184502 0.368604 1.418685
F 4.579780 -1.701498 0.478523
47
10-BF4-GS_40 Eopt
-1349.644204
O -1.867228 0.255504 -0.618232
C -1.098783 -0.978881 -0.537650
C -1.718413 -2.192744 -0.679765
C -0.853441 -3.285078 -0.533650
C 0.521231 -3.107509 -0.260402
C 1.417017 -4.316104 -0.145713
C 1.063946 -1.819008 -0.104419
C 0.221553 -0.710141 -0.238803
C 0.367351 0.744336 -0.170677
C -0.876619 1.335802 -0.432454
C -1.109667 2.672479 -0.681612
C 0.033360 3.478144 -0.490176
C 1.292951 2.967727 -0.144983
C 1.468250 1.578696 -0.010573
C 2.470906 3.890166 0.039002
C -2.411437 3.259507 -1.157864
C -3.209887 -2.178166 -0.909302
C -3.818139 -1.061009 -0.080720
C -3.139809 0.138686 0.131529
C -3.535617 1.144106 0.989241
C -4.759980 0.972126 1.643662
C -5.511663 -0.185391 1.435270

C -5.035251 -1.195994 0.596466
H -1.253355 -4.291018 -0.623975
H 2.337061 -4.075693 0.392976
H 0.911940 -5.136781 0.373027
H 1.699885 -4.680800 -1.140872
H 2.114897 -1.689577 0.129009
H -0.074749 4.547082 -0.652210
H 2.449932 1.163630 0.186498
H 3.295833 3.605591 -0.622563
H 2.200093 4.928129 -0.170435
H 2.849613 3.833416 1.065600
H -3.071918 2.502906 -1.586748
H -2.209558 4.012036 -1.924871
H -2.948397 3.756330 -0.342773
H -3.663133 -3.133073 -0.637551
H -3.414201 -2.008164 -1.975380
H -2.927518 2.019852 1.166457
H -5.108712 1.743728 2.321510
H -6.459445 -0.315476 1.947283
H -5.598017 -2.116008 0.472893
B 4.958038 -0.332687 0.414878
F 4.598600 -0.552603 -0.934394
F 4.820480 1.041660 0.719419
F 4.102530 -1.085022 1.254807
F 6.295082 -0.732613 0.621852
47
10-BF4-GS_41 Eopt
-1349.644179
O -1.879354 0.228710 -0.619631
C -1.035505 -0.957860 -0.628256
C -1.589711 -2.204282 -0.752037
C -0.650538 -3.243574 -0.706627
C 0.728275 -2.987277 -0.543284
C 1.704572 -4.137578 -0.537693
C 1.203328 -1.670098 -0.398461
C 0.287634 -0.613779 -0.433079
C 0.350285 0.845421 -0.341746
C -0.943276 1.364221 -0.487354
C -1.273128 2.689421 -0.690645
C -0.168568 3.558705 -0.573688
C 1.143309 3.119304 -0.340501
C 1.410859 1.742193 -0.255239
C 2.268757 4.112862 -0.206505
C -2.639876 3.204139 -1.054990
C -3.092341 -2.282551 -0.862085
C -3.705008 -1.214570 0.025885
C -3.086797 0.021157 0.214062
C -3.482592 0.987458 1.115844
C -4.646033 0.731244 1.848901
C -5.340149 -0.466292 1.669411
C -4.862831 -1.432836 0.780973
H -0.995929 -4.269926 -0.790154
H 2.036982 -4.363600 -1.558508
H 2.593298 -3.898264 0.051674
H 1.247129 -5.044215 -0.131101
H 2.261378 -1.485006 -0.252051
H -0.351967 4.621588 -0.705613
H 2.427502 1.378677 -0.155171
H 3.183122 3.737444 -0.675165
H 2.009486 5.073424 -0.659994
H 2.496012 4.291526 0.851650
H -2.539580 4.002435 -1.795166
H -3.156060 3.624426 -0.185598
H -3.273859 2.422506 -1.479129
H -3.459744 -3.267066 -0.566414
H -3.392406 -2.119798 -1.906393
H -2.917590 1.895974 1.268711
H -4.992509 1.469744 2.563725
H -6.241193 -0.661785 2.241508
H -5.377274 -2.383135 0.677593
B 4.832017 -0.338085 0.663330
F 3.961019 0.013592 1.719577
F 4.640354 -1.698576 0.329369
F 4.543911 0.464321 -0.466929
F 6.169598 -0.130092 1.061167
47
10-BF4-GS_42 Eopt
-1349.644183
O -1.864368 0.226647 -0.608871
C -1.031061 -0.966000 -0.551681
C -1.584897 -2.207951 -0.702626
C -0.656831 -3.254690 -0.590774
C 0.708635 -3.006243 -0.340040
C 1.680632 -4.156255 -0.240308
C 1.183664 -1.689520 -0.176230
C 0.280389 -0.628896 -0.275049
C 0.347480 0.830858 -0.194813
C -0.929867 1.356468 -0.429455
C -1.235985 2.680919 -0.670867
C -0.134460 3.544142 -0.496379
C 1.156604 3.099088 -0.173434
C 1.406811 1.721670 -0.050565
C 2.279474 4.086676 0.016025
C -2.573967 3.197343 -1.127863
C -3.077897 -2.274172 -0.910917
C -3.736804 -1.197373 -0.067704
C -3.122279 0.035374 0.150065
C -3.564782 1.010744 1.019703
C -4.772690 0.767161 1.681955
C -5.462917 -0.427130 1.468685
C -4.939586 -1.403283 0.617218
H -1.003233 -4.278773 -0.693300
H 1.165443 -5.118726 -0.294620
H 2.414762 -4.114294 -1.052823
H 2.240037 -4.115030 0.700048
H 2.233685 -1.511193 0.026702
H -0.301059 4.605972 -0.655921
H 2.410743 1.351818 0.123763
H 3.224069 3.685498 -0.362412
H 2.069791 5.031798 -0.492405
H 2.421526 4.303915 1.081887
H -2.423264 3.979892 -1.876241
H -3.138434 3.638358 -0.299514
H -3.186761 2.410951 -1.573888
H -3.473396 -3.254234 -0.638168
H -3.306929 -2.111297 -1.973072
H -3.004169 1.916899 1.200776
H -5.156775 1.512820 2.369684
H -6.398267 -0.612289 1.986646
H -5.453333 -2.350968 0.489164
B 4.887968 -0.329033 0.465733
F 6.265647 -0.172367 0.724969
F 4.576782 -1.705199 0.365780
F 4.137449 0.244970 1.518572
F 4.555973 0.315628 -0.748621
47
10-BF4-GS_44 Eopt
-1349.644238
O 1.866768 -0.255253 -0.616985
C 1.097972 0.978862 -0.534714
C 1.716635 2.192942 -0.678397
C 0.851372 3.284878 -0.530250
C -0.522374 3.106688 -0.253296
C -1.418941 4.314554 -0.136932
C -1.064011 1.817832 -0.095517
C -0.221532 0.709458 -0.232537
C -0.366452 -0.745189 -0.164436
C 0.877397 -1.335808 -0.428986
C 1.110178 -2.672328 -0.679560
C -0.032183 -3.478421 -0.486612
C -1.291483 -2.968851 -0.139275
C -1.467008 -1.579950 -0.003500
C -2.468745 -3.891827 0.046346
C 2.411204 -3.258761 -1.158566
C 3.207517 2.179137 -0.911737
C 3.818471 1.062284 -0.084734
C 3.141285 -0.137662 0.129603
C 3.539618 -1.142814 0.986446
C 4.765731 -0.970288 1.637448
C 5.516516 0.187373 1.426579
C 5.037497 1.197671 0.588889
H 1.250496 4.290991 -0.622086
H -0.909851 5.139443 0.371037
H -1.713234 4.672089 -1.131371
H -2.332819 4.076090 0.413053
H -2.114212 1.687520 0.140738
H 0.076179 -4.547182 -0.649589
H -2.448607 -1.165176 0.194740
H -2.841731 -3.839937 1.075398
H -3.297195 -3.603547 -0.609090
H -2.199482 -4.928899 -0.169479
H 2.949707 -3.756125 -0.344820
H 3.070800 -2.501684 -1.587964
H 2.208136 -4.010666 -1.925877
H 3.661033 3.134273 -0.641217
H 3.409131 2.009154 -1.978328
H 2.932137 -2.018647 1.165460
H 5.116604 -1.741571 2.314552
H 6.465732 0.317826 1.935832
H 5.599705 2.117786 0.463522
B -4.960694 0.332376 0.407760
F -6.299077 0.728865 0.612916
F -4.818767 -1.039942 0.718609
F -4.601572 0.547559 -0.942332
F -4.107826 1.091265 1.244697
47
10-BF4-GS_45 Eopt
-1349.644160
O 1.885670 -0.229994 -0.613036
C 1.050415 0.962384 -0.605350
C 1.611255 2.205663 -0.732883
C 0.679743 3.250853 -0.672334
C -0.698930 3.003067 -0.493330
C -1.666683 4.160517 -0.472803
C -1.180958 1.688832 -0.347379
C -0.272386 0.626725 -0.395466
C -0.344446 -0.832240 -0.309236
C 0.943526 -1.359611 -0.473506
C 1.260845 -2.686100 -0.687623
C 0.151616 -3.547921 -0.560229
C -1.153996 -3.100369 -0.308504
C -1.410340 -1.721608 -0.213162
C -2.284880 -4.086652 -0.166285
C 2.618571 -3.209003 -1.073163
C 3.112657 2.273861 -0.864711
C 3.731270 1.197772 0.009170
C 3.107348 -0.034377 0.202224
C 3.510772 -1.007127 1.093637

C 4.687705 -0.762172 1.808786
C 5.387325 0.431200 1.623059
C 4.902556 1.404800 0.746448
H 1.030772 4.275211 -0.757440
H -1.971899 4.423950 -1.493115
H -2.571098 3.909438 0.087063
H -1.212290 5.049390 -0.025157
H -2.239076 1.509375 -0.193768
H 0.325685 -4.611384 -0.699609
H -2.423891 -1.353313 -0.098716
H -2.031140 -5.051694 -0.613262
H -2.512549 -4.256258 0.893238
H -3.197601 -3.709677 -0.636827
H 3.142519 -3.639393 -0.213366
H 3.253054 -2.429382 -1.500195
H 2.502339 -4.001377 -1.817361
H 3.491617 3.254375 -0.570506
H 3.395778 2.113583 -1.914142
H 2.942241 -1.912442 1.251998
H 5.040380 -1.506096 2.514919
H 6.298858 0.617876 2.181331
H 5.421804 2.352020 0.638772
B -4.886345 0.333607 0.617553
F -4.669733 -0.762842 -0.251038
F -4.003106 0.240016 1.717112
F -4.639979 1.537903 -0.082281
F -6.220188 0.316785 1.076042
47
10-BF4-GS_46 Eopt
-1349.643071
O -1.273267 0.494619 -0.324237
C -2.273954 -0.553665 -0.423881
C -3.506449 -0.291616 -0.964784
C -4.367720 -1.395106 -0.963884
C -3.966877 -2.645802 -0.441509
C -4.934942 -3.802720 -0.479152
C -2.686300 -2.813989 0.113270
C -1.806173 -1.725519 0.134664
C -0.435117 -1.480179 0.581291
C -0.090941 -0.156997 0.278682
C 1.177382 0.388770 0.322299
C 2.134201 -0.480209 0.883507
C 1.845080 -1.791588 1.294884
C 0.551928 -2.307267 1.112721
C 2.930726 -2.644894 1.898936
C 1.568934 1.738814 -0.214519
C -3.765672 1.121093 -1.426707
C -3.088352 2.081055 -0.465878
C -1.874161 1.766651 0.143325
C -1.259085 2.501822 1.135490
C -1.875432 3.701127 1.508117
C -3.068906 4.096379 0.901828
C -3.674827 3.285228 -0.060794
H -5.369345 -1.284252 -1.369523
H -5.194816 -4.059965 -1.512235
H -4.510015 -4.690617 -0.004499
H -5.867491 -3.550424 0.037633
H -2.388964 -3.773830 0.521964
H 3.155409 -0.119043 0.955455
H 0.329479 -3.342372 1.349961
H 3.059670 -2.405496 2.961735
H 2.686838 -3.708127 1.823572
H 3.890414 -2.468538 1.405247
H 0.828035 2.134842 -0.912183
H 2.525019 1.651137 -0.735797
H 1.699780 2.466423 0.593427
H -4.835105 1.333678 -1.473955
H -3.358306 1.254118 -2.438309
H -0.350763 2.170623 1.618289
H -1.417406 4.311314 2.279017
H -3.543787 5.026780 1.195439
H -4.625906 3.571962 -0.498520
B 5.569158 0.242308 -0.791852
F 6.885110 0.562042 -1.191489
F 5.331140 0.761222 0.503710
F 4.647252 0.811512 -1.700975
F 5.406225 -1.160893 -0.772471
47
10-BF4-GS_47 Eopt
-1349.643269
O -1.284133 0.461340 -0.464528
C -2.477259 -0.365964 -0.411249
C -3.701167 0.153730 -0.745156
C -4.755899 -0.757277 -0.609517
C -4.537613 -2.080205 -0.163423
C -5.708006 -3.027101 -0.062030
C -3.245922 -2.517594 0.179375
C -2.173177 -1.626370 0.062190
C -0.729038 -1.670416 0.290382
C -0.178361 -0.430632 -0.056744
C 1.164823 -0.143272 -0.203733
C 2.003773 -1.201474 0.200074
C 1.524698 -2.444535 0.644809
C 0.142543 -2.691793 0.660695
C 2.496051 -3.512143 1.079222
C 1.727773 1.127831 -0.779676
C -3.742697 1.606996 -1.147935
C -2.746323 2.377609 -0.301234
C -1.540032 1.807628 0.103660
C -0.643370 2.367205 0.989983
C -0.940786 3.649022 1.464661
C -2.110002 4.294812 1.059385
C -3.008987 3.657119 0.200845
H -5.765989 -0.435007 -0.845742
H -6.039075 -3.341216 -1.059194
H -5.443481 -3.924970 0.502302
H -6.561385 -2.548252 0.428862
H -3.088502 -3.530169 0.535252
H 3.076101 -1.048129 0.129814
H -0.243211 -3.670022 0.928311
H 2.055129 -4.509117 0.992159
H 3.412820 -3.479526 0.484004
H 2.782584 -3.364030 2.127726
H 2.578910 0.887283 -1.420291
H 2.094702 1.795280 0.006548
H 0.988187 1.671878 -1.371160
H -4.742869 2.024742 -1.020167
H -3.481252 1.698833 -2.211073
H 0.243518 1.843127 1.317258
H -0.255344 4.126717 2.156245
H -2.336350 5.288711 1.431240
H -3.939464 4.142703 -0.076697
B 5.709463 0.275542 -0.343128
F 7.101484 0.499778 -0.414055
F 5.465109 -1.026466 0.154342
F 5.119244 1.224838 0.522716
F 5.143560 0.397813 -1.633119
47
10-BF4-GS_48 Eopt
-1349.643310
O -1.298287 0.454035 -0.520816
C -2.496161 -0.358479 -0.396015
C -3.729658 0.175155 -0.666040
C -4.786811 -0.720736 -0.464867
C -4.560730 -2.042957 -0.020406
C -5.735844 -2.973517 0.152632
C -3.257677 -2.494872 0.254326
C -2.181555 -1.618860 0.070730
C -0.727299 -1.678302 0.220111
C -0.182916 -0.448223 -0.168321
C 1.153023 -0.176756 -0.392055
C 2.001583 -1.239463 -0.022566
C 1.534656 -2.472736 0.461108
C 0.152982 -2.705421 0.553307
C 2.518191 -3.544833 0.855185
C 1.693260 1.083025 -1.013118
C -3.772788 1.625961 -1.078236
C -2.725037 2.389241 -0.288590
C -1.506320 1.805618 0.055078
C -0.557944 2.355898 0.891885
C -0.813603 3.643593 1.375134
C -1.993583 4.303293 1.027720
C -2.944755 3.674081 0.220489
H -5.804342 -0.387108 -0.647195
H -6.558888 -2.476419 0.675969
H -6.118134 -3.299129 -0.822269
H -5.454983 -3.865800 0.717897
H -3.094078 -3.506403 0.610395
H 3.070170 -1.096721 -0.146898
H -0.227202 -3.676690 0.852301
H 2.054936 -4.535178 0.835454
H 3.386962 -3.548856 0.190877
H 2.888249 -3.367982 1.872491
H 2.094878 1.763245 -0.255797
H 0.929238 1.618341 -1.581472
H 2.514943 0.829873 -1.686442
H -4.760050 2.057898 -0.905567
H -3.562238 1.705597 -2.153616
H 0.336895 1.820858 1.177363
H -0.087196 4.114708 2.028371
H -2.186887 5.301803 1.405810
H -3.881657 4.171500 -0.010398
B 5.668872 0.275670 -0.189278
F 5.241583 0.336310 -1.535739
F 5.496943 -1.041526 0.297885
F 7.032010 0.635030 -0.109609
F 4.897824 1.168139 0.590873
47
10-BF4-GS_49 Eopt
-1349.642484
O 1.270033 -0.091846 -0.980186
C 2.175962 -1.016608 -0.321098
C 2.062415 -2.367857 -0.524371
C 2.985021 -3.126095 0.206408
C 3.927699 -2.517867 1.066049
C 4.912249 -3.385038 1.811199
C 3.953773 -1.121608 1.227857
C 3.037686 -0.338232 0.516503
C 2.740588 1.087997 0.384555
C 1.700942 1.247521 -0.541094
C 1.288446 2.437944 -1.107275
C 1.914448 3.566843 -0.540713
C 2.901613 3.491052 0.454165
C 3.341753 2.233481 0.899180
C 3.499124 4.750713 1.026825
C 0.313436 2.572163 -2.246046
C 0.957766 -2.832879 -1.440448
C -0.258048 -1.949373 -1.227643

C -0.131435 -0.592332 -0.936402
C -1.158848 0.254766 -0.577894
C -2.450163 -0.283062 -0.578993
C -2.650969 -1.624514 -0.908141
C -1.565323 -2.449615 -1.211248
H 2.968379 -4.208371 0.115552
H 4.404173 -4.215123 2.312938
H 5.644836 -3.820450 1.121329
H 5.457663 -2.809581 2.563476
H 4.669903 -0.662057 1.900610
H 1.628815 4.542437 -0.924224
H 4.155203 2.151494 1.612552
H 2.947513 5.061595 1.922722
H 4.541856 4.597306 1.319461
H 3.454603 5.574521 0.309110
H -0.679040 2.867080 -1.889678
H 0.207969 1.642365 -2.808887
H 0.662391 3.350620 -2.929728
H 0.699001 -3.875928 -1.250296
H 1.295834 -2.762685 -2.483450
H -0.986464 1.282451 -0.291100
H -3.287752 0.348894 -0.308081
H -3.655212 -2.034018 -0.898833
H -1.719760 -3.503912 -1.419362
B -5.942514 0.217926 0.910811
F -5.337457 1.350266 0.311912
F -6.065823 -0.810239 -0.051561
F -5.136274 -0.232112 1.980731
F -7.221161 0.569207 1.395037
47
10-BF4-GS_5 Eopt
-1349.647653
O -0.199857 -0.251461 -0.383379
C -0.552563 1.140932 -0.206550
C -1.848004 1.506461 0.051385
C -2.028953 2.888964 0.175167
C -0.949545 3.791694 0.043095
C -1.199464 5.269784 0.217933
C 0.345887 3.323574 -0.237409
C 0.554728 1.946254 -0.375617
C 1.696771 1.066204 -0.622235
C 1.254407 -0.263767 -0.589717
C 2.056416 -1.386553 -0.538673
C 3.427521 -1.096864 -0.695649
C 3.937822 0.203795 -0.822808
C 3.062466 1.300493 -0.753988
C 5.417717 0.427300 -1.005256
C 1.577708 -2.789919 -0.281934
C -2.868014 0.398740 0.131281
C -2.519572 -0.663960 -0.893138
C -1.194852 -0.969087 -1.203259
C -0.782954 -1.817742 -2.209909
C -1.782306 -2.478229 -2.932423
C -3.127616 -2.250515 -2.638785
C -3.488521 -1.340343 -1.642084
H -3.023829 3.275209 0.377954
H -1.400849 5.506174 1.269543
H -0.334858 5.858435 -0.099331
H -2.070129 5.594412 -0.361323
H 1.169003 4.022365 -0.343378
H 4.120616 -1.933438 -0.677414
H 3.444556 2.315991 -0.769800
H 5.655236 0.583026 -2.064964
H 5.752828 1.315328 -0.460778
H 5.996013 -0.432941 -0.657640
H 2.273676 -3.287000 0.398647
H 1.544271 -3.375009 -1.207450
H 0.587410 -2.806858 0.174236
H -3.877768 0.773525 -0.045276
H -2.843501 -0.040257 1.137126
H 0.262040 -1.963592 -2.443808
H -1.496850 -3.160312 -3.726001
H -3.900107 -2.764977 -3.201013
H -4.535421 -1.133957 -1.441995
B -0.283101 -1.355419 2.911819
F 0.782775 -2.242479 3.183356
F 0.236536 -0.107895 2.500710
F -1.094402 -1.894474 1.886437
F -1.062960 -1.177445 4.077724
47
10-BF4-GS_50 Eopt
-1349.642421
O 1.272267 -0.101079 -0.990834
C 2.184067 -1.006153 -0.313716
C 2.097735 -2.360761 -0.508427
C 3.021803 -3.098891 0.240784
C 3.941511 -2.466661 1.110357
C 4.929734 -3.312064 1.874972
C 3.942696 -1.069133 1.260110
C 3.024843 -0.305646 0.529978
C 2.705820 1.113650 0.385967
C 1.676030 1.250006 -0.553561
C 1.252699 2.428121 -1.134076
C 1.853341 3.572741 -0.571454
C 2.828720 3.519845 0.436672
C 3.283843 2.270992 0.899572
C 3.398547 4.791396 1.007756
C 0.287214 2.538909 -2.285447
C 1.012386 -2.850520 -1.434602
C -0.221045 -1.984271 -1.245425
C -0.121715 -0.623575 -0.960668
C -1.165670 0.206853 -0.619086
C -2.448811 -0.351034 -0.634493
C -2.622912 -1.697271 -0.959169
C -1.520892 -2.507589 -1.244305
H 3.023780 -4.181861 0.159881
H 5.452312 -2.723179 2.634019
H 4.429928 -4.151498 2.370151
H 5.684008 -3.735133 1.199508
H 4.642204 -0.594212 1.940360
H 1.558051 4.539983 -0.964481
H 4.089433 2.209936 1.622791
H 3.319399 5.618537 0.296961
H 2.853639 5.080562 1.915796
H 4.450932 4.666766 1.282010
H 0.207800 1.605381 -2.846046
H 0.629108 3.324014 -2.967900
H -0.715366 2.813146 -1.942202
H 0.767883 -3.894155 -1.237897
H 1.361653 -2.783290 -2.474849
H -1.014083 1.238593 -0.332155
H -3.300504 0.268311 -0.371565
H -3.621643 -2.124328 -0.964306
H -1.654066 -3.562678 -1.447988
B -5.913108 0.213681 0.937206
F -6.122805 -0.722446 -0.102197
F -5.270614 1.363070 0.409839
F -7.157740 0.585409 1.494468
F -5.095143 -0.362514 1.939904
47
10-BF4-GS_51 Eopt
-1349.642450
O -1.269205 -0.081336 0.956183
C -2.179805 -1.025315 0.332018
C -2.039103 -2.372809 0.542833
C -2.970767 -3.151351 -0.154447
C -3.947262 -2.565073 -0.991249
C -4.940828 -3.453400 -1.698532
C -3.999347 -1.170811 -1.164347
C -3.075516 -0.367432 -0.486249
C -2.795913 1.064205 -0.375480
C -1.732740 1.247220 0.518510
C -1.322599 2.448332 1.063290
C -1.980201 3.563222 0.504950
C -2.993620 3.464129 -0.461151
C -3.427948 2.196397 -0.882473
C -3.625745 4.709570 -1.027595
C -0.321331 2.605099 2.175894
C -0.900827 -2.812901 1.429749
C 0.294447 -1.912832 1.174082
C 0.137880 -0.560657 0.875214
C 1.140453 0.299547 0.479684
C 2.439754 -0.217662 0.448034
C 2.671948 -1.552638 0.783263
C 1.608767 -2.392176 1.124310
H -2.934920 -4.232422 -0.055051
H -5.657247 -3.877834 -0.985127
H -5.504736 -2.897111 -2.451581
H -4.437950 -4.291225 -2.192399
H -4.741420 -0.728175 -1.820144
H -1.698771 4.546171 0.872509
H -4.259964 2.096576 -1.571718
H -3.574662 5.540071 -0.318060
H -3.103219 5.021230 -1.940506
H -4.673474 4.537857 -1.290710
H -0.664267 3.383782 2.862345
H 0.657795 2.911076 1.793053
H -0.189068 1.681096 2.742690
H -0.631695 -3.853552 1.240830
H -1.209171 -2.738788 2.481685
H 0.942155 1.322029 0.191131
H 3.260307 0.425044 0.151161
H 3.682885 -1.943647 0.750293
H 1.786110 -3.441936 1.337151
B 6.023357 0.216775 -0.847975
F 5.410478 1.337244 -0.235839
F 5.323937 -0.109479 -2.031961
F 7.365197 0.526290 -1.158762
F 5.983719 -0.881767 0.041120
47
10-BF4-GS_52 Eopt
-1349.645826
O -0.978516 -0.328403 0.874563
C -2.246282 0.100093 0.312808
C -3.319166 -0.753283 0.288024
C -4.459114 -0.206281 -0.312877
C -4.460922 1.106529 -0.836922
C -5.725605 1.649323 -1.455889
C -3.302339 1.901424 -0.785662
C -2.144621 1.378427 -0.197254
C -0.785134 1.847933 0.072628
C -0.087544 0.840628 0.750323
C 1.132749 0.972560 1.382792
C 1.741288 2.221385 1.151977
C 1.145892 3.249800 0.404517
C -0.146973 3.071925 -0.113773
C 1.888725 4.541007 0.174934
C 1.772231 -0.062851 2.266235

C -3.106303 -2.136465 0.853183
C -1.705369 -2.599864 0.495169
C -0.637357 -1.705867 0.432592
C 0.626401 -1.987095 -0.040531
C 0.875037 -3.312677 -0.413493
C -0.135301 -4.271512 -0.323429
C -1.415597 -3.912673 0.107143
H -5.360378 -0.808658 -0.383157
H -6.122943 0.958868 -2.207424
H -6.503294 1.784939 -0.695110
H -5.549446 2.615457 -1.935374
H -3.307448 2.903247 -1.201629
H 2.717989 2.391668 1.595326
H -0.653035 3.880861 -0.630128
H 2.572158 4.438917 -0.676912
H 1.200448 5.361655 -0.045851
H 2.491508 4.813304 1.046316
H 1.040481 -0.768803 2.665690
H 2.267541 0.433221 3.104939
H 2.534526 -0.630523 1.724502
H -3.842913 -2.841283 0.463285
H -3.225771 -2.105650 1.944890
H 1.389758 -1.229506 -0.152856
H 1.859818 -3.575050 -0.783981
H 0.063886 -5.297184 -0.616449
H -2.213618 -4.648591 0.127399
B 4.232666 -0.404579 -0.869159
F 4.708171 0.567633 0.038339
F 4.134942 -1.654016 -0.212217
F 2.953595 -0.025728 -1.340881
F 5.122998 -0.512508 -1.959593
47
10-BF4-GS_58 Eopt
-1349.644542
O 1.205617 -0.532339 0.668808
C 0.590241 0.777113 0.512570
C -0.731576 0.964736 0.823846
C -1.185415 2.266829 0.583620
C -0.328982 3.264738 0.065088
C -0.876640 4.650938 -0.169482
C 1.011439 2.972272 -0.242000
C 1.490778 1.675419 -0.021442
C 2.764093 0.973001 -0.181144
C 2.609481 -0.346617 0.265020
C 3.625476 -1.254188 0.492040
C 4.888383 -0.791947 0.069061
C 5.106572 0.484866 -0.471082
C 4.035284 1.388817 -0.567573
C 6.485172 0.891283 -0.925636
C 3.465643 -2.590364 1.166197
C -1.498263 -0.238150 1.313207
C -1.023446 -1.458498 0.546710
C 0.307869 -1.605268 0.161134
C 0.804695 -2.593058 -0.663673
C -0.100341 -3.571589 -1.089175
C -1.439818 -3.506524 -0.701813
C -1.897938 -2.451977 0.091529
H -2.224374 2.507890 0.786948
H -1.732470 4.620620 -0.852760
H -1.226696 5.096213 0.768596
H -0.117903 5.310295 -0.598610
H 1.659667 3.740101 -0.650800
H 5.736160 -1.458373 0.201791
H 4.197356 2.405129 -0.911258
H 6.605789 0.699185 -1.999025
H 6.657143 1.959168 -0.762224
H 7.260258 0.327523 -0.399353
H 4.305760 -2.754561 1.846351
H 3.466411 -3.408479 0.438230
H 2.540845 -2.651997 1.743436
H -2.571100 -0.102334 1.179303
H -1.312468 -0.375410 2.387474
H 1.835709 -2.611141 -0.986852
H 0.251109 -4.369526 -1.734500
H -2.139828 -4.262028 -1.043156
H -2.950817 -2.367023 0.339253
B -5.086829 0.062654 -0.281898
F -6.326751 0.451760 -0.832146
F -4.069911 0.207026 -1.251799
F -4.788393 0.877584 0.836703
F -5.155788 -1.288681 0.132148
47
10-BF4-GS_59 Eopt
-1349.644189
O 1.146338 -0.480994 0.483699
C 0.750535 0.897433 0.243477
C -0.565318 1.270073 0.338327
C -0.787675 2.621856 0.049491
C 0.271992 3.487372 -0.304993
C -0.027543 4.938990 -0.587171
C 1.590433 3.007634 -0.396656
C 1.842091 1.658083 -0.122361
C 3.014986 0.784767 -0.081633
C 2.609142 -0.496702 0.315680
C 3.439637 -1.533003 0.695317
C 4.806374 -1.252926 0.490578
C 5.283742 -0.023555 0.010838
C 4.377166 1.018073 -0.248336
C 6.760795 0.186244 -0.204964
C 2.991868 -2.826491 1.321551
C -1.564582 0.194573 0.680827
C -1.142241 -1.097742 0.007039
C 0.200500 -1.431422 -0.160623
C 0.681786 -2.501319 -0.886379
C -0.269302 -3.363637 -1.442382
C -1.631572 -3.111881 -1.270363
C -2.061572 -1.982792 -0.569068
H -1.801675 3.008717 0.092027
H -0.351331 5.453109 0.325310
H 0.853396 5.457390 -0.974175
H -0.836010 5.037562 -1.319228
H 2.397043 3.674908 -0.681113
H 5.521050 -2.028216 0.752562
H 4.731738 1.999295 -0.546563
H 7.049481 1.219498 0.008945
H 7.354250 -0.480200 0.426814
H 7.027749 -0.020247 -1.248833
H 3.010117 -3.648255 0.597945
H 1.982434 -2.759276 1.732215
H 3.675017 -3.089162 2.133738
H -2.569400 0.471763 0.362069
H -1.593995 0.055967 1.770243
H 1.739145 -2.667058 -1.036435
H 0.067314 -4.221127 -2.015174
H -2.365787 -3.782072 -1.705369
H -3.120496 -1.764096 -0.477863
B -5.500368 -0.017440 0.261425
F -4.740617 0.948222 -0.442298
F -5.443876 -1.249986 -0.430722
F -4.960358 -0.182431 1.557320
F -6.840299 0.412514 0.355367
47
10-BF4-TS_1 Eopt
-1349.627011
C -3.864694 -1.236826 -0.531287
C -2.969026 -2.300667 -0.679925
C -1.560475 -2.197242 -0.748910
C -1.103279 -0.878338 -0.656400
C -1.959755 0.223026 -0.523063
C -3.340182 0.050216 -0.460471
O 0.309097 -0.258918 -0.648395
C 0.122697 1.172957 -0.574481
C -1.222556 1.464478 -0.480399
C 1.131696 2.107220 -0.614487
C 0.709210 3.440457 -0.527431
C -0.643885 3.802187 -0.405584
C -1.621330 2.799067 -0.389359
C 2.559521 1.695652 -0.739754
C 2.712323 0.216479 -0.971546
C 1.693865 -0.735374 -0.882091
C 1.932624 -2.091761 -0.955619
C 3.227458 -2.547478 -1.198750
C 4.269891 -1.635030 -1.350123
C 4.001774 -0.276476 -1.225243
H -3.376968 -3.303925 -0.749422
H -3.981774 0.918830 -0.354839
H 1.467696 4.218295 -0.553892
H -2.673418 3.051669 -0.310052
H 3.041696 2.248387 -1.552851
H 3.098189 1.958903 0.178825
H 1.150858 -2.803561 -0.806560
H 3.404484 -3.616228 -1.254825
H 5.281921 -1.976247 -1.541479
H 4.807763 0.447477 -1.303095
C -0.840604 -3.512068 -0.918038
H -1.583747 -4.307106 -0.993539
H -0.242280 -3.543414 -1.831199
H -0.211255 -3.746605 -0.055270
C -5.346320 -1.488802 -0.430174
H -5.651217 -2.327209 -1.063430
H -5.622194 -1.737954 0.601773
H -5.918292 -0.603815 -0.722414
C -1.030245 5.253922 -0.274961
H -0.890645 5.597729 0.757262
H -0.412594 5.888775 -0.917683
H -2.080095 5.409422 -0.538142
B 0.652091 -0.479029 2.718297
F 1.693237 0.282709 2.140149
F 0.574353 -1.735170 2.074053
F 0.917401 -0.670563 4.093023
F -0.573170 0.205188 2.563341
47
10-BF4-TS_10 Eopt
-1349.623734
C 5.087303 -1.235148 0.173041
C 5.074875 0.163511 0.197841
C 3.926007 0.985089 0.135536
C 2.735630 0.254822 0.038450
C 2.693414 -1.146700 0.009336
C 3.866383 -1.895845 0.074404
O 1.255234 0.676820 -0.053873
C 0.486276 -0.549334 -0.133981
C 1.337211 -1.634754 -0.094204
C -0.885091 -0.619727 -0.234942
C -1.407721 -1.918288 -0.292883
C -0.594881 -3.065866 -0.252980
C 0.794299 -2.920744 -0.153339
C -1.725481 0.611307 -0.279116

C -0.905681 1.871388 -0.216815
C 0.485219 1.949102 -0.110086
C 1.168889 3.146538 -0.050548
C 0.455317 4.343298 -0.099980
C -0.934220 4.321183 -0.208085
C -1.590187 3.096897 -0.263840
H 6.026720 0.679528 0.271079
H 3.809613 -2.978968 0.045170
H -2.485945 -2.025930 -0.368220
H 1.441532 -3.790714 -0.120286
H -2.327479 0.618476 -1.194330
H -2.442084 0.603308 0.548506
H 2.232841 3.177227 0.035045
H 1.000255 5.280027 -0.052434
H -1.501672 5.245068 -0.246534
H -2.672393 3.059613 -0.345335
C 4.208242 2.466456 0.184413
H 5.285178 2.612197 0.278199
H 3.896262 2.977841 -0.730013
H 3.745438 2.945869 1.050653
C 6.385884 -1.991357 0.276355
H 6.282825 -3.007396 -0.113890
H 7.184478 -1.484927 -0.273824
H 6.704337 -2.065981 1.323303
C -1.227587 -4.432850 -0.321064
H -1.754421 -4.571479 -1.271969
H -0.477327 -5.222413 -0.227568
H -1.965376 -4.561596 0.478304
B -5.334634 -0.333952 0.270310
F -6.677321 -0.600672 0.613938
F -4.577691 -0.123565 1.447190
F -4.800524 -1.435019 -0.440000
F -5.272918 0.822765 -0.540185
47
10-BF4-TS_11 Eopt
-1349.623805
C 1.540095 3.889422 -0.070477
C 0.208821 3.461192 -0.132198
C -0.232733 2.118679 -0.139269
C 0.821484 1.199504 -0.081888
C 2.169659 1.573976 -0.014747
C 2.535016 2.919184 -0.008811
O 0.861420 -0.347320 -0.066285
C 2.257052 -0.715494 0.011020
C 3.038898 0.421676 0.044287
C 2.739022 -2.005588 0.042735
C 4.133427 -2.115340 0.121885
C 4.984151 -0.995756 0.163119
C 4.426998 0.288029 0.121835
C 1.818529 -3.178807 -0.010977
C 0.371077 -2.773188 -0.101082
C -0.121429 -1.465577 -0.123733
C -1.467707 -1.170909 -0.190326
C -2.393471 -2.212362 -0.245791
C -1.953081 -3.534391 -0.234984
C -0.589518 -3.795182 -0.162006
H -0.568056 4.218141 -0.177383
H 3.585168 3.186313 0.046136
H 4.564748 -3.112534 0.150638
H 5.060165 1.168611 0.147321
H 2.070352 -3.808397 -0.872202
H 1.960695 -3.803785 0.878357
H -1.822590 -0.163493 -0.195230
H -3.448995 -1.970317 -0.291014
H -2.663863 -4.352983 -0.278362
H -0.229565 -4.819888 -0.146857
C -1.728172 1.940937 -0.204013
H -2.196146 2.925562 -0.225861
H -2.043525 1.420276 -1.110323
H -2.123730 1.422934 0.671707
C 1.867459 5.359685 -0.069409
H 1.398791 5.863002 0.783448
H 2.946594 5.523408 -0.014071
H 1.492503 5.843347 -0.978211
C 6.477280 -1.188075 0.250299
H 7.000274 -0.228362 0.254919
H 6.748720 -1.729644 1.163487
H 6.846652 -1.775440 -0.597668
B -5.388910 0.217179 0.184085
F -6.561468 0.960106 0.441659
F -5.722908 -1.142556 -0.018606
F -4.755140 0.719865 -0.975251
F -4.509610 0.324387 1.286443
47
10-BF4-TS_13 Eopt
-1349.623778
C -1.486310 3.893571 -0.001876
C -0.159416 3.447677 -0.003171
C 0.264209 2.099325 -0.003225
C -0.803600 1.194229 -0.002123
C -2.148278 1.586497 -0.000601
C -2.495633 2.936375 -0.000483
O -0.865294 -0.352883 -0.001910
C -2.267965 -0.701552 -0.000012
C -3.034947 0.446195 0.000854
C -2.768838 -1.984731 0.000720
C -4.166850 -2.075198 0.002796
C -5.003118 -0.944132 0.003962
C -4.426920 0.331866 0.002892
C -1.863147 -3.170478 -0.000852
C -0.407348 -2.785498 -0.002464
C 0.103904 -1.484711 -0.002968
C 1.456016 -1.209148 -0.004228
C 2.367709 -2.264272 -0.005090
C 1.909316 -3.580021 -0.004827
C 0.540212 -3.821369 -0.003513
H 0.628927 4.194015 -0.004042
H -3.543724 3.216860 0.000784
H -4.613194 -3.066156 0.003518
H -5.048114 1.221306 0.003611
H -2.068552 -3.795319 0.876075
H -2.070704 -3.794563 -0.877822
H 1.826272 -0.207017 -0.004396
H 3.427080 -2.036889 -0.005895
H 2.609861 -4.408496 -0.005537
H 0.165609 -4.840949 -0.003179
C 1.758294 1.901724 -0.003782
H 2.240108 2.879964 -0.003421
H 2.107521 1.376728 0.887323
H 2.107047 1.377627 -0.895719
C -1.794168 5.368084 -0.001629
H -1.365726 5.855729 0.881016
H -1.364460 5.856389 -0.883271
H -2.872540 5.545930 -0.002281
C -6.501231 -1.116205 0.006283
H -7.010486 -0.149104 0.007186
H -6.831907 -1.676249 -0.875578
H -6.829134 -1.676452 0.889048
B 5.391390 0.209047 0.004101
F 4.600662 0.343550 -1.160467
F 4.574196 0.350589 1.149430
F 5.995089 -1.069322 0.014870
F 6.390073 1.207971 0.012542
47
10-BF4-TS_14 Eopt
-1349.623744
C 5.092963 -1.219422 0.173925
C 5.074040 0.179955 0.201191
C 3.922441 0.995859 0.138144
C 2.734632 0.259947 0.038791
C 2.698625 -1.141015 0.011691
C 3.875688 -1.885269 0.077959
O 1.252294 0.675494 -0.051061
C 0.488725 -0.554101 -0.128781
C 1.344602 -1.635641 -0.087788
C -0.882288 -0.630869 -0.230145
C -1.399097 -1.931887 -0.284937
C -0.581161 -3.075748 -0.242442
C 0.807397 -2.924217 -0.144020
C -1.727982 0.596301 -0.279261
C -0.913963 1.860135 -0.217539
C 0.476409 1.944359 -0.109015
C 1.154346 3.144979 -0.048175
C 0.435334 4.338406 -0.099217
C -0.953883 4.309798 -0.209825
C -1.604051 3.082457 -0.266113
H 6.023558 0.699938 0.278718
H 3.823459 -2.968650 0.053329
H -2.476807 -2.044476 -0.360363
H 1.458535 -3.791249 -0.109910
H -2.327429 0.598662 -1.196189
H -2.446880 0.587167 0.546305
H 2.217979 3.180637 0.039600
H 0.975819 5.277659 -0.050712
H -1.525568 5.231018 -0.249486
H -2.685958 3.040126 -0.349025
C 4.196852 2.478536 0.190969
H 5.272868 2.629685 0.286884
H 3.883801 2.990213 -0.722943
H 3.729923 2.953599 1.057381
C 6.400284 -1.964329 0.247109
H 6.948569 -1.702515 1.158738
H 6.239094 -3.045238 0.241848
H 7.042240 -1.709092 -0.603465
C -1.207837 -4.445668 -0.307172
H -1.943281 -4.576553 0.494020
H -1.736104 -4.588077 -1.256715
H -0.453847 -5.231714 -0.214100
B -5.334673 -0.332363 0.263730
F -4.582101 -0.120935 1.443275
F -5.274984 0.825917 -0.544708
F -4.794148 -1.430126 -0.446682
F -6.677369 -0.604709 0.602985
47
10-BF4-TS_15 Eopt
-1349.623648
C 5.127534 -1.153614 0.158085
C 5.083554 0.245202 0.184345
C 3.917268 1.040258 0.124996
C 2.742434 0.283178 0.030325
C 2.731354 -1.118254 0.006406
C 3.921909 -1.841134 0.068440
O 1.252491 0.671977 -0.056683
C 0.510375 -0.571436 -0.120703
C 1.385678 -1.637404 -0.081683
C -0.860073 -0.673373 -0.206380
C -1.354159 -1.983689 -0.247535
C -0.515567 -3.112584 -0.208558

C 0.870971 -2.935689 -0.125732
C -1.727884 0.538187 -0.256113
C -0.937061 1.816691 -0.195851
C 0.452684 1.926513 -0.104527
C 1.109059 3.139579 -0.055263
C 0.367420 4.319471 -0.095591
C -1.022586 4.265119 -0.184978
C -1.650496 3.025925 -0.233349
H 6.023686 0.782356 0.258488
H 3.888519 -2.925340 0.046620
H -2.430653 -2.115423 -0.307645
H 1.538056 -3.790714 -0.092911
H -2.325069 0.529207 -1.174608
H -2.448981 0.518747 0.567346
H 2.173267 3.194950 0.014466
H 0.891142 5.268564 -0.055877
H -1.611768 5.175596 -0.215612
H -2.732445 2.963174 -0.302194
C 4.165184 2.527595 0.178804
H 5.238071 2.697831 0.277826
H 3.845903 3.033596 -0.736230
H 3.686716 2.993679 1.043594
C 6.448258 -1.874930 0.226357
H 6.999346 -1.596279 1.131230
H 6.306194 -2.958536 0.230755
H 7.078570 -1.615277 -0.631597
C -1.118204 -4.493786 -0.259881
H -0.349652 -5.265719 -0.168064
H -1.845062 -4.632794 0.547744
H -1.651024 -4.651448 -1.204476
B -5.372421 -0.326171 0.234321
F -5.279020 0.574512 -0.851411
F -6.730022 -0.562709 0.538742
F -4.745697 -1.546420 -0.112335
F -4.725100 0.229939 1.363150
47
10-BF4-TS_17 Eopt
-1349.623725
C -5.299033 0.466767 0.239444
C -5.003409 -0.901111 0.227915
C -3.714659 -1.471412 0.122703
C -2.697748 -0.512810 0.025369
C -2.941357 0.867663 0.029706
C -4.239991 1.362707 0.136335
O -1.164545 -0.622837 -0.108929
C -0.662360 0.734913 -0.161749
C -1.715304 1.623339 -0.084224
C 0.664957 1.086478 -0.266289
C 0.909529 2.465678 -0.297320
C -0.119141 3.422665 -0.227003
C -1.447765 2.993939 -0.118758
C 1.738970 0.054630 -0.333522
C 1.196551 -1.347574 -0.271128
C -0.148434 -1.710983 -0.171806
C -0.571565 -3.024159 -0.133794
C 0.374233 -4.047179 -0.189317
C 1.730162 -3.737459 -0.281272
C 2.121078 -2.404112 -0.321149
H -5.829889 -1.600318 0.307450
H -4.402542 2.435400 0.136856
H 1.941551 2.796325 -0.377000
H -2.259201 3.711696 -0.059794
H 2.315448 0.175353 -1.257442
H 2.451699 0.201451 0.483964
H -1.606914 -3.275767 -0.061963
H 0.033852 -5.076681 -0.158161
H 2.476475 -4.523987 -0.322136
H 3.171750 -2.139536 -0.391595
C -3.690080 -2.980244 0.138426
H -4.713311 -3.343056 0.242881
H -3.298074 -3.396496 -0.793300
H -3.122946 -3.373407 0.985654
C -6.723007 0.938052 0.376215
H -6.805211 2.008227 0.169894
H -7.384167 0.399750 -0.310508
H -7.092943 0.759847 1.392862
C 0.219174 4.891567 -0.262921
H 0.773533 5.143189 -1.173783
H -0.683716 5.506701 -0.230026
H 0.852418 5.166523 0.588090
B 5.432037 0.263955 0.308520
F 4.632862 0.255719 1.476664
F 5.073446 1.368393 -0.497713
F 6.792855 0.360927 0.668434
F 5.217901 -0.933973 -0.413861
47
10-BF4-TS_18 Eopt
-1349.623733
C 5.081753 -1.278531 -0.064336
C 5.087534 0.121304 -0.076957
C 3.948415 0.956990 -0.054912
C 2.745002 0.240092 -0.018161
C 2.684668 -1.160092 -0.005306
C 3.850525 -1.924225 -0.027064
O 1.264927 0.678144 0.008738
C 0.479955 -0.539205 0.042174
C 1.320128 -1.633464 0.031194
C -0.895329 -0.595399 0.081975
C -1.432909 -1.888926 0.109145
C -0.631154 -3.044941 0.096074
C 0.762314 -2.913993 0.056364
C -1.722952 0.645407 0.094963
C -0.886793 1.896265 0.080191
C 0.508233 1.957939 0.037169
C 1.208286 3.146988 0.016364
C 0.507726 4.352117 0.042838
C -0.885450 4.346123 0.089472
C -1.558091 3.129649 0.106438
H 6.048370 0.625371 -0.105394
H 3.778284 -3.006655 -0.015064
H -2.513723 -1.986784 0.144472
H 1.402231 -3.790052 0.046110
H -2.396725 0.651975 -0.768034
H -2.369806 0.653492 0.978367
H 2.275509 3.163713 -0.020803
H 1.065327 5.282435 0.026591
H -1.443013 5.276559 0.110648
H -2.643211 3.105281 0.140040
C 4.251593 2.435608 -0.073066
H 5.333811 2.567638 -0.104732
H 3.844640 2.930559 -0.958710
H 3.895284 2.941917 0.827859
C 6.378080 -2.045507 -0.090574
H 6.998585 -1.792656 0.776344
H 6.198352 -3.123479 -0.080738
H 6.957533 -1.801679 -0.987911
C -1.280478 -4.405279 0.130363
H -1.982018 -4.524241 -0.702486
H -0.534888 -5.202373 0.069105
H -1.851934 -4.540499 1.055606
B -5.350522 -0.332908 -0.100865
F -5.270728 1.059098 0.135101
F -4.735914 -0.636208 -1.338027
F -4.683424 -1.024628 0.937367
F -6.705016 -0.729891 -0.136988
47
10-BF4-TS_19 Eopt
-1349.623262
C 5.337370 0.033595 0.397337
C 4.888800 -1.291859 0.357147
C 3.550763 -1.710818 0.181307
C 2.656509 -0.641518 0.039685
C 3.054380 0.702042 0.074699
C 4.393791 1.044889 0.252579
O 1.131488 -0.575413 -0.176901
C 0.788853 0.830164 -0.237855
C 1.929055 1.593107 -0.089769
C -0.482164 1.330099 -0.413978
C -0.567963 2.728327 -0.435183
C 0.555523 3.562562 -0.289958
C 1.819311 2.985434 -0.115956
C -1.658822 0.427018 -0.566820
C -1.283783 -1.027745 -0.480715
C 0.003600 -1.540848 -0.306081
C 0.274173 -2.892772 -0.252335
C -0.775686 -3.803256 -0.366312
C -2.080624 -3.342845 -0.533507
C -2.316083 -1.974007 -0.589578
H 5.625467 -2.081067 0.471150
H 4.677725 2.091615 0.276727
H -1.549838 3.174935 -0.567431
H 2.701078 3.606896 -0.001228
H -2.409559 0.652275 0.197330
H -2.147815 0.613420 -1.530020
H 1.270224 -3.257096 -0.127460
H -0.555463 -4.864549 -0.321753
H -2.907675 -4.039799 -0.619055
H -3.325171 -1.596184 -0.719039
C 3.353664 -3.207142 0.184305
H 4.322974 -3.684093 0.333726
H 2.707264 -3.536805 1.001635
H 2.961157 -3.574286 -0.767334
C 6.799171 0.337502 0.597177
H 7.406657 -0.132205 -0.184188
H 6.984347 1.414425 0.575529
H 7.149669 -0.051340 1.559807
C 0.387731 5.060812 -0.321987
H -0.032934 5.386554 -1.279974
H -0.299377 5.393674 0.463798
H 1.344558 5.569479 -0.178290
B -5.532919 0.202189 0.496501
F -5.627608 -1.068926 1.105621
F -6.777484 0.862637 0.577755
F -4.546539 0.970219 1.160957
F -5.167926 0.046078 -0.861716
47
10-BF4-TS_2 Eopt
-1349.626868
C -3.784641 -1.032579 -0.804968
C -2.900691 -2.100828 -0.994385
C -1.489948 -2.021360 -0.996001
C -1.015503 -0.721357 -0.788282
C -1.857538 0.383122 -0.606899
C -3.242909 0.233916 -0.613714
O 0.405941 -0.129578 -0.683343
C 0.240385 1.292053 -0.488431
C -1.102740 1.601442 -0.429184
C 1.264778 2.205998 -0.391110

C	0.859465	3.533758	-0.203404
C	-0.492615	3.911055	-0.116121
C	-1.484779	2.930436	-0.235967
C	2.691377	1.778019	-0.475048
C	2.834452	0.312607	-0.789356
C	1.794801	-0.618822	-0.849320
C	2.012799	-1.968442	-1.032738
C	3.313237	-2.433779	-1.219682
C	4.381633	-1.538861	-1.209019
C	4.130199	-0.189617	-0.987746
H	-3.322448	-3.088925	-1.148898
H	-3.873460	1.104049	-0.464465
H	1.630532	4.295016	-0.118669
H	-2.535671	3.194729	-0.181025
H	3.217959	2.368738	-1.232405
H	3.191959	1.980557	0.479826
H	1.206948	-2.668282	-1.012836
H	3.473253	-3.497198	-1.362073
H	5.398577	-1.887848	-1.354874
H	4.952608	0.518895	-0.948681
C	-0.784543	-3.335947	-1.214876
H	-1.536017	-4.113271	-1.359838
H	-0.156018	-3.327634	-2.108338
H	-0.190036	-3.624027	-0.344393
C	-5.272807	-1.265984	-0.793241
H	-5.580865	-1.897754	-1.632614
H	-5.574331	-1.777169	0.128734
H	-5.820850	-0.321828	-0.849522
C	-0.859171	5.354530	0.121045
H	-0.279417	6.021760	-0.524990
H	-1.922148	5.528650	-0.066099
H	-0.649443	5.640704	1.158691
B	0.235975	-1.006404	2.661671
F	1.085709	0.043262	2.243602
F	0.601162	-1.416034	3.964247
F	-1.102528	-0.555691	2.665083
F	0.363435	-2.097836	1.772795

47

10-BF4-TS_20 Eopt

-1349.623782

C	-5.068468	-1.264267	0.176032
C	-5.067651	0.134498	0.196765
C	-3.925562	0.965538	0.133800
C	-2.728990	0.244614	0.042032
C	-2.675401	-1.156560	0.013589
C	-3.842152	-1.915207	0.079020
O	-1.251584	0.678054	-0.045387
C	-0.473107	-0.541689	-0.130951
C	-1.315524	-1.633724	-0.092503
C	0.898483	-0.600855	-0.236919
C	1.430775	-1.895170	-0.303144
C	0.626905	-3.049121	-0.264018
C	-0.762938	-2.915224	-0.158086
C	1.728920	0.637375	-0.273810
C	0.898164	1.890566	-0.218765
C	-0.492805	1.956396	-0.105777
C	-1.186361	3.147753	-0.040708
C	-0.483702	4.350653	-0.096727
C	0.905080	4.340478	-0.215902
C	1.571536	3.121894	-0.273149
H	-6.024004	0.642740	0.265734
H	-3.776666	-2.997847	0.050822
H	2.509140	-1.994340	-0.386029
H	-1.403343	-3.790238	-0.126483
H	2.434465	0.634449	0.563336
H	2.341946	0.649624	-1.181347
H	-2.249588	3.168582	0.055302
H	-1.036282	5.282691	-0.045236
H	1.464075	5.269237	-0.260327
H	2.653576	3.094080	-0.360346
C	-4.221027	2.444816	0.173190
H	-5.300316	2.581463	0.252105
H	-3.774556	2.931731	1.043982
H	-3.900964	2.955584	-0.738625
C	-6.360854	-2.030868	0.280109
H	-6.250446	-3.045359	-0.112169
H	-6.676837	-2.110019	1.327480
H	-7.164326	-1.529714	-0.267765
C	1.269943	-4.410903	-0.339295
H	1.800633	-4.539406	-1.289443
H	2.006292	-4.539265	0.461507
H	0.525340	-5.206606	-0.252892
B	5.306474	-0.336843	0.275904
F	6.625861	-0.740934	0.573257
F	5.312508	0.998441	-0.189054
F	4.767726	-1.179737	-0.724790
F	4.510636	-0.424832	1.442286

47

10-BF4-TS_22 Eopt

-1349.623729

C	-1.275436	3.090848	-0.348675
C	0.022275	3.582575	-0.187221
C	1.199701	2.802684	-0.077072
C	0.947568	1.430183	-0.145922
C	-0.334892	0.884380	-0.303205
C	-1.450840	1.709310	-0.405776
O	1.876024	0.185575	-0.084015
C	1.010944	-0.971063	-0.194677
C	-0.301436	-0.560783	-0.328604
C	1.431284	-2.279840	-0.170495
C	0.407338	-3.232098	-0.294212
C	-0.945133	-2.879388	-0.430942
C	-1.302327	-1.523057	-0.452705
C	2.875068	-2.629395	-0.023750
C	3.748385	-1.411820	0.131551
C	3.319541	-0.081411	0.104589
C	4.185145	0.985879	0.237470
C	5.546896	0.746311	0.415194
C	6.023783	-0.562759	0.455499
C	5.125785	-1.614457	0.312926
H	0.156970	4.658100	-0.136290
H	-2.433498	1.264522	-0.520157
H	0.684975	-4.282534	-0.280428
H	-2.338220	-1.222624	-0.558197
H	3.014454	-3.284651	0.843585
H	3.210921	-3.200579	-0.897419
H	3.832800	1.993814	0.205526
H	6.218589	1.591632	0.519539
H	7.080938	-0.763300	0.594490
H	5.480272	-2.640704	0.338682
C	2.469800	3.598701	0.096885
H	2.218198	4.659885	0.095713
H	2.961133	3.388743	1.050490
H	3.174494	3.437980	-0.722883
C	-2.451144	4.024221	-0.468659
H	-3.278533	3.691539	0.165821
H	-2.180659	5.044574	-0.184932
H	-2.821872	4.045261	-1.500343
C	-2.010694	-3.941736	-0.526050
H	-2.516989	-4.064718	0.438812
H	-2.775924	-3.667523	-1.258207
H	-1.585222	-4.908512	-0.808727
B	-4.943382	-0.348045	0.464452
F	-4.384158	0.017394	-0.786123
F	-6.226009	0.227574	0.588982
F	-5.041625	-1.755020	0.537443
F	-4.108627	0.122387	1.502795

47

10-BF4-TS_24 Eopt

-1349.623364

C	1.353139	2.986019	-0.131909
C	0.062504	3.525155	-0.077024
C	-1.143914	2.790494	-0.034278
C	-0.935676	1.406101	-0.054773
C	0.333199	0.814331	-0.105456
C	1.483733	1.600752	-0.144177
O	-1.908814	0.197818	-0.038088
C	-1.081614	-0.989653	-0.071559
C	0.249970	-0.629059	-0.111081
C	-1.552854	-2.283057	-0.062579
C	-0.561003	-3.272166	-0.093226
C	0.812314	-2.970786	-0.133703
C	1.222254	-1.631198	-0.143580
C	-3.016305	-2.575765	-0.026748
C	-3.852223	-1.325118	0.045186
C	-3.372230	-0.012288	0.035738
C	-4.203326	1.088527	0.087210
C	-5.583119	0.902250	0.158418
C	-6.111562	-0.387363	0.177224
C	-5.246139	-1.473948	0.120519
H	-0.037609	4.605938	-0.064383
H	2.457472	1.125802	-0.182165
H	-0.877854	-4.311795	-0.084074
H	2.275233	-1.375825	-0.174155
H	-3.303230	-3.145942	-0.918293
H	-3.249510	-3.213235	0.833628
H	-3.811434	2.081870	0.074034
H	-6.227884	1.773692	0.198545
H	-7.183250	-0.546304	0.234223
H	-5.640019	-2.486011	0.132607
C	-2.395051	3.632895	0.030396
H	-2.104974	4.684259	0.030652
H	-3.042166	3.480650	-0.837251
H	-2.962450	3.459072	0.948340
C	2.555150	3.892713	-0.178669
H	2.555380	4.584893	0.670400
H	3.482853	3.317223	-0.154979
H	2.549616	4.497590	-1.092680
C	1.826673	-4.085859	-0.175361
H	2.842166	-3.699852	-0.062888
H	1.642561	-4.813919	0.621915
H	1.772142	-4.625180	-1.128438
B	5.102237	-0.316974	0.162488
F	6.473429	-0.499473	0.441263
F	4.371704	-0.277264	1.372182
F	4.916920	0.897971	-0.537505
F	4.633364	-1.388841	-0.633292

47

10-BF4-TS_26 Eopt

-1349.623418

C	-1.342870	3.000501	-0.025981
C	-0.048144	3.532687	-0.016685
C	1.155058	2.791688	-0.012554
C	0.938965	1.408417	-0.018951
C	-0.333974	0.823520	-0.028483
C	-1.481106	1.615971	-0.031487
O	1.905699	0.195028	-0.023450
C	1.071693	-0.988056	-0.026746

C	-0.258209	-0.620028	-0.032680
C	1.535297	-2.284253	-0.019710
C	0.537093	-3.267672	-0.022304
C	-0.835037	-2.958016	-0.032240
C	-1.236650	-1.616231	-0.036491
C	2.997455	-2.585022	-0.007790
C	3.842237	-1.338473	0.017221
C	3.369793	-0.022877	0.004276
C	4.208332	1.073559	0.010212
C	5.588615	0.879713	0.038152
C	6.109942	-0.412774	0.059187
C	5.237080	-1.494844	0.047708
H	0.058643	4.612775	-0.011699
H	-2.458247	1.146402	-0.037305
H	0.847928	-4.309157	-0.015178
H	-2.287413	-1.352247	-0.042415
H	3.244300	-3.203072	0.863081
H	3.263161	-3.178197	-0.890699
H	3.821518	2.068842	-0.007172
H	6.239434	1.747582	0.042976
H	7.182022	-0.577489	0.082384
H	5.626132	-2.508739	0.061418
C	2.412291	3.627165	-0.000319
H	2.128155	4.680302	0.003940
H	3.013774	3.455527	0.896000
H	3.024268	3.465838	-0.891638
C	-2.541436	3.912863	-0.030710
H	-2.540201	4.551828	-0.921012
H	-3.470922	3.339831	-0.020212
H	-2.532270	4.572726	0.843957
C	-1.858728	-4.065177	-0.045405
H	-1.680763	-4.774689	0.769972
H	-2.870655	-3.666860	0.058789
H	-1.810305	-4.628682	-0.984565
B	-5.087539	-0.319149	0.040734
F	-4.441650	-0.794101	1.205991
F	-6.442781	-0.711957	0.056443
F	-5.000810	1.091291	-0.001104
F	-4.449495	-0.861117	-1.099775

47

10-BF4-TS_28 Eopt

-1349.623732

C	1.274160	3.079312	-0.229329
C	-0.027598	3.575554	-0.124976
C	-1.210535	2.799568	-0.051296
C	-0.959105	1.425872	-0.092988
C	0.327176	0.875522	-0.195198
C	1.448332	1.696700	-0.263452
O	-1.891774	0.184189	-0.048377
C	-1.027396	-0.975586	-0.121694
C	0.290623	-0.569941	-0.210288
C	-1.453091	-2.282310	-0.107911
C	-0.428371	-3.238774	-0.190141
C	0.928882	-2.891416	-0.279936
C	1.291718	-1.535893	-0.293129
C	-2.902679	-2.626551	-0.016960
C	-3.778297	-1.405186	0.086395
C	-3.343753	-0.076457	0.071411
C	-4.210198	0.994775	0.159487
C	-5.579803	0.760939	0.272919
C	-6.063164	-0.546192	0.296411
C	-5.163468	-1.601987	0.203185
H	-0.162127	4.651886	-0.095222
H	2.433385	1.249147	-0.339474
H	-0.710091	-4.288132	-0.183306
H	2.331576	-1.239965	-0.366240
H	-3.203075	-3.208388	-0.896411
H	-3.080562	-3.270084	0.852235
H	-3.852228	2.000992	0.141839
H	-6.252389	1.609241	0.341534
H	-7.126567	-0.742156	0.385190
H	-5.522342	-2.626937	0.218459
C	-2.484542	3.600956	0.059954
H	-2.229501	4.661322	0.056100
H	-3.155355	3.431289	-0.785948
H	-3.014978	3.404659	0.995397
C	2.456558	4.008066	-0.314196
H	2.891078	3.986447	-1.320516
H	2.171170	5.038865	-0.089115
H	3.242658	3.701777	0.383223
C	1.993618	-3.956864	-0.345894
H	2.703342	-3.752802	-1.153802
H	2.568702	-3.985977	0.586765
H	1.556769	-4.945881	-0.507296
B	5.010081	-0.337797	0.302223
F	5.009067	-1.746162	0.408220
F	4.320557	0.219593	1.402937
F	6.338343	0.138461	0.283357
F	4.356468	0.041391	-0.896930

47

10-BF4-TS_29 Eopt

-1349.623616

C	1.269124	3.065474	-0.092795
C	-0.037098	3.566996	-0.053795
C	-1.221735	2.796715	-0.024583
C	-0.972914	1.419131	-0.039354
C	0.312912	0.865231	-0.077543
C	1.440360	1.684726	-0.104632
O	-1.911092	0.182611	-0.029694
C	-1.049473	-0.980698	-0.055543
C	0.273151	-0.580169	-0.084756
C	-1.480962	-2.285331	-0.049280
C	-0.457556	-3.246452	-0.074985
C	0.903664	-2.905101	-0.105658
C	1.272703	-1.550844	-0.110637
C	-2.934456	-2.623177	-0.018639
C	-3.808726	-1.398071	0.035262
C	-3.368706	-0.071202	0.027595
C	-4.233726	1.004086	0.065062
C	-5.608012	0.775958	0.117778
C	-6.097139	-0.529174	0.132144
C	-5.198425	-1.589139	0.090815
H	-0.169920	4.644354	-0.045756
H	2.427629	1.237620	-0.140298
H	-0.743931	-4.294477	-0.069434
H	2.317547	-1.264482	-0.131156
H	-3.198687	-3.211941	-0.905052
H	-3.152646	-3.258144	0.847696
H	-3.871177	2.008579	0.053319
H	-6.279683	1.627304	0.146712
H	-7.164273	-0.720408	0.173727
H	-5.561735	-2.612647	0.099784
C	-2.498080	3.601100	0.019992
H	-2.239447	4.660683	0.020575
H	-3.128258	3.426529	-0.855753
H	-3.072145	3.413603	0.931061
C	2.446760	4.004330	-0.122425
H	2.437885	4.669180	0.748384
H	3.389050	3.451720	-0.123595
H	2.418219	4.637445	-1.016459
C	1.965810	-3.974909	-0.135678
H	2.673864	-3.844881	0.689319
H	1.526463	-4.973012	-0.060568
H	2.543576	-3.923206	-1.065186
B	5.091825	-0.328540	0.121026
F	4.848031	-1.639555	-0.347370
F	4.570305	0.603761	-0.808151
F	4.453252	-0.148720	1.369606
F	6.480904	-0.125362	0.264252

47

10-BF4-TS_3 Eopt

-1349.626768

C	-3.818977	-1.736847	-0.233856
C	-2.793353	-2.637738	-0.542646
C	-1.431273	-2.313231	-0.732271
C	-1.169415	-0.944526	-0.591894
C	-2.161979	-0.002117	-0.292560
C	-3.487750	-0.391902	-0.110955
O	0.123153	-0.110931	-0.682032
C	-0.264409	1.265111	-0.476379
C	-1.619469	1.335168	-0.226917
C	0.582374	2.347935	-0.545596
C	-0.024483	3.590181	-0.320165
C	-1.396259	3.727082	-0.041652
C	-2.203169	2.583467	-0.000540
C	2.035318	2.173477	-0.833865
C	2.384268	0.751747	-1.182294
C	1.533862	-0.350824	-1.071076
C	1.960463	-1.645890	-1.276368
C	3.276568	-1.879532	-1.671537
C	4.151797	-0.809028	-1.843033
C	3.699651	0.481140	-1.589191
H	-3.052399	-3.686996	-0.642512
H	-4.236298	0.357629	0.123229
H	0.600671	4.478180	-0.362108
H	-3.266680	2.662323	0.198953
H	2.340842	2.838537	-1.648442
H	2.620386	2.467462	0.046041
H	1.309207	-2.476359	-1.115328
H	3.602461	-2.902300	-1.827697
H	5.178532	-0.976580	-2.151150
H	4.377041	1.324766	-1.684946
C	-0.540392	-3.492104	-1.038436
H	-1.154575	-4.393123	-1.066517
H	-0.054501	-3.404882	-2.012857
H	0.212770	-3.645711	-0.260864
C	-5.228745	-2.226632	-0.028729
H	-5.541544	-2.883699	-0.846871
H	-5.305557	-2.803084	0.900652
H	-5.931291	-1.391525	0.032651
C	-1.980152	5.091878	0.223927
H	-3.070415	5.078525	0.143099
H	-1.722933	5.431740	1.234467
H	-1.589043	5.833839	-0.479464
B	1.578264	-0.744596	2.522605
F	1.589658	0.578782	2.026716
F	0.469283	-1.438089	1.986455
F	2.771237	-1.400862	2.145404
F	1.478704	-0.717522	3.932437

47

10-BF4-TS_30 Eopt

-1349.623646

C	-1.319020	3.028867	-0.144344
C	-0.020919	3.548626	-0.080883
C	1.173917	2.795033	-0.032180
C	0.944626	1.414116	-0.055381
C	-0.332550	0.842386	-0.114324
C	-1.470577	1.645947	-0.159849

O 1.899398 0.190148 -0.033257
C 1.054235 -0.984733 -0.073744
C -0.273204 -0.602316 -0.120612
C 1.503779 -2.283260 -0.068701
C 0.494280 -3.258282 -0.112176
C -0.871137 -2.935547 -0.157429
C -1.259018 -1.586563 -0.162962
C 2.961625 -2.600879 -0.026390
C 3.817793 -1.363969 0.047825
C 3.359041 -0.043432 0.041573
C 4.207860 1.043754 0.098101
C 5.584386 0.834845 0.169713
C 6.091860 -0.463248 0.184110
C 5.209063 -1.535593 0.123330
H 0.096035 4.627752 -0.066973
H -2.450082 1.184033 -0.209666
H 0.795057 -4.302277 -0.110026
H -2.307456 -1.314487 -0.196191
H 3.180421 -3.241009 0.835928
H 3.242720 -3.177278 -0.915791
H 3.831580 2.043214 0.088056
H 6.243294 1.695461 0.213634
H 7.160841 -0.639628 0.241008
H 5.586518 -2.553951 0.132523
C 2.437882 3.617031 0.039607
H 2.164898 4.672972 0.041293
H 2.998424 3.431972 0.959593
H 3.086156 3.456298 -0.825558
C -2.509466 3.950429 -0.196817
H -2.510463 4.637202 0.656663
H -2.489482 4.560716 -1.106970
H -3.443874 3.384588 -0.184695
C -1.917821 -4.020363 -0.191324
H -1.461160 -5.010096 -0.273822
H -2.528118 -3.999038 0.718458
H -2.598616 -3.880984 -1.037398
B -5.051874 -0.326569 0.178491
F -4.414531 0.149833 1.347163
F -4.834869 -1.717834 0.058138
F -6.436577 -0.065420 0.253275
F -4.506501 0.331099 -0.950723
47
10-BF4-TS_31 Eopt
-1349.623675
C 1.296078 3.051469 -0.237648
C -0.000196 3.560768 -0.128955
C -1.190654 2.796626 -0.053854
C -0.953236 1.420492 -0.098954
C 0.327465 0.857039 -0.201346
C 1.456355 1.667110 -0.271850
O -1.899048 0.188847 -0.062705
C -1.046529 -0.979874 -0.132483
C 0.276323 -0.588191 -0.216095
C -1.486226 -2.281735 -0.115892
C -0.471605 -3.249418 -0.192214
C 0.889448 -2.917016 -0.277313
C 1.267217 -1.565229 -0.290430
C -2.939442 -2.610550 -0.025977
C -3.800977 -1.380452 0.089330
C -3.352643 -0.056446 0.068593
C -4.207331 1.023981 0.157716
C -5.578278 0.804619 0.283693
C -6.074779 -0.497350 0.317480
C -5.186927 -1.562707 0.219392
H -0.123496 4.638359 -0.096202
H 2.438558 1.213713 -0.350592
H -0.764702 -4.295568 -0.182054
H 2.312654 -1.285710 -0.349407
H -3.247496 -3.180437 -0.910728
H -3.123369 -3.260472 0.837050
H -3.839746 2.026452 0.130848
H -6.241594 1.660087 0.353528
H -7.139276 -0.681962 0.416830
H -5.556024 -2.583944 0.240092
C -2.455825 3.610978 0.065050
H -2.189159 4.668468 0.068680
H -3.129447 3.455393 -0.781476
H -2.987382 3.413398 0.999488
C 2.487122 3.968618 -0.327518
H 2.214105 5.000624 -0.092792
H 3.277196 3.649460 0.359356
H 2.911333 3.949583 -1.338290
C 1.942089 -3.994580 -0.342187
H 2.573874 -3.973534 0.552786
H 1.489728 -4.986791 -0.418391
H 2.601332 -3.846877 -1.203770
B 5.040162 -0.331646 0.307821
F 6.415292 -0.129694 0.551987
F 4.818429 -1.670058 -0.089014
F 4.303403 -0.060602 1.483201
F 4.610631 0.537943 -0.724358
47
10-BF4-TS_32 Eopt
-1349.622935
C -1.957267 -2.134659 -1.048111
C -2.202609 -0.763126 -1.174544
C -1.264411 0.272033 -0.957913
C -0.002510 -0.208878 -0.594228
C 0.299544 -1.569827 -0.455839
C -0.676242 -2.538114 -0.683565
O 1.335749 0.503793 -0.256326
C 2.286226 -0.541861 0.048016
C 1.677394 -1.775035 -0.070574
C 3.602467 -0.339922 0.400237
C 4.335108 -1.508373 0.645741
C 3.773832 -2.793963 0.543048
C 2.428084 -2.926197 0.179553
C 4.176348 1.034320 0.503823
C 3.172701 2.104410 0.163403
C 1.836421 1.897446 -0.190419
C 0.969266 2.931517 -0.478216
C 1.429536 4.246250 -0.423519
C 2.755143 4.505986 -0.080338
C 3.601116 3.440617 0.206638
H -3.203798 -0.447266 -1.446000
H -0.423055 -3.586852 -0.568385
H 5.380021 -1.405203 0.926565
H 1.971693 -3.906362 0.091647
H 4.550400 1.204937 1.520108
H 5.041692 1.128064 -0.162237
H -0.049784 2.745580 -0.739203
H 0.739731 5.052021 -0.650146
H 3.125886 5.524533 -0.034443
H 4.636439 3.624725 0.478356
C -1.806958 1.666106 -1.154082
H -2.861333 1.592742 -1.418328
H -1.753691 2.260556 -0.239073
H -1.303963 2.192490 -1.969906
C -3.063539 -3.130734 -1.273682
H -2.664104 -4.136867 -1.426687
H -3.732490 -3.158588 -0.405943
H -3.670618 -2.857834 -2.142137
C 4.619910 -4.008863 0.829854
H 4.947279 -4.017749 1.875818
H 4.064326 -4.930745 0.640123
H 5.521046 -4.015975 0.206976
B -5.144398 0.388411 0.925222
F -6.257069 0.881730 1.643919
F -5.114230 -1.021739 1.009621
F -5.252417 0.776667 -0.431511
F -3.955956 0.920074 1.475271
47
10-BF4-TS_37 Eopt
-1349.622948
C 1.930151 -2.132555 -0.866708
C 2.184785 -0.762156 -0.973739
C 1.240200 0.275980 -0.791705
C -0.036770 -0.202874 -0.487150
C -0.349536 -1.564797 -0.373421
C 0.631129 -2.534539 -0.564298
O -1.387570 0.511517 -0.203037
C -2.354566 -0.533467 0.045880
C -1.744564 -1.767576 -0.054257
C -3.684614 -0.329743 0.340280
C -4.431400 -1.497603 0.542831
C -3.869927 -2.784158 0.454622
C -2.508916 -2.917801 0.153546
C -4.256798 1.045942 0.433501
C -3.235881 2.115267 0.145824
C -1.885709 1.905973 -0.148729
C -1.002306 2.938749 -0.387408
C -1.459919 4.254818 -0.342812
C -2.799072 4.516944 -0.058904
C -3.661300 3.452720 0.180232
H 3.196513 -0.447638 -1.202787
H 0.368386 -3.583362 -0.471637
H -5.487301 -1.393075 0.778375
H -2.051341 -3.898672 0.081589
H -5.093545 1.149984 -0.266673
H -4.672150 1.208019 1.435090
H 0.027178 2.750330 -0.602005
H -0.757424 5.059692 -0.530317
H -3.167793 5.536572 -0.021632
H -4.707284 3.638686 0.406075
C 1.797220 1.668711 -0.956882
H 2.860614 1.591833 -1.181043
H 1.329435 2.203859 -1.787696
H 1.711140 2.256701 -0.039940
C 3.038980 -3.135746 -1.043877
H 2.665391 -4.060808 -1.492982
H 3.839517 -2.736697 -1.671819
H 3.479144 -3.391354 -0.072811
C -4.734878 -3.999437 0.675948
H -5.252996 -3.942705 1.639353
H -5.502681 -4.078581 -0.102388
H -4.139584 -4.915887 0.659938
B 5.356581 0.362345 0.746417
F 5.292996 0.743288 -0.616071
F 5.311020 -1.046521 0.841361
F 6.562325 0.836722 1.309640
F 4.259721 0.919103 1.442723
47
10-BF4-TS_38 Eopt
-1349.626993
C 3.858156 1.227666 -0.548131
C 2.961274 2.290922 -0.701359
C 1.553631 2.186833 -0.767324
C 1.096706 0.867599 -0.665347

C 1.953279 -0.231960 -0.525955
C 3.334546 -0.058135 -0.466378
O -0.315511 0.248217 -0.648882
C -0.128737 -1.183022 -0.565512
C 1.216885 -1.473365 -0.472379
C -1.137267 -2.118067 -0.598675
C -0.713925 -3.450510 -0.504426
C 0.639606 -3.810865 -0.382797
C 1.616575 -2.807241 -0.374215
C -2.565438 -1.707877 -0.724659
C -2.719293 -0.229922 -0.963747
C -1.701086 0.722871 -0.881468
C -1.940721 2.078769 -0.961291
C -3.236252 2.532563 -1.204263
C -4.278525 1.618831 -1.348905
C -4.009529 0.261093 -1.217330
H 3.369116 3.293960 -0.776809
H 3.976314 -0.925661 -0.354033
H -1.472026 -4.228903 -0.525237
H 2.668969 -3.058822 -0.295722
H -3.048088 -2.264959 -1.534508
H -3.103223 -1.966864 0.195696
H -1.159035 2.791718 -0.817101
H -3.413936 3.600926 -1.265429
H -5.291085 1.958554 -1.540118
H -4.815332 -0.463636 -1.289754
C 0.832275 3.499836 -0.943959
H 1.574654 4.294715 -1.028304
H 0.230721 3.523639 -1.855230
H 0.205781 3.740752 -0.080890
C 5.340157 1.485482 -0.466673
H 5.593005 1.987731 0.474453
H 5.906827 0.551997 -0.514116
H 5.673230 2.135281 -1.282704
C 1.026808 -5.261711 -0.245024
H 0.886630 -5.600839 0.788661
H 0.410024 -5.899907 -0.885286
H 2.076926 -5.417758 -0.506764
B -0.627051 0.519680 2.719137
F -1.654703 -0.265078 2.147570
F -0.570921 1.771172 2.063904
F -0.896189 0.718627 4.092124
F 0.610159 -0.144142 2.570568
47
10-BF4-TS_39 Eopt
-1349.623516
C -0.865836 3.881187 0.071113
C 0.368850 3.222500 0.099177
C 0.565061 1.822568 0.084610
C -0.637280 1.106772 0.043294
C -1.898573 1.715524 0.007759
C -2.018491 3.103928 0.022677
O -0.954838 -0.409271 0.020183
C -2.395360 -0.521105 -0.017867
C -2.961337 0.737849 -0.033950
C -3.100958 -1.704012 -0.030589
C -4.493985 -1.562465 -0.075890
C -5.130938 -0.308537 -0.103569
C -4.352415 0.854995 -0.080265
C -2.404243 -3.023050 0.008225
C -0.905660 -2.883074 0.054763
C -0.187661 -1.684076 0.058051
C 1.190872 -1.634637 0.087744
C 1.916803 -2.824611 0.124677
C 1.248043 -4.047232 0.130141
C -0.141711 -4.060233 0.093673
H 1.268736 3.828436 0.133819
H -3.004996 3.554053 -0.000831
H -5.097605 -2.466233 -0.089107
H -4.819722 1.833660 -0.098318
H -2.739613 -3.593439 0.882137
H -2.681453 -3.616413 -0.870792
H 1.719948 -0.706566 0.079750
H 2.998885 -2.768581 0.145077
H 1.801898 -4.979704 0.158818
H -0.679128 -5.004184 0.092452
C 2.008006 1.385404 0.109010
H 2.641706 2.271780 0.125655
H 2.251040 0.805274 1.001884
H 2.281621 0.818114 -0.783149
C -0.927394 5.386119 0.082910
H -1.957731 5.737925 0.178646
H -0.342999 5.795913 0.913376
H -0.510319 5.798574 -0.843042
C -6.636044 -0.229793 -0.150267
H -6.975282 0.806168 -0.229032
H -7.031268 -0.786429 -1.007016
H -7.078044 -0.666046 0.752839
B 5.517523 0.103595 -0.102796
F 4.665098 -1.023272 -0.183738
F 5.278824 0.786301 1.111683
F 6.863442 -0.321368 -0.149594
F 5.255628 0.968313 -1.189819
47
10-BF4-TS_4 Eopt
-1349.623999
C 1.189764 3.900118 0.000681
C -0.101182 3.359441 -0.003177
C -0.428042 1.984461 -0.005966
C 0.702512 1.158820 -0.002774
C 2.015776 1.646749 0.000102
C 2.265520 3.018270 0.001754
O 0.876631 -0.380120 -0.003293
C 2.300424 -0.627017 -0.000531
C 2.982537 0.573084 0.000681
C 2.891702 -1.871271 0.001091
C 4.292550 -1.860721 0.002637
C 5.045358 -0.672471 0.002796
C 4.379254 0.559033 0.002215
C 2.073062 -3.119253 0.002019
C 0.593153 -2.838448 0.000470
C -0.007889 -1.576694 -0.002411
C -1.375291 -1.395423 -0.006028
C -2.211986 -2.510736 -0.007310
C -1.663252 -3.791506 -0.003214
C -0.280540 -3.937356 0.000689
H -0.940817 4.047136 -0.004737
H 3.290589 3.373588 0.003823
H 4.809491 -2.816729 0.003760
H 4.935132 1.490550 0.002829
H 2.323877 -3.728557 -0.874013
H 2.322673 -3.726177 0.880050
H -1.812516 -0.420774 -0.008647
H -3.283870 -2.353174 -0.013900
H -2.304046 -4.667025 -0.003804
H 0.162792 -4.928934 0.003184
C -1.906251 1.685280 -0.013848
H -2.453503 2.628042 -0.020667
H -2.211273 1.135645 -0.906677
H -2.223932 1.141384 0.878059
C 1.387996 5.393376 0.002911
H 2.450103 5.650911 0.001814
H 0.922476 5.849701 -0.877668
H 0.925180 5.846597 0.886547
C 6.551882 -0.737993 0.003590
H 6.991121 0.262789 0.005106
H 6.919644 -1.274486 0.885380
H 6.920505 -1.272079 -0.879326
B -5.341079 0.165824 0.005165
F -6.670855 -0.309391 0.044768
F -5.343986 1.574320 -0.108019
F -4.670595 -0.210646 1.192416
F -4.673855 -0.394843 -1.109039
47
10-BF4-TS_40 Eopt
-1349.622669
C -1.697513 -2.386878 -0.402427
C -2.075406 -1.040461 -0.465907
C -1.204846 0.070184 -0.390322
C 0.135977 -0.302079 -0.241047
C 0.569667 -1.632056 -0.174679
C -0.345792 -2.680736 -0.255460
O 1.447969 0.521746 -0.110455
C 2.518284 -0.439690 0.023996
C 2.003504 -1.719851 -0.016314
C 3.851819 -0.127787 0.170022
C 4.709633 -1.230093 0.279598
C 4.248348 -2.558483 0.245603
C 2.877808 -2.803261 0.095364
C 4.316691 1.290334 0.206911
C 3.183736 2.272782 0.066833
C 1.831565 1.953713 -0.085596
C 0.846589 2.911234 -0.217704
C 1.196516 4.260515 -0.196299
C 2.531346 4.631373 -0.043928
C 3.498614 3.640911 0.083811
H -3.130594 -0.815114 -0.573540
H 0.009526 -3.704362 -0.199103
H 5.773217 -1.038914 0.396065
H 2.496160 -3.818231 0.066101
H 4.845375 1.483924 1.147525
H 5.043442 1.464552 -0.594995
H -0.180086 2.641207 -0.338522
H 0.414729 5.005206 -0.300304
H 2.816598 5.677920 -0.025552
H 4.543764 3.912145 0.201452
C -1.887849 1.412367 -0.482260
H -2.959600 1.251293 -0.591925
H -1.749010 2.007837 0.423734
H -1.557775 1.983181 -1.354151
C -2.741348 -3.469156 -0.486279
H -3.307129 -3.393779 -1.421134
H -2.286582 -4.461794 -0.435833
H -3.462039 -3.375960 0.333276
C 5.226365 -3.698369 0.380517
H 5.688300 -3.699101 1.374622
H 4.732808 -4.662727 0.235703
H 6.034662 -3.611712 -0.353590
B -5.780136 0.313905 0.351133
F -7.128443 0.272993 0.766487
F -5.514025 -0.785010 -0.500348
F -5.536327 1.517599 -0.350463
F -4.933546 0.248181 1.481161
47
10-BF4-TS_41 Eopt
-1349.622666
C -1.696560 -2.388933 -0.390702
C -2.074728 -1.042939 -0.461036

C -1.204093 0.068222 -0.394318
C 0.136964 -0.303070 -0.244758
C 0.571109 -1.632606 -0.173228
C -0.344378 -2.681832 -0.246013
O 1.449499 0.521628 -0.125389
C 2.520157 -0.438789 0.013897
C 2.005531 -1.719453 -0.019326
C 3.852419 -0.125738 0.166748
C 4.710355 -1.227432 0.285143
C 4.250072 -2.555914 0.252095
C 2.879560 -2.801806 0.100960
C 4.315765 1.292751 0.207822
C 3.181431 2.274329 0.072516
C 1.830700 1.953993 -0.089766
C 0.845596 2.910648 -0.226968
C 1.193146 4.260349 -0.195494
C 2.525969 4.632494 -0.029305
C 3.493907 3.642856 0.099817
H -3.130166 -0.818341 -0.567716
H 0.011346 -3.705098 -0.185691
H 5.772703 -1.035143 0.410220
H 2.497888 -3.816991 0.079833
H 4.845992 1.483410 1.148094
H 5.040801 1.470899 -0.594865
H -0.179311 2.639616 -0.360151
H 0.411177 5.004341 -0.303042
H 2.809323 5.679383 -0.002613
H 4.537831 3.915066 0.225772
C -1.887343 1.409722 -0.494031
H -2.958975 1.247800 -0.603711
H -1.749325 2.010219 0.408727
H -1.556676 1.975768 -1.368807
C -2.740527 -3.471702 -0.466210
H -3.460672 -3.372719 0.353130
H -3.306941 -3.402808 -1.401195
H -2.285827 -4.464012 -0.409190
C 5.229609 -3.696899 0.363984
H 4.714588 -4.644022 0.544391
H 5.811283 -3.800207 -0.559881
H 5.940381 -3.528605 1.179471
B -5.780572 0.313566 0.348486
F -7.129130 0.272542 0.763004
F -5.512636 -0.787931 -0.499096
F -5.537538 1.515173 -0.356925
F -4.934754 0.252559 1.479346
47
10-BF4-TS_42 Eopt
-1349.622513
C -1.818682 -2.172013 0.061177
C -2.112041 -0.803270 0.064611
C -1.168381 0.248740 0.044515
C 0.153339 -0.210117 0.020283
C 0.503449 -1.565950 0.017738
C -0.480841 -2.552928 0.037020
O 1.523098 0.525916 -0.020290
C 2.535960 -0.505547 -0.024798
C 1.936397 -1.748869 -0.006243
C 3.895098 -0.284352 -0.036110
C 4.683965 -1.442347 -0.033386
C 4.134515 -2.736854 -0.020231
C 2.742329 -2.889014 -0.003966
C 4.455559 1.098977 -0.045775
C 3.382819 2.155192 -0.005371
C 2.003392 1.928938 -0.001505
C 1.077765 2.952758 0.008962
C 1.519292 4.274973 0.029007
C 2.884839 4.553357 0.036107
C 3.790158 3.498470 0.017121
H -3.154504 -0.508381 0.086112
H -0.188034 -3.597705 0.032632
H 5.764052 -1.322831 -0.039447
H 2.292889 -3.876133 0.013044
H 5.127864 1.234321 0.809043
H 5.067302 1.245834 -0.943779
H 0.027554 2.756035 0.000413
H 0.783444 5.071897 0.038170
H 3.241157 5.577886 0.053203
H 4.858142 3.696549 0.016576
C -1.769100 1.632501 0.055804
H -2.855072 1.541515 0.081290
H -1.474877 2.202305 0.941000
H -1.518600 2.196633 -0.846598
C -2.931702 -3.185893 0.092788
H -3.662023 -2.989753 -0.698612
H -2.544630 -4.200490 -0.032010
H -3.471011 -3.138210 1.045441
C 5.041840 -3.941054 -0.037166
H 4.486165 -4.857511 0.177586
H 5.515674 -4.056152 -1.019312
H 5.843589 -3.841464 0.701801
B -5.931669 0.312655 -0.046101
F -5.331029 1.587410 0.080919
F -5.497368 -0.516897 1.013785
F -7.336146 0.444413 0.001604
F -5.549382 -0.263721 -1.279332
47
10-BF4-TS_43 Eopt
-1349.622520
C 1.816501 -2.174054 0.124449
C 2.110494 -0.805461 0.136378
C 1.167924 0.247040 0.100512
C -0.153484 -0.210956 0.052405
C -0.504226 -1.566498 0.040815
C 0.479124 -2.554062 0.074924
O -1.522108 0.525982 -0.010519
C -2.535180 -0.504979 -0.031278
C -1.936678 -1.748441 -0.005982
C -3.894080 -0.282980 -0.064651
C -4.683297 -1.440352 -0.078415
C -4.134606 -2.735433 -0.058768
C -2.743287 -2.888340 -0.020517
C -4.453215 1.100864 -0.081579
C -3.380826 2.156288 -0.017403
C -2.001777 1.929140 0.007447
C -1.075627 2.952089 0.037321
C -1.516585 4.274504 0.056951
C -2.881924 4.553814 0.044160
C -3.787601 3.499744 0.005633
H 3.152644 -0.511145 0.176470
H 0.185778 -3.598590 0.062839
H -5.763124 -1.320482 -0.104208
H -2.294816 -3.875827 -0.000513
H -5.046799 1.250614 -0.991267
H -5.142377 1.234509 0.759924
H -0.025611 2.754358 0.044859
H -0.780493 5.070874 0.081648
H -3.237767 5.578516 0.060905
H -4.855321 3.698625 -0.010713
C 1.768560 1.630577 0.123236
H 2.854058 1.539555 0.162754
H 1.530560 2.196130 -0.781642
H 1.461179 2.198832 1.004982
C 2.928365 -3.188515 0.172673
H 3.661174 -3.002830 -0.619081
H 3.465016 -3.129276 1.126112
H 2.540786 -4.204215 0.058959
C -5.043664 -3.938355 -0.077647
H -4.469240 -4.867693 -0.047566
H -5.724483 -3.928548 0.780825
H -5.661488 -3.947398 -0.982621
B 5.930952 0.312461 -0.096575
F 7.330958 0.488909 -0.123633
F 5.579168 -0.530182 0.983468
F 5.298359 1.568046 0.064637
F 5.502955 -0.275660 -1.308876
47
10-BF4-TS_45 Eopt
-1349.623733
C 5.298349 0.527243 0.189970
C 5.024121 -0.845184 0.181811
C 3.743145 -1.435554 0.095766
C 2.709975 -0.493030 0.014461
C 2.931698 0.891118 0.017372
C 4.223852 1.406452 0.105281
O 1.177660 -0.627587 -0.098773
C 0.652320 0.722086 -0.135180
C 1.691967 1.627279 -0.073958
C -0.682422 1.052186 -0.210631
C -0.950076 2.427247 -0.231404
C 0.064197 3.400673 -0.179170
C 1.401545 2.993499 -0.098866
C -1.740464 0.003191 -0.261582
C -1.174788 -1.389879 -0.205023
C 0.177830 -1.731613 -0.137012
C 0.622811 -3.037776 -0.111716
C -0.307699 -4.075768 -0.143292
C -1.670344 -3.787862 -0.200599
C -2.083357 -2.460945 -0.231777
H 5.862356 -1.531630 0.248081
H 4.369854 2.481363 0.106750
H -1.988773 2.741140 -0.288789
H 2.202226 3.724180 -0.053479
H -2.446056 0.141389 0.563618
H -2.329782 0.112702 -1.178790
H 1.663469 -3.273196 -0.067929
H 0.049758 -5.099717 -0.121745
H -2.404619 -4.586384 -0.222722
H -3.139403 -2.212865 -0.278790
C 3.743309 -2.944562 0.112149
H 4.773795 -3.289881 0.203927
H 3.193148 -3.347368 0.965835
H 3.347196 -3.367426 -0.814926
C 6.717240 1.020175 0.300936
H 6.772925 2.099569 0.139064
H 7.125897 0.803841 1.294930
H 7.363680 0.526027 -0.431792
C -0.298489 4.863960 -0.204450
H -0.867413 5.110305 -1.107783
H -0.926271 5.124984 0.654913
H 0.594720 5.493514 -0.179460
B -5.446201 0.268494 0.238977
F -6.821723 0.404494 0.523832
F -5.200425 -1.015133 -0.304242
F -5.054561 1.256292 -0.693468
F -4.698325 0.424353 1.430085
47
10-BF4-TS_46 Eopt
-1349.623827

C -1.526110 3.891111 -0.004783
C -0.195107 3.457443 -0.009183
C 0.240819 2.113033 -0.010024
C -0.818698 1.198200 -0.006307
C -2.166885 1.578176 -0.001717
C -2.526714 2.924767 -0.001009
O -0.866183 -0.349072 -0.005227
C -2.265452 -0.710829 0.000515
C -3.042965 0.429761 0.002460
C -2.754346 -1.998632 0.003687
C -4.151437 -2.101975 0.009170
C -4.998144 -0.978644 0.011333
C -4.433768 0.302620 0.007954
C -1.837737 -3.175978 0.001131
C -0.385601 -2.777468 -0.006261
C 0.113389 -1.472025 -0.009436
C 1.462765 -1.183785 -0.015708
C 2.385041 -2.229706 -0.018764
C 1.938651 -3.549729 -0.016260
C 0.571927 -3.804214 -0.010225
H 0.586234 4.211123 -0.012079
H -3.577409 3.195390 0.002801
H -4.588601 -3.097035 0.011802
H -5.063152 1.186311 0.009442
H -2.034328 -3.800972 0.879982
H -2.042586 -3.803654 -0.873899
H 1.822688 -0.178044 -0.017711
H 3.442468 -1.992229 -0.022528
H 2.646966 -4.371602 -0.018727
H 0.207208 -4.827382 -0.008046
C 1.736702 1.928621 -0.014662
H 2.209874 2.911029 -0.014785
H 2.093350 1.405869 0.874711
H 2.086568 1.408629 -0.908570
C -1.847430 5.362723 -0.003308
H -1.425805 5.852916 0.881229
H -1.419978 5.856314 -0.883071
H -2.927355 5.530613 -0.006352
C -6.494645 -1.164319 0.017046
H -7.012753 -0.201928 0.019107
H -6.822145 -1.727369 -0.864096
H -6.815426 -1.727613 0.900488
B 5.404649 0.214340 0.013183
F 5.776800 -1.149808 0.053646
F 6.562498 1.022230 0.026017
F 4.667619 0.463194 -1.167740
F 4.605847 0.515005 1.140557
47
10-BF4-TS_47 Eopt
-1349.622722
C -1.871093 3.872708 0.000674
C -0.516329 3.521061 0.005056
C 0.000821 2.205741 0.007685
C -1.000774 1.227308 0.004915
C -2.370007 1.524506 -0.000736
C -2.810815 2.847018 -0.003255
O -0.953719 -0.319416 0.004752
C -2.328383 -0.766618 -0.000737
C -3.174415 0.324203 -0.004564
C -2.736726 -2.082379 -0.001126
C -4.124732 -2.271917 -0.006869
C -5.038989 -1.202914 -0.011427
C -4.554887 0.110789 -0.009951
C -1.749618 -3.201436 0.005204
C -0.324698 -2.713960 0.008430
C 0.092770 -1.380386 0.007714
C 1.421669 -1.009676 0.009699
C 2.407597 -1.995991 0.014121
C 2.043180 -3.341080 0.015501
C 0.694917 -3.679337 0.012299
H 0.216970 4.321544 0.005881
H -3.875814 3.053907 -0.009087
H -4.499519 -3.292061 -0.007577
H -5.238302 0.953492 -0.012802
H -1.914235 -3.836334 0.883530
H -1.908335 -3.841503 -0.870415
H 1.719872 0.016126 0.008019
H 3.448046 -1.692388 0.016923
H 2.800989 -4.117601 0.018890
H 0.394248 -4.723065 0.012856
C 1.506009 2.114312 0.011587
H 1.917017 3.124328 0.014273
H 1.887192 1.614574 0.904544
H 1.891791 1.617465 -0.881040
C -2.281821 5.321852 0.009912
H -2.007626 5.796428 0.959213
H -1.779052 5.877395 -0.788641
H -3.361580 5.426412 -0.123138
C -6.520967 -1.481454 -0.017847
H -7.098303 -0.553512 -0.016246
H -6.806059 -2.060151 -0.903600
H -6.812487 -2.067462 0.860931
B 5.808860 0.227862 -0.011041
F 6.504833 0.751610 1.100861
F 6.462505 0.608396 -1.204021
F 5.772100 -1.184272 0.077321
F 4.486605 0.729192 -0.017834
47
10-BF4-TS_50 Eopt
-1349.623806
C -1.534436 3.889887 -0.062817
C -0.203441 3.459803 -0.117461
C 0.236203 2.116644 -0.124282
C -0.819684 1.198940 -0.074398
C -2.167675 1.575293 -0.014789
C -2.531116 2.920991 -0.008890
O -0.862042 -0.347915 -0.060126
C -2.258580 -0.714032 0.009404
C -3.038938 0.424262 0.038421
C -2.742575 -2.003406 0.039360
C -4.137569 -2.111088 0.110447
C -4.986900 -0.990264 0.146296
C -4.427637 0.292699 0.108213
C -1.823412 -3.177956 -0.007167
C -0.374902 -2.774503 -0.089690
C 0.119486 -1.467588 -0.112073
C 1.466416 -1.174846 -0.173916
C 2.391037 -2.217631 -0.222299
C 1.948840 -3.539063 -0.210427
C 0.584579 -3.797934 -0.143971
H 0.574838 4.215657 -0.156381
H -3.581210 3.189438 0.040441
H -4.570559 -3.107611 0.137326
H -5.059571 1.174259 0.130510
H -1.971697 -3.801250 0.882355
H -2.071086 -3.808639 -0.868798
H 1.822738 -0.167933 -0.180783
H 3.447044 -1.976967 -0.263029
H 2.658749 -4.358672 -0.248510
H 0.223186 -4.822144 -0.128990
C 1.731727 1.936742 -0.180152
H 2.201416 2.920678 -0.196087
H 2.120970 1.415328 0.696253
H 2.051895 1.418274 -1.086136
C -1.859820 5.360595 -0.061976
H -2.937957 5.525927 0.006720
H -1.379239 5.866234 0.782731
H -1.496396 5.840644 -0.977449
C -6.480834 -1.180401 0.223914
H -7.002064 -0.219757 0.232543
H -6.846487 -1.760709 -0.630545
H -6.758430 -1.728247 1.131449
B 5.389892 0.216383 0.164858
F 5.709621 -1.156922 0.050063
F 4.517209 0.403290 1.261651
F 6.570721 0.962942 0.368626
F 4.755352 0.648916 -1.022272
47
10-BF4-TS_51 Eopt
-1349.621643
C -3.803125 3.252903 0.060848
C -2.440027 3.558140 -0.029622
C -1.380548 2.624855 -0.091725
C -1.821648 1.295969 -0.051343
C -3.173457 0.937364 0.035159
C -4.168711 1.911477 0.093018
O -1.077756 -0.058088 -0.091115
C -2.095334 -1.082886 -0.020283
C -3.342793 -0.496887 0.052280
C -1.859816 -2.440057 -0.023387
C -3.007857 -3.239842 0.048715
C -4.306438 -2.703886 0.121389
C -4.473155 -1.313775 0.123378
C -0.473423 -2.986965 -0.096194
C 0.571650 -1.904370 -0.151686
C 0.335833 -0.527430 -0.154738
C 1.348704 0.407655 -0.215849
C 2.673381 -0.022880 -0.272669
C 2.963757 -1.386017 -0.268515
C 1.917954 -2.299621 -0.209402
H -2.154234 4.604850 -0.057320
H -5.208141 1.608074 0.161200
H -2.877251 -4.318712 0.048555
H -5.463399 -0.874497 0.179814
H -0.280804 -3.627667 0.772032
H -0.373010 -3.628730 -0.979231
H 1.144148 1.455841 -0.220045
H 3.467265 0.713437 -0.319979
H 3.992653 -1.725232 -0.308450
H 2.125126 -3.366013 -0.206323
C -0.005830 3.238713 -0.189190
H -0.110413 4.324280 -0.201312
H 0.619619 2.989838 0.671808
H 0.508731 2.957250 -1.111213
C -4.828747 4.354718 0.115056
H -4.801512 4.957327 -0.799774
H -5.837285 3.949113 0.227246
H -4.633045 5.029854 0.955215
C -5.497639 -3.624868 0.202176
H -5.515385 -4.318443 -0.645662
H -5.462833 -4.229047 1.115864
H -6.433689 -3.060546 0.202747
B 6.516483 0.285646 0.166281
F 7.886540 0.607314 0.282465
F 5.891565 0.410392 1.428045
F 5.899855 1.168567 -0.751960
F 6.381676 -1.043869 -0.297679
47

10-BF4-TS_52 Eopt
-1349.622102
C 5.496847 -0.737848 -0.058945
C 4.785985 -1.943524 -0.045426
C 3.379562 -2.079217 -0.017640
C 2.712239 -0.847376 -0.003220
C 3.376993 0.386696 -0.014106
C 4.769191 0.447429 -0.041622
O 1.218266 -0.467576 0.029294
C 1.164351 0.978481 0.030181
C 2.445530 1.490956 0.006477
C 0.010724 1.730904 0.049002
C 0.211173 3.117442 0.046128
C 1.490415 3.702569 0.024566
C 2.620815 2.876774 0.003548
C -1.335652 1.088230 0.068464
C -1.256794 -0.415160 0.068749
C -0.090059 -1.182514 0.050628
C -0.097952 -2.562623 0.055178
C -1.318256 -3.237399 0.078668
C -2.511507 -2.517245 0.097465
C -2.466088 -1.128147 0.092178
H 5.354369 -2.867994 -0.056499
H 5.260008 1.414702 -0.048845
H -0.666543 3.758237 0.060562
H 3.618624 3.302186 -0.014762
H -1.894662 1.417808 0.951547
H -1.918866 1.416531 -0.799357
H 0.808980 -3.125897 0.042358
H -1.315866 -4.322278 0.082323
H -3.469921 -3.024815 0.116331
H -3.386055 -0.553403 0.106920
C 2.884703 -3.505029 -0.008374
H 3.747737 -4.171720 -0.031435
H 2.324052 -3.742146 0.899657
H 2.278851 -3.738830 -0.887476
C 7.002390 -0.741923 -0.104353
H 7.417409 -1.401065 0.665147
H 7.359224 -1.107913 -1.074177
H 7.404166 0.263006 0.047217
C 1.629491 5.203918 0.024085
H 1.128139 5.642755 -0.845758
H 1.168820 5.639273 0.917901
H 2.680200 5.504024 0.000524
B -6.273569 0.064888 -0.067842
F -6.734763 0.166884 -1.399029
F -5.876754 -1.266634 0.193609
F -5.162816 0.926261 0.111281
F -7.303617 0.435859 0.823349
47
10-BF4-TS_53 Eopt
-1349.623212
C -5.312439 -0.082204 0.472186
C -4.828200 -1.392102 0.408391
C -3.481857 -1.771513 0.192944
C -2.623486 -0.677407 0.038774
C -3.060361 0.655315 0.087911
C -4.401921 0.958012 0.303511
O -1.106391 -0.564924 -0.215305
C -0.807312 0.850630 -0.279250
C -1.966229 1.579084 -0.105571
C 0.445143 1.388704 -0.474981
C 0.488039 2.788887 -0.497096
C -0.657197 3.589082 -0.332089
C -1.899255 2.974056 -0.133383
C 1.646839 0.521623 -0.641175
C 1.315368 -0.944284 -0.562144
C 0.046767 -1.496249 -0.369441
C -0.182781 -2.856184 -0.321271
C 0.891476 -3.734349 -0.458983
C 2.179340 -3.234527 -0.644155
C 2.372956 -1.859036 -0.694607
H -5.538328 -2.203278 0.532330
H -4.715666 1.996109 0.337852
H 1.453541 3.265194 -0.645458
H -2.797758 3.567567 -0.000171
H 2.125964 0.728609 -1.605040
H 2.393175 0.760486 0.123415
H -1.165244 -3.251327 -0.182048
H 0.703216 -4.801909 -0.418041
H 3.025317 -3.905903 -0.746907
H 3.368591 -1.450967 -0.834872
C -3.246305 -3.261975 0.171937
H -4.200309 -3.765869 0.331156
H -2.861392 -3.605846 -0.791443
H -2.577410 -3.586312 0.973297
C -6.769069 0.190606 0.741175
H -6.933740 0.370932 1.810565
H -7.109467 1.079389 0.201896
H -7.393670 -0.657239 0.447397
C -0.534503 5.091593 -0.367402
H 0.153426 5.445527 0.408427
H -0.137329 5.428737 -1.331428
H -1.503843 5.571862 -0.210408
B 5.487361 0.191401 0.588942
F 6.637702 0.958771 0.871635
F 5.182457 0.286755 -0.789794
F 5.721438 -1.160488 0.925059
F 4.395644 0.682746 1.344824
47
10-BF4-TS_54 Eopt
-1349.623726
C -5.298352 0.465425 -0.248852
C -5.001972 -0.902387 -0.237339
C -3.713664 -1.472180 -0.125960
C -2.697521 -0.513064 -0.024565
C -2.941569 0.867271 -0.031201
C -4.239992 1.361901 -0.143812
O -1.164389 -0.622469 0.110741
C -0.662691 0.735476 0.162541
C -1.715813 1.623481 0.082465
C 0.664360 1.087561 0.268707
C 0.908477 2.466870 0.298352
C -0.120376 3.423457 0.225334
C -1.448724 2.994200 0.115772
C 1.738610 0.056122 0.338544
C 1.196925 -1.346280 0.274206
C -0.147804 -1.710221 0.173411
C -0.570277 -3.023554 0.133576
C 0.375941 -4.046196 0.188793
C 1.731647 -3.735934 0.282145
C 2.121910 -2.402445 0.323740
H -5.827585 -1.601819 -0.323784
H -4.402489 2.434587 -0.149259
H 1.940291 2.797923 0.379046
H -2.260299 3.711641 0.054937
H 2.453399 0.203486 -0.477028
H 2.312565 0.176784 1.264071
H -1.605438 -3.275561 0.060491
H 0.036064 -5.075820 0.156183
H 2.478284 -4.522171 0.322681
H 3.172421 -2.137478 0.395100
C -3.688356 -2.981006 -0.142137
H -4.711347 -3.344323 -0.247241
H -3.120387 -3.373944 -0.988934
H -3.296901 -3.397066 0.789892
C -6.725379 0.934360 -0.360231
H -7.280673 0.705324 0.556925
H -6.773953 2.013648 -0.525158
H -7.240515 0.433801 -1.186580
C 0.217476 4.892501 0.259788
H -0.685651 5.507314 0.227450
H 0.772855 5.145004 1.169770
H 0.849594 5.167021 -0.592216
B 5.431921 0.263473 -0.308700
F 5.218845 -0.934171 0.414451
F 5.075489 1.368272 0.497990
F 6.791970 0.359759 -0.671642
F 4.630156 0.255240 -1.475072
47
10-BF4-TS_6 Eopt
-1349.623688
C -5.251093 -0.745547 0.088454
C -5.056256 0.640679 0.093720
C -3.809560 1.304637 0.052861
C -2.721697 0.423578 0.001525
C -2.862896 -0.970874 0.000405
C -4.125145 -1.560746 0.043781
O -1.196934 0.646386 -0.068311
C -0.593536 -0.670876 -0.079189
C -1.580567 -1.634214 -0.044522
C 0.759546 -0.923523 -0.112072
C 1.107060 -2.280638 -0.112238
C 0.149229 -3.310707 -0.082502
C -1.211283 -2.981252 -0.048292
C 1.755497 0.185042 -0.144671
C 1.109559 1.542728 -0.095416
C -0.262080 1.805818 -0.069709
C -0.782265 3.084052 -0.056659
C 0.087495 4.173944 -0.054233
C 1.465705 3.965315 -0.066570
C 1.954958 2.664380 -0.088504
H -5.933933 1.278186 0.133021
H -4.207885 -2.642451 0.041212
H 2.163306 -2.534004 -0.135720
H -1.968847 -3.757125 -0.022331
H 2.456035 0.084878 0.689911
H 2.363153 0.111975 -1.053050
H -1.835645 3.258905 -0.049185
H -0.328668 5.175670 -0.042940
H 2.152876 4.804949 -0.062422
H 3.024748 2.479082 -0.104714
C -3.898589 2.810908 0.077007
H -4.949616 3.094013 0.145135
H -3.394890 3.242044 0.945297
H -3.506283 3.261988 -0.838562
C -6.643056 -1.319657 0.131701
H -7.171521 -0.995525 1.035090
H -7.231251 -0.981478 -0.728622
H -6.618919 -2.412224 0.121885
C 0.595917 -4.750761 -0.082353
H -0.260095 -5.430509 -0.077520
H 1.205290 -4.971387 -0.965687
H 1.211427 -4.967980 0.797819
B 5.398915 -0.292684 0.111918
F 5.142552 1.010566 -0.374883
F 4.776607 -0.453556 1.372556
F 4.877025 -1.244713 -0.793970

F 6.791590 -0.481231 0.245536
47
10-BF4-TS_7 Eopt
-1349.623793
C -5.069194 -1.265556 0.174802
C -5.068500 0.134054 0.197645
C -3.927438 0.964793 0.135041
C -2.729894 0.243684 0.042672
C -2.675968 -1.156708 0.016446
C -3.843349 -1.915963 0.081984
O -1.252370 0.677424 -0.041093
C -0.473602 -0.542171 -0.124192
C -1.316001 -1.634227 -0.084924
C 0.897962 -0.601388 -0.230488
C 1.430377 -1.895794 -0.293757
C 0.626601 -3.049770 -0.252358
C -0.763328 -2.915850 -0.147823
C 1.728058 0.636943 -0.272079
C 0.897172 1.890066 -0.216988
C -0.493685 1.955830 -0.102543
C -1.187227 3.147086 -0.035845
C -0.484754 4.350054 -0.092774
C 0.903846 4.339984 -0.214076
C 1.570351 3.121461 -0.272179
H -6.025109 0.641813 0.269417
H -3.777374 -2.998586 0.058417
H 2.508737 -1.995043 -0.376631
H -1.403762 -3.790822 -0.115603
H 2.436131 0.635937 0.562917
H 2.338324 0.647475 -1.181529
H -2.250268 3.167711 0.062250
H -1.037289 5.282050 -0.040088
H 1.462705 5.268793 -0.259179
H 2.652302 3.093753 -0.360532
C -4.222073 2.444183 0.177042
H -5.301360 2.581212 0.255386
H -3.775933 2.929427 1.048965
H -3.901109 2.956326 -0.733674
C -6.366784 -2.027291 0.248055
H -7.016470 -1.772916 -0.596810
H -6.191954 -3.105980 0.232904
H -6.913340 -1.780252 1.164900
C 1.269880 -4.411612 -0.324425
H 2.003856 -4.539164 0.478697
H 0.525045 -5.207242 -0.239374
H 1.803404 -4.541038 -1.272847
B 5.310987 -0.335770 0.266655
F 4.765159 -1.189577 -0.720880
F 5.317346 0.993752 -0.214505
F 4.521160 -0.407837 1.438205
F 6.631090 -0.739099 0.561900
47
10-BF4-TS_9 Eopt
-1349.623619
C -5.176302 -1.030154 0.001815
C -5.088166 0.363891 0.003257
C -3.892023 1.122040 0.004090
C -2.741742 0.327779 0.002176
C -2.775344 -1.076094 -0.001094
C -3.987908 -1.758319 -0.001836
O -1.237030 0.666434 0.003729
C -0.533648 -0.601043 -0.000290
C -1.443645 -1.638100 -0.003327
C 0.835254 -0.747688 -0.003469
C 1.288318 -2.073576 -0.010121
C 0.412858 -3.174780 -0.012123
C -0.969719 -2.952449 -0.009232
C 1.742620 0.435328 -0.002001
C 0.992110 1.739227 -0.004088
C -0.396167 1.894453 -0.001948
C -1.014778 3.128311 -0.003584
C -0.233661 4.283153 -0.008000
C 1.156774 4.183291 -0.010334
C 1.746037 2.924318 -0.008200
H -6.011669 0.933256 0.003386
H -3.988312 -2.843621 -0.005089
H 2.361699 -2.239921 -0.015419
H -1.666128 -3.784529 -0.012733
H 2.402529 0.400774 0.871083
H 2.404719 0.399906 -0.873301
H -2.078777 3.218502 -0.001495
H -0.727163 5.249108 -0.009454
H 1.775930 5.074205 -0.013620
H 2.827470 2.825918 -0.009848
C -4.098837 2.616795 0.005693
H -5.170993 2.817417 0.009512
H -3.683401 3.091308 0.898435
H -3.689698 3.092095 -0.889636
C -6.513121 -1.723974 0.013768
H -6.580468 -2.456442 -0.797669
H -6.658669 -2.266478 0.955076
H -7.333047 -1.010011 -0.096845
C 0.971152 -4.575415 -0.009795
H 1.683781 -4.714187 -0.829763
H 1.509158 -4.777559 0.923570
H 0.176927 -5.319385 -0.113126
B 5.383679 -0.317708 0.007902
F 4.971654 0.284386 1.219615
F 6.779595 -0.525399 0.034785
F 4.723664 -1.559526 -0.147611
F 5.052150 0.529956 -1.074629
47
6-A-2Me-1 Eopt
-891.105543
C 4.431022 -0.763033 -0.218367
C 4.022320 -2.077327 0.039748
C 2.677108 -2.392936 0.265605
C 1.730772 -1.364193 0.226237
C 2.107723 -0.036506 -0.026231
C 3.464458 0.247005 -0.244607
H 4.766640 -2.870705 0.064985
H 0.686867 -1.592968 0.420759
H 3.766337 1.266611 -0.466854
C 5.881314 -0.451569 -0.503257
H 6.548460 -1.050869 0.124558
H 6.132469 -0.670547 -1.548491
H 6.104427 0.604499 -0.325541
C 2.261380 -3.809306 0.585599
H 2.868959 -4.536502 0.037773
H 2.382018 -4.021873 1.655166
H 1.211329 -3.982457 0.332499
C 1.089497 1.042019 -0.060766
C -0.171438 0.812784 -0.623262
C 1.363734 2.309734 0.482204
C -1.147718 1.815347 -0.646005
H -0.397107 -0.157305 -1.054425
C 0.408435 3.327540 0.470435
H 2.327532 2.490293 0.949408
C -0.848309 3.063117 -0.095151
H -1.605687 3.844427 -0.100881
C 0.709951 4.684964 1.060003
H 1.677323 4.690870 1.569852
H 0.737648 5.456233 0.281049
H -0.055822 4.983539 1.784487
C -2.503734 1.546117 -1.275144
H -2.409385 1.567474 -2.367945
H -3.185055 2.363247 -1.009506
C -3.108000 0.220070 -0.859603
C -3.369897 -0.038162 0.494027
C -3.389916 -0.774114 -1.799395
C -3.907825 -1.254761 0.921037
H -3.132535 0.726160 1.230530
C -3.926035 -2.000222 -1.390847
H -3.185415 -0.593623 -2.851351
C -4.182047 -2.239281 -0.042551
H -4.138190 -2.769661 -2.127617
H -4.594908 -3.195154 0.269958
C -4.202001 -1.505732 2.380771
H -5.281965 -1.500455 2.571882
H -3.820656 -2.481118 2.701571
H -3.748054 -0.739439 3.015254
47
6-A-2Me-10 Eopt
-891.104883
C 5.166860 0.335442 0.163073
C 5.326207 -1.040063 -0.030808
C 4.221635 -1.890181 -0.182996
C 2.940041 -1.337454 -0.137183
C 2.744693 0.040041 0.059031
C 3.869959 0.861466 0.209309
H 6.329353 -1.460314 -0.063098
H 2.077254 -1.981883 -0.278581
H 3.732729 1.923680 0.391495
C 6.366540 1.242499 0.302429
H 7.246188 0.691070 0.647171
H 6.622957 1.704593 -0.659038
H 6.172737 2.052814 1.012226
C 4.421832 -3.372955 -0.389422
H 5.091913 -3.570371 -1.233508
H 4.870523 -3.837684 0.496514
H 3.472613 -3.878562 -0.587239
C 1.376152 0.610981 0.107561
C 0.333475 -0.078181 0.750329
C 1.091409 1.845103 -0.491737
C -0.958914 0.445279 0.795307
H 0.542312 -1.025841 1.238406
C -0.197502 2.393026 -0.459017
H 1.880184 2.373551 -1.019862
C -1.213284 1.679350 0.182686
H -2.224200 2.079667 0.195512
C -0.477853 3.733330 -1.096147
H -0.259619 4.552430 -0.399555
H 0.139776 3.888275 -1.986232
H -1.527837 3.822372 -1.389915
C -2.079111 -0.305424 1.489763
H -2.438999 0.278733 2.345093
H -1.678320 -1.239890 1.900478
C -3.251437 -0.614308 0.577650
C -4.546432 -0.209745 0.910901
C -3.050360 -1.308879 -0.624176
C -5.645332 -0.486098 0.082512
H -4.707084 0.336883 1.838205
C -4.128747 -1.591925 -1.458914
H -2.045784 -1.610199 -0.907146
C -5.419705 -1.182382 -1.109123
H -3.966288 -2.127336 -2.389980
H -6.254851 -1.402004 -1.768988
C -7.035617 -0.056955 0.486667

H -7.720839 -0.073285 -0.365693
H -7.446193 -0.724295 1.254455
H -7.035580 0.955793 0.903113
47
6-A-2Me-11 Eopt
-891.104877
C 4.071059 -1.024967 0.930858
C 3.951235 -2.150887 0.103480
C 2.930999 -2.252148 -0.846835
C 2.019332 -1.196225 -0.968866
C 2.110126 -0.057761 -0.157480
C 3.145359 0.010749 0.790312
H 4.668402 -2.963098 0.203128
H 1.240555 -1.250294 -1.724388
H 3.212414 0.876406 1.442800
C 5.180026 -0.943539 1.953072
H 5.148461 -1.794897 2.642363
H 6.164234 -0.953507 1.470276
H 5.107215 -0.027852 2.546351
C 2.798732 -3.480450 -1.715831
H 3.738685 -4.037762 -1.765172
H 2.032086 -4.159041 -1.321737
H 2.505726 -3.216047 -2.736942
C 1.133625 1.050843 -0.294859
C -0.219799 0.783802 -0.534469
C 1.545633 2.390264 -0.180558
C -1.150559 1.821413 -0.662740
H -0.561788 -0.245111 -0.581547
C 0.636794 3.443008 -0.299029
H 2.596385 2.610811 -0.016375
C -0.712477 3.141635 -0.538140
H -1.432251 3.952898 -0.626958
C 1.089821 4.879451 -0.187144
H 2.147543 4.942791 0.082940
H 0.953906 5.412511 -1.135508
H 0.514113 5.418997 0.573288
C -2.612614 1.513253 -0.940457
H -2.732065 1.252908 -1.999647
H -3.201390 2.423891 -0.779531
C -3.168946 0.389274 -0.091533
C -3.572975 -0.816800 -0.669807
C -3.258427 0.532798 1.300428
C -4.067365 -1.879488 0.102013
H -3.498677 -0.935990 -1.749011
C -3.745822 -0.511256 2.083126
H -2.929860 1.458094 1.765626
C -4.146597 -1.711926 1.488347
H -3.809022 -0.395781 3.161329
H -4.520215 -2.523844 2.106708
C -4.523760 -3.159019 -0.557264
H -4.524929 -3.993181 0.150370
H -5.543295 -3.057518 -0.949613
H -3.876643 -3.427529 -1.398490
47
6-A-2Me-12 Eopt
-891.105029
C 4.064420 -0.705434 1.107420
C 4.238033 -1.719320 0.156646
C 3.381012 -1.843638 -0.942820
C 2.335389 -0.925487 -1.085738
C 2.131479 0.099147 -0.150080
C 3.006479 0.193241 0.943534
H 5.056871 -2.425888 0.275761
H 1.680978 -0.994413 -1.949986
H 2.843776 0.965131 1.690240
C 5.014001 -0.576846 2.274960
H 5.292286 -1.557707 2.673572
H 5.940476 -0.072246 1.973884
H 4.569767 0.006384 3.086755
C 3.565823 -2.960449 -1.942704
H 4.619137 -3.241178 -2.038447
H 3.013749 -3.857186 -1.634693
H 3.198202 -2.673545 -2.932503
C 1.012621 1.060242 -0.311105
C -0.235875 0.631882 -0.777313
C 1.181500 2.419375 0.008596
C -1.299516 1.529850 -0.927892
H -0.397026 -0.421016 -0.986857
C 0.136550 3.334083 -0.128738
H 2.151878 2.767815 0.350181
C -1.103187 2.871935 -0.596723
H -1.928347 3.573303 -0.702487
C 0.332407 4.795282 0.199098
H 1.302175 4.969955 0.673275
H 0.286921 5.412990 -0.705741
H -0.446902 5.157933 0.878504
C -2.643626 1.043049 -1.439061
H -2.566392 0.817896 -2.509899
H -3.372039 1.857852 -1.348495
C -3.161122 -0.184881 -0.716460
C -3.337748 -0.159382 0.674962
C -3.460154 -1.359832 -1.409728
C -3.808382 -1.271022 1.377551
H -3.087389 0.747625 1.220229
C -3.932080 -2.484108 -0.723136
H -3.323342 -1.398693 -2.487212
C -4.104983 -2.440522 0.658114
H -4.160737 -3.394568 -1.269665
H -4.469142 -3.317707 1.187063
C -3.992717 -1.225456 2.875603
H -5.038427 -1.403509 3.152002
H -3.390411 -1.995029 3.371759
H -3.698017 -0.254441 3.282842
47
6-A-2Me-13 Eopt
-891.105442
C -2.475950 -2.485601 -0.530696
C -3.799913 -2.350211 -0.094952
C -4.288565 -1.130610 0.388782
C -3.425759 -0.030648 0.428826
C -2.092927 -0.134006 0.003553
C -1.633281 -1.371536 -0.472642
H -4.463605 -3.211691 -0.133324
H -3.787044 0.914834 0.823453
H -0.610625 -1.458000 -0.828368
C -1.980331 -3.798312 -1.089742
H -2.407788 -4.648670 -0.548872
H -2.261531 -3.907264 -2.144567
H -0.890404 -3.869508 -1.030843
C -5.706665 -1.017419 0.896465
H -6.391619 -1.635062 0.306986
H -5.778898 -1.352597 1.938619
H -6.062282 0.016463 0.858544
C -1.184231 1.037698 0.054436
C 0.158714 0.880851 0.416761
C -1.647696 2.324454 -0.271060
C 1.031390 1.974139 0.454003
H 0.530055 -0.104200 0.679941
C -0.797982 3.431770 -0.241483
H -2.679900 2.454289 -0.583246
C 0.543864 3.239797 0.120890
H 1.218615 4.093352 0.139373
C -1.299936 4.808487 -0.607490
H -2.354522 4.783316 -0.895421
H -1.197779 5.503582 0.233982
H -0.732170 5.228568 -1.445565
C 2.480133 1.784814 0.872981
H 2.534680 1.709294 1.966241
H 3.047229 2.681665 0.597586
C 3.128013 0.560768 0.260347
C 3.506219 -0.526058 1.052373
C 3.322980 0.484499 -1.126503
C 4.076195 -1.683385 0.499146
H 3.349548 -0.475308 2.128092
C 3.886757 -0.655748 -1.693877
H 3.016550 1.314820 -1.756796
C 4.259826 -1.735412 -0.886315
H 4.032927 -0.710149 -2.768865
H 4.694756 -2.623507 -1.337201
C 4.498273 -2.828960 1.387735
H 4.626829 -3.751349 0.814116
H 5.452587 -2.611672 1.883213
H 3.759630 -3.017443 2.173702
47
6-A-2Me-14 Eopt
-891.105032
C 4.064336 -0.702390 1.109597
C 4.242122 -1.714354 0.155467
C 3.387434 -1.838414 -0.944046
C 2.338456 -0.921876 -1.086213
C 2.131818 0.099902 -0.150070
C 3.006592 0.194336 0.945420
H 5.063358 -2.418335 0.273373
H 1.685793 -0.990305 -1.951975
H 2.842344 0.965844 1.692049
C 5.004831 -0.587697 2.285896
H 5.103258 -1.543618 2.811907
H 6.009213 -0.291423 1.960375
H 4.653680 0.158293 3.004291
C 3.575246 -2.948099 -1.951323
H 4.597605 -3.336690 -1.931419
H 2.898857 -3.786139 -1.742201
H 3.361733 -2.603197 -2.968032
C 1.012248 1.060270 -0.310556
C -0.235651 0.631258 -0.777730
C 1.179869 2.419259 0.010370
C -1.299847 1.528527 -0.928466
H -0.395806 -0.421661 -0.987970
C 0.134336 3.333295 -0.127127
H 2.149703 2.768187 0.353006
C -1.104726 2.870548 -0.596301
H -1.930361 3.571353 -0.702079
C 0.328901 4.794412 0.201839
H 1.297980 4.969401 0.677307
H 0.284177 5.412628 -0.702696
H -0.451467 5.156200 0.880484
C -2.643295 1.040966 -1.440599
H -2.565073 0.815269 -2.511249
H -3.372107 1.855534 -1.351102
C -3.160968 -0.186790 -0.717785
C -3.337370 -0.161139 0.673641
C -3.460112 -1.361839 -1.410881
C -3.808103 -1.272647 1.376417
H -3.086513 0.745789 1.218810
C -3.931723 -2.486091 -0.724068
H -3.323153 -1.400927 -2.488339
C -4.104385 -2.442358 0.657230
H -4.159912 -3.396801 -1.270378

H	-4.467863	-3.319677	1.186417
C	-3.995802	-1.225514	2.873999
H	-5.048664	-1.365153	3.145817
H	-3.424802	-2.018667	3.369726
H	-3.667224	-0.267295	3.285622

47

6-A-2Me-2 Eopt

-891.104877

C	4.071361	-1.023443	0.932360
C	3.953181	-2.149062	0.104381
C	2.933972	-2.250619	-0.847044
C	2.021683	-1.195320	-0.969512
C	2.110776	-0.057181	-0.157434
C	3.144929	0.011606	0.791470
H	4.670771	-2.960848	0.204446
H	1.243661	-1.249633	-1.725791
H	3.210574	0.876896	1.444592
C	5.179932	-0.941076	1.954925
H	5.157021	-1.799168	2.636093
H	6.163948	-0.936886	1.471569
H	5.098705	-0.031699	2.556767
C	2.803410	-3.478735	-1.716561
H	3.744932	-4.033175	-1.768484
H	2.039800	-4.159935	-1.321069
H	2.507196	-3.214537	-2.736784
C	1.133480	1.050675	-0.295189
C	-0.219488	0.782666	-0.536245
C	1.544290	2.390379	-0.179804
C	-1.150947	1.819594	-0.664946
H	-0.560623	-0.246503	-0.584101
C	0.634737	3.442454	-0.298667
H	2.594702	2.611683	-0.014465
C	-0.714059	3.140105	-0.539282
H	-1.434407	3.950816	-0.628459
C	1.086499	4.879201	-0.185638
H	2.144018	4.943259	0.085072
H	0.950657	5.412750	-1.133739
H	0.509908	5.417789	0.574807
C	-2.612481	1.510253	-0.944012
H	-2.730457	1.247674	-2.002803
H	-3.201904	2.420921	-0.785657
C	-3.169297	0.387699	-0.093471
C	-3.572782	-0.819410	-0.669821
C	-3.259853	0.533788	1.298208
C	-4.067710	-1.880756	0.103612
H	-3.497690	-0.940581	-1.748757
C	-3.747812	-0.508799	2.082417
H	-2.931718	1.459978	1.761927
C	-4.148082	-1.710628	1.489509
H	-3.811919	-0.391343	3.160352
H	-4.522190	-2.521400	2.109065
C	-4.523000	-3.161583	-0.553911
H	-4.535855	-3.991951	0.158022
H	-5.537604	-3.057326	-0.958110
H	-3.867949	-3.437628	-1.386561

47

6-A-2Me-3 Eopt

-891.105529

C	4.430378	-0.769364	0.220729
C	4.019741	-2.081933	-0.030104
C	2.671781	-2.396510	-0.256002
C	1.729316	-1.367121	-0.223159
C	2.108496	-0.037729	0.029820
C	3.463104	0.243145	0.250387
H	4.761984	-2.877374	-0.048327
H	0.684990	-1.594200	-0.416377
H	3.766088	1.261538	0.477519
C	5.888032	-0.441253	0.442525
H	6.455198	-1.326959	0.743188
H	6.346807	-0.050687	-0.474322
H	6.011055	0.321192	1.218376
C	2.256615	-3.820553	-0.540618
H	2.686034	-4.177274	-1.484229
H	2.600047	-4.498692	0.248675
H	1.169238	-3.910214	-0.612563
C	1.090298	1.040774	0.065977
C	-0.171590	0.810462	0.625896
C	1.365823	2.310126	-0.472638
C	-1.147229	1.813629	0.650916
H	-0.398666	-0.160823	1.053577
C	0.411251	3.328530	-0.458560
H	2.330272	2.491579	-0.938142
C	-0.846321	3.063062	0.104757
H	-1.603177	3.844868	0.112246
C	0.713918	4.687419	-1.044148
H	0.729852	5.458514	-0.264751
H	1.686774	4.697307	-1.543380
H	-0.044961	4.983194	-1.777050
C	-2.504176	1.543030	1.277431
H	-3.184488	2.361854	1.014438
H	-2.411039	1.559747	2.370413
C	-3.109083	0.219230	0.855681
C	-3.369171	-0.033357	-0.499383
C	-3.393403	-0.778397	1.791065
C	-3.907624	-1.247703	-0.932045
H	-3.129981	0.733666	-1.232476
C	-3.930117	-2.002347	1.376820
H	-3.190341	-0.602313	2.844046
C	-4.184310	-2.235781	0.027216
H	-4.144146	-2.774496	2.110204
H	-4.597672	-3.189986	-0.289711
C	-4.199503	-1.492800	-2.393236
H	-3.814098	-2.465182	-2.718335
H	-5.279284	-1.490812	-2.585360
H	-3.747847	-0.721802	-3.023626

47

6-A-2Me-4 Eopt

-891.105430

C	4.287010	-1.137021	0.390861
C	3.793811	-2.357879	-0.080758
C	2.467958	-2.492079	-0.515872
C	1.630163	-1.375714	-0.466255
C	2.093015	-0.136307	0.006025
C	3.424616	-0.034922	0.431269
H	4.453003	-3.223202	-0.108125
H	0.606152	-1.461890	-0.817737
H	3.786623	0.909351	0.828341
C	5.724724	-1.002811	0.834200
H	6.127343	-1.959965	1.178859
H	6.359383	-0.657253	0.008585
H	5.824710	-0.278224	1.648261
C	1.966336	-3.814434	-1.046114
H	2.410449	-4.042336	-2.022620
H	2.226356	-4.637347	-0.371454
H	0.879730	-3.806850	-1.168631
C	1.184926	1.035806	0.057891
C	-0.158221	0.879929	0.419927
C	1.649498	2.322543	-0.266321
C	-1.029856	1.974010	0.458378
H	-0.530740	-0.104942	0.682030
C	0.800946	3.430683	-0.235276
H	2.681777	2.451715	-0.578532
C	-0.541110	3.239587	0.126832
H	-1.215064	4.093753	0.146149
C	1.304460	4.807455	-0.598906
H	1.209388	5.499409	0.246011
H	2.357240	4.780566	-0.893235
H	0.732779	5.232511	-1.431761
C	-2.478923	1.785271	0.876494
H	-3.044747	2.683867	0.604226
H	-2.533780	1.705791	1.969450
C	-3.128125	0.564287	0.259144
C	-3.506198	-0.525777	1.046747
C	-3.324159	0.494104	-1.127889
C	-4.077021	-1.680498	0.488925
H	-3.348744	-0.479724	2.122567
C	-3.888874	-0.643409	-1.699763
H	-3.017859	1.327021	-1.754813
C	-4.261772	-1.726419	-0.896589
H	-4.035938	-0.693070	-2.774859
H	-4.697457	-2.612359	-1.350964
C	-4.498483	-2.829851	1.372911
H	-5.449692	-2.612466	1.874260
H	-4.632592	-3.748506	0.794608
H	-3.756647	-3.025176	2.154212

47

6-A-2Me-5 Eopt

-891.104824

C	-2.780021	-2.322168	-0.821797
C	-3.843887	-2.240419	0.081929
C	-4.046002	-1.100132	0.872217
C	-3.158920	-0.029300	0.742155
C	-2.078392	-0.080310	-0.154307
C	-1.903425	-1.235686	-0.927519
H	-4.528690	-3.080743	0.175328
H	-3.289156	0.848483	1.368329
H	-1.084574	-1.280322	-1.639956
C	-2.596669	-3.540793	-1.695238
H	-3.085297	-4.418660	-1.262068
H	-3.028825	-3.379643	-2.690738
H	-1.536315	-3.775017	-1.833884
C	-5.184615	-1.049317	1.863382
H	-6.132672	-1.335417	1.394736
H	-5.012948	-1.740287	2.697588
H	-5.303264	-0.045777	2.281732
C	-1.138869	1.061806	-0.275088
C	0.235916	0.837385	-0.444329
C	-1.602502	2.384327	-0.214618
C	1.133232	1.902211	-0.553428
H	0.613101	-0.179703	-0.450266
C	-0.724463	3.468475	-0.317618
H	-2.667018	2.571032	-0.103606
C	0.641543	3.211045	-0.482798
H	1.335950	4.045292	-0.555049
C	-1.248015	4.884864	-0.280579
H	-1.694276	5.164205	-1.243014
H	-2.022578	5.003988	0.484120
H	-0.447729	5.599791	-0.067890
C	2.620096	1.653146	-0.755697
H	3.169283	2.555032	-0.460494
H	2.816965	1.511020	-1.825914
C	3.154877	0.459593	0.006300
C	3.568943	-0.694683	-0.666113
C	3.212526	0.480417	1.405978
C	4.039383	-1.823076	0.020409
H	3.519933	-0.717853	-1.752878
C	3.676803	-0.632148	2.106158
H	2.878342	1.363523	1.943434

C 4.085406 -1.778124 1.418860
H 3.714591 -0.610861 3.191442
H 4.439864 -2.643908 1.972045
C 4.508981 -3.044441 -0.733188
H 5.585797 -2.990413 -0.937023
H 4.329798 -3.959350 -0.160054
H 3.998153 -3.140427 -1.696348

47

6-A-2Me-6 Eopt

-891.105033

C -3.385541 -1.833585 -0.946596
C -4.235134 -1.717378 0.157761
C -4.056563 -0.708511 1.114944
C -3.003876 0.193793 0.948399
C -2.130195 0.102940 -0.148221
C -2.335414 -0.917993 -1.085571
H -5.051800 -2.426302 0.277958
H -2.838684 0.962705 1.697526
H -1.680809 -0.986336 -1.949899
C -3.613761 -2.900081 -1.991520
H -4.194673 -3.734883 -1.588571
H -4.165761 -2.497350 -2.849808
H -2.666119 -3.295988 -2.370154
C -4.971784 -0.622046 2.313399
H -6.024506 -0.618278 2.009929
H -4.829762 -1.479609 2.981798
H -4.783951 0.286389 2.892492
C -1.010520 1.063645 -0.306149
C 0.237943 0.635722 -0.772770
C -1.178976 2.422003 0.016832
C 1.301964 1.533605 -0.921025
H 0.398591 -0.416811 -0.984512
C -0.133590 3.336604 -0.118146
H -2.149276 2.769965 0.359173
C 1.106076 2.875037 -0.586837
H 1.931588 3.576284 -0.690650
C -0.328882 4.797091 0.213204
H -0.281680 5.417103 -0.689968
H -1.299187 4.971234 0.686473
H 0.449725 5.157441 0.894642
C 2.645941 1.047730 -1.433535
H 3.375564 1.860750 -1.336887
H 2.569641 0.829675 -2.505923
C 3.160603 -0.185684 -0.718359
C 3.336545 -0.169233 0.673252
C 3.457020 -1.357122 -1.418717
C 3.804204 -1.286331 1.369200
H 3.087681 0.734855 1.224031
C 3.925393 -2.486900 -0.738809
H 3.320291 -1.389090 -2.496442
C 4.097595 -2.452299 0.642810
H 4.151255 -3.394816 -1.290707
H 4.458355 -3.333940 1.166640
C 3.992737 -1.248596 2.866942
H 3.431343 -2.051902 3.357140
H 5.047462 -1.378331 3.136637
H 3.654016 -0.297220 3.286157

47

6-A-2Me-7 Eopt

-891.104970

C 4.590113 -1.502054 0.485400
C 5.343491 -0.793876 -0.456464
C 4.822982 0.328938 -1.114574
C 3.522515 0.739616 -0.812108
C 2.738205 0.048949 0.126327
C 3.287560 -1.071741 0.764196
H 6.355563 -1.122299 -0.684134
H 3.098356 1.592555 -1.334012
H 2.700509 -1.600811 1.509727
C 5.181367 -2.682637 1.218903
H 5.965349 -3.166592 0.628799
H 5.630904 -2.369080 2.169297
H 4.417680 -3.431545 1.450368
C 5.644889 1.056350 -2.152213
H 6.656750 1.260919 -1.786158
H 5.744656 0.458686 -3.066318
H 5.185054 2.009655 -2.427391
C 1.356887 0.494913 0.433610
C 0.327670 -0.442049 0.628721
C 1.046593 1.857900 0.529491
C -0.976731 -0.034723 0.910369
H 0.545411 -1.501414 0.527268
C -0.254605 2.292490 0.812819
H 1.836485 2.592872 0.401940
C -1.256514 1.335413 0.995308
H -2.276243 1.657517 1.192271
C -0.558979 3.765343 0.950752
H -0.374497 4.110030 1.975854
H 0.068969 4.366571 0.286054
H -1.606080 3.978067 0.715772
C -2.086720 -1.051409 1.096622
H -2.474949 -0.987671 2.120213
H -1.667003 -2.058649 0.987351
C -3.236883 -0.873583 0.123310
C -4.547454 -0.717758 0.581689
C -2.999210 -0.856514 -1.258805
C -5.626393 -0.553441 -0.300785
H -4.736466 -0.721767 1.653497
C -4.057612 -0.694097 -2.149602
H -1.982732 -0.955122 -1.629039
C -5.364357 -0.542023 -1.674530
H -3.866968 -0.678510 -3.218879
H -6.183618 -0.409602 -2.376284
C -7.034227 -0.415659 0.227842
H -7.701278 0.016346 -0.523716
H -7.445165 -1.392212 0.512474
H -7.066214 0.221983 1.117379

47

6-A-2Me-8 Eopt

-891.105175

C 4.420727 -1.264743 0.887115
C 5.099784 -0.976440 -0.300922
C 4.535834 -0.151335 -1.284017
C 3.267693 0.388734 -1.058289
C 2.557504 0.116643 0.122832
C 3.148581 -0.714333 1.084142
H 6.087125 -1.402763 -0.466416
H 2.808627 1.008136 -1.823393
H 2.620443 -0.913838 2.012404
C 5.060145 -2.118849 1.956226
H 5.805365 -2.797815 1.531263
H 5.569034 -1.496870 2.703081
H 4.314278 -2.719746 2.485752
C 5.277961 0.120540 -2.571140
H 6.303906 0.451291 -2.375937
H 5.340228 -0.782880 -3.189553
H 4.779805 0.894435 -3.161731
C 1.209297 0.694581 0.345648
C 0.192372 -0.067895 0.945696
C 0.918104 2.008964 -0.042376
C -1.081575 0.461829 1.152800
H 0.393038 -1.098498 1.224185
C -0.352199 2.565062 0.156996
H 1.700542 2.617221 -0.487608
C -1.342928 1.778192 0.750344
H -2.340565 2.187550 0.890635
C -0.632193 3.997330 -0.231789
H -0.345097 4.685725 0.572773
H -0.068941 4.285853 -1.124676
H -1.695935 4.153228 -0.434102
C -2.183133 -0.377102 1.770043
H -2.525302 0.091134 2.700548
H -1.770962 -1.354454 2.049357
C -3.377132 -0.574875 0.853812
C -3.192096 -1.041422 -0.455973
C -4.675039 -0.301708 1.292398
C -4.268505 -1.246396 -1.322744
H -2.181780 -1.235808 -0.807650
C -5.765055 -0.498187 0.437653
H -4.835862 0.066810 2.302145
C -5.564265 -0.966862 -0.858699
H -6.770727 -0.280183 0.785607
H -6.414506 -1.114519 -1.519886
C -4.050527 -1.758596 -2.726548
H -4.498635 -2.750382 -2.859653
H -4.509056 -1.093774 -3.467137
H -2.985165 -1.838649 -2.959273

47

6-A-2Me-9 Eopt

-891.105123

C 5.001044 0.189511 -0.005768
C 5.061721 -1.207032 0.001616
C 3.898757 -1.989739 0.043560
C 2.660114 -1.346623 0.079817
C 2.563729 0.055452 0.076259
C 3.744893 0.807677 0.035110
H 6.032502 -1.697741 -0.024237
H 1.751657 -1.941877 0.088430
H 3.686324 1.892208 0.062651
C 6.260321 1.021037 -0.070757
H 7.135929 0.444277 0.240809
H 6.444425 1.375992 -1.092325
H 6.187883 1.903952 0.572409
C 3.992957 -3.497158 0.047228
H 4.545467 -3.863019 -0.825565
H 4.519136 -3.857977 0.938699
H 3.001153 -3.957510 0.034606
C 1.239240 0.722792 0.117405
C 0.200733 0.208095 0.912511
C 0.991964 1.875579 -0.639610
C -1.051092 0.822391 0.952947
H 0.382136 -0.672512 1.521985
C -0.255454 2.512913 -0.614203
H 1.775090 2.266408 -1.283283
C -1.268528 1.971787 0.181760
H -2.249534 2.440881 0.193857
C -0.493185 3.765744 -1.423418
H -0.171369 4.657038 -0.870446
H 0.066803 3.744921 -2.363499
H -1.553665 3.891061 -1.660727
C -2.170438 0.253933 1.802398
H -2.482384 0.995279 2.547434
H -1.787537 -0.606450 2.364822
C -3.384643 -0.169591 0.995421
C -3.234009 -0.997376 -0.126704
C -4.668682 0.249091 1.353206
C -4.330639 -1.417494 -0.883876
H -2.234481 -1.307334 -0.421674

C	-5.778312	-0.158938	0.605475
H	-4.802968	0.897015	2.215410
C	-5.611511	-0.985382	-0.503635
H	-6.772656	0.174714	0.888178
H	-6.476846	-1.295627	-1.083961
C	-4.149069	-2.320729	-2.080417
H	-4.563554	-3.317831	-1.889125
H	-4.661607	-1.920326	-2.961969
H	-3.091376	-2.441180	-2.330424
44			
6-A-Me-1			Eopt
-851.779186			
C	-4.256640	-0.540307	0.326857
C	-3.968861	-1.861712	-0.032376
C	-2.668476	-2.261631	-0.367530
C	-1.645422	-1.310366	-0.335589
C	-1.901379	0.024389	0.017148
C	-3.214160	0.392969	0.343631
H	-4.772804	-2.594748	-0.052240
H	-0.637467	-1.602499	-0.615598
H	-3.421486	1.416693	0.642518
C	-5.655440	-0.137803	0.730421
H	-6.408500	-0.740065	0.213047
H	-5.808361	-0.276113	1.808015
H	-5.847992	0.915528	0.505170
C	-2.387961	-3.684485	-0.789512
H	-2.919871	-4.400383	-0.154236
H	-2.713924	-3.860285	-1.822049
H	-1.319399	-3.911374	-0.736932
C	-0.802128	1.020528	0.041898
C	0.470360	0.665451	0.503957
C	-1.011542	2.336250	-0.407968
C	1.519791	1.591462	0.519514
H	0.646860	-0.343272	0.863565
C	0.017683	3.279093	-0.402189
H	-1.986262	2.615383	-0.797490
C	1.283132	2.889636	0.063027
H	2.096427	3.612563	0.064953
C	-0.212772	4.686847	-0.898349
H	-1.232444	4.814491	-1.272038
H	-0.054326	5.419098	-0.098029
H	0.477921	4.940102	-1.710648
C	2.887249	1.186693	1.042467
H	2.857416	1.132691	2.137787
H	3.608536	1.973909	0.792998
C	3.371698	-0.141435	0.497385
C	3.558691	-0.312828	-0.882156
C	3.620158	-1.225498	1.345676
C	3.989233	-1.534515	-1.397960
H	3.352286	0.516655	-1.553272
C	4.049890	-2.454185	0.833559
H	3.473934	-1.108849	2.416481
C	4.235938	-2.612158	-0.540255
H	4.236131	-3.284518	1.508439
H	4.569026	-3.564561	-0.941514
H	4.130055	-1.648826	-2.468858
44			
6-A-Me-2			Eopt
-851.778684			
C	3.985614	-0.487264	1.001206
C	4.151105	-1.552158	0.103972
C	3.226818	-1.796978	-0.915914
C	2.119146	-0.948742	-1.036431
C	1.923526	0.123415	-0.156132
C	2.869438	0.339664	0.860201
H	5.018251	-2.201826	0.203574
H	1.410294	-1.110699	-1.843613
H	2.716690	1.152074	1.564680
C	4.998027	-0.247094	2.096040
H	5.127949	-1.138488	2.719965
H	5.980405	0.002192	1.677987
H	4.692150	0.577102	2.746347
C	3.404275	-2.961850	-1.860830
H	4.444945	-3.297612	-1.889726
H	2.789774	-3.815904	-1.550177
H	3.103319	-2.697570	-2.879593
C	0.742458	1.010997	-0.292869
C	-0.510290	0.486442	-0.631644
C	0.856180	2.396360	-0.078511
C	-1.631471	1.315236	-0.760044
H	-0.625303	-0.585712	-0.757671
C	-0.246321	3.243675	-0.196257
H	1.827475	2.817964	0.163423
C	-1.488512	2.685865	-0.536252
H	-2.357617	3.334329	-0.626180
C	-0.111064	4.732471	0.018289
H	0.884164	4.991835	0.389748
H	-0.273161	5.283007	-0.915992
H	-0.848174	5.098001	0.741874
C	-2.979422	0.723129	-1.129885
H	-2.959279	0.394947	-2.176343
H	-3.740178	1.510616	-1.069566
C	-3.389287	-0.446755	-0.257132
C	-3.480875	-0.293981	1.134034
C	-3.675432	-1.698101	-0.812964
C	-3.856891	-1.363144	1.946041
H	-3.242705	0.668049	1.579652
C	-4.050862	-2.774644	-0.002813
H	-3.603116	-1.832554	-1.889297
C	-4.143328	-2.609908	1.379632
H	-4.267338	-3.739038	-0.453059
H	-4.432832	-3.443750	2.011981
H	-3.922713	-1.226783	3.021557
44			
6-A-Me-3			Eopt
-851.778795			
C	-3.788151	-1.922024	-0.198701
C	-4.910787	-1.092427	-0.078870
C	-4.781681	0.288661	0.105262
C	-3.497338	0.840667	0.164989
C	-2.353004	0.038281	0.051524
C	-2.517567	-1.344436	-0.127987
H	-5.904932	-1.531750	-0.130439
H	-3.384132	1.908438	0.330062
H	-1.639972	-1.974064	-0.243412
C	-3.952593	-3.404509	-0.436281
H	-4.740073	-3.824456	0.198126
H	-4.230399	-3.607759	-1.477764
H	-3.025169	-3.946232	-0.229781
C	-6.002316	1.160991	0.279615
H	-6.858531	0.763888	-0.274168
H	-6.293901	1.221351	1.335551
H	-5.815745	2.181782	-0.067324
C	-0.997135	0.637031	0.117564
C	0.045545	-0.021292	0.792135
C	-0.724203	1.866786	-0.495633
C	1.326250	0.528140	0.854217
H	-0.154830	-0.964912	1.291445
C	0.552935	2.440825	-0.445940
H	-1.512429	2.371693	-1.047117
C	1.569010	1.757554	0.227386
H	2.571077	2.179002	0.255672
C	0.818987	3.776606	-1.098422
H	0.233988	3.895226	-2.015779
H	0.547469	4.601752	-0.428259
H	1.876904	3.892393	-1.351282
C	2.448002	-0.192196	1.575476
H	2.044974	-1.098955	2.043007
H	2.824141	0.434326	2.392823
C	3.608879	-0.565204	0.671023
C	4.927222	-0.267936	1.033716
C	3.380995	-1.226383	-0.544517
C	5.997444	-0.627473	0.208688
H	5.118798	0.250334	1.969955
C	4.445138	-1.589262	-1.369760
H	2.360958	-1.444842	-0.847881
C	5.759505	-1.290835	-0.995930
H	4.249655	-2.101042	-2.307641
H	6.587888	-1.569796	-1.640115
H	7.013449	-0.385763	0.507008
44			
6-A-Me-4			Eopt
-851.778835			
C	4.204578	-1.466264	-0.523036
C	4.942189	-0.800170	0.460234
C	4.408395	0.289680	1.162885
C	3.111190	0.709746	0.861996
C	2.344541	0.065103	-0.123416
C	2.907385	-1.021062	-0.806500
H	5.953237	-1.133947	0.684546
H	2.677400	1.537410	1.415596
H	2.336713	-1.509969	-1.591166
C	4.783764	-2.652999	-1.256220
H	5.877269	-2.644770	-1.228170
H	4.449126	-3.595040	-0.804317
H	4.470074	-2.665063	-2.304804
C	5.224803	0.986518	2.225524
H	5.527084	0.289195	3.015118
H	6.141152	1.414518	1.802793
H	4.659897	1.798098	2.692335
C	0.969557	0.526997	-0.435415
C	-0.054717	-0.398577	-0.699903
C	0.659856	1.892976	-0.467569
C	-1.353185	0.022955	-0.987214
H	0.161880	-1.461798	-0.649052
C	-0.635563	2.342078	-0.755653
H	1.446268	2.620441	-0.286682
C	-1.632352	1.395838	-1.008163
H	-2.647677	1.727985	-1.211527
C	-0.937089	3.820317	-0.824525
H	-0.346007	4.382459	-0.094866
H	-0.700575	4.223301	-1.817128
H	-1.995427	4.019198	-0.632056
C	-2.459854	-0.981670	-1.239582
H	-2.031711	-1.991515	-1.231180
H	-2.872923	-0.830266	-2.243879
C	-3.590057	-0.904869	-0.228992
C	-4.923358	-0.824036	-0.645464
C	-3.316900	-0.921350	1.146458
C	-5.964499	-0.768051	0.286403
H	-5.149786	-0.803916	-1.708575
C	-4.351877	-0.868445	2.079698
H	-2.284914	-0.964373	1.483292
C	-5.681672	-0.791237	1.652817
H	-4.121619	-0.883064	3.141070
H	-6.487345	-0.746298	2.379383
H	-6.992925	-0.703446	-0.056910
44			

6-A-Me-5 Eopt
-851.778796

C	4.781650	0.287079	-0.102687
C	4.909722	-1.093181	0.082676
C	3.785886	-1.922662	0.200856
C	2.516412	-1.344534	0.129052
C	2.352699	0.038851	-0.051935
C	3.497040	0.840023	-0.164905
H	5.903573	-1.532837	0.137232
H	1.638312	-1.973194	0.245278
H	3.384854	1.907940	-0.330076
C	6.001665	1.160819	-0.274411
H	6.875944	0.726222	0.219346
H	6.250166	1.283377	-1.335949
H	5.837940	2.160367	0.140008
C	3.951813	-3.406586	0.428273
H	4.634579	-3.847146	-0.306467
H	4.369005	-3.609666	1.421798
H	2.994233	-3.929701	0.355938
C	0.996972	0.637846	-0.118441
C	-0.045820	-0.020955	-0.792307
C	0.724239	1.868329	0.493485
C	-1.326422	0.528727	-0.855090
H	0.154274	-0.965219	-1.290510
C	-0.552691	2.442647	0.442998
H	1.512494	2.373547	1.044628
C	-1.568911	1.758874	-0.229690
H	-2.570890	2.180515	-0.258457
C	-0.818629	3.779280	1.093779
H	-0.228127	3.902059	2.007023
H	-0.554040	4.603506	0.419712
H	-1.875270	3.892529	1.353105
C	-2.448344	-0.192333	-1.575319
H	-2.045266	-1.099106	-2.042791
H	-2.825320	0.433705	-2.392648
C	-3.608467	-0.565548	-0.669957
C	-3.379418	-1.224727	0.546454
C	-4.927289	-0.270562	-1.032763
C	-4.442892	-1.587894	1.372429
H	-2.359021	-1.441390	0.849882
C	-5.996847	-0.630412	-0.207006
H	-5.119791	0.246157	-1.969670
C	-5.757748	-1.291773	0.998478
H	-7.013238	-0.390479	-0.505450
H	-6.585599	-1.570955	1.643252
H	-4.246521	-2.098090	2.310987

44

6-A-Me-6 Eopt
-851.779186

C	-4.255094	-0.540646	0.331047
C	-3.967474	-1.862664	-0.030018
C	-2.668736	-2.261521	-0.368526
C	-1.645015	-1.309493	-0.337639
C	-1.900433	0.023959	0.016423
C	-3.213548	0.392229	0.346143
H	-4.771090	-2.596111	-0.047825
H	-0.637361	-1.601609	-0.619070
H	-3.420087	1.415476	0.646910
C	-5.654499	-0.141761	0.736074
H	-6.407030	-0.662708	0.135598
H	-5.845510	-0.391040	1.787242
H	-5.811594	0.934560	0.619615
C	-2.386519	-3.681809	-0.797954
H	-3.009315	-4.395347	-0.249239
H	-2.596514	-3.818104	-1.866120
H	-1.338059	-3.946543	-0.632879
C	-0.801761	1.020741	0.040930
C	0.471354	0.666197	0.501698
C	-1.012438	2.336523	-0.408111
C	1.520265	1.592794	0.516576
H	0.648647	-0.342546	0.860841
C	0.016306	3.279927	-0.403086
H	-1.987764	2.615295	-0.796378
C	1.282417	2.890999	0.060714
H	2.095288	3.614406	0.062161
C	-0.215426	4.687702	-0.898598
H	-1.235646	4.814988	-1.270917
H	-0.056226	5.419837	-0.098326
H	0.474074	4.941422	-1.711770
C	2.888390	1.189033	1.038658
H	2.860047	1.138143	2.134176
H	3.609484	1.975413	0.786014
C	3.371885	-0.140674	0.496695
C	3.558639	-0.315466	-0.882456
C	3.619622	-1.222913	1.347517
C	3.988223	-1.538684	-1.395412
H	3.352833	0.512603	-1.555504
C	4.048406	-2.453128	0.838269
H	3.473582	-1.103623	2.418056
C	4.234208	-2.614483	-0.535181
H	4.234131	-3.281984	1.515103
H	4.566576	-3.568067	-0.934224
H	4.128889	-1.655635	-2.466046

44

6-A-Me-7 Eopt
-851.778833

C	4.408263	0.282645	1.166397
C	4.937286	-0.808665	0.470396
C	4.198686	-1.473338	-0.518752
C	2.907176	-1.025294	-0.804501
C	2.344862	0.066384	-0.121949
C	3.109099	0.707297	0.862362
H	5.943919	-1.150471	0.702400
H	2.334129	-1.515754	-1.586031
H	2.675385	1.533473	1.418810
C	5.224516	1.003301	2.213178
H	5.997305	0.353472	2.634220
H	5.727850	1.878682	1.784139
H	4.594314	1.359263	3.034169
C	4.793397	-2.650963	-1.254145
H	5.761377	-2.394740	-1.698884
H	4.961475	-3.495778	-0.575754
H	4.133761	-2.992916	-2.056443
C	0.969845	0.527104	-0.435507
C	-0.054036	-0.398996	-0.699748
C	0.659666	1.892952	-0.468566
C	-1.352782	0.021968	-0.986701
H	0.162836	-1.462146	-0.648836
C	-0.635995	2.341463	-0.756385
H	1.446054	2.620667	-0.288527
C	-1.632527	1.394725	-1.008022
H	-2.648002	1.726395	-1.211422
C	-0.938000	3.819552	-0.826276
H	-0.345626	4.382651	-0.098412
H	-0.703533	4.221477	-1.819803
H	-1.996012	4.018399	-0.631992
C	-2.459091	-0.983181	-1.238739
H	-2.030711	-1.992907	-1.229076
H	-2.871546	-0.832845	-2.243452
C	-3.589918	-0.905584	-0.228943
C	-3.317683	-0.922420	1.146689
C	-4.922869	-0.823499	-0.646282
C	-4.353216	-0.868568	2.079254
H	-2.285962	-0.966516	1.484200
C	-5.964568	-0.766548	0.284906
H	-5.148577	-0.803158	-1.709542
C	-5.682657	-0.790064	1.651501
H	-6.992709	-0.700984	-0.059076
H	-6.488767	-0.744398	2.377538
H	-4.123669	-0.883517	3.140775

44

6-A-Me-Cl-1 Eopt
-1311.377860

C	4.620066	-0.889621	-0.149424
C	4.145918	-2.198107	0.005693
C	2.778624	-2.469766	0.135990
C	1.876772	-1.401518	0.104072
C	2.319631	-0.078890	-0.046798
C	3.696611	0.160179	-0.169705
H	4.856255	-3.022136	0.026216
H	0.815357	-1.596657	0.227641
H	4.050107	1.177560	-0.311759
C	6.095695	-0.623698	-0.331455
H	6.700432	-1.288202	0.293848
H	6.399369	-0.789069	-1.372584
H	6.349138	0.409016	-0.074791
C	2.290876	-3.883207	0.348756
H	2.898587	-4.602769	-0.208886
H	2.344446	-4.161285	1.408607
H	1.250135	-3.997226	0.031902
C	1.347693	1.041547	-0.074024
C	0.120848	0.904432	-0.733017
C	1.631662	2.257867	0.572022
C	-0.812601	1.947383	-0.748822
H	-0.109477	-0.024286	-1.245817
C	0.719104	3.313958	0.569299
H	2.567884	2.365260	1.112033
C	-0.505779	3.141486	-0.093997
H	-1.231076	3.952536	-0.094499
C	1.030516	4.614447	1.270874
H	1.964020	4.547322	1.836218
H	1.132784	5.435871	0.551811
H	0.232207	4.891389	1.968231
C	-2.132227	1.775305	-1.477853
H	-1.960952	1.798544	-2.560844
H	-2.778759	2.631602	-1.250935
C	-2.856177	0.489820	-1.127283
C	-3.098443	0.170050	0.216226
C	-3.287284	-0.394018	-2.121522
C	-3.764449	-1.008630	0.536054
H	-2.754502	0.830204	1.005194
C	-3.954677	-1.575328	-1.785219
H	-3.097122	-0.159190	-3.164857
C	-4.200474	-1.895130	-0.450359
H	-4.282220	-2.254429	-2.566458
H	-4.714013	-2.809161	-0.175378
Cl	-4.059060	-1.395663	2.227533

44

6-A-Me-Cl-10 Eopt
-1311.377387

C	-5.489690	0.202433	-0.178638
C	-5.583083	-1.180988	0.001304
C	-4.439499	-1.979005	0.147271
C	-3.185778	-1.365156	0.109262
C	-3.056554	0.022004	-0.072812
C	-4.219370	0.790365	-0.217070
H	-6.564985	-1.649029	0.027301
H	-2.293586	-1.969402	0.245703

H -4.133051 1.859627 -0.388847
C -6.731165 1.052259 -0.311779
H -7.582810 0.462682 -0.663476
H -7.010789 1.492167 0.653579
H -6.575326 1.877700 -1.013402
C -4.569110 -3.471458 0.339242
H -5.223679 -3.708337 1.185324
H -5.001685 -3.947349 -0.548695
H -3.596204 -3.934438 0.525962
C -1.716904 0.658216 -0.113485
C -0.641048 0.025269 -0.759588
C -1.492449 1.900134 0.495216
C 0.623885 0.612099 -0.798000
H -0.802802 -0.927286 -1.255845
C -0.232407 2.511970 0.467414
H -2.306846 2.386265 1.024968
C 0.817624 1.853487 -0.178258
H 1.806625 2.305672 -0.190975
C -0.020263 3.861151 1.111706
H -0.288960 4.671186 0.422335
H -0.638293 3.976138 2.007480
H 1.025520 4.006039 1.397792
C 1.783107 -0.084432 -1.481173
H 2.196810 0.561678 -2.263761
H 1.411925 -0.983875 -1.987326
C 2.898109 -0.474722 -0.526750
C 4.231282 -0.198357 -0.847116
C 2.611893 -1.127388 0.680517
C 5.249200 -0.579447 0.027701
H 4.479532 0.311814 -1.772005
C 3.639833 -1.504519 1.543080
H 1.578549 -1.326400 0.946873
C 4.973964 -1.233641 1.225558
H 3.406700 -2.009872 2.475389
H 5.779923 -1.518538 1.891958
Cl 6.919057 -0.216358 -0.394232
44
6-A-Me-Cl-11 Eopt
-1311.377352
C 4.178163 -1.383785 0.983369
C 3.972535 -2.486576 0.141492
C 2.982735 -2.476812 -0.845353
C 2.189405 -1.331529 -0.989371
C 2.368137 -0.213676 -0.164568
C 3.370211 -0.257144 0.819796
H 4.597766 -3.369208 0.259106
H 1.437120 -1.300905 -1.772618
H 3.502546 0.593353 1.482203
C 5.251022 -1.424375 2.045649
H 5.098942 -2.265274 2.731730
H 6.244851 -1.545756 1.599264
H 5.259455 -0.504984 2.637588
C 2.757161 -3.678188 -1.732406
H 3.616337 -4.354732 -1.714020
H 1.878979 -4.248199 -1.405241
H 2.581999 -3.378859 -2.770877
C 1.515980 0.990316 -0.324412
C 0.150954 0.866354 -0.609114
C 2.059275 2.279965 -0.185828
C -0.662587 1.995985 -0.756336
H -0.292897 -0.122077 -0.677290
C 1.269219 3.422023 -0.325107
H 3.121292 2.389448 0.013570
C -0.096221 3.263402 -0.607797
H -0.725221 4.144970 -0.712801
C 1.867439 4.802771 -0.197688
H 2.889569 4.759251 0.188361
H 1.899225 5.310193 -1.169293
H 1.276489 5.431399 0.477300
C -2.140557 1.836083 -1.064805
H -2.264293 1.476225 -2.093199
H -2.621472 2.820281 -1.022211
C -2.851087 0.886182 -0.121291
C -3.490786 -0.257004 -0.609532
C -2.862629 1.133554 1.258836
C -4.130644 -1.126115 0.275607
H -3.491855 -0.473969 -1.672608
C -3.508009 0.258720 2.130198
H -2.351122 2.009245 1.647065
C -4.150610 -0.885198 1.646316
H -3.511480 0.459816 3.197144
H -4.652158 -1.573955 2.316282
Cl -4.925218 -2.561529 -0.361493
44
6-A-Me-Cl-12 Eopt
-1311.377387
C 4.209369 -0.696180 1.342661
C 4.587173 -1.612576 0.352854
C 3.908913 -1.689701 -0.868878
C 2.834184 -0.823218 -1.095139
C 2.428574 0.103544 -0.124012
C 3.127638 0.152812 1.092058
H 5.426477 -2.279700 0.538276
H 2.318094 -0.852997 -2.050600
H 2.805966 0.846940 1.863122
C 4.968529 -0.617231 2.645927
H 5.242734 -1.612797 3.009276
H 5.897704 -0.046676 2.524998
H 4.376571 -0.123495 3.421889
C 4.313351 -2.703147 -1.913128
H 5.387805 -2.907206 -1.877015
H 3.793048 -3.656261 -1.756229
H 4.066074 -2.356733 -2.921093
C 1.281930 1.010741 -0.375420
C 0.139110 0.552592 -1.040680
C 1.317507 2.349574 0.054553
C -0.948248 1.401795 -1.279107
H 0.077142 -0.488691 -1.343265
C 0.247152 3.216174 -0.169815
H 2.208201 2.722474 0.551749
C -0.884819 2.724895 -0.839027
H -1.729340 3.387393 -1.016891
C 0.304487 4.657596 0.276432
H 1.190872 4.849670 0.887132
H 0.337466 5.336443 -0.583963
H -0.578298 4.925500 0.867365
C -2.187674 0.867343 -1.966668
H -1.921720 0.458047 -2.948006
H -2.878965 1.697850 -2.155846
C -2.908559 -0.208088 -1.171956
C -3.123365 -0.042080 0.202922
C -3.383136 -1.366727 -1.796315
C -3.809138 -1.019941 0.917063
H -2.745575 0.837222 0.712813
C -4.067830 -2.341017 -1.065507
H -3.214467 -1.508679 -2.860049
C -4.288971 -2.176810 0.301951
H -4.428199 -3.237326 -1.561109
H -4.815123 -2.926529 0.881456
Cl -4.071384 -0.794503 2.643023
44
6-A-Me-Cl-13 Eopt
-1311.377784
C 2.502866 -2.644500 0.537820
C 3.843308 -2.631097 0.132936
C 4.450871 -1.463964 -0.345520
C 3.691587 -0.291382 -0.411917
C 2.345319 -0.272196 -0.018114
C 1.765385 -1.459638 0.453781
H 4.425743 -3.548348 0.191230
H 4.145329 0.614750 -0.803334
H 0.731103 -1.451943 0.785561
C 1.878395 -3.903401 1.091173
H 2.248506 -4.793109 0.572010
H 2.115015 -4.023872 2.155631
H 0.788900 -3.882267 0.996507
C 5.884710 -1.482335 -0.819924
H 6.497300 -2.154916 -0.211319
H 5.950343 -1.830135 -1.858328
H 6.331060 -0.484413 -0.778791
C 1.547268 0.975907 -0.098733
C 0.205990 0.939699 -0.495930
C 2.117167 2.218830 0.230653
C -0.560463 2.108964 -0.564089
H -0.246326 -0.009909 -0.764612
C 1.375088 3.399214 0.169547
H 3.148752 2.256359 0.568341
C 0.030773 3.327955 -0.228166
H -0.561364 4.239646 -0.273970
C 1.992565 4.727849 0.535784
H 3.030757 4.608326 0.857299
H 1.981881 5.417070 -0.316544
H 1.441256 5.211181 1.350367
C -2.011974 2.040246 -1.004493
H -2.062105 1.803394 -2.073878
H -2.469016 3.029145 -0.883884
C -2.814655 1.012772 -0.231631
C -3.394203 -0.078870 -0.885031
C -2.963731 1.130043 1.157763
C -4.109894 -1.026859 -0.151692
H -3.289093 -0.195581 -1.958538
C -3.684097 0.178261 1.876297
H -2.500136 1.965258 1.674312
C -4.266116 -0.915200 1.226980
H -3.794972 0.279385 2.951650
H -4.825949 -1.662748 1.777021
Cl -4.826115 -2.395827 -0.994218
44
6-A-Me-Cl-14 Eopt
-1311.377390
C 4.208441 -0.701266 1.339098
C 4.590144 -1.607950 0.340466
C 3.910446 -1.679315 -0.879702
C 2.831529 -0.814557 -1.098433
C 2.426129 0.105229 -0.121912
C 3.127591 0.148881 1.094004
H 5.435523 -2.269551 0.517958
H 2.316897 -0.836701 -2.054977
H 2.809003 0.841614 1.867521
C 4.940226 -0.665576 2.659786
H 4.715928 -1.556264 3.258919
H 6.025212 -0.638189 2.512219
H 4.656041 0.210849 3.248824
C 4.318682 -2.678486 -1.936177
H 5.371679 -2.957735 -1.836051
H 3.725142 -3.597739 -1.856282
H 4.166629 -2.278115 -2.943337

C 1.281003 1.015800 -0.368078
C 0.137111 0.562530 -1.034798
C 1.318940 2.352487 0.068276
C -0.949056 1.414532 -1.268641
H 0.073447 -0.477261 -1.342138
C 0.249778 3.221755 -0.151403
H 2.210449 2.721647 0.566794
C -0.883363 2.735396 -0.822251
H -1.727013 3.400008 -0.996304
C 0.309710 4.661045 0.301340
H 1.194964 4.848119 0.915230
H 0.346886 5.343533 -0.556013
H -0.573963 4.928748 0.891010
C -2.189436 0.885513 -1.958877
H -1.925115 0.486505 -2.944897
H -2.882036 1.717103 -2.138120
C -2.907023 -0.198736 -1.173395
C -3.124723 -0.043439 0.202293
C -3.375032 -1.355207 -1.806622
C -3.806817 -1.029578 0.908496
H -2.751916 0.834080 0.718913
C -4.056083 -2.337940 -1.083719
H -3.204101 -1.488808 -2.871074
C -4.280092 -2.184413 0.284499
H -4.411401 -3.232458 -1.586156
H -4.803516 -2.940719 0.857884
Cl -4.072787 -0.817581 2.635564
44
6-A-Me-Cl-2 Eopt
-1311.377352
C 4.178241 -1.383744 0.983442
C 3.972711 -2.486510 0.141526
C 2.982979 -2.476748 -0.845401
C 2.189630 -1.331494 -0.989446
C 2.368245 -0.213663 -0.164572
C 3.370233 -0.257131 0.819867
H 4.597954 -3.369132 0.259169
H 1.437397 -1.300872 -1.772741
H 3.502466 0.593333 1.482336
C 5.251216 -1.424198 2.045611
H 5.100942 -2.266749 2.730044
H 6.245277 -1.542619 1.598925
H 5.257797 -0.505932 2.639318
C 2.757454 -3.678180 -1.732392
H 3.617447 -4.353728 -1.715483
H 1.880461 -4.249310 -1.403981
H 2.580327 -3.378820 -2.770514
C 1.516033 0.990289 -0.324428
C 0.151029 0.866259 -0.609235
C 2.059238 2.279957 -0.185735
C -0.662569 1.995843 -0.756443
H -0.292758 -0.122196 -0.677481
C 1.269122 3.421980 -0.325018
H 3.121233 2.389493 0.013761
C -0.096286 3.263292 -0.607797
H -0.725335 4.144826 -0.712774
C 1.867313 4.802745 -0.197650
H 2.888731 4.759403 0.190305
H 1.900992 5.309317 -1.169641
H 1.275211 5.432042 0.475691
C -2.140519 1.835873 -1.064957
H -2.264206 1.475902 -2.093317
H -2.621462 2.820063 -1.022487
C -2.851075 0.886059 -0.121371
C -3.490956 -0.257049 -0.609547
C -2.862486 1.133453 1.258757
C -4.130864 -1.126063 0.275653
H -3.492130 -0.474036 -1.672620
C -3.507918 0.258717 2.130177
H -2.350837 2.009084 1.646932
C -4.150703 -0.885126 1.646357
H -3.511289 0.459824 3.197120
H -4.652291 -1.573807 2.316372
Cl -4.925668 -2.561379 -0.361373
44
6-A-Me-Cl-3 Eopt
-1311.377847
C -4.614906 -0.900958 -0.156307
C -4.137123 -2.206188 -0.005856
C -2.766968 -2.473895 0.127867
C -1.870579 -1.403809 0.103513
C -2.317932 -0.080648 -0.050272
C -3.693040 0.153237 -0.178987
H -4.844145 -3.033162 0.006750
H -0.808794 -1.594953 0.228517
H -4.049466 1.168796 -0.327888
C -6.095107 -0.625655 -0.277313
H -6.640728 -1.514571 -0.606916
H -6.517361 -0.317802 0.687317
H -6.293411 0.179818 -0.991589
C -2.279027 -3.891897 0.307211
H -2.646815 -4.321193 1.246488
H -2.631734 -4.537897 -0.504652
H -1.186706 -3.937042 0.324222
C -1.348188 1.041664 -0.078232
C -0.118585 0.904873 -0.732183
C -1.637231 2.259869 0.562031
C 0.812361 1.950053 -0.749003
H 0.115984 -0.025250 -1.240425
C -0.727287 3.318194 0.558192
H -2.575529 2.367097 1.098455
C 0.500302 3.146060 -0.100167
H 1.223661 3.958848 -0.101323
C -1.043794 4.620368 1.254349
H -1.134328 5.441386 0.533309
H -1.984564 4.556531 1.807896
H -0.253627 4.895591 1.961655
C 2.134770 1.778624 -1.473252
H 2.780367 2.634778 -1.243153
H 1.967464 1.802948 -2.556852
C 2.857443 0.492849 -1.121319
C 3.096898 0.172937 0.222667
C 3.289753 -0.391359 -2.114693
C 3.761304 -1.006284 0.543809
H 2.751991 0.833381 1.010971
C 3.955536 -1.573206 -1.777081
H 3.101713 -0.156445 -3.158391
C 4.198489 -1.893174 -0.441748
H 4.283991 -2.252610 -2.557671
H 4.710739 -2.807625 -0.165757
Cl 4.052279 -1.393562 2.235864
44
6-A-Me-Cl-4 Eopt
-1311.377767
C -4.448786 -1.470070 -0.348768
C -3.837217 -2.638118 0.117983
C -2.495259 -2.650209 0.522989
C -1.762546 -1.463468 0.447945
C -2.345371 -0.274449 -0.020533
C -3.689984 -0.295476 -0.415225
H -4.415151 -3.558856 0.164955
H -0.727299 -1.455421 0.776248
H -4.144097 0.609321 -0.809481
C -5.902335 -1.468995 -0.759302
H -6.222203 -2.458826 -1.098243
H -6.547228 -1.185198 0.081753
H -6.087477 -0.755609 -1.568253
C -1.865178 -3.918683 1.047392
H -2.265852 -4.179605 2.034321
H -2.064684 -4.765468 0.381878
H -0.781227 -3.813328 1.145398
C -1.547897 0.973960 -0.101746
C -0.206226 0.938642 -0.497780
C -2.119132 2.216802 0.225797
C 0.559135 2.108570 -0.566798
H 0.247409 -0.010722 -0.765017
C -1.378252 3.397866 0.163603
H -3.150960 2.253747 0.562804
C -0.033553 3.327418 -0.232952
H 0.557773 4.239607 -0.279325
C -1.997485 4.726423 0.527141
H -1.990443 5.412833 -0.327508
H -3.034622 4.605837 0.851658
H -1.445073 5.213597 1.338663
C 2.011138 2.040583 -1.005688
H 2.467011 3.030201 -0.886567
H 2.062570 1.801676 -2.074551
C 2.814101 1.015451 -0.229994
C 3.395416 -0.076888 -0.880655
C 2.961600 1.135594 1.159322
C 4.111281 -1.022735 -0.144713
H 3.291539 -0.195821 -1.954035
C 3.682157 0.185942 1.880469
H 2.496630 1.971352 1.673752
C 4.265941 -0.908213 1.233905
H 3.791804 0.289280 2.955740
H 4.825948 -1.654119 1.785992
Cl 4.829785 -2.392598 -0.983796
44
6-A-Me-Cl-5 Eopt
-1311.377352
C -2.991970 -2.472480 -0.844358
C -3.975510 -2.483207 0.149414
C -4.176896 -1.380746 0.991890
C -3.371035 -0.252843 0.822672
C -2.371660 -0.210952 -0.163845
C -2.195192 -1.330176 -0.988000
H -4.597393 -3.367455 0.272819
H -3.499020 0.596978 1.486865
H -1.441378 -1.302552 -1.769849
C -2.813214 -3.651499 -1.771195
H -3.209283 -4.569978 -1.328336
H -3.339561 -3.488459 -2.719815
H -1.757306 -3.815586 -2.008093
C -5.226685 -1.429943 2.076640
H -6.195668 -1.749157 1.677568
H -4.948853 -2.142016 2.862866
H -5.360125 -0.451232 2.545897
C -1.516435 0.990815 -0.324132
C -0.151800 0.863892 -0.609256
C -2.057041 2.281602 -0.185618
C 0.664029 1.991857 -0.756851
H 0.289982 -0.125471 -0.677534
C -1.264681 3.421991 -0.325287
H -3.118800 2.393214 0.014040
C 0.100355 3.260473 -0.608328
H 0.731163 4.140701 -0.713707
C -1.859947 4.804008 -0.197815

H	-1.888574	5.312385	-1.169014
H	-2.882941	4.762524	0.186160
H	-1.268887	5.430656	0.478928
C	2.141469	1.828893	-1.065884
H	2.624150	2.812326	-1.024850
H	2.264004	1.467629	-2.093930
C	2.851417	0.879014	-0.121837
C	3.497573	-0.260327	-0.610560
C	2.857118	1.123088	1.258922
C	4.138124	-1.128808	0.274682
H	3.503179	-0.474778	-1.674138
C	3.503198	0.248875	2.130399
H	2.340535	1.995638	1.647474
C	4.152350	-0.891118	1.646015
H	3.502052	0.447362	3.197836
H	4.654418	-1.579400	2.316085
Cl	4.940994	-2.559281	-0.363135

44

6-A-Me-Cl-6 Eopt

-1311.377401

C	-3.918414	-1.666156	-0.887331
C	-4.586762	-1.611520	0.342042
C	-4.198947	-0.715992	1.345749
C	-3.121128	0.140783	1.100181
C	-2.427978	0.108484	-0.118822
C	-2.839256	-0.803703	-1.102415
H	-5.426003	-2.280408	0.521407
H	-2.795443	0.823352	1.879983
H	-2.326349	-0.820734	-2.059868
C	-4.380818	-2.609703	-1.972224
H	-4.719786	-3.562572	-1.553554
H	-5.220885	-2.181900	-2.533293
H	-3.579545	-2.817995	-2.687205
C	-4.902146	-0.706025	2.682324
H	-5.967548	-0.931837	2.573950
H	-4.474549	-1.458925	3.355988
H	-4.808184	0.266195	3.175048
C	-1.280781	1.017246	-0.362122
C	-0.140041	0.565453	-1.035270
C	-1.313613	2.350623	0.084337
C	0.948312	1.415743	-1.264922
H	-0.080400	-0.472176	-1.350666
C	-0.242367	3.218359	-0.131586
H	-2.202781	2.718503	0.588021
C	0.887746	2.733483	-0.808462
H	1.733180	3.396660	-0.979240
C	-0.298281	4.655114	0.329624
H	-0.366098	5.340968	-0.523265
H	-1.166178	4.833523	0.970334
H	0.600815	4.926465	0.893544
C	2.186036	0.887734	-1.960403
H	2.877925	1.719590	-2.141349
H	1.918088	0.489845	-2.945888
C	2.907274	-0.197370	-1.179196
C	3.119678	-0.050122	0.198149
C	3.384892	-1.346430	-1.818856
C	3.806086	-1.036662	0.899715
H	2.739573	0.821329	0.719652
C	4.070144	-2.329567	-1.100613
H	3.218057	-1.473899	-2.884716
C	4.288901	-2.183999	0.269372
H	4.432801	-3.218316	-1.608018
H	4.815421	-2.940850	0.839195
Cl	4.065098	-0.834792	2.629077

44

6-A-Me-Cl-7 Eopt

-1311.377502

C	-4.843515	-1.558827	-0.568208
C	-5.603253	-0.954408	0.438880
C	-5.114586	0.138094	1.168153
C	-3.839359	0.625301	0.870922
C	-3.049572	0.039289	-0.131537
C	-3.566585	-1.054188	-0.840024
H	-6.595485	-1.341482	0.661569
H	-3.439011	1.455280	1.445971
H	-2.976032	-1.501316	-1.634795
C	-5.403727	-2.705632	-1.375759
H	-6.130581	-3.283258	-0.796826
H	-5.915663	-2.339290	-2.274271
H	-4.612285	-3.385811	-1.704962
C	-5.940897	0.751057	2.273894
H	-6.972917	0.923350	1.950296
H	-5.980457	0.090837	3.148718
H	-5.523429	1.708102	2.599065
C	-1.695250	0.565202	-0.432267
C	-0.633296	-0.310298	-0.718126
C	-1.442813	1.943193	-0.432673
C	0.645545	0.172884	-0.995282
H	-0.805191	-1.382560	-0.692995
C	-0.167929	2.453585	-0.710715
H	-2.259124	2.632466	-0.235497
C	0.867453	1.556286	-0.985471
H	1.866968	1.936020	-1.184192
C	0.069863	3.944446	-0.745814
H	-0.209351	4.363150	-1.720562
H	-0.525238	4.461118	0.013603
H	1.123441	4.183123	-0.574190
C	1.794205	-0.777975	-1.264065
H	2.235100	-0.559773	-2.243445
H	1.405242	-1.801887	-1.321138
C	2.885806	-0.718105	-0.209970
C	4.229060	-0.623743	-0.588113
C	2.566654	-0.767855	1.154252
C	5.224641	-0.588223	0.388803
H	4.502533	-0.577063	-1.637080
C	3.572590	-0.734497	2.118470
H	1.525435	-0.820877	1.456689
C	4.916802	-0.643448	1.745516
H	3.314161	-0.774759	3.172289
H	5.705806	-0.612184	2.488006
Cl	6.907946	-0.463883	-0.110673

44

6-A-Me-Cl-8 Eopt

-1311.377539

C	4.533761	-0.660749	1.437924
C	5.158058	-1.142560	0.282375
C	4.583540	-0.973315	-0.984520
C	3.360838	-0.304049	-1.081519
C	2.706484	0.188506	0.058923
C	3.306071	-0.000857	1.311871
H	6.110505	-1.661417	0.369302
H	2.892385	-0.188863	-2.054721
H	2.821955	0.396632	2.199565
C	5.189590	-0.811557	2.790072
H	5.822445	-1.703287	2.828860
H	5.825957	0.053100	3.016036
H	4.444995	-0.887134	3.588332
C	5.258163	-1.536764	-2.212700
H	6.332659	-1.325560	-2.208740
H	5.140640	-2.626147	-2.261333
H	4.832931	-1.116809	-3.128581
C	1.405496	0.891662	-0.059802
C	0.384236	0.673327	0.881461
C	1.162873	1.782083	-1.113287
C	-0.845501	1.322525	0.775285
H	0.545368	-0.037209	1.687125
C	-0.062961	2.449213	-1.240472
H	1.950839	1.977066	-1.835452
C	-1.058917	2.206117	-0.291404
H	-2.022406	2.701459	-0.386733
C	-0.285267	3.432853	-2.364468
H	0.187793	4.397089	-2.141545
H	0.144318	3.068636	-3.303130
H	-1.351088	3.616694	-2.526824
C	-1.957738	1.045322	1.764335
H	-2.307289	1.986318	2.204430
H	-1.559297	0.446743	2.592964
C	-3.149482	0.321520	1.159769
C	-2.953051	-0.731726	0.256570
C	-4.456588	0.677644	1.510729
C	-4.052682	-1.407218	-0.264819
H	-1.950370	-1.013243	-0.045363
C	-5.550244	-0.008585	0.977408
H	-4.619757	1.497934	2.204075
C	-5.358913	-1.061306	0.082069
H	-6.559464	0.279581	1.255441
H	-6.198664	-1.599471	-0.342161
Cl	-3.787709	-2.727919	-1.398020

44

6-A-Me-Cl-9 Eopt

-1311.377514

C	5.117835	0.038293	0.031328
C	5.049443	1.425135	-0.132932
C	3.821470	2.085096	-0.282946
C	2.648503	1.327428	-0.268848
C	2.682354	-0.067955	-0.108948
C	3.925874	-0.696581	0.038393
H	5.969858	2.005315	-0.145805
H	1.689853	1.829789	-0.359946
H	3.967936	-1.778108	0.132583
C	6.446774	-0.655487	0.215253
H	7.264996	-0.071929	-0.216795
H	6.670426	-0.798693	1.279743
H	6.448365	-1.644072	-0.254369
C	3.776155	3.585302	-0.451293
H	4.247151	4.094270	0.397267
H	4.311886	3.897947	-1.355067
H	2.746848	3.945791	-0.529704
C	1.426639	-0.857835	-0.098536
C	0.372302	-0.540399	-0.971749
C	1.260364	-1.932184	0.785499
C	-0.816475	-1.270616	-0.963513
H	0.492471	0.275697	-1.678412
C	0.078552	-2.683912	0.810234
H	2.055289	-2.169705	1.486826
C	-0.953540	-2.337651	-0.066418
H	-1.885118	-2.898652	-0.045802
C	-0.068581	-3.850942	1.757169
H	0.356722	-4.764240	1.322845
H	0.452097	-3.664990	2.701536
H	-1.120156	-4.051812	1.981582
C	-1.960353	-0.898872	-1.881964
H	-2.305481	-1.785009	-2.426494
H	-1.596995	-0.193610	-2.639918
C	-3.148550	-0.279549	-1.163147
C	-2.949982	0.615869	-0.104069
C	-4.456930	-0.572012	-1.565816

C -4.048239 1.203019 0.518314
H -1.947090 0.842419 0.239834
C -5.548659 0.023350 -0.930069
H -4.622148 -1.271267 -2.380679
C -5.355053 0.920467 0.121122
H -6.558562 -0.214627 -1.250017
H -6.193543 1.386467 0.625620
Cl -3.779071 2.326426 1.846635
46
6-B-2Me-1 Eopt
-965.126357
C -4.171773 -1.112229 0.530843
C -3.435770 -2.193317 -0.003262
C -2.133621 -2.063757 -0.496371
C -1.602285 -0.778050 -0.428043
C -2.287907 0.328704 0.098138
C -3.591248 0.156584 0.582244
H -3.900819 -3.176242 -0.031532
H -4.143993 0.995837 0.994303
C -5.575014 -1.344520 1.041671
H -6.230808 -1.721697 0.248424
H -5.587342 -2.082881 1.851738
H -6.013969 -0.419712 1.426148
C -1.340401 -3.213671 -1.055916
H -0.416015 -3.365301 -0.487162
H -1.920321 -4.139519 -1.024156
H -1.048810 -3.024838 -2.095194
C -1.364700 1.445869 -0.010875
C -0.201366 0.917653 -0.594541
C -1.403852 2.805509 0.324647
C 0.939806 1.669239 -0.865131
C -0.285611 3.601530 0.068610
H -2.291196 3.237618 0.778088
C 0.860137 3.021140 -0.518719
H 1.724160 3.653178 -0.711693
C -0.285659 5.073510 0.410612
H -1.233441 5.373641 0.865996
H 0.516973 5.318943 1.115606
H -0.133423 5.690337 -0.482671
C 2.182772 1.040848 -1.454275
H 1.914659 0.464735 -2.346520
H 2.860946 1.838369 -1.775298
C 2.890693 0.130032 -0.465679
C 2.765512 -1.258965 -0.558123
C 3.646223 0.672828 0.581129
C 3.374022 -2.116721 0.367576
H 2.162989 -1.680712 -1.358154
C 4.260367 -0.165601 1.511042
H 3.742476 1.751459 0.672304
C 4.123614 -1.552181 1.406729
H 4.843557 0.260513 2.322328
H 4.599651 -2.199546 2.138663
C 3.239644 -3.614408 0.228228
H 4.020569 -4.021690 -0.426091
H 3.329292 -4.114909 1.197065
H 2.273728 -3.887603 -0.208221
O -0.336294 -0.431267 -0.858397
46
6-B-2Me-2 Eopt
-965.126236
C -4.235221 -0.923918 0.647919
C -3.607819 -2.056322 0.082147
C -2.327855 -2.022757 -0.479972
C -1.701894 -0.778691 -0.445818
C -2.278638 0.376144 0.107533
C -3.563760 0.300038 0.660368
H -4.143347 -3.003137 0.084166
H -4.032680 1.178255 1.094637
C -5.622567 -1.053764 1.233082
H -6.344897 -1.383294 0.477256
H -5.645277 -1.788729 2.046023
H -5.972835 -0.099337 1.635864
C -1.652830 -3.226727 -1.079120
H -0.691065 -3.420899 -0.592071
H -2.280240 -4.115850 -0.976766
H -1.444369 -3.072824 -2.143957
C -1.285167 1.423763 -0.057575
C -0.195310 0.810784 -0.697337
C -1.207910 2.783821 0.270935
C 0.982368 1.476802 -1.029546
C -0.049477 3.495722 -0.046003
H -2.036902 3.280305 0.766996
C 1.020552 2.830998 -0.685715
H 1.919859 3.395845 -0.921504
C 0.074057 4.965179 0.284503
H -0.811191 5.326967 0.814804
H 0.947093 5.160073 0.917855
H 0.190147 5.569213 -0.622978
C 2.137973 0.756229 -1.680333
H 1.802975 0.287692 -2.612555
H 2.900499 1.495122 -1.954858
C 2.757208 -0.312055 -0.793026
C 3.075390 -0.028466 0.541678
C 3.022029 -1.589775 -1.291522
C 3.654329 -0.986834 1.379262
H 2.851325 0.958194 0.939687
C 3.599844 -2.560822 -0.468985
H 2.764022 -1.830394 -2.319223
C 3.913161 -2.262951 0.856329
H 3.796963 -3.554161 -0.862029
H 4.355928 -3.024579 1.493306
C 4.004339 -0.656749 2.810759
H 3.646342 -1.432007 3.496707
H 5.090326 -0.580828 2.944447
H 3.563854 0.295950 3.117799
O -0.437917 -0.524933 -0.942622
46
6-B-2Me-3 Eopt
-965.125936
C 5.205299 0.219175 0.225470
C 4.994612 -1.175408 0.150475
C 3.727461 -1.753768 0.023524
C 2.668245 -0.849905 -0.024811
C 2.823359 0.544175 0.045304
C 4.110079 1.083356 0.172140
H 5.858209 -1.835395 0.193194
H 4.256333 2.158138 0.228268
C 6.611370 0.755947 0.362577
H 7.092682 0.384705 1.274781
H 7.238902 0.450940 -0.482825
H 6.615136 1.848681 0.404129
C 3.502932 -3.239593 -0.056710
H 3.003274 -3.513592 -0.992568
H 4.451629 -3.779589 -0.002235
H 2.860920 -3.586866 0.760482
C 1.480235 1.091477 -0.041288
C 0.627736 -0.017844 -0.155755
C 0.927322 2.378951 -0.032965
C -0.758685 0.073927 -0.262930
C -0.456968 2.521709 -0.138603
H 1.564475 3.254217 0.054865
C -1.272540 1.373300 -0.250786
H -2.347285 1.504082 -0.329313
C -1.099520 3.889543 -0.133193
H -0.348941 4.679640 -0.041843
H -1.663971 4.067590 -1.055733
H -1.802035 3.995573 0.701490
C -1.601656 -1.182661 -0.388956
H -1.316842 -1.701869 -1.312527
H -1.331276 -1.863207 0.427537
C -3.091510 -0.939078 -0.380670
C -3.783861 -0.828683 0.831789
C -3.798750 -0.776014 -1.576488
C -5.156910 -0.561560 0.876273
H -3.235027 -0.947116 1.763694
C -5.169007 -0.505738 -1.552761
H -3.274133 -0.853036 -2.525018
C -5.842807 -0.397485 -0.335919
H -5.711557 -0.377798 -2.484979
H -6.908908 -0.185689 -0.324204
C -5.888215 -0.481151 2.194896
H -6.660807 0.294074 2.175627
H -6.385043 -1.431340 2.426912
H -5.202873 -0.258356 3.017809
O 1.337226 -1.204978 -0.147651
46
6-B-2Me-4 Eopt
-965.126074
C -4.619649 -0.424124 0.720400
C -4.206749 -1.643730 0.139788
C -2.946161 -1.830404 -0.436521
C -2.110540 -0.716349 -0.401072
C -2.471191 0.517253 0.164249
C -3.743547 0.662864 0.731878
H -4.900736 -2.481318 0.139374
H -4.046562 1.606658 1.175819
C -6.002017 -0.313031 1.320890
H -6.778674 -0.506904 0.572052
H -6.144078 -1.037365 2.131237
H -6.175740 0.685529 1.731333
C -2.503317 -3.130397 -1.051797
H -1.626031 -3.533952 -0.533589
H -3.301648 -3.875300 -1.004165
H -2.219739 -2.995041 -2.101387
C -1.315383 1.380130 -0.011695
C -0.353708 0.594796 -0.669577
C -1.007766 2.706513 0.315114
C 0.909382 1.057736 -1.026782
C 0.247373 3.216936 -0.024570
H -1.734233 3.332914 0.824566
C 1.177015 2.387366 -0.686935
H 2.150583 2.797682 -0.945673
C 0.621187 4.643007 0.307467
H -0.202276 5.162064 0.805706
H 1.492007 4.681629 0.972101
H 0.876139 5.209041 -0.595864
C 1.931352 0.163908 -1.695913
H 1.420796 -0.515695 -2.385823
H 2.609428 0.784265 -2.290199
C 2.733419 -0.642413 -0.689272
C 3.977622 -0.182684 -0.244571
C 2.223894 -1.834521 -0.159161
C 4.723655 -0.881251 0.713376
H 4.375976 0.744067 -0.652617
C 2.952431 -2.541005 0.798331
H 1.253624 -2.193601 -0.487053
C 4.192335 -2.068393 1.233458
H 2.551355 -3.462812 1.210013

H 4.752030 -2.624088 1.981393
C 6.080565 -0.381832 1.149094
H 6.294138 -0.662032 2.185116
H 6.875792 -0.806960 0.523978
H 6.149046 0.707478 1.070417
O -0.826553 -0.682313 -0.912976
46
6-B-2Me-5 Eopt
-965.126357
C -4.419415 -0.623288 0.562149
C -3.911322 -1.759846 -0.104996
C -2.630718 -1.811151 -0.665017
C -1.876909 -0.648619 -0.520725
C -2.333309 0.506579 0.133926
C -3.622561 0.516963 0.681601
H -4.544243 -2.640497 -0.188875
H -3.999108 1.397831 1.193371
C -5.816682 -0.657133 1.136917
H -6.564609 -0.837048 0.356025
H -5.923242 -1.456618 1.879161
H -6.065894 0.288409 1.626398
C -2.086196 -3.022437 -1.372897
H -1.188749 -3.403014 -0.872091
H -2.828822 -3.823834 -1.400078
H -1.798854 -2.782972 -2.402676
C -1.237366 1.457767 0.057212
C -0.210683 0.797151 -0.638448
C -1.028448 2.769308 0.500734
C 1.025334 1.370983 -0.921200
C 0.195864 3.388862 0.238399
H -1.806376 3.300458 1.041445
C 1.194230 2.681724 -0.464498
H 2.143964 3.174809 -0.659451
C 0.464068 4.803352 0.697616
H -0.400602 5.219893 1.221629
H 1.320860 4.844182 1.380006
H 0.691187 5.461058 -0.149296
C 2.121928 0.607001 -1.632143
H 1.672610 -0.064001 -2.371626
H 2.753635 1.315812 -2.176252
C 2.979037 -0.196168 -0.669082
C 2.503020 -1.400060 -0.131999
C 4.236251 0.269512 -0.272469
C 3.253153 -2.140090 0.787712
H 1.521716 -1.757146 -0.431536
C 4.998071 -0.456793 0.646666
H 4.618910 1.201590 -0.679745
C 4.509944 -1.651574 1.174650
H 5.973171 -0.088066 0.951907
H 5.105785 -2.210534 1.891726
C 2.732003 -3.448336 1.333244
H 3.179903 -4.300817 0.807588
H 2.969485 -3.559962 2.396126
H 1.646756 -3.522048 1.218126
O -0.589157 -0.484965 -0.997000
43
6-B-Me-1 Eopt
-925.799939
C -4.147252 -0.681298 0.492859
C -3.557225 -1.868522 0.004740
C -2.238783 -1.935729 -0.456423
C -1.533904 -0.735421 -0.402951
C -2.070963 0.470371 0.076502
C -3.396658 0.495324 0.528975
H -4.154534 -2.777444 -0.012641
H -3.836251 1.414726 0.904683
C -5.580922 -0.701916 0.970477
H -6.264447 -1.000917 0.167346
H -5.716732 -1.412632 1.793849
H -5.893735 0.284032 1.325259
C -1.603871 -3.198736 -0.972169
H -0.700630 -3.444759 -0.403236
H -2.298043 -4.040072 -0.902254
H -1.300649 -3.088660 -2.019563
C -0.999140 1.445220 -0.035889
C 0.091992 0.743835 -0.574448
C -0.855603 2.806523 0.263459
C 1.332504 1.322182 -0.833596
C 0.368109 3.432146 0.017490
H -1.684181 3.370573 0.681845
C 1.435409 2.681157 -0.523324
H 2.383957 3.180618 -0.708274
C 0.564722 4.899682 0.319856
H -0.336409 5.337178 0.758361
H 1.389512 5.054975 1.024834
H 0.803079 5.465207 -0.588496
C 2.483170 0.508626 -1.377071
H 2.175237 0.004729 -2.299805
H 3.299325 1.190201 -1.642978
C 2.985524 -0.533490 -0.391570
C 2.985747 -1.892815 -0.719821
C 3.449586 -0.146583 0.872783
C 3.446066 -2.848441 0.190066
H 2.612357 -2.206015 -1.690915
C 3.911012 -1.096445 1.784509
H 3.437359 0.905068 1.146385
C 3.911047 -2.453037 1.445653
H 4.267544 -0.779343 2.760227
H 4.267490 -3.193564 2.155316
O -0.221322 -0.579950 -0.806147
H 3.436915 -3.900112 -0.081406
43
6-B-Me-2 Eopt
-925.799929
C -4.373470 -0.338687 0.575414
C -3.966386 -1.573288 0.023162
C -2.684963 -1.798312 -0.490133
C -1.821962 -0.706973 -0.420773
C -2.175897 0.540345 0.117992
C -3.469764 0.724512 0.622201
H -4.682209 -2.391758 -0.006013
H -3.768171 1.679614 1.044511
C -5.779598 -0.186034 1.107680
H -6.524286 -0.367896 0.324192
H -5.978958 -0.898024 1.916890
H -5.946745 0.820838 1.500108
C -2.249395 -3.114008 -1.076593
H -1.407319 -3.537030 -0.517187
H -3.068649 -3.837205 -1.060657
H -1.915296 -2.993763 -2.113068
C -0.990119 1.370897 -0.007490
C -0.019219 0.555324 -0.613107
C -0.662646 2.690745 0.325702
C 1.271449 0.981574 -0.911201
C 0.621359 3.164620 0.045179
H -1.395573 3.340245 0.795390
C 1.559140 2.305678 -0.565809
H 2.555677 2.686801 -0.777523
C 1.017694 4.581882 0.388553
H 0.183598 5.128219 0.837465
H 1.851414 4.600283 1.099948
H 1.338429 5.133275 -0.502730
C 2.300128 0.056827 -1.526245
H 1.796857 -0.643994 -2.200305
H 2.994324 0.648631 -2.130782
C 3.079024 -0.716529 -0.476699
C 2.496477 -1.807504 0.184034
C 4.378884 -0.334252 -0.126195
C 3.199295 -2.497037 1.172841
H 1.485868 -2.107068 -0.076046
C 5.085657 -1.022721 0.863465
H 4.842648 0.508373 -0.632710
C 4.496973 -2.106924 1.517066
H 6.094423 -0.713004 1.120665
H 5.043814 -2.644554 2.285788
O -0.513759 -0.711537 -0.869650
H 2.734260 -3.340489 1.675004
43
6-B-Me-Cl-1 Eopt
-1385.399415
C -3.915610 -1.896171 0.508436
C -2.934663 -2.764660 -0.020524
C -1.700943 -2.321855 -0.507788
C -1.502996 -0.944960 -0.438569
C -2.439203 -0.038778 0.084476
C -3.663696 -0.523932 0.562188
H -3.144775 -3.831509 -0.048157
H -4.406518 0.154294 0.971864
C -5.222043 -2.465030 1.011929
H -5.764934 -2.983607 0.213241
H -5.056863 -3.190764 1.816545
H -5.872833 -1.677046 1.400932
C -0.646298 -3.242287 -1.060396
H 0.289391 -3.156266 -0.496970
H -0.977926 -4.282822 -1.019492
H -0.414826 -2.996646 -2.103065
C -1.812862 1.269375 -0.014815
C -0.552347 1.039970 -0.590508
C -2.177837 2.577888 0.327398
C 0.379718 2.044342 -0.840691
C -1.279521 3.620370 0.089002
H -3.146370 2.782275 0.774384
C -0.021410 3.335557 -0.486024
H 0.669887 4.156755 -0.662077
C -1.636088 5.047032 0.437144
H -2.623685 5.105270 0.902830
H -0.910682 5.480668 1.135004
H -1.649245 5.683489 -0.455286
C 1.742502 1.732282 -1.418949
H 1.627213 1.170135 -2.352083
H 2.245088 2.672505 -1.667094
C 2.598970 0.929952 -0.454765
C 2.732920 -0.453103 -0.612772
C 3.222534 1.554398 0.632575
C 3.471767 -1.185147 0.313778
H 2.243090 -0.958467 -1.436513
C 3.967369 0.809194 1.546859
H 3.115805 2.627058 0.766583
C 4.097907 -0.573915 1.397832
H 4.449725 1.302813 2.384964
H 4.669229 -1.164170 2.104972
O -0.356346 -0.298664 -0.861477
Cl 3.603586 -2.929870 0.109785
43
6-B-Me-Cl-2 Eopt
-1385.398792
C -4.326563 -1.068537 0.790259
C -3.701469 -2.160206 0.147308

C -2.466877 -2.063280 -0.502197
C -1.884654 -0.798148 -0.473975
C -2.461207 0.318351 0.152975
C -3.699861 0.178930 0.792460
H -4.201617 -3.126063 0.158046
H -4.167131 1.026491 1.285303
C -5.662432 -1.266919 1.468363
H -6.423865 -1.607170 0.757070
H -5.598581 -2.020955 2.261338
H -6.018820 -0.336917 1.919816
C -1.795010 -3.224789 -1.183127
H -0.801181 -3.407327 -0.759964
H -2.389736 -4.135479 -1.076106
H -1.654887 -3.028340 -2.252089
C -1.520713 1.407450 -0.050045
C -0.458443 0.854418 -0.783569
C -1.468152 2.759495 0.313723
C 0.666949 1.574594 -1.177139
C -0.361357 3.523890 -0.061005
H -2.276129 3.209874 0.882897
C 0.683136 2.918932 -0.794949
H 1.542855 3.523576 -1.075298
C -0.265978 4.986047 0.308290
H -1.128135 5.300614 0.902666
H 0.637445 5.191360 0.893782
H -0.225812 5.620557 -0.584708
C 1.796234 0.916572 -1.930878
H 1.412980 0.461372 -2.850856
H 2.512844 1.688652 -2.235758
C 2.517884 -0.153075 -1.126638
C 2.854947 0.080356 0.212305
C 2.865432 -1.375252 -1.711069
C 3.532154 -0.899125 0.933486
H 2.578297 1.012714 0.692233
C 3.544855 -2.348040 -0.974977
H 2.593432 -1.570373 -2.744285
C 3.886296 -2.119462 0.358716
H 3.805922 -3.294710 -1.438201
H 4.409380 -2.868109 0.942408
O -0.668166 -0.482498 -1.050854
Cl 3.946296 -0.590177 2.616391
43
6-B-Me-Cl-3 Eopt
-1385.398042
C 5.478358 0.193030 0.334978
C 5.259951 -1.199450 0.243756
C 3.993802 -1.767695 0.070113
C 2.943858 -0.855293 -0.008462
C 3.106652 0.536973 0.076008
C 4.392102 1.065854 0.250087
H 6.116718 -1.866129 0.311697
H 4.544146 2.139078 0.318454
C 6.882801 0.717995 0.523892
H 7.328198 0.339687 1.451268
H 7.537427 0.410640 -0.299767
H 6.893843 1.810473 0.569621
C 3.761374 -3.251316 -0.026344
H 3.288723 -3.516144 -0.978705
H 4.704133 -3.798381 0.053656
H 3.092596 -3.598940 0.768937
C 1.771324 1.095169 -0.052329
C 0.915457 -0.007063 -0.203017
C 1.227465 2.386429 -0.053588
C -0.465545 0.096763 -0.356609
C -0.151337 2.540927 -0.204804
H 1.867457 3.256255 0.062313
C -0.970924 1.399510 -0.353186
H -2.041017 1.541291 -0.467472
C -0.783844 3.913323 -0.209401
H -0.030349 4.697073 -0.092249
H -1.320669 4.101021 -1.146355
H -1.508467 4.019682 0.606027
C -1.309599 -1.155242 -0.516149
H -0.976890 -1.689935 -1.414096
H -1.096833 -1.827358 0.323852
C -2.794580 -0.898749 -0.601522
C -3.554560 -0.780851 0.568790
C -3.426643 -0.736203 -1.839885
C -4.917472 -0.504818 0.484448
H -3.084839 -0.897121 1.539880
C -4.793558 -0.460934 -1.909966
H -2.842984 -0.822316 -2.751803
C -5.554273 -0.341942 -0.745454
H -5.274100 -0.339321 -2.875877
H -6.616607 -0.130877 -0.786920
O 1.614410 -1.199370 -0.178838
Cl -5.856507 -0.361895 1.965301
43
6-B-Me-Cl-4 Eopt
-1385.398686
C 4.921336 -0.461278 -0.771462
C 4.511602 -1.675007 -0.176550
C 3.260216 -1.851328 0.422735
C 2.430646 -0.732669 0.394767
C 2.788539 0.495666 -0.183276
C 4.051536 0.630816 -0.773771
H 5.200957 -2.516333 -0.182721
H 4.352255 1.570453 -1.227839
C 6.293758 -0.361668 -1.396093
H 7.081776 -0.555318 -0.659238
H 6.417826 -1.091912 -2.204013
H 6.466039 0.633250 -1.815735
C 2.822197 -3.144962 1.054727
H 1.935971 -3.550258 0.553264
H 3.617159 -3.892935 1.000728
H 2.555302 -3.000559 2.107440
C 1.641561 1.367171 0.008412
C 0.686944 0.591853 0.687941
C 1.335683 2.693102 -0.320966
C -0.565974 1.065736 1.064283
C 0.089455 3.213791 0.036772
H 2.056558 3.311881 -0.847278
C -0.833486 2.394369 0.720315
H -1.800486 2.811700 0.992105
C -0.281329 4.639855 -0.298142
H 0.532584 5.147315 -0.823116
H -1.169563 4.679614 -0.938971
H -0.506860 5.215825 0.606663
C -1.580922 0.182553 1.758139
H -1.059540 -0.521887 2.414067
H -2.222844 0.803494 2.390053
C -2.437474 -0.587397 0.768583
C -3.733722 -0.151964 0.473669
C -1.932060 -1.717897 0.111773
C -4.499702 -0.842699 -0.464628
H -4.147506 0.719351 0.970274
C -2.711483 -2.397596 -0.823808
H -0.923856 -2.053355 0.331157
C -4.006556 -1.966560 -1.123211
H -2.312363 -3.272779 -1.327557
H -4.620063 -2.489342 -1.847859
O 1.154748 -0.687952 0.928716
Cl -6.129173 -0.279288 -0.820142
43
6-B-Me-Cl-5 Eopt
-1385.399415
C 3.916477 -1.894510 -0.508601
C 2.936067 -2.763437 0.020642
C 1.702224 -2.321208 0.508111
C 1.503568 -0.944414 0.438809
C 2.439210 -0.037831 -0.084540
C 3.663856 -0.522406 -0.562442
H 3.146713 -3.830180 0.048313
H 4.406239 0.156160 -0.972351
C 5.223105 -2.462769 -1.012261
H 5.766529 -2.980735 -0.213537
H 5.058133 -3.188890 -1.816569
H 5.873319 -1.674536 -1.401725
C 0.648161 -3.242128 1.061016
H -0.287737 -3.156505 0.497884
H 0.980240 -4.282515 1.019962
H 0.416928 -2.996642 2.103778
C 1.812244 1.270023 0.014743
C 0.551967 1.040067 0.590753
C 2.176473 2.578658 -0.327771
C -0.380574 2.043985 0.840937
C 1.277674 3.620723 -0.089340
H 3.144798 2.783470 -0.775014
C 0.019833 3.335355 0.485968
H -0.671873 4.156216 0.661979
C 1.633511 5.047527 -0.437656
H 2.620388 5.106023 -0.904834
H 0.906974 5.481268 -1.134264
H 1.647977 5.683737 0.454937
C -1.743184 1.731403 1.419348
H -1.627645 1.169211 2.352419
H -2.246014 2.671464 1.667608
C -2.599513 0.928946 0.455119
C -2.732427 -0.454305 0.612574
C -3.223950 1.553433 -0.631655
C -3.471142 -1.186449 -0.313996
H -2.242011 -0.959667 1.435943
C -3.968618 0.808116 -1.545991
H -3.118047 2.626223 -0.765246
C -4.098142 -0.575146 -1.397563
H -4.451671 1.301810 -2.383663
H -4.669341 -1.165497 -2.104702
O 0.356679 -0.298663 0.861871
Cl -3.601661 -2.931348 -0.110771
41
7-A-Me-1 Eopt
-812.449082
C 5.509755 -0.092456 -0.658735
C 5.421467 -1.420939 -0.243127
C 4.212759 -1.932539 0.251584
C 3.104870 -1.082409 0.313284
C 3.173335 0.258187 -0.102276
C 4.395293 0.744701 -0.590297
H 6.293959 -2.066608 -0.301946
H 2.170192 -1.462041 0.716675
H 4.464232 1.769336 -0.941499
C 4.121425 -3.362621 0.728146
H 4.516449 -4.056749 -0.021572
H 4.702539 -3.510666 1.646117
H 3.086836 -3.646593 0.939886
C 1.979515 1.135532 -0.026199
C 0.706252 0.645227 -0.355052
C 2.095587 2.477681 0.371345

C	-0.434398	1.453244	-0.294678
H	0.607023	-0.382826	-0.693593
C	0.967994	3.295517	0.434015
H	3.067326	2.871563	0.651668
C	-0.290308	2.788241	0.104562
H	-1.165290	3.431435	0.152089
C	-1.795393	0.881398	-0.610934
H	-1.712774	0.123707	-1.398495
H	-2.454649	1.667034	-0.996829
H	6.448139	0.291002	-1.048760
H	1.069554	4.330738	0.746517
C	-2.460836	0.237642	0.630037
H	-1.800923	-0.548549	1.014414
H	-2.541948	0.994363	1.418582
C	-3.822150	-0.334396	0.316631
C	-3.956704	-1.647050	-0.156102
C	-4.976890	0.449588	0.443429
C	-5.209072	-2.163639	-0.494455
H	-3.071291	-2.270397	-0.255641
C	-6.232264	-0.061251	0.106846
H	-4.890488	1.468247	0.813788
C	-6.352368	-1.370972	-0.364489
H	-5.292763	-3.184766	-0.854971
H	-7.115669	0.561041	0.216452
H	-7.327652	-1.771391	-0.624080

41

7-A-Me-10 Eopt

-812.449076

C	3.910174	-2.343426	-0.708140
C	5.163236	-1.957641	-0.232544
C	5.358963	-0.672980	0.295657
C	4.272686	0.206057	0.330484
C	3.003029	-0.163525	-0.144886
C	2.834827	-1.454879	-0.666499
H	5.995983	-2.655272	-0.270744
H	4.407581	1.195226	0.759101
H	1.869762	-1.752305	-1.064283
C	6.709078	-0.260876	0.832291
H	6.969826	-0.835079	1.729141
H	7.499330	-0.435660	0.093921
H	6.724783	0.799464	1.098997
C	1.869344	0.792027	-0.095941
C	0.579945	0.356532	0.247068
C	2.058985	2.153198	-0.385759
C	-0.505900	1.236991	0.304184
H	0.425969	-0.688492	0.503072
C	0.986604	3.042796	-0.330868
H	3.043094	2.507794	-0.675261
C	-0.288892	2.589505	0.010698
H	-1.120351	3.288254	0.055260
C	-1.888692	0.727230	0.631840
H	-2.476860	1.515328	1.115327
H	-1.825804	-0.102351	1.345253
H	3.771937	-3.337232	-1.124067
H	1.144569	4.092434	-0.561055
C	-2.646220	0.242428	-0.628478
H	-2.709009	1.071487	-1.342448
H	-2.056843	-0.545405	-1.111396
C	-4.028586	-0.269118	-0.302825
C	-5.131415	0.595698	-0.297758
C	-4.231885	-1.610115	0.050717
C	-6.402940	0.135933	0.051581
H	-4.991784	1.637851	-0.574777
C	-5.500752	-2.075951	0.401100
H	-3.387684	-2.295464	0.046945
C	-6.591697	-1.202948	0.403360
H	-7.245658	0.821104	0.044996
H	-5.638321	-3.119795	0.667820
H	-7.579869	-1.563490	0.672502

41

7-A-Me-11 Eopt

-812.454213

C	-4.314931	0.951309	-0.267037
C	-4.335857	-0.443632	-0.262731
C	-3.140190	-1.172095	-0.177018
C	-1.934296	-0.470222	-0.098639
C	-1.894041	0.934891	-0.104248
C	-3.103454	1.640183	-0.188268
H	-5.284034	-0.971592	-0.326852
H	-1.006203	-1.025189	-0.005078
H	-3.093700	2.725205	-0.219369
C	-3.155927	-2.682193	-0.149974
H	-3.629590	-3.056823	0.765347
H	-3.720355	-3.088578	-0.996615
H	-2.141551	-3.087419	-0.192892
C	-0.593329	1.641308	-0.016591
C	0.534475	1.131041	-0.675604
C	-0.449161	2.814195	0.742282
C	1.788287	1.741956	-0.575604
H	0.433463	0.234202	-1.278295
C	0.791615	3.444222	0.838060
H	-1.303309	3.215817	1.278350
C	1.906866	2.909314	0.189145
H	2.873375	3.399319	0.276882
C	2.997184	1.101159	-1.212273
H	3.738288	1.866328	-1.469598
H	2.707451	0.604623	-2.145217
H	-5.245812	1.505882	-0.343186
H	0.892556	4.348761	1.430845
C	3.671409	0.056409	-0.280635
H	4.561890	-0.334468	-0.786911
H	4.012583	0.564457	0.628015
C	2.749259	-1.081398	0.087943
C	2.548010	-2.150975	-0.795341
C	2.020876	-1.060123	1.284741
C	1.644499	-3.172182	-0.493620
H	3.103519	-2.182735	-1.729677
C	1.118736	-2.079983	1.594365
H	2.151093	-0.228297	1.970899
C	0.926688	-3.140017	0.705180
H	1.504405	-3.993323	-1.190604
H	0.559596	-2.040933	2.524414
H	0.226023	-3.934239	0.944744

41

7-A-Me-12 Eopt

-812.453528

C	-2.597425	-2.097947	-0.152116
C	-3.927697	-1.680936	-0.207364
C	-4.245821	-0.315012	-0.193301
C	-3.201780	0.611935	-0.118116
C	-1.856926	0.210310	-0.060869
C	-1.564784	-1.162633	-0.078698
H	-4.725610	-2.417295	-0.261154
H	-3.436380	1.672900	-0.127382
H	-0.535341	-1.496239	-0.004803
C	-5.682459	0.141353	-0.287073
H	-6.073983	0.005409	-1.302529
H	-6.326486	-0.431297	0.388733
H	-5.781389	1.200502	-0.033011
C	-0.765161	1.210110	0.026105
C	0.440477	1.002910	-0.659542
C	-0.897806	2.369129	0.808134
C	1.507255	1.902363	-0.566321
H	0.551076	0.117861	-1.277902
C	0.151926	3.283119	0.898673
H	-1.813515	2.539407	1.365535
C	1.350624	3.050239	0.220727
H	2.168632	3.761560	0.303403
C	2.824380	1.590606	-1.233846
H	3.344955	2.519326	-1.493636
H	2.648382	1.045808	-2.168189
H	-2.362167	-3.158482	-0.154146
H	0.039740	4.174235	1.509508
C	3.757244	0.738811	-0.329038
H	4.704171	0.581674	-0.858796
H	3.984302	1.309623	0.578037
C	3.148566	-0.591565	0.044516
C	3.193005	-1.671638	-0.847687
C	2.459118	-0.753925	1.253338
C	2.563659	-2.880675	-0.544108
H	3.722769	-1.562617	-1.791138
C	1.830788	-1.961396	1.564697
H	2.399555	0.079911	1.946801
C	1.879039	-3.029210	0.665384
H	2.609939	-3.706029	-1.248599
H	1.295132	-2.063779	2.503712
H	1.388886	-3.968178	0.904574

41

7-A-Me-13 Eopt

-812.451793

C	-3.769864	0.179958	1.430648
C	-4.256834	-0.573245	0.362410
C	-3.543682	-0.639852	-0.843524
C	-2.338277	0.060223	-0.945131
C	-1.830207	0.822820	0.119718
C	-2.564890	0.873950	1.313964
H	-5.194325	-1.114226	0.462861
H	-1.787451	0.028792	-1.880923
H	-2.177316	1.432323	2.160122
C	-4.081447	-1.430928	-2.012103
H	-4.404187	-2.430025	-1.700202
H	-4.950357	-0.933176	-2.459236
H	-3.326443	-1.547981	-2.794502
C	-0.537545	1.538294	-0.007817
C	0.534839	0.951685	-0.695599
C	-0.345495	2.800426	0.577793
C	1.781388	1.579254	-0.791981
H	0.412092	-0.039852	-1.119260
C	0.887735	3.444169	0.478781
H	-1.170152	3.286147	1.089818
C	1.947860	2.836442	-0.197285
H	2.909851	3.338642	-0.262641
C	2.947986	0.863891	-1.429188
H	3.656219	1.593126	-1.838786
H	2.597177	0.247876	-2.264865
H	-4.324592	0.217079	2.363796
H	1.020812	4.425962	0.923884
C	3.703865	-0.049109	-0.423347
H	4.562613	-0.494902	-0.938857
H	4.098714	0.572584	0.387766
C	2.820103	-1.133770	0.143503
C	2.588230	-2.312364	-0.578696
C	2.138159	-0.947819	1.353028
C	1.691161	-3.275384	-0.112075
H	3.110862	-2.473157	-1.518857
C	1.239270	-1.906432	1.824319
H	2.295170	-0.032035	1.915637
C	1.009484	-3.072532	1.090994

H 1.524201 -4.182127 -0.686335
H 0.710156 -1.736998 2.757297
H 0.305930 -3.816113 1.452819
41
7-A-Me-14 Eopt
-812.451712
C 3.182546 -0.773658 1.908026
C 4.111179 -0.946764 0.881892
C 3.905911 -0.349593 -0.370648
C 2.754377 0.419129 -0.561777
C 1.808056 0.606496 0.460123
C 2.037563 -0.003047 1.702270
H 5.000506 -1.548633 1.050972
H 2.572565 0.863502 -1.536394
H 1.330639 0.144934 2.512293
C 4.902341 -0.547804 -1.488140
H 5.901302 -0.207181 -1.193035
H 4.987864 -1.606284 -1.759920
H 4.609966 0.005718 -2.384751
C 0.589104 1.416052 0.219709
C -0.646293 1.012749 0.747386
C 0.634429 2.584507 -0.558177
C -1.821774 1.730909 0.504522
H -0.702096 0.089950 1.316019
C -0.527507 3.317534 -0.798945
H 1.584536 2.930508 -0.952857
C -1.750130 2.892482 -0.275071
H -2.654066 3.462935 -0.473636
C -3.152051 1.200967 0.981888
H -3.844526 2.031493 1.160660
H -3.024028 0.674499 1.934348
H 3.354481 -1.234417 2.876493
H -0.478388 4.227342 -1.390219
C -3.801239 0.226785 -0.040649
H -4.778539 -0.082515 0.348058
H -3.980374 0.767611 -0.976482
C -2.941514 -0.985768 -0.304383
C -2.968374 -2.081197 0.569419
C -2.032674 -1.005215 -1.370390
C -2.104647 -3.163689 0.390277
H -3.668840 -2.082595 1.401421
C -1.166309 -2.084332 -1.554474
H -1.988658 -0.155863 -2.046070
C -1.196719 -3.166457 -0.672120
H -2.139911 -4.003212 1.078558
H -0.460642 -2.074531 -2.379808
H -0.519449 -4.003779 -0.810072
41
7-A-Me-2 Eopt
-812.451027
C -4.357689 -1.796139 0.820748
C -5.187020 -0.677494 0.898621
C -4.742029 0.570924 0.439063
C -3.452315 0.665029 -0.091465
C -2.601041 -0.449863 -0.176640
C -3.072550 -1.686918 0.287577
H -6.185714 -0.770759 1.317574
H -3.106245 1.622167 -0.471686
H -2.423261 -2.556089 0.255127
C -5.648447 1.777518 0.495559
H -6.479940 1.679733 -0.212541
H -6.084277 1.900153 1.493016
H -5.106811 2.694681 0.247981
C -1.235372 -0.318641 -0.741000
C -0.448625 0.809489 -0.462833
C -0.697166 -1.319668 -1.566960
C 0.842195 0.955614 -0.982266
H -0.842920 1.578892 0.196274
C 0.586913 -1.185766 -2.092952
H -1.298089 -2.188312 -1.816945
C 1.354534 -0.056687 -1.802594
H 2.361381 0.033991 -2.199178
C 1.692092 2.138667 -0.585133
H 1.074740 3.043290 -0.533823
H 2.460949 2.314427 -1.345598
H -4.708764 -2.756413 1.187338
H 0.990581 -1.963020 -2.735120
C 2.384100 1.937429 0.789673
H 2.941193 2.849198 1.034884
H 1.612073 1.814091 1.557254
C 3.312916 0.747438 0.802670
C 4.631190 0.866982 0.342345
C 2.859955 -0.514260 1.210187
C 5.476477 -0.243272 0.288348
H 4.998581 1.839964 0.024303
C 3.700668 -1.627671 1.159587
H 1.836164 -0.624952 1.555734
C 5.012277 -1.496465 0.697001
H 6.496343 -0.129623 -0.067689
H 3.329896 -2.597587 1.477873
H 5.667744 -2.361306 0.658017
41
7-A-Me-3 Eopt
-812.451793
C 3.774244 0.190742 1.424969
C 4.260744 -0.563324 0.357452
C 3.543499 -0.636720 -0.845962
C 2.337316 0.061602 -0.946984
C 1.829675 0.825594 0.117456
C 2.566564 0.880741 1.309860
H 5.201800 -1.098562 0.455698
H 1.786017 0.029191 -1.882404
H 2.180738 1.441831 2.155017
C 4.068658 -1.451087 -2.004252
H 5.078080 -1.133218 -2.288363
H 4.123869 -2.514958 -1.745466
H 3.425146 -1.353687 -2.882913
C 0.536555 1.540136 -0.010325
C -0.535850 0.951533 -0.696409
C 0.343869 2.803117 0.573259
C -1.783014 1.577849 -0.792879
H -0.412569 -0.040558 -1.118605
C -0.890014 3.445593 0.474175
H 1.168500 3.290464 1.083764
C -1.950141 2.835840 -0.200066
H -2.912618 3.337097 -0.265498
C -2.949426 0.860432 -1.428114
H -2.598748 0.243821 -2.263408
H -3.658862 1.588394 -1.837887
H 4.332802 0.234383 2.355551
H -1.023588 4.428020 0.917729
C -3.703359 -0.052209 -0.420485
H -4.098000 0.569997 0.390337
H -4.562202 -0.499404 -0.934624
C -2.818024 -1.135412 0.146700
C -2.135182 -0.947537 1.355430
C -2.585727 -2.314581 -0.574414
C -1.235098 -1.904866 1.827028
H -2.292507 -0.031266 1.917156
C -1.687445 -3.276332 -0.107494
H -3.109004 -2.476862 -1.513959
C -1.004956 -3.071591 1.094791
H -0.705369 -1.733979 2.759392
H -1.520233 -4.183567 -0.680906
H -0.300591 -3.814252 1.456948
41
7-A-Me-4 Eopt
-812.451004
C 5.026653 1.019583 -0.353280
C 5.318929 -0.342435 -0.420146
C 4.298577 -1.295910 -0.287422
C 2.990055 -0.847833 -0.086243
C 2.675501 0.520049 -0.014127
C 3.715016 1.451858 -0.151381
H 6.343497 -0.670262 -0.576492
H 2.189089 -1.577702 -0.007232
H 3.499537 2.512750 -0.073597
C 4.609759 -2.770968 -0.377253
H 5.001182 -3.033471 -1.366935
H 5.367801 -3.061354 0.358829
H 3.717628 -3.377627 -0.198815
C 1.276257 0.963958 0.199891
C 0.432907 0.277857 1.087076
C 0.762731 2.082330 -0.477872
C -0.890875 0.675589 1.304031
H 0.825789 -0.573173 1.637625
C -0.553018 2.492173 -0.267570
H 1.389780 2.613836 -1.186724
C -1.377749 1.792918 0.615293
H -2.407936 2.104217 0.760432
C -1.788340 -0.131654 2.210805
H -2.606447 0.496949 2.579200
H -1.224515 -0.475237 3.086084
H 5.825376 1.749456 -0.448634
H -0.943039 3.350983 -0.805908
C -2.391740 -1.370913 1.497287
H -1.573017 -2.022643 1.172234
H -2.985105 -1.936542 2.225099
C -3.249840 -0.997926 0.312552
C -2.702258 -0.911710 -0.974263
C -4.599819 -0.664343 0.487161
C -3.481247 -0.503312 -2.058448
H -1.653627 -1.152679 -1.122898
C -5.383863 -0.254284 -0.593198
H -5.040404 -0.727924 1.479447
C -4.825338 -0.171746 -1.871556
H -3.037086 -0.440466 -3.047518
H -6.429383 -0.003895 -0.438292
H -5.432892 0.145097 -2.713927
41
7-A-Me-5 Eopt
-812.450989
C -3.999705 -1.764071 1.124055
C -5.128325 -0.978681 0.889915
C -5.013080 0.251532 0.226076
C -3.745240 0.668926 -0.189334
C -2.596135 -0.106980 0.038650
C -2.740578 -1.334209 0.703140
H -6.105029 -1.319620 1.223689
H -3.648244 1.609369 -0.724616
H -1.863516 -1.937847 0.914077
C -6.236711 1.091539 -0.052810
H -6.881521 0.613753 -0.799992
H -6.837177 1.230821 0.852703
H -5.963182 2.079856 -0.432530
C -1.263132 0.364128 -0.410706
C -0.332537 -0.532832 -0.957287
C -0.900976 1.717250 -0.303001

C 0.931676 -0.114489 -1.386582
H -0.610081 -1.577757 -1.070464
C 0.353699 2.148374 -0.731309
H -1.595629 2.424579 0.138921
C 1.267314 1.239536 -1.267646
H 2.250770 1.576785 -1.581189
C 1.931952 -1.121420 -1.901364
H 2.659954 -0.623145 -2.550937
H 1.421934 -1.879040 -2.507883
H -4.098522 -2.710639 1.647550
H 0.627285 3.194714 -0.632211
C 2.693377 -1.839825 -0.755428
H 1.968325 -2.378493 -0.135132
H 3.359966 -2.589470 -1.197529
C 3.489195 -0.884432 0.100640
C 2.932901 -0.317814 1.254993
C 4.779752 -0.492305 -0.280033
C 3.645554 0.615749 2.009766
H 1.927850 -0.600647 1.554463
C 5.497194 0.441733 0.470212
H 5.226582 -0.924393 -1.172475
C 4.930534 1.000098 1.619193
H 3.195412 1.045244 2.899935
H 6.497839 0.729780 0.161094
H 5.486603 1.725312 2.205681
41
7-A-Me-6 Eopt
-812.451022
C 4.615025 -1.404547 -0.693795
C 5.257752 -0.171149 -0.798107
C 4.604846 1.006318 -0.404501
C 3.302278 0.911659 0.093804
C 2.638330 -0.321690 0.207883
C 3.314156 -1.483343 -0.194587
H 6.271943 -0.119162 -1.185829
H 2.779543 1.820038 0.380268
H 2.831155 -2.449622 -0.089559
C 5.289750 2.345630 -0.538173
H 6.277046 2.335163 -0.063515
H 5.438809 2.609887 -1.591791
H 4.700847 3.142063 -0.074854
C 1.255303 -0.389212 0.740146
C 0.313210 -1.264611 0.178575
C 0.855316 0.418770 1.817615
C -0.997122 -1.347732 0.661584
H 0.602427 -1.876148 -0.672420
C -0.446865 0.344667 2.310000
H 1.574879 1.083499 2.285042
C -1.369779 -0.531288 1.736310
H -2.388097 -0.571684 2.111289
C -2.006705 -2.234104 -0.027379
H -2.809993 -2.493061 0.671208
H -1.531964 -3.172576 -0.337089
H 5.133184 -2.311412 -0.991955
H -0.743279 0.969609 3.147294
C -2.629816 -1.561810 -1.279959
H -1.831141 -1.350333 -1.999539
H -3.311824 -2.276764 -1.754874
C -3.368743 -0.288267 -0.947050
C -2.724219 0.954264 -1.006859
C -4.698082 -0.330215 -0.505018
C -3.388788 2.124517 -0.635243
H -1.689415 1.000571 -1.333806
C -5.367621 0.836633 -0.130761
H -5.213165 -1.286772 -0.454827
C -4.713106 2.069805 -0.194184
H -2.870839 3.077739 -0.686709
H -6.399136 0.784043 0.205444
H -5.231704 2.979144 0.094406
41
7-A-Me-7 Eopt
-812.451028
C -4.359803 -1.795013 -0.821826
C -5.189010 -0.676004 -0.898281
C -4.743522 0.571700 -0.437783
C -3.453338 0.664964 0.092227
C -2.602347 -0.450065 0.175949
C -3.074514 -1.686695 -0.289219
H -6.187893 -0.768772 -1.316874
H -3.107057 1.621720 0.473209
H -2.425355 -2.556006 -0.258062
C -5.649425 1.778806 -0.491845
H -6.095042 1.896537 -1.485524
H -6.473896 1.685692 0.225086
H -5.104523 2.696737 -0.254489
C -1.236332 -0.319906 0.739620
C -0.449474 0.808437 0.462365
C -0.697752 -1.322067 1.563895
C 0.841642 0.953727 0.981166
H -0.844021 1.578679 -0.195585
C 0.586788 -1.189115 2.089098
H -1.298602 -2.190918 1.813331
C 1.354440 -0.059837 1.799768
H 2.361589 0.030123 2.195720
C 1.691416 2.137332 0.585474
H 2.459691 2.312676 1.346631
H 1.073881 3.041843 0.534463
H -4.711375 -2.754777 -1.189282
H 0.990685 -1.967368 2.729920
C 2.384552 1.937446 -0.788891
H 1.613158 1.813925 -1.557090
H 2.940928 2.849886 -1.033286
C 3.314311 0.748280 -0.801740
C 2.863067 -0.513460 -1.210826
C 4.631939 0.868608 -0.339550
C 3.704695 -1.626221 -1.159936
H 1.839864 -0.624822 -1.557962
C 5.478092 -0.240909 -0.285264
H 4.998076 1.841684 -0.020326
C 5.015517 -1.494248 -0.695465
H 3.335155 -2.596195 -1.479500
H 6.497396 -0.126659 0.072214
H 5.671693 -2.358550 -0.656217
41
7-A-Me-8 Eopt
-812.449135
C -5.214121 -0.158039 1.338876
C -5.397797 -1.343247 0.626825
C -4.461368 -1.746774 -0.336335
C -3.343826 -0.937239 -0.559699
C -3.140356 0.259150 0.148589
C -4.094101 0.640650 1.104083
H -6.272001 -1.960467 0.817627
H -2.624374 -1.228552 -1.319961
H -3.943614 1.547358 1.681366
C -4.672081 -3.014905 -1.128841
H -4.893717 -3.862004 -0.470660
H -5.516102 -2.911847 -1.821180
H -3.786789 -3.267615 -1.718930
C -1.943011 1.096597 -0.107839
C -0.688997 0.507124 -0.331463
C -2.036038 2.497724 -0.134681
C 0.454160 1.274686 -0.578412
H -0.598643 -0.575249 -0.290595
C -0.904983 3.275891 -0.379604
H -3.001080 2.973564 0.007797
C 0.333239 2.670344 -0.600567
H 1.210585 3.281868 -0.795321
C 1.799880 0.611675 -0.748003
H 2.433549 1.209299 -1.413135
H 1.680464 -0.368906 -1.222649
H -5.940820 0.140519 2.088877
H -0.991943 4.358049 -0.411718
C 2.533239 0.423919 0.602662
H 2.656185 1.404924 1.075533
H 1.896568 -0.169608 1.268554
C 3.876194 -0.244717 0.435299
C 5.030965 0.513470 0.198589
C 3.990248 -1.641399 0.462742
C 6.266354 -0.104524 -0.006207
H 4.960688 1.598393 0.179493
C 5.222724 -2.265383 0.258641
H 3.104919 -2.244024 0.650858
C 6.366097 -1.497863 0.022422
H 7.150427 0.501005 -0.183508
H 5.291073 -3.348952 0.288512
H 7.326095 -1.980605 -0.133413
41
7-A-Me-9 Eopt
-812.449125
C -4.201127 -2.026231 -1.179495
C -5.229740 -1.835165 -0.257240
C -5.178091 -0.771342 0.655668
C -4.076563 0.088214 0.615810
C -3.030934 -0.087171 -0.306530
C -3.107276 -1.159917 -1.207216
H -6.078905 -2.513670 -0.243847
H -4.015018 0.901167 1.333941
H -2.324444 -1.298122 -1.946204
C -6.280697 -0.574808 1.668714
H -7.260700 -0.519271 1.181939
H -6.317997 -1.408746 2.379367
H -6.135931 0.345483 2.241248
C -1.874856 0.842273 -0.323817
C -0.572511 0.362064 -0.532182
C -2.054998 2.222322 -0.135328
C 0.534387 1.217063 -0.557775
H -0.416779 -0.706363 -0.656928
C -0.960935 3.086827 -0.158161
H -3.056339 2.618875 -0.000868
C 0.326441 2.589592 -0.368085
H 1.174880 3.268800 -0.389363
C 1.928560 0.660541 -0.718659
H 2.569635 1.390636 -1.225671
H 1.907186 -0.233979 -1.351449
H -4.255809 -2.847364 -1.888368
H -1.114291 4.153848 -0.025426
C 2.571611 0.292323 0.640783
H 2.596323 1.187758 1.272243
H 1.927449 -0.434239 1.148875
C 3.963407 -0.269716 0.480607
C 5.080452 0.576486 0.455210
C 4.165473 -1.645229 0.303042
C 6.364563 0.064907 0.257011
H 4.941605 1.645616 0.596856
C 5.447109 -2.163014 0.104480
H 3.310037 -2.316090 0.325617
C 6.552092 -1.308311 0.079953

H 7.217817 0.736849 0.245142
H 5.583169 -3.232591 -0.026561
H 7.549904 -1.708838 -0.071282
40
7-B-Me-1 Eopt
-886.472193
C -4.988772 -1.360927 0.363786
C -4.089914 -2.426234 0.176970
C -2.723456 -2.220474 -0.052595
C -2.327821 -0.885324 -0.081569
C -3.195964 0.206438 0.101205
C -4.555821 -0.036208 0.328640
H -4.462074 -3.446414 0.210690
H -5.252966 0.782852 0.473475
C -1.739146 -3.340506 -0.254825
H -0.955586 -3.321200 0.510941
H -2.240040 -4.310719 -0.208491
H -1.239130 -3.256645 -1.226111
C -2.361615 1.391730 -0.006642
C -1.058999 0.917622 -0.246672
C -2.578734 2.772488 0.072196
C 0.056976 1.733771 -0.417943
C -1.484230 3.620621 -0.093069
H -3.570976 3.172041 0.255816
C -0.196781 3.108808 -0.332240
H 0.633882 3.797629 -0.460026
C 1.430188 1.157314 -0.639046
H 1.386545 0.374899 -1.405241
H 2.100991 1.936662 -1.015605
O -1.028380 -0.464173 -0.293581
C 2.030100 0.547730 0.651905
H 1.337106 -0.207155 1.037395
H 2.105671 1.333484 1.411708
C 3.384035 -0.072439 0.406765
C 4.561060 0.662158 0.601544
C 3.488753 -1.387003 -0.069512
C 5.810899 0.101019 0.328905
H 4.496860 1.681286 0.975105
C 4.734982 -1.953015 -0.344139
H 2.583813 -1.970086 -0.222197
C 5.901728 -1.209493 -0.146044
H 6.712137 0.685190 0.490846
H 4.796039 -2.974457 -0.708410
H 6.872197 -1.648875 -0.356067
H -1.624556 4.695622 -0.039071
H -6.037658 -1.579190 0.538174
40
7-B-Me-2 Eopt
-886.473973
C -4.664707 0.724644 1.149169
C -4.633848 -0.661125 0.912125
C -3.544410 -1.290594 0.296104
C -2.500575 -0.440519 -0.061512
C -2.494461 0.948966 0.159081
C -3.600172 1.544608 0.777437
H -5.481164 -1.270202 1.214623
H -3.628187 2.613729 0.962068
C -3.484505 -2.770579 0.031048
H -2.633961 -3.230719 0.546340
H -4.398931 -3.263120 0.371101
H -3.358963 -2.976034 -1.037775
C -1.223059 1.422423 -0.362568
C -0.567587 0.280763 -0.859020
C -0.584500 2.665096 -0.450301
C 0.695946 0.291444 -1.443987
C 0.682687 2.713123 -1.030101
H -1.061008 3.565202 -0.075119
C 1.306346 1.549795 -1.512054
H 2.302115 1.618061 -1.939956
C 1.375186 -0.976203 -1.890295
H 0.685809 -1.578333 -2.493350
H 2.229669 -0.720148 -2.525340
O -1.335364 -0.856326 -0.678518
C 1.866872 -1.838520 -0.697376
H 2.335644 -2.744772 -1.097684
H 0.998411 -2.154929 -0.111285
C 2.842566 -1.095717 0.182642
C 2.396746 -0.370666 1.295781
C 4.207125 -1.059261 -0.134489
C 3.288742 0.372565 2.071009
H 1.340476 -0.381645 1.549229
C 5.104372 -0.318000 0.637311
H 4.569611 -1.618968 -0.993682
C 4.646493 0.402519 1.743648
H 2.922678 0.929518 2.928509
H 6.159102 -0.305971 0.377972
H 5.341932 0.978871 2.346385
H 1.203513 3.662363 -1.106070
H -5.535370 1.159118 1.630260
40
7-B-Me-3 Eopt
-886.476271
C 4.029366 0.566647 0.486198
C 3.770860 -0.601016 -0.254681
C 2.516330 -0.868607 -0.816609
C 1.549556 0.106926 -0.584472
C 1.768784 1.285378 0.152067
C 3.036226 1.521663 0.698168
H 4.567890 -1.325485 -0.397894
H 3.239147 2.420279 1.272081
C 2.206021 -2.110290 -1.606119
H 1.900380 -1.862226 -2.628899
H 3.078897 -2.766267 -1.657760
H 1.378073 -2.661637 -1.149164
C 0.495741 1.986092 0.152591
C -0.387316 1.166430 -0.577439
C 0.017502 3.178398 0.706648
C -1.737597 1.445976 -0.768380
C -1.325711 3.502892 0.512346
H 0.671753 3.831620 1.275282
C -2.181354 2.650499 -0.205540
H -3.226649 2.924141 -0.323780
C -2.672223 0.465919 -1.428690
H -3.474161 1.011126 -1.938730
H -2.133264 -0.112998 -2.185169
O 0.249939 0.034699 -1.041542
C -3.312816 -0.514373 -0.405366
H -4.025441 -1.152348 -0.940394
H -3.884290 0.069744 0.324797
C -2.289775 -1.368369 0.304392
C -1.645764 -0.905560 1.460452
C -1.903524 -2.606250 -0.224605
C -0.621950 -1.645467 2.054055
H -1.933657 0.052348 1.883784
C -0.889689 -3.357192 0.373218
H -2.396029 -2.980743 -1.118769
C -0.238027 -2.873709 1.510468
H -0.121957 -1.261279 2.938120
H -0.607284 -4.317137 -0.049781
H 0.559383 -3.449298 1.970721
H -1.721474 4.424882 0.926700
H 5.021326 0.721760 0.898971
12
C6H6 Eopt -
232.287286
C -1.342699 -0.388464 0.000003
C -0.334817 -1.357035 0.000069
C 1.007752 -0.968574 -0.000059
C 1.342667 0.388576 0.000007
C 0.334929 1.357005 0.000062
C -1.007832 0.968493 -0.000058
H -2.385679 -0.690347 -0.000062
H -0.595094 -2.411179 0.000080
H 1.790716 -1.720834 -0.000135
H 2.385717 0.690193 0.000015
H 0.594957 2.411204 0.000010
H -1.790617 1.720950 -0.000048
24
E Eopt -
576.702089
O 0.050470 -1.192775 0.940672
C 1.217515 -0.512176 0.398677
C 1.227560 0.853734 0.279940
C 2.409737 1.357627 -0.281421
C 3.444243 0.489035 -0.670029
C 3.348610 -0.902324 -0.535193
C 2.176885 -1.431690 0.016376
C -0.017939 1.591126 0.711703
C -1.233398 0.762974 0.331834
C -1.204944 -0.629368 0.377420
C -2.192939 -1.470127 -0.091328
C -3.352893 -0.866371 -0.588300
C -3.464808 0.524462 -0.613863
C -2.409687 1.326345 -0.174945
H 2.522206 2.427577 -0.421755
H 4.160515 -1.545328 -0.854777
H -0.075380 2.575432 0.243288
H 0.008139 1.753413 1.798631
H -2.075209 -2.544934 -0.092963
H -4.156120 -1.491949 -0.962263
H -4.366176 0.987201 -0.999711
H -2.484850 2.406566 -0.239588
H 4.345565 0.913636 -1.098307
H 2.029900 -2.498901 0.136834
24
F Eopt -
576.706658
O 1.256089 0.654889 0.000000
C 0.000000 1.378139 0.000000
C 0.248684 2.737723 0.000000
C -1.246765 0.794078 0.000000
C -2.321763 1.697404 0.000000
C -2.120745 3.081194 0.000000
C -0.833622 3.620298 0.000000
C -1.415315 -0.690201 0.000000
C -0.097982 -1.420174 0.000000
C 1.169414 -0.831716 0.000000
C 2.336094 -1.568011 0.000000
C 2.267767 -2.960358 0.000000
C 1.028362 -3.595779 0.000000
C -0.126641 -2.823309 0.000000
H -3.331362 1.299118 0.000000
H -2.979680 3.743050 0.000000
H -0.676421 4.692981 0.000000
H -1.997361 -0.997317 0.877000
H -1.997361 -0.997317 -0.877000
H 3.292275 -1.092604 0.000000
H 3.189262 -3.531863 0.000000

H 0.961141 -4.677882 0.000000
H -1.100012 -3.305384 0.000000
H 1.265884 3.112370 0.000000
27
G Eopt -616.021128
O 0.049858 -0.791654 1.203491
C -1.226721 -0.600546 0.481591
C -1.637067 0.721049 0.389366
C -1.879179 -1.694442 -0.054559
C -2.851360 0.928407 -0.279938
C -3.085773 -1.446192 -0.714186
H -1.477598 -2.696177 0.036640
C -3.571322 -0.140894 -0.815576
H -3.224803 1.942362 -0.381084
H -3.636150 -2.274603 -1.146155
H -4.511633 0.047052 -1.324029
C 1.251166 -0.382910 0.449604
C 1.427690 0.864001 -0.114633
C 2.156413 -1.442491 0.467342
C 2.665570 0.988680 -0.780256
C 3.371400 -1.271789 -0.194840
C 3.603415 -0.046436 -0.827239
H 2.895436 1.936399 -1.257934
H 4.116474 -2.059072 -0.207226
H 4.539536 0.114718 -1.350628
C -0.756408 1.842690 0.875268
H -1.330404 2.771479 0.891961
H -0.422328 1.655720 1.902234
C 0.463796 2.031465 -0.047120
H 0.112188 2.250674 -1.062756
H 1.026883 2.910843 0.282351
H 1.923816 -2.369720 0.979758
27
H Eopt -616.008193
O 0.000294 -1.374999 0.344731
C -1.313946 -0.669976 0.105813
C -1.422432 0.631488 -0.404650
C -2.412321 -1.401016 0.518821
C -2.740052 1.048769 -0.672933
C -3.699536 -0.939493 0.257120
H -2.275450 -2.306385 1.070674
C -3.863587 0.279793 -0.394345
H -2.868099 2.047167 -1.081044
H -4.552785 -1.526898 0.578441
H -4.853190 0.657687 -0.626398
C 1.284274 -0.661418 0.182000
C 1.566446 0.636379 0.579628
C 2.183089 -1.497904 -0.462814
C 2.907894 1.017063 0.406176
C 3.504425 -1.081723 -0.642935
C 3.865091 0.176829 -0.169852
H 3.188274 2.023921 0.700316
H 4.225471 -1.728943 -1.128273
H 4.886350 0.525271 -0.278036
C -0.352039 1.697822 -0.459838
H -0.858727 2.660865 -0.551381
H 0.300110 1.599639 -1.333560
C 0.506300 1.666717 0.827256
H -0.121448 1.447515 1.696502
H 0.978679 2.636117 0.998715
H 1.866822 -2.455955 -0.820482
32
N-phenyl-carbazole Eopt -748.642493
N 0.238928 0.000002 -0.000009
C -0.576724 1.135081 -0.047636
C -0.217511 2.482324 -0.137156
C -1.245889 3.423185 -0.191842
C -2.598055 3.035157 -0.162737
C -2.948438 1.688419 -0.086709
C -1.935836 0.723843 -0.030716
C -1.935830 -0.723855 0.030710
C -0.576717 -1.135082 0.047626
C -0.217485 -2.482320 0.137154
C -1.245852 -3.423193 0.191849
C -2.598023 -3.035176 0.162744
C -2.948421 -1.688443 0.086712
C 2.356009 0.716898 0.977859
C 1.656869 0.000006 -0.000006
C 2.356021 -0.716884 -0.977861
C 3.751419 -0.722486 -0.968902
C 4.452981 0.000015 0.000004
C 3.751405 0.722510 0.968909
H -0.994450 4.477122 -0.260945
H -3.992412 1.389630 -0.074693
H -0.994405 -4.477127 0.260958
H -3.992399 -1.389668 0.074696
H 1.802637 -1.255191 -1.739904
H 4.290062 -1.281427 -1.727947
H 5.538410 0.000020 0.000011
H 4.290041 1.281452 1.727957
H -3.373447 3.793383 -0.205174
H -3.373407 -3.793410 0.205188
H 0.822382 -2.787307 0.169001
H 0.822351 2.787329 -0.169004
H 1.802617 1.255196 1.739903
32
O-phenyl-benzoduranium-ts
Eopt -768.798989
O -0.233440 0.000003 -0.000349
C 0.618701 -1.174216 -0.000210
C 0.183194 -2.477896 -0.000290
C 1.210575 -3.433374 -0.000157
C 2.560194 -3.051727 0.000040
C 2.940357 -1.707288 0.000118
C 1.939652 -0.729939 -0.000006
C 1.939652 0.729936 0.000040
C 0.618700 1.174213 -0.000133
C 0.183200 2.477897 -0.000158
C 1.210582 3.433373 0.000028
C 2.560201 3.051723 0.000205
C 2.940359 1.707283 0.000212
C -2.315812 -0.000287 1.229955
C -1.679817 0.000004 -0.000111
C -2.316198 0.000292 -1.229977
C -3.713724 0.000292 -1.216124
C -4.403990 0.000001 0.000316
C -3.713341 -0.000289 1.216540
H 0.947980 -4.485340 -0.000209
H 3.987532 -1.426073 0.000276
H 0.947990 4.485340 0.000031
H 3.987534 1.426065 0.000347
H -1.748773 0.000504 -2.153925
H -4.257326 0.000514 -2.154422
H -5.488704 -0.000002 0.000487
H -4.256650 -0.000512 2.155009
H 3.325725 -3.819742 0.000137
H 3.325734 3.819735 0.000342
H -0.865164 2.750241 -0.000298
H -0.865172 -2.750236 -0.000443
H -1.748101 -0.000499 2.153727
32
O-phenyl-benzoduranium
Eopt -768.800035
O 0.229900 0.000073 -0.525193
C -0.607521 1.171363 -0.273769
C -0.182333 2.475248 -0.364853
C -1.177867 3.431895 -0.115941
C -2.491830 3.050705 0.192397
C -2.864735 1.706207 0.269474
C -1.891769 0.729972 0.032736
C -1.891633 -0.730248 0.032751
C -0.607309 -1.171417 -0.273756
C -0.181848 -2.475215 -0.364758
C -1.177200 -3.432040 -0.115789
C -2.491239 -3.051090 0.192517
C -2.864408 -1.706664 0.269535
C 1.969405 0.000407 1.188048
C 1.642190 0.000162 -0.157493
C 2.547394 -0.000051 -1.202085
C 3.904327 -0.000015 -0.860010
C 4.288712 0.000233 0.483311
C 3.329819 0.000447 1.503032
H -0.919436 4.483662 -0.166727
H -3.884077 1.425010 0.509291
H -0.918561 -4.483758 -0.166513
H -3.883798 -1.425657 0.509367
H 2.211101 -0.000216 -2.232752
H 4.652676 -0.000175 -1.644759
H 5.342643 0.000253 0.739667
H 3.637041 0.000646 2.543029
H -3.234948 3.818881 0.375913
H -3.234210 -3.819403 0.376065
H 0.839037 -2.747380 -0.603604
H 0.838497 2.747591 -0.603737
H 1.201910 0.000564 1.954219
23
Ph2O Eopt -538.594283
C -2.616870 1.240678 -0.867188
C -1.363116 0.629032 -0.819730
C -1.195222 -0.519784 -0.041309
C -2.263929 -1.062420 0.672998
C -3.514519 -0.444874 0.610769
C -3.696164 0.710045 -0.154588
H -2.749920 2.133047 -1.471441
H -0.525459 1.034006 -1.376416
H -2.101098 -1.957814 1.263161
H -4.346281 -0.866639 1.166828
H -4.668280 1.190493 -0.197875
O 0.000054 -1.213124 -0.000355
C 1.195253 -0.519692 0.041004
C 2.264350 -1.062596 -0.672534
C 1.362725 0.629466 0.819011
C 3.514882 -0.444985 -0.609947
H 2.101844 -1.958249 -1.262395
C 2.616444 1.241167 0.866830
H 0.524797 1.034670 1.375116
C 3.696113 0.710273 0.155005
H 4.346931 -0.866963 -1.165414
H 2.749154 2.133799 1.470768
H 4.668191 1.190773 0.198568
34
Ph3O+ Eopt -770.000999
C -0.239618 -1.430839 -0.126708

C 0.326234 -2.267678 -1.072176
C -0.996954 -1.850364 0.952661
C 0.113807 -3.639693 -0.913487
H 0.913773 -1.869594 -1.891600
C -1.201396 -3.227221 1.084916
H -1.422119 -1.141885 1.653865
C -0.647275 -4.115059 0.158643
H 0.539224 -4.331002 -1.632644
H -1.792051 -3.599484 1.914793
H -0.810685 -5.181302 0.271408
C -1.119692 0.923626 -0.126978
C -1.103319 1.791828 0.950133
C -2.128776 0.849087 -1.070856
C -2.193966 2.657068 1.081717
H -0.276512 1.808211 1.650453
C -3.211034 1.718606 -0.912775
H -2.078395 0.139207 -1.888579
C -3.241220 2.618143 0.157243
H -2.220102 3.356618 1.910031
H -4.023470 1.693598 -1.630687
H -4.083224 3.292464 0.269522
C 1.359414 0.507878 -0.126923
C 1.802522 1.413914 -1.074210
C 2.100472 0.063870 0.953899
C 3.096997 1.915650 -0.915875
H 1.164599 1.721986 -1.894772
C 3.395346 0.574764 1.085768
H 1.698682 -0.656680 1.656639
C 3.888312 1.496172 0.157766
H 3.483904 2.627766 -1.636473
H 4.012274 0.250942 1.916843
H 4.893552 1.887471 0.270343
O 0.000178 0.000398 -0.299462
45
SM-6-2Me Eopt
-964.219667
O -0.242801 -0.356944 0.549883
C -0.632435 1.014749 0.295161
C -1.907819 1.422899 0.579565
C -2.142870 2.771778 0.289616
C -1.123592 3.601608 -0.232765
C 0.168343 3.094139 -0.462438
C 0.431932 1.746581 -0.188810
C 1.604226 0.866184 -0.163299
C 1.215840 -0.380794 0.343946
C 2.053954 -1.381919 0.788090
C 3.411678 -1.128144 0.515290
C 3.875397 0.055215 -0.083443
C 2.961806 1.076265 -0.394378
C -2.821480 0.373681 1.170013
C -2.558351 -0.960447 0.486884
C -1.278809 -1.316279 0.046156
C -0.977932 -2.353067 -0.833393
C -2.067871 -3.197950 -1.120113
C -3.344689 -2.964922 -0.620061
C -3.597731 -1.827039 0.145815
H -3.125119 3.195014 0.478345
H 0.948707 3.748174 -0.836121
H 4.135040 -1.881989 0.812998
H 3.311730 2.028036 -0.779931
H -3.872501 0.643940 1.051052
H -2.628364 0.299053 2.249340
H -1.894862 -4.037292 -1.785878
H -4.154885 -3.639764 -0.873649
H -4.607794 -1.589373 0.463449
C 1.611018 -2.593162 1.566610
H 0.558350 -2.835663 1.413722
H 2.210451 -3.467348 1.300854
H 1.754061 -2.410635 2.637396
C 0.329639 -2.587742 -1.553091
H 0.788428 -1.657072 -1.890320
H 1.061330 -3.118607 -0.940552
H 0.134166 -3.201375 -2.434343
C 5.345076 0.236779 -0.367280
H 5.586223 -0.115853 -1.376902
H 5.635027 1.288929 -0.308473
H 5.961246 -0.329202 0.335622
C -1.436519 5.044680 -0.545459
H -1.977980 5.519620 0.277962
H -0.526693 5.619671 -0.730746
H -2.067016 5.118746 -1.438587
42
SM-6-Cl-Me Eopt
-1384.480878
O -0.266090 -0.225254 0.592857
C -0.686274 1.131391 0.285198
C -1.968386 1.524253 0.558019
C -2.228561 2.858987 0.224807
C -1.223175 3.690589 -0.319533
C 0.081141 3.202042 -0.522059
C 0.369822 1.869400 -0.206249
C 1.564042 1.022793 -0.128941
C 1.209453 -0.217872 0.412565
C 2.063912 -1.175510 0.913556
C 3.419425 -0.878644 0.675468
C 3.858848 0.297036 0.044909
C 2.919645 1.271785 -0.330988
C -2.862163 0.486822 1.198141
C -2.567616 -0.877074 0.590769
C -1.274567 -1.211934 0.169466
C -0.997798 -2.308212 -0.645295
C -2.039366 -3.213380 -0.883013
C -3.314688 -2.970085 -0.383524
C -3.585272 -1.790451 0.310492
H -3.218706 3.269358 0.399539
H 0.852482 3.860619 -0.906354
H 4.159118 -1.594025 1.023784
H 3.246837 2.221700 -0.740236
H -3.918365 0.725678 1.059144
H -2.674863 0.473288 2.280949
H -1.835371 -4.083820 -1.495772
H -4.110937 -3.678389 -0.583745
H -4.598300 -1.559091 0.622508
C 1.636470 -2.409174 1.662456
H 0.595760 -2.353426 1.988172
H 1.750039 -3.303297 1.043000
H 2.260340 -2.536363 2.550783
C 5.329193 0.521922 -0.201447
H 5.615833 0.129867 -1.184253
H 5.577929 1.586059 -0.186288
H 5.942446 0.014753 0.547438
C -1.561283 5.114679 -0.687145
H -2.198403 5.579957 0.070143
H -0.661016 5.723394 -0.796539
H -2.103807 5.147816 -1.638856
Cl 0.505346 -2.556366 -1.483658
42
SM-6-Me Eopt
-924.900062
O -0.207814 -0.495283 0.591486
C -0.682228 0.864891 0.378714
C -2.002702 1.179676 0.568225
C -2.308332 2.516936 0.287613
C -1.321428 3.426561 -0.153556
C -1.700414 4.864561 -0.409483
C 0.005073 3.001299 -0.346182
C 0.340751 1.669328 -0.081339
C 1.551155 0.848494 -0.137151
C 1.239316 -0.446369 0.298084
C 2.143317 -1.443737 0.607182
C 3.472619 -1.106122 0.281987
C 3.854165 0.138787 -0.241630
C 2.882375 1.137315 -0.422460
C 5.296652 0.410686 -0.585286
C 1.808322 -2.749802 1.280886
C -2.922484 0.057181 0.986294
C -2.494078 -1.216166 0.276358
C -1.151639 -1.484527 0.012864
C -0.677341 -2.529799 -0.752842
C -1.623110 -3.434747 -1.245498
C -2.979162 -3.249378 -0.974543
C -3.407488 -2.143439 -0.237130
H -3.331467 2.861820 0.405081
H -2.663004 4.935755 -0.923649
H -1.791169 5.412830 0.535117
H -0.949766 5.372986 -1.018586
H 0.753224 3.701144 -0.702466
H 4.241476 -1.846940 0.482445
H 3.169031 2.129760 -0.753968
H 5.483525 0.197175 -1.644186
H 5.556862 1.457841 -0.410361
H 5.973156 -0.214440 0.002297
H 1.839437 -3.587660 0.576678
H 0.819691 -2.734816 1.742691
H 2.544221 -2.954874 2.062403
H -3.962049 0.287285 0.744305
H -2.867799 -0.079163 2.075262
H 0.373979 -2.639515 -0.977700
H -1.290623 -4.273054 -1.847384
H -3.709088 -3.953239 -1.358964
H -4.467152 -1.980417 -0.067541
39
SM-7-Me Eopt
-885.575527
O -0.006096 -0.107978 -0.470299
C -1.166928 -0.788134 0.144121
C -2.387042 -0.463379 -0.430315
C -0.972724 -1.637381 1.216647
C -3.502621 -1.103975 0.125735
C -2.109873 -2.256237 1.743398
H 0.013067 -1.818421 1.627292
C -3.366474 -1.996470 1.191223
H -4.485051 -0.891350 -0.284153
H -2.005962 -2.935256 2.582335
H -4.245778 -2.483161 1.599052
C 1.332111 -0.684690 -0.293936
C 2.169004 0.299753 0.227348
C 1.680221 -1.967125 -0.675204
C 3.504031 -0.036119 0.457624
C 3.033365 -2.265307 -0.418482
C 3.915554 -1.333624 0.140908
H 4.202351 0.692318 0.853667
H 3.398357 -3.251903 -0.685791
C 0.119888 1.323257 -0.130777
C -0.882125 2.255330 -0.308949
C 1.415406 1.546807 0.332633
C -0.497915 3.550470 0.098656
C 1.754320 2.843936 0.715638

C 0.773959 3.835271 0.603020
H -1.222384 4.352866 -0.002496
H 2.749197 3.077868 1.077055
H 1.009193 4.853250 0.893960
C -2.485730 0.600698 -1.492453
H -3.480236 0.570350 -1.943013
H -1.764774 0.413658 -2.296153
C -2.255554 2.003798 -0.896557
H -3.005810 2.188469 -0.118407
H -2.427529 2.753706 -1.676011
H 4.947906 -1.621040 0.308379
C 0.740674 -2.957109 -1.309146
H 0.179759 -3.515444 -0.552319
H 0.013313 -2.465039 -1.960044
H 1.303866 -3.675682 -1.907762
36
n6-a-Me-TS Eopt
-826.069407
C -3.946073 -1.432122 0.000024
C -2.974031 -2.434595 0.000032
C -1.585018 -2.211885 0.000003
C -1.154824 -0.851685 -0.000036
C -2.168603 0.164156 -0.000034
C -3.535549 -0.110949 -0.000008
N 0.113482 -0.163907 -0.000107
C -0.155606 1.217511 -0.000081
C -1.535890 1.456005 -0.000056
C 0.771464 2.257187 -0.000066
C 0.269611 3.559548 -0.000039
C -1.111696 3.816961 -0.000030
C -2.026344 2.766619 -0.000036
C 2.233509 1.933278 -0.000082
C 2.499921 0.442494 0.000041
C 1.488220 -0.553149 -0.000019
C 1.899679 -1.890134 -0.000022
C 3.242227 -2.257866 0.000087
C 4.236977 -1.283778 0.000195
C 3.842817 0.050395 0.000160
H -4.998783 -1.694995 0.000046
H -3.307871 -3.467404 0.000066
H -4.249236 0.706808 -0.000011
H 0.969460 4.391281 -0.000022
H -1.464260 4.843499 -0.000011
H -3.094390 2.959993 -0.000019
H 2.719118 2.385487 -0.875758
H 2.719182 2.385659 0.875468
H 1.169141 -2.667256 -0.000126
H 3.497655 -3.313185 0.000083
H 5.288325 -1.551840 0.000288
H 4.597469 0.833263 0.000215
C -0.772461 -3.492480 0.000033
H -1.457404 -4.342673 0.000096
H -0.146416 -3.597222 -0.890319
H -0.146349 -3.597139 0.890347
36
n6-a-Me Eopt
-826.083509
N -0.061274 -0.153759 0.336087
C 0.084312 1.233418 0.184922
C -0.946251 2.144979 0.365836
C -0.632402 3.483955 0.129927
C 0.671257 3.859767 -0.253335
C 1.690868 2.917377 -0.413756
C 1.392245 1.565765 -0.195717
C 2.125322 0.309179 -0.238160
C 1.216364 -0.729389 0.129752
C 1.665334 -2.034062 0.390505
C 3.015576 -2.291138 0.108383
C 3.898343 -1.302001 -0.344763
C 3.466345 0.015420 -0.488681
C -2.251800 1.565517 0.856968
C -2.458965 0.174737 0.279388
C -1.349103 -0.665754 0.027984
C -1.535132 -1.895435 -0.612052
C -2.821138 -2.339498 -0.920204
C -3.929396 -1.544527 -0.628117
C -3.733447 -0.286840 -0.052547
H -1.395483 4.247239 0.255450
H 2.688467 3.231462 -0.704709
H 3.395425 -3.292544 0.293775
H 4.159269 0.802169 -0.770382
H -3.099318 2.208901 0.602312
H -2.219676 1.505930 1.956556
H -0.676803 -2.485493 -0.899584
H -2.948586 -3.299813 -1.410317
H -4.931296 -1.884422 -0.869445
H -4.586729 0.359102 0.136905
H 0.887121 4.910238 -0.421761
H 4.935560 -1.558114 -0.536594
C 0.826967 -3.100467 1.056746
H -0.016718 -2.670319 1.600004
H 0.422480 -3.829597 0.345779
H 1.443418 -3.654441 1.770918
39
n7-a-Me-1 Eopt
-865.409317
N -0.030394 -0.094775 -0.234212
C -1.347653 -0.504886 0.122640
C -2.453343 0.141267 -0.455096
C -1.536680 -1.510926 1.077942
C -3.736296 -0.278769 -0.088133
C -2.823503 -1.919830 1.423944
H -0.670945 -1.959790 1.551781
C -3.931252 -1.310689 0.831574
H -4.594468 0.216783 -0.534384
H -2.957045 -2.703613 2.163175
H -4.936821 -1.624766 1.093039
C 1.093017 -0.948234 -0.165915
C 2.234879 -0.181337 0.174822
C 1.189090 -2.311799 -0.497686
C 3.487824 -0.789401 0.290618
C 2.458524 -2.888481 -0.358524
C 3.586123 -2.156358 0.044788
H 4.364940 -0.204403 0.549552
H 2.573857 -3.939906 -0.608122
C 0.420835 1.231933 -0.021685
C -0.289996 2.437570 -0.116922
C 1.808563 1.201810 0.266116
C 0.439599 3.604389 0.148989
C 2.503169 2.386048 0.524106
C 1.802503 3.589100 0.478423
H -0.073697 4.559519 0.071441
H 3.566057 2.367183 0.744305
H 2.316317 4.525739 0.670621
C -2.247728 1.328702 -1.355745
H -3.195631 1.588334 -1.836709
H -1.529482 1.090938 -2.149757
C -1.736846 2.543662 -0.554128
H -2.388918 2.682194 0.318263
H -1.838910 3.449437 -1.162953
H 4.546137 -2.656358 0.126440
C 0.044888 -3.119267 -1.057999
H -0.612701 -3.517742 -0.278372
H -0.583294 -2.513420 -1.717666
H 0.433069 -3.963726 -1.633963
39
n7-a-Me-TS Eopt
-865.372893
N -0.080911 -0.124615 0.314888
C -1.451864 -0.500195 0.069983
C -2.455002 0.361396 -0.459339
C -1.838531 -1.814512 0.357609
C -3.717867 -0.181490 -0.746786
C -3.093911 -2.331132 0.057630
H -1.146327 -2.453520 0.853734
C -4.052352 -1.513665 -0.530212
H -4.467716 0.490924 -1.153636
H -3.312221 -3.366232 0.302717
H -5.039905 -1.885554 -0.782658
C 1.075674 -0.977468 0.085612
C 2.156615 -0.131128 -0.295441
C 1.367120 -2.359274 0.277266
C 3.392672 -0.610736 -0.727351
C 2.606184 -2.810091 -0.216580
C 3.595137 -1.981772 -0.749662
H 4.170063 0.083770 -1.029170
H 2.824964 -3.868623 -0.108916
C 0.388537 1.220884 0.262925
C -0.271814 2.433703 0.531187
C 1.748868 1.234356 -0.100637
C 0.487231 3.606855 0.474843
C 2.486941 2.420248 -0.163282
C 1.850481 3.614739 0.147408
H -0.016806 4.548035 0.676277
H 3.537923 2.398808 -0.433400
H 2.396244 4.552577 0.125314
C -2.341578 1.857250 -0.626989
H -3.341529 2.248542 -0.835322
H -1.711478 2.129858 -1.481604
C -1.762285 2.513328 0.642530
H -2.150263 1.998196 1.529102
H -2.067307 3.561619 0.712477
H 4.530138 -2.405929 -1.100910
C 0.646116 -3.401768 1.119379
H 0.176183 -2.961955 2.002686
H -0.102633 -3.981637 0.571110
H 1.388813 -4.113737 1.487433
33
ox6-H-TS Eopt
-806.908696
C -3.686012 -2.133820 -0.000166
C -1.136761 -1.128379 0.000110
C -2.213663 -0.234350 0.000057
C -3.511384 -0.750955 -0.000072
O 0.092720 -0.306777 0.000236
C -0.328833 1.078619 0.000140
C -1.715083 1.129941 0.000078
C 0.541912 2.139709 0.000049
C -0.086893 3.395613 -0.000056
C -1.482206 3.515437 -0.000080
C -2.314303 2.391805 -0.000023
C 2.025032 1.922355 0.000060
C 2.416366 0.460352 -0.000052
C 1.534314 -0.620711 0.000111
C 1.936849 -1.947020 0.000189
C 3.303693 -2.224562 0.000024
C 4.229504 -1.182533 -0.000203
C 3.780817 0.133958 -0.000222

H -4.686818 -2.550861 -0.000273
H -4.363022 -0.079836 -0.000108
H 0.530055 4.288794 -0.000132
H -1.925568 4.505068 -0.000161
H -3.393157 2.496773 -0.000072
H 2.468735 2.408878 -0.876777
H 2.468686 2.408702 0.877024
H 1.238411 -2.762711 0.000391
H 3.629840 -3.258457 0.000081
H 5.292993 -1.393515 -0.000343
H 4.497455 0.949878 -0.000358
C -2.581171 -2.990469 -0.000126
C -1.265696 -2.503218 0.000013
H -0.449794 -3.204408 0.000050
H -2.728461 -4.064729 -0.000204
33
ox6-H Eopt -
806.915549
O -0.037812 -0.282446 0.540006
C 0.207457 1.112944 0.215665
C -0.786331 2.048273 0.344567
C -0.385167 3.344245 -0.014104
C 0.926657 3.595098 -0.449086
C 1.888121 2.582156 -0.556823
C 1.512238 1.278267 -0.215274
C 2.160465 -0.030456 -0.156765
C 1.234048 -0.971026 0.304514
C 1.504996 -2.282793 0.625548
C 2.822240 -2.695489 0.376233
C 3.779434 -1.807072 -0.131348
C 3.466805 -0.469767 -0.387532
C -2.135878 1.578979 0.827134
C -2.405750 0.173099 0.321522
C -1.385314 -0.756158 0.129466
C -1.536625 -2.015131 -0.417287
C -2.835334 -2.405852 -0.758602
C -3.906237 -1.534412 -0.559076
C -3.687993 -0.257616 -0.039481
H -1.098560 4.159697 0.046768
H 2.892595 2.805901 -0.897422
H 3.099526 -3.720391 0.596131
H 4.224340 0.220277 -0.741999
H -2.927048 2.251631 0.488731
H -2.155604 1.591409 1.925945
H -0.696400 -2.670169 -0.597300
H -2.995423 -3.388455 -1.187969
H -4.911482 -1.840952 -0.826230
H -4.519901 0.429037 0.081788
H 1.202214 4.609975 -0.714061
H 4.789226 -2.160736 -0.307711
H 0.772026 -2.951391 1.057659
36
ox6-a-Cl-b-Me-TS Eopt
-1305.784390
C -4.528312 0.005597 -0.166695
C -4.166889 1.347369 -0.101683
C -2.803320 1.773532 0.025615
C -2.075063 0.643231 0.082698
C -2.169873 -0.705361 0.053526
C -3.570394 -0.993272 -0.095427
O 0.451500 -0.060025 -0.339048
C 0.199295 -1.395298 -0.128083
C -1.145777 -1.746694 0.075202
C 1.212812 -2.358070 -0.120081
C 0.859755 -3.686656 0.120589
C -0.468882 -4.057121 0.337913
C -1.467638 -3.092246 0.310489
C 2.629925 -1.947901 -0.417181
C 2.847326 -0.482274 -0.144324
C 1.757037 0.393159 -0.150885
C 1.955858 1.765325 0.016604
C 3.233338 2.280893 0.216983
C 4.322299 1.410813 0.243332
C 4.125366 0.042821 0.061822
H -5.572791 -0.262399 -0.281303
H -4.907974 2.141368 -0.087345
H -3.834564 -2.036679 -0.226263
H 1.640656 -4.441061 0.133528
H -0.721618 -5.092101 0.537655
H -2.491563 -3.384482 0.509112
H 3.327041 -2.549708 0.173772
H 2.856788 -2.159037 -1.472365
H 3.362490 3.348769 0.348325
H 5.320862 1.803573 0.399123
H 4.974130 -0.634014 0.068153
C -2.475061 3.234011 0.168902
H -1.939229 3.587540 -0.715036
H -3.402007 3.799934 0.262239
H -1.860623 3.408740 1.053099
Cl 0.588565 2.844624 -0.019188
36
ox6-a-Cl-b-Me Eopt
-1305.824625
O -0.015643 0.126332 0.539952
C 0.157024 1.516244 0.154488
C -0.844682 2.414920 0.405192
C -0.540980 3.719107 -0.012809
C 0.703725 4.002149 -0.602635
C 1.694626 3.027191 -0.785828
C 1.413256 1.715486 -0.383675
C 2.147914 0.454574 -0.252654
C 1.323743 -0.492843 0.367392
C 1.700324 -1.696565 0.923109
C 3.049451 -2.015811 0.666850
C 3.905178 -1.157880 -0.030750
C 3.477833 0.097802 -0.474805
C -2.069355 1.875531 1.107942
C -2.381466 0.482966 0.580374
C -1.353803 -0.376958 0.175383
C -1.572218 -1.526942 -0.580911
C -2.899994 -1.931405 -0.765568
C -3.948657 -1.159479 -0.275640
C -3.691971 0.059098 0.353406
H -1.264568 4.514759 0.130886
H 2.654656 3.289995 -1.215158
H 3.431937 -2.956804 1.049894
H 4.166153 0.789350 -0.947167
H -2.935100 2.523453 0.957946
H -1.875100 1.842978 2.189113
H -3.088721 -2.836424 -1.331365
H -4.970760 -1.484663 -0.434772
H -4.511466 0.703348 0.654136
C 0.806719 -2.590701 1.739403
H -0.100519 -2.079085 2.066796
H 0.510367 -3.475221 1.168462
H 1.340723 -2.931434 2.630011
H 0.910565 5.020624 -0.912954
H 4.935105 -1.457229 -0.191777
Cl -0.325772 -2.410189 -1.410780
36
ox6-a-Me-TS Eopt
-846.221794
C -3.973829 -1.366455 -0.000085
C -3.035077 -2.393576 -0.000037
C -1.630782 -2.212259 0.000025
C -1.259530 -0.862535 0.000027
C -2.175060 0.200801 -0.000022
C -3.546733 -0.047355 -0.000078
O 0.122557 -0.159867 0.000122
C -0.141817 1.264998 0.000042
C -1.507166 1.481773 -0.000016
C 0.823154 2.247470 0.000013
C 0.327725 3.560714 -0.000055
C -1.044379 3.831015 -0.000094
C -1.980104 2.795601 -0.000081
C 2.278654 1.913178 0.000043
C 2.529854 0.427689 -0.000006
C 1.554059 -0.571716 0.000062
C 1.861084 -1.917110 0.000123
C 3.197116 -2.314873 0.000062
C 4.208242 -1.356868 -0.000049
C 3.863332 -0.010884 -0.000074
H -5.031235 -1.606058 -0.000128
H -3.389304 -3.418187 -0.000048
H -4.246426 0.780406 -0.000115
H 1.038853 4.381035 -0.000081
H -1.383243 4.861049 -0.000146
H -3.043783 3.003892 -0.000125
H 2.762376 2.359788 -0.877020
H 2.762319 2.359705 0.877183
H 1.095461 -2.660636 0.000226
H 3.430399 -3.373788 0.000106
H 5.251242 -1.652988 -0.000103
H 4.640901 0.747308 -0.000135
C -0.833555 -3.496272 0.000065
H -1.528376 -4.336131 0.000025
H -0.215331 -3.603238 -0.894755
H -0.215433 -3.603243 0.894956
39
ox6-a-Me-b-Me-TS Eopt
-885.507343
C 3.744425 -1.470034 0.939263
C 2.896631 -2.440322 0.420120
C 1.645912 -2.183500 -0.198103
C 1.259981 -0.843171 -0.105964
C 2.152136 0.170532 0.266811
C 3.397821 -0.132527 0.811022
O -0.057124 -0.084353 -0.589963
C 0.310730 1.334750 -0.448545
C 1.616176 1.475161 -0.020192
C -0.570627 2.391348 -0.550529
C -0.014284 3.671275 -0.419594
C 1.341436 3.852737 -0.130359
C 2.162931 2.753586 0.115132
C -2.030825 2.107932 -0.559622
C -2.282466 0.803065 0.156937
C -1.512019 -0.344768 -0.092492
C -2.076526 -1.618488 -0.046863
C -3.231729 -1.732340 0.754890
C -3.874404 -0.629361 1.296067
C -3.450114 0.639288 0.904247
H 4.691480 -1.759869 1.380513
H 3.220574 -3.475479 0.430920
H 4.067701 0.665466 1.109438
H -0.671765 4.530852 -0.503109
H 1.740340 4.856911 -0.038810
H 3.193947 2.883292 0.423323
H -2.578850 2.920095 -0.077338

H -2.427659 2.015291 -1.580322
H -3.667873 -2.721706 0.858381
H -4.759389 -0.751938 1.910359
H -4.042963 1.518488 1.134590
C 1.072051 -3.370297 -0.925874
H 0.686631 -3.102640 -1.907837
H 1.878218 -4.086834 -1.086269
H 0.296947 -3.880287 -0.357750
C -1.839478 -2.744289 -1.019197
H -1.612875 -3.695805 -0.538905
H -2.780286 -2.877951 -1.562492
H -1.083738 -2.506525 -1.757926
39
ox6-a-Me-b-Me Eopt
-885.563552
O -0.026665 -0.025574 0.498746
C 0.038940 1.386338 0.184496
C -1.030752 2.191466 0.471442
C -0.831190 3.533108 0.114611
C 0.385104 3.940570 -0.462344
C 1.446781 3.055275 -0.696195
C 1.269776 1.708571 -0.354602
C 2.092988 0.497299 -0.300416
C 1.338243 -0.532486 0.279388
C 1.806576 -1.738991 0.757754
C 3.164137 -1.962277 0.449905
C 3.949690 -1.012964 -0.211589
C 3.436995 0.238505 -0.568929
C -2.212406 1.517583 1.129672
C -2.415590 0.144203 0.507443
C -1.334016 -0.621644 0.060269
C -1.408265 -1.731694 -0.776801
C -2.720394 -2.191188 -1.003526
C -3.838123 -1.541163 -0.490718
C -3.688512 -0.354052 0.226162
H -1.615046 4.261858 0.293099
H 2.379424 3.411313 -1.118732
H 3.612230 -2.899324 0.765521
H 4.070609 0.996925 -1.014508
H -3.123524 2.108325 1.017368
H -2.017279 1.428687 2.207557
H -2.847314 -3.065827 -1.633248
H -4.829618 -1.928605 -0.697865
H -4.558263 0.208012 0.550466
H 0.510065 4.985595 -0.724513
H 4.990738 -1.239624 -0.414616
C 1.009604 -2.702209 1.597705
H -0.067923 -2.584964 1.474551
H 1.275426 -3.735475 1.361553
H 1.239635 -2.538410 2.656309
C -0.268585 -2.400486 -1.508912
H 0.456790 -1.680645 -1.891138
H 0.268815 -3.120977 -0.888702
H -0.677455 -2.944864 -2.361976
36
ox6-a-Me Eopt
-846.243659
O -0.030562 -0.156512 0.529798
C -0.061567 1.276211 0.275653
C -1.206642 1.993787 0.506929
C -1.087195 3.355362 0.190983
C 0.118685 3.868056 -0.316841
C 1.241786 3.062438 -0.546027
C 1.147152 1.698607 -0.245245
C 2.031314 0.534615 -0.300727
C 1.344492 -0.580882 0.198930
C 1.884443 -1.812187 0.518285
C 3.237921 -1.934245 0.137858
C 3.954892 -0.883936 -0.440986
C 3.372586 0.371542 -0.642968
C -2.409609 1.229426 1.006225
C -2.439759 -0.130928 0.330377
C -1.264688 -0.815147 0.025142
C -1.177994 -1.972130 -0.721426
C -2.382730 -2.543156 -1.144267
C -3.597517 -1.934471 -0.826898
C -3.621711 -0.734022 -0.113667
H -1.936202 4.015486 0.335473
H 2.154225 3.486897 -0.948959
H 3.739050 -2.876518 0.336660
H 3.951584 1.202178 -1.030210
H -3.334821 1.770288 0.797535
H -2.344885 1.110773 2.096727
H -0.226475 -2.412338 -0.983807
H -2.359410 -3.455910 -1.728973
H -4.528898 -2.380786 -1.157474
H -4.567268 -0.241593 0.090298
H 0.177300 4.926081 -0.548102
H 4.996602 -1.036874 -0.701339
C 1.171108 -2.918317 1.252250
H 0.257846 -2.571099 1.737959
H 0.905535 -3.742136 0.581771
H 1.830505 -3.326080 2.022587
42
ox6-a-iPr-TS Eopt
-924.862654
C 2.960641 -2.699450 0.043179
C 3.295238 -1.349594 0.081302
C 2.373688 -0.276215 0.104518
C 1.044616 -0.721212 0.069148
C 0.662510 -2.068485 0.037286
C 1.627778 -3.074736 0.025667
O -0.350387 0.040688 0.051109
C -1.366188 -0.990733 0.011109
C -0.769999 -2.237640 0.009825
C -2.726278 -0.768921 -0.028386
C -3.519731 -1.925941 -0.061265
C -2.958773 -3.206072 -0.058250
C -1.574946 -3.377838 -0.024794
C -3.294461 0.610467 -0.042679
C -2.228199 1.669669 0.031655
C -0.848769 1.447678 0.077401
C 0.062022 2.482033 0.144308
C -0.387171 3.800969 0.169401
C -1.752272 4.071465 0.126665
C -2.645331 3.009675 0.059335
H 3.747007 -3.445883 0.030514
H 4.344033 -1.089835 0.097501
H 1.323991 -4.114804 0.001483
H -4.598763 -1.809685 -0.091170
H -3.608864 -4.073511 -0.084487
H -1.134923 -4.368342 -0.026288
H -3.887020 0.758237 -0.953917
H -3.987732 0.737172 0.797326
H 1.111040 2.291702 0.174896
H 0.341192 4.602463 0.221646
H -2.116736 5.092411 0.146116
H -3.713734 3.201844 0.026132
C 2.974493 1.127401 0.105303
H 2.408762 1.723716 0.821817
C 4.418789 1.208254 0.629218
H 5.144523 0.778323 -0.066834
H 4.681264 2.262985 0.748839
H 4.530408 0.722865 1.602654
C 2.904154 1.730551 -1.314105
H 3.230737 2.774908 -1.303571
H 3.574231 1.174507 -1.976659
H 1.902826 1.687480 -1.749917
42
ox6-a-iPr Eopt -
924.890632
O 0.237275 0.021074 0.393110
C 1.375342 -0.881219 0.303263
C 2.624224 -0.456444 0.677633
C 3.621558 -1.427980 0.507230
C 3.303643 -2.694503 -0.012131
C 2.002841 -3.045124 -0.396470
C 0.989843 -2.091410 -0.241893
C -0.451365 -2.025047 -0.481757
C -0.926243 -0.773582 -0.064345
C -2.245432 -0.389918 0.083166
C -3.150467 -1.358466 -0.402681
C -2.736193 -2.593376 -0.908060
C -1.386143 -2.957405 -0.928154
C 2.750997 0.971972 1.151181
C 1.825665 1.842989 0.318679
C 0.593352 1.369121 -0.126509
C -0.246027 2.016520 -1.009166
C 0.145204 3.289466 -1.436094
C 1.345236 3.842140 -0.986952
C 2.181409 3.118416 -0.134009
H 4.647611 -1.195143 0.772680
H 1.792509 -4.026537 -0.805934
H -4.211582 -1.141663 -0.350220
H -1.074853 -3.941648 -1.259064
H 3.779505 1.327414 1.062454
H 2.478480 1.035039 2.213794
H -1.158888 1.560856 -1.365982
H -0.489765 3.834991 -2.125165
H 1.644685 4.829457 -1.320888
H 3.134503 3.535239 0.175896
H 4.098556 -3.423085 -0.128936
H -3.483596 -3.298096 -1.256237
C -2.720592 0.862126 0.803009
H -1.856574 1.508829 0.978460
C -3.286537 0.471952 2.183599
H -3.578119 1.371373 2.733752
H -4.171449 -0.163487 2.080182
H -2.547417 -0.071108 2.780031
C -3.751016 1.658011 -0.013768
H -4.006989 2.581443 0.512765
H -3.374162 1.926786 -1.005336
H -4.676441 1.091310 -0.151763
36
ox6-b-Me-TS Eopt
-846.214393
C -3.579280 -2.295678 0.360117
C -2.462579 -3.063130 0.022852
C -1.218726 -2.479257 -0.259325
C -1.142504 -1.107226 -0.154741
C -2.249727 -0.306729 0.138368
C -3.481484 -0.907802 0.406940
O 0.021707 -0.190502 -0.461527
C -0.533533 1.156842 -0.257326
C -1.877983 1.086837 0.066771
C 0.204216 2.312577 -0.335909
C -0.512049 3.499858 -0.125913
C -1.882979 3.483002 0.153264

C	-2.579570	2.278409	0.265129
C	1.672887	2.213418	-0.552836
C	2.203871	0.901509	-0.026001
C	1.531307	-0.326464	-0.132964
C	2.184776	-1.561101	-0.047030
C	3.493742	-1.505298	0.474720
C	4.138678	-0.318109	0.783103
C	3.509439	0.880105	0.471627
H	-4.526250	-2.781054	0.568195
H	-2.542952	-4.142835	-0.038647
H	-4.343689	-0.291667	0.636541
H	0.021437	4.443989	-0.170185
H	-2.405831	4.420662	0.306044
H	-3.635547	2.263981	0.509579
H	1.909000	2.275851	-1.624884
H	2.189837	3.044896	-0.069025
H	4.020118	-2.447289	0.588888
H	5.143641	-0.327683	1.189863
H	4.031323	1.824602	0.584549
H	-0.406517	-3.111708	-0.545326
C	1.784199	-2.914632	-0.588899
H	1.288611	-3.542120	0.155539
H	1.170869	-2.831924	-1.486563
H	2.696083	-3.436477	-0.884029

36

ox6-b-Me Eopt

-846.234364

O	0.012378	-0.133906	-0.503326
C	-0.306336	1.222698	-0.113229
C	0.607551	2.222936	-0.311717
C	0.152044	3.485085	0.097393
C	-1.143403	3.639283	0.621681
C	-2.041666	2.570073	0.741817
C	-1.607747	1.297890	0.349470
C	-2.203722	-0.027237	0.167328
C	-1.242603	-0.876263	-0.393133
C	-1.473456	-2.125271	-0.920138
C	-2.782036	-2.603123	-0.764071
C	-3.769584	-1.825011	-0.143882
C	-3.500303	-0.530515	0.308323
C	1.902693	1.832544	-0.981598
C	2.337576	0.452685	-0.514440
C	1.415061	-0.538037	-0.160272
C	1.705218	-1.719057	0.518213
C	3.082635	-1.969413	0.673311
C	4.056954	-1.074056	0.246706
C	3.684127	0.150167	-0.306947
H	0.798158	4.350516	-0.007616
H	-3.046966	2.732124	1.113556
H	-3.030614	-3.587380	-1.145037
H	-4.285698	0.083926	0.734131
H	2.694045	2.553892	-0.767755
H	1.751642	1.838254	-2.070384
H	3.375164	-2.883553	1.179734
H	5.105872	-1.306273	0.394598
H	4.434790	0.890608	-0.563532
H	-1.466296	4.629371	0.924681
H	-4.772256	-2.225517	-0.042434
H	-0.706120	-2.700050	-1.422458
C	0.725944	-2.671869	1.162944
H	-0.137301	-2.160264	1.590311
H	0.356570	-3.430690	0.468361
H	1.236314	-3.194775	1.973891

39

ox7-a-Me-1 Eopt

-885.575527

O	-0.006096	-0.107978	-0.470299
C	-1.166928	-0.788134	0.144121
C	-2.387042	-0.463379	-0.430315
C	-0.972724	-1.637381	1.216647
C	-3.502621	-1.103975	0.125735
C	-2.109873	-2.256237	1.743398
H	0.013067	-1.818421	1.627292
C	-3.366474	-1.996470	1.191223
H	-4.485051	-0.891350	-0.284153
H	-2.005962	-2.935256	2.582335
H	-4.245778	-2.483161	1.599052
C	1.332111	-0.684690	-0.293936
C	2.169004	0.299753	0.227348
C	1.680221	-1.967125	-0.675204
C	3.504031	-0.036119	0.457624
C	3.033365	-2.265307	-0.418482
C	3.915554	-1.333624	0.140908
H	4.202351	0.692318	0.853667
H	3.398357	-3.251903	-0.685791
C	0.119888	1.323257	-0.130777
C	-0.882125	2.255330	-0.308949
C	1.415406	1.546807	0.332633
C	-0.497915	3.550470	0.098656
C	1.754320	2.843936	0.715638
C	0.773959	3.835271	0.603020
H	-1.222384	4.352866	-0.002496
H	2.749197	3.077868	1.077055
H	1.009193	4.853250	0.893960
C	-2.485730	0.600698	-1.492453
H	-3.480236	0.570350	-1.943013
H	-1.764774	0.413658	-2.296153
C	-2.255554	2.003798	-0.896557
H	-3.005810	2.188469	-0.118407
H	-2.427529	2.753706	-1.676011
H	4.947906	-1.621040	0.308379
C	0.740674	-2.957109	-1.309146
H	0.179759	-3.515444	-0.552319
H	0.013313	-2.465039	-1.960044
H	1.303866	-3.675682	-1.907762

39

ox7-a-Me-2 Eopt

-885.562895

O	-0.000867	-0.087248	0.567573
C	-1.047051	-0.949965	-0.096982
C	-2.389932	-0.682383	0.156514
C	-0.581294	-1.940109	-0.951195
C	-3.284905	-1.569034	-0.476281
C	-1.508328	-2.783747	-1.557687
H	0.474195	-2.056233	-1.152419
C	-2.868530	-2.605624	-1.302912
H	-4.346793	-1.411289	-0.311638
H	-1.161823	-3.564794	-2.225054
H	-3.602967	-3.258018	-1.762288
C	1.413263	-0.447199	0.339944
C	2.024216	0.574059	-0.391472
C	2.008167	-1.582671	0.852245
C	3.373127	0.422842	-0.711837
C	3.370097	-1.695513	0.503822
C	4.027513	-0.731345	-0.267421
H	3.903726	1.188601	-1.266164
H	3.921386	-2.557553	0.865812
C	-0.087008	1.316099	0.140985
C	-1.134797	2.115326	0.528733
C	1.074188	1.681173	-0.522721
C	-1.042668	3.428397	0.034482
C	1.141720	2.996413	-0.992173
C	0.059021	3.844338	-0.728427
H	-1.824613	4.139117	0.282167
H	2.017531	3.360104	-1.517461
H	0.092749	4.868464	-1.084057
C	-3.031004	0.452324	0.942412
H	-3.595752	0.014829	1.773256
H	-3.776182	0.892897	0.271123
C	-2.157067	1.585997	1.496909
H	-1.623985	1.240507	2.391103
H	-2.812369	2.398052	1.820825
H	5.077736	-0.870225	-0.500310
C	1.295504	-2.605874	1.693543
H	0.501881	-2.152431	2.292605
H	0.836444	-3.380543	1.069842
H	2.000374	-3.094539	2.369192

39

ox7-a-Me-TS Eopt

-885.532520

O	-0.067975	-0.123534	0.507839
C	-1.457420	-0.550194	0.094977
C	-2.369506	0.288005	-0.561243
C	-1.782230	-1.830539	0.500794
C	-3.558478	-0.345846	-0.972190
C	-2.980474	-2.411789	0.094307
H	-1.129269	-2.366302	1.156706
C	-3.857368	-1.675385	-0.696561
H	-4.287684	0.264702	-1.495122
H	-3.219076	-3.420711	0.412021
H	-4.790155	-2.106036	-1.043327
C	1.166861	-0.967501	0.119302
C	2.116926	-0.054403	-0.346315
C	1.443222	-2.320050	0.341314
C	3.336519	-0.499028	-0.851464
C	2.674089	-2.725934	-0.228817
C	3.584598	-1.866624	-0.837443
H	4.067880	0.209979	-1.221902
H	2.932170	-3.775162	-0.130553
C	0.368386	1.280864	0.366367
C	-0.384353	2.412715	0.640068
C	1.668580	1.300366	-0.113587
C	0.324789	3.621202	0.528401
C	2.340114	2.519029	-0.238358
C	1.661065	3.679752	0.121098
H	-0.213334	4.541532	0.731399
H	3.363452	2.551973	-0.593900
H	2.157345	4.641591	0.053294
C	-2.343920	1.797401	-0.650792
H	-3.359035	2.125476	-0.884510
H	-1.699913	2.162804	-1.457436
C	-1.881653	2.408609	0.692845
H	-2.287660	1.833525	1.531024
H	-2.241555	3.434599	0.793157
H	4.511098	-2.263029	-1.237692
C	0.752214	-3.372325	1.181027
H	0.329229	-2.956377	2.098533
H	-0.022289	-3.916105	0.634475
H	1.500827	-4.104133	1.488744

39

ox7-a-Me-flip-TS Eopt

-885.562366

O	-0.016661	-0.103593	0.611117
C	0.954465	-1.010723	-0.096365
C	2.302130	-0.870205	0.218888
C	0.439706	-1.864567	-1.059109
C	3.156408	-1.729119	-0.498637
C	1.326985	-2.699566	-1.736740

H -0.617030 -1.883979 -1.287950
C 2.689711 -2.635354 -1.446481
H 4.221549 -1.668051 -0.297789
H 0.948047 -3.381079 -2.490035
H 3.391002 -3.276970 -1.968618
C -1.450467 -0.356232 0.375825
C -1.983563 0.712641 -0.346794
C -2.118828 -1.465710 0.852735
C -3.338253 0.658679 -0.675521
C -3.483227 -1.479633 0.497900
C -4.071201 -0.455638 -0.253071
H -3.811928 1.465061 -1.223749
H -4.091978 -2.312561 0.834971
C 0.183590 1.287764 0.173805
C 1.315780 1.991019 0.510230
C -0.948286 1.738919 -0.487927
C 1.334469 3.290021 -0.029133
C -0.903698 3.040474 -0.996420
C 0.260071 3.787956 -0.781475
H 2.186973 3.929312 0.176964
H -1.750601 3.466839 -1.521935
H 0.317929 4.798561 -1.171009
C 2.938898 0.069908 1.225705
H 3.046463 -0.467128 2.175325
H 3.956941 0.239992 0.867403
C 2.289572 1.447853 1.522066
H 1.744865 1.388413 2.471786
H 3.084856 2.180149 1.677679
H -5.127207 -0.519063 -0.492537
C -1.470384 -2.557832 1.658070
H -0.987246 -3.294217 1.006530
H -0.704994 -2.160540 2.330112
H -2.217622 -3.079926 2.258612
45
ox7-a-iPr-1 Eopt
-964.222406
O -0.226212 -0.013571 0.348325
C -0.219497 1.307813 -0.318282
C -0.959943 2.278495 0.340538
C 0.460289 1.471786 -1.509632
C -0.962822 3.544007 -0.261214
C 0.432614 2.747905 -2.078899
H 0.995282 0.653914 -1.975676
C -0.267576 3.779729 -1.449448
H -1.522143 4.345836 0.210658
H 0.957256 2.926440 -3.010907
H -0.281299 4.770037 -1.891573
C 0.847866 -0.970873 0.036863
C 0.259134 -2.153675 -0.410267
C 2.191087 -0.724227 0.240171
C 1.102833 -3.207460 -0.760688
C 3.006111 -1.811228 -0.139886
C 2.481444 -3.009523 -0.634170
H 0.701409 -4.153292 -1.106246
H 4.080193 -1.715289 -0.024060
C -1.490685 -0.756710 0.188378
C -2.730652 -0.260072 0.535187
C -1.192713 -2.021177 -0.316425
C -3.774442 -1.172793 0.273404
C -2.254366 -2.893806 -0.552016
C -3.546533 -2.444028 -0.260658
H -4.789221 -0.870202 0.513428
H -2.082050 -3.892462 -0.936906
H -4.391547 -3.101384 -0.434255
C -1.788239 1.930510 1.549930
H -2.129976 2.849322 2.031619
H -1.185974 1.387579 2.286847
C -3.020289 1.090909 1.156735
H -3.636704 1.668373 0.457456
H -3.636140 0.924789 2.047152
H 3.161209 -3.810916 -0.902941
C 2.759089 0.538568 0.864609
H 1.933756 1.237352 1.031979
C 3.377580 0.215897 2.238378
H 4.233448 -0.458642 2.137993
H 3.729821 1.136041 2.713665
H 2.649823 -0.258285 2.903614
C 3.775124 1.227707 -0.060947
H 3.333080 1.480221 -1.028643
H 4.131187 2.152737 0.401208
H 4.645807 0.589871 -0.240969
45
ox7-a-iPr-2 Eopt
-964.210020
O 0.215383 -0.010145 0.398372
C 0.361316 1.317340 -0.306436
C 1.380964 2.175682 0.097258
C -0.527661 1.571438 -1.341436
C 1.407773 3.405464 -0.591457
C -0.453843 2.798969 -1.994203
H -1.263192 0.838795 -1.640341
C 0.511191 3.727704 -1.603285
H 2.180195 4.117549 -0.315935
H -1.143963 3.015750 -2.801858
H 0.578240 4.690674 -2.097511
C -0.926806 -0.864594 -0.001852
C -0.426267 -2.008669 -0.630230
C -2.243930 -0.568099 0.279256
C -1.347263 -2.946459 -1.094166
C -3.139931 -1.541778 -0.212113
C -2.707189 -2.685329 -0.889644
H -1.018711 -3.859490 -1.577541
H -4.201598 -1.399517 -0.042696
C 1.390864 -0.866285 0.195484
C 2.594921 -0.573340 0.788364
C 1.033386 -2.004731 -0.509963
C 3.602878 -1.506387 0.488186
C 2.054552 -2.919639 -0.783976
C 3.337896 -2.636088 -0.300739
H 4.593756 -1.362988 0.907134
H 1.855267 -3.836466 -1.327096
H 4.143189 -3.334117 -0.502579
C 2.498247 1.959923 1.107858
H 2.382226 2.699610 1.907956
H 3.424385 2.224043 0.585907
C 2.688402 0.578669 1.750901
H 1.932691 0.425646 2.530952
H 3.659372 0.562980 2.251661
H -3.444083 -3.399477 -1.241229
C -2.698586 0.637839 1.081947
H -1.829781 1.284886 1.240616
C -3.765246 1.458857 0.339401
H -3.410150 1.794106 -0.639075
H -4.682054 0.880932 0.189141
H -4.025732 2.344228 0.926009
C -3.201631 0.184462 2.466260
H -3.476864 1.055924 3.067420
H -4.086063 -0.453503 2.373026
H -2.434429 -0.378103 3.006585
45
ox7-a-iPr-TS Eopt
-964.174714
O 0.283229 0.067235 0.421113
C 0.303918 1.524601 0.048360
C 1.376604 2.137467 -0.616428
C -0.791463 2.236916 0.497094
C 1.158079 3.476937 -0.987232
C -0.957576 3.571607 0.133448
H -1.507190 1.770414 1.143820
C 0.004683 4.183939 -0.662457
H 1.961649 3.982965 -1.512633
H -1.828826 4.113842 0.483083
H -0.107571 5.215638 -0.976971
C -0.837293 -0.888554 -0.080265
C -0.176925 -2.061329 -0.457636
C -2.227859 -0.765133 -0.032045
C -0.876244 -3.093483 -1.077422
C -2.877644 -1.801263 -0.747384
C -2.240409 -2.915929 -1.282542
H -0.364396 -3.999041 -1.381966
H -3.956656 -1.741582 -0.821503
C 1.517031 -0.745247 0.410416
C 2.799460 -0.325154 0.738441
C 1.210244 -2.014742 -0.054580
C 3.757229 -1.353784 0.759640
C 2.191164 -3.009785 -0.051757
C 3.461927 -2.673538 0.404590
H 4.777148 -1.085186 1.015442
H 1.960273 -4.012922 -0.391061
H 4.243413 -3.424569 0.439556
C 2.796837 1.630501 -0.705336
H 3.427116 2.466823 -1.014554
H 2.929151 0.839839 -1.450403
C 3.236248 1.109289 0.687626
H 2.804082 1.731886 1.476965
H 4.322270 1.154143 0.792056
H -2.823160 -3.670934 -1.798517
C -3.147492 0.159493 0.765689
H -2.551785 0.651456 1.533091
C -3.842007 1.202264 -0.125900
H -4.444064 1.882030 0.484581
H -3.130184 1.791659 -0.707248
H -4.515299 0.705434 -0.831245
C -4.187740 -0.649991 1.569322
H -4.704114 0.025627 2.257636
H -4.948968 -1.105687 0.931398
H -3.713345 -1.440204 2.157721
48
ox7-a-tBu-1 Eopt
-1003.539358
O -0.282419 -0.033025 0.356530
C -0.429244 1.217854 -0.429570
C -1.198861 2.189298 0.196198
C 0.135847 1.312846 -1.685402
C -1.363333 3.380939 -0.521094
C -0.054747 2.516527 -2.371561
H 0.707052 0.500054 -2.115472
C -0.792656 3.546905 -1.785643
H -1.948515 4.180864 -0.078255
H 0.376256 2.639493 -3.358897
H -0.931752 4.481258 -2.318570
C 0.821212 -0.952375 -0.011391
C 0.234770 -2.136508 -0.472910
C 2.177443 -0.693460 0.135073
C 1.060698 -3.148180 -0.954397
C 2.964860 -1.737102 -0.408394
C 2.436349 -2.911397 -0.947145
H 0.648436 -4.084428 -1.312973

H 4.041364 -1.630791 -0.378939
C -1.520026 -0.836622 0.294097
C -2.755986 -0.393101 0.718455
C -1.208211 -2.073269 -0.261081
C -3.774642 -1.349306 0.520334
C -2.243778 -2.989971 -0.437229
C -3.529625 -2.604168 -0.043701
H -4.783465 -1.091580 0.828296
H -2.057002 -3.972338 -0.855835
H -4.355314 -3.296484 -0.167226
C -1.871692 1.908427 1.514238
H -2.232092 2.844869 1.945816
H -1.153218 1.484275 2.223699
C -3.067388 0.951271 1.342185
H -3.831114 1.440593 0.726532
H -3.526037 0.775531 2.321612
H 3.114629 -3.665445 -1.331668
C 2.859405 0.502179 0.827704
C 3.358614 1.507294 -0.233634
H 2.531273 1.953563 -0.789973
H 3.908322 2.315820 0.258109
H 4.033839 1.025666 -0.947314
C 1.926176 1.218327 1.824016
H 1.463169 0.513194 2.521573
H 2.513283 1.928151 2.412317
H 1.147336 1.794573 1.325700
C 4.074656 -0.010285 1.638158
H 3.781290 -0.787152 2.350674
H 4.870486 -0.406135 1.003796
H 4.502077 0.823921 2.200929
48
ox7-a-tBu-2 Eopt
-1003.528463
O 0.282742 -0.028791 0.385018
C 0.510079 1.230626 -0.416420
C 1.513266 2.100885 0.002932
C -0.277754 1.407349 -1.544070
C 1.641194 3.259688 -0.788118
C -0.108133 2.568802 -2.294402
H -1.009986 0.669458 -1.838066
C 0.847915 3.506255 -1.903338
H 2.405582 3.978259 -0.507363
H -0.718517 2.727361 -3.176385
H 0.988483 4.417334 -2.474529
C -0.848324 -0.890395 -0.051797
C -0.298165 -2.025202 -0.669199
C -2.189326 -0.617329 0.155759
C -1.163299 -2.951375 -1.241421
C -3.020755 -1.576480 -0.474907
C -2.534690 -2.688541 -1.162019
H -0.786631 -3.849354 -1.717795
H -4.093599 -1.448425 -0.401310
C 1.461264 -0.894594 0.279728
C 2.630405 -0.583989 0.929410
C 1.148930 -2.031651 -0.443682
C 3.655006 -1.519081 0.701657
C 2.185412 -2.947925 -0.648024
C 3.436219 -2.658997 -0.087675
H 4.621244 -1.367484 1.172090
H 2.022640 -3.867657 -1.198390
H 4.252387 -3.358357 -0.233522
C 2.502574 1.959919 1.150624
H 2.258914 2.713291 1.908512
H 3.474888 2.250461 0.739890
C 2.663675 0.600902 1.854989
H 1.862270 0.470939 2.591773
H 3.602199 0.610303 2.414085
H -3.240149 -3.379523 -1.610842
C -2.823230 0.515603 0.982923
C -1.810554 1.277444 1.859465
H -1.234297 0.599912 2.496812
H -1.122738 1.887629 1.272467
H -2.359090 1.959271 2.514441
C -3.526852 1.512747 0.035999
H -4.037249 2.280025 0.626009
H -2.810948 2.015057 -0.620110
H -4.275434 1.015502 -0.587519
C -3.870535 -0.109676 1.936726
H -3.411679 -0.854742 2.593815
H -4.300893 0.677588 2.562020
H -4.695268 -0.586431 1.402913
48
ox7-a-tBu-TS Eopt
-1003.481295
O 0.368770 0.061212 0.329574
C 0.454826 1.513899 -0.057680
C 1.557842 2.065108 -0.730108
C -0.594363 2.291708 0.393247
C 1.418403 3.412954 -1.105028
C -0.677236 3.635696 0.030995
H -1.344289 1.872961 1.030419
C 0.313053 4.189646 -0.770956
H 2.248816 3.868975 -1.634329
H -1.515820 4.227135 0.380970
H 0.261252 5.226500 -1.084414
C -0.754732 -0.905330 -0.210432
C -0.073981 -2.083208 -0.525509
C -2.155371 -0.795628 -0.216087
C -0.726901 -3.125051 -1.180636
C -2.744267 -1.837745 -0.974191
C -2.071290 -2.948528 -1.476266
H -0.193070 -4.031761 -1.440443
H -3.815258 -1.796355 -1.112196
C 1.585456 -0.769377 0.410702
C 2.864837 -0.363598 0.775591
C 1.282685 -2.047015 -0.031087
C 3.793247 -1.412227 0.894697
C 2.236767 -3.063630 0.063392
C 3.485999 -2.739854 0.582309
H 4.806006 -1.153994 1.187089
H 2.001452 -4.072488 -0.255136
H 4.245598 -3.505913 0.692386
C 2.952890 1.490816 -0.789684
H 3.621796 2.274725 -1.150247
H 3.052745 0.644618 -1.475870
C 3.353237 1.049856 0.644629
H 2.931350 1.739497 1.381714
H 4.438312 1.062191 0.766496
H -2.621675 -3.702661 -2.027803
C -3.115652 0.116128 0.577366
C -2.547848 0.318716 2.014382
H -3.015217 1.185111 2.492524
H -2.792685 -0.558419 2.619681
H -1.465942 0.428807 2.080488
C -3.456395 1.399840 -0.226950
H -3.928693 2.142828 0.424126
H -2.602718 1.857928 -0.718305
H -4.172726 1.147141 -1.013494
C -4.480041 -0.582587 0.824209
H -4.359089 -1.571388 1.274854
H -5.050593 0.037112 1.522599
H -5.086700 -0.675764 -0.079140

7 NMR Spectra

2-Bromo-4-methylphenol, S1:

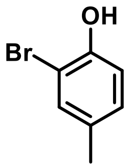

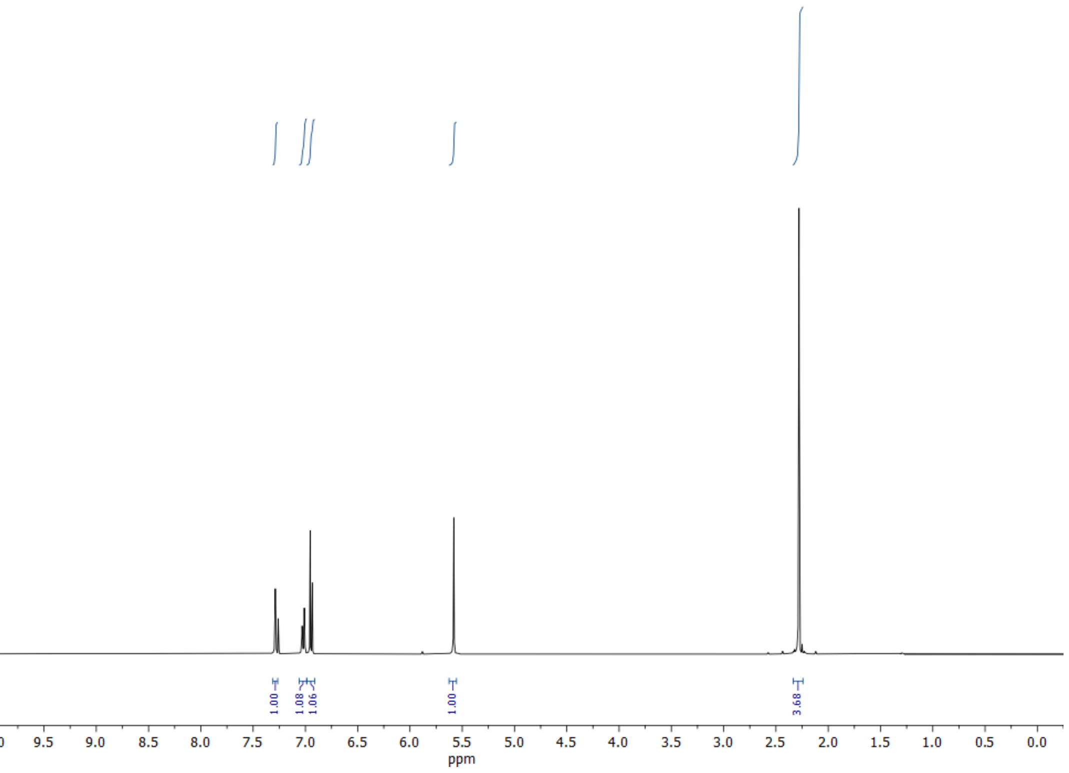

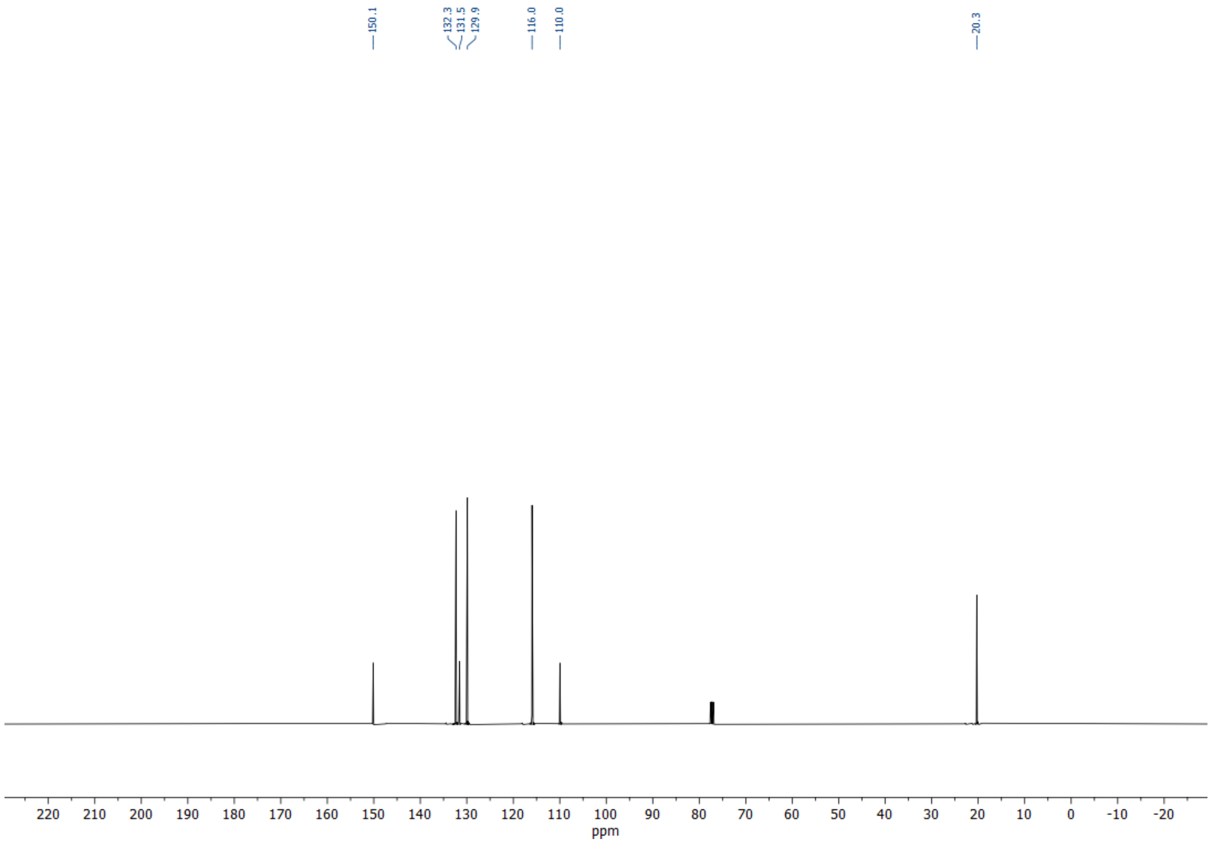

2-Bromo-4-methylphenyl 2-bromobenzoate, S2:

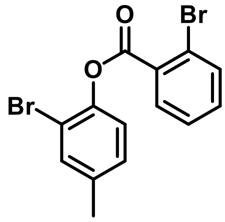

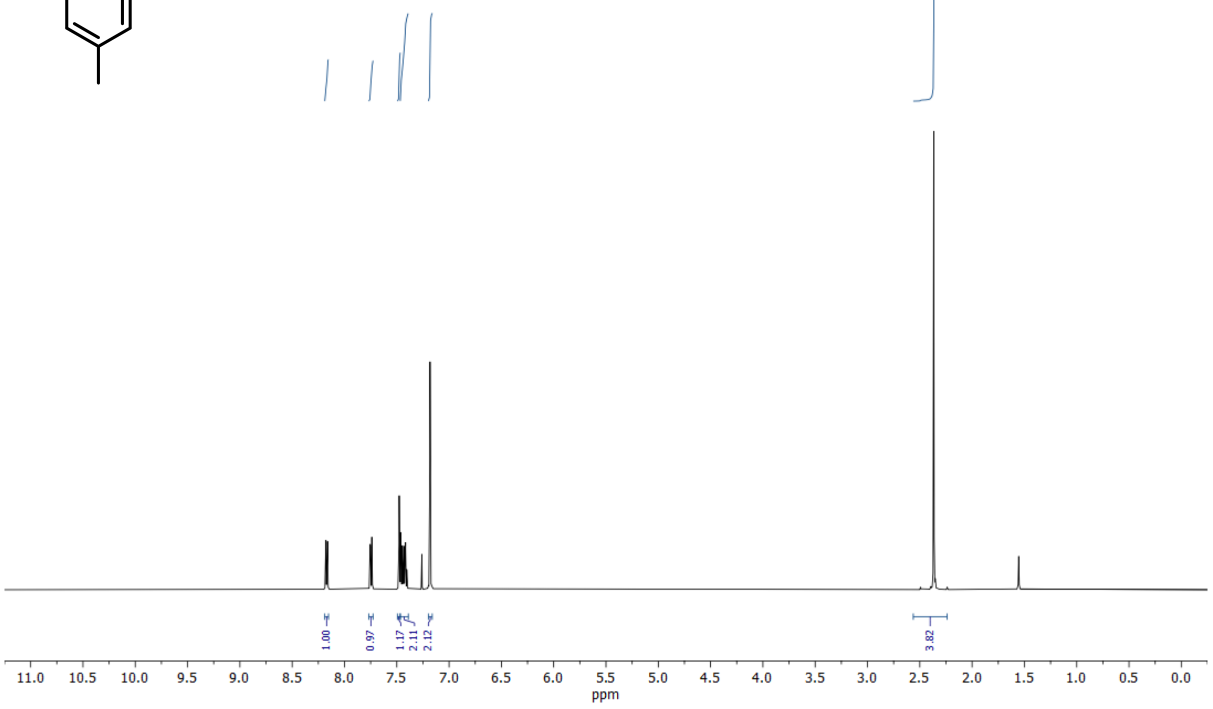

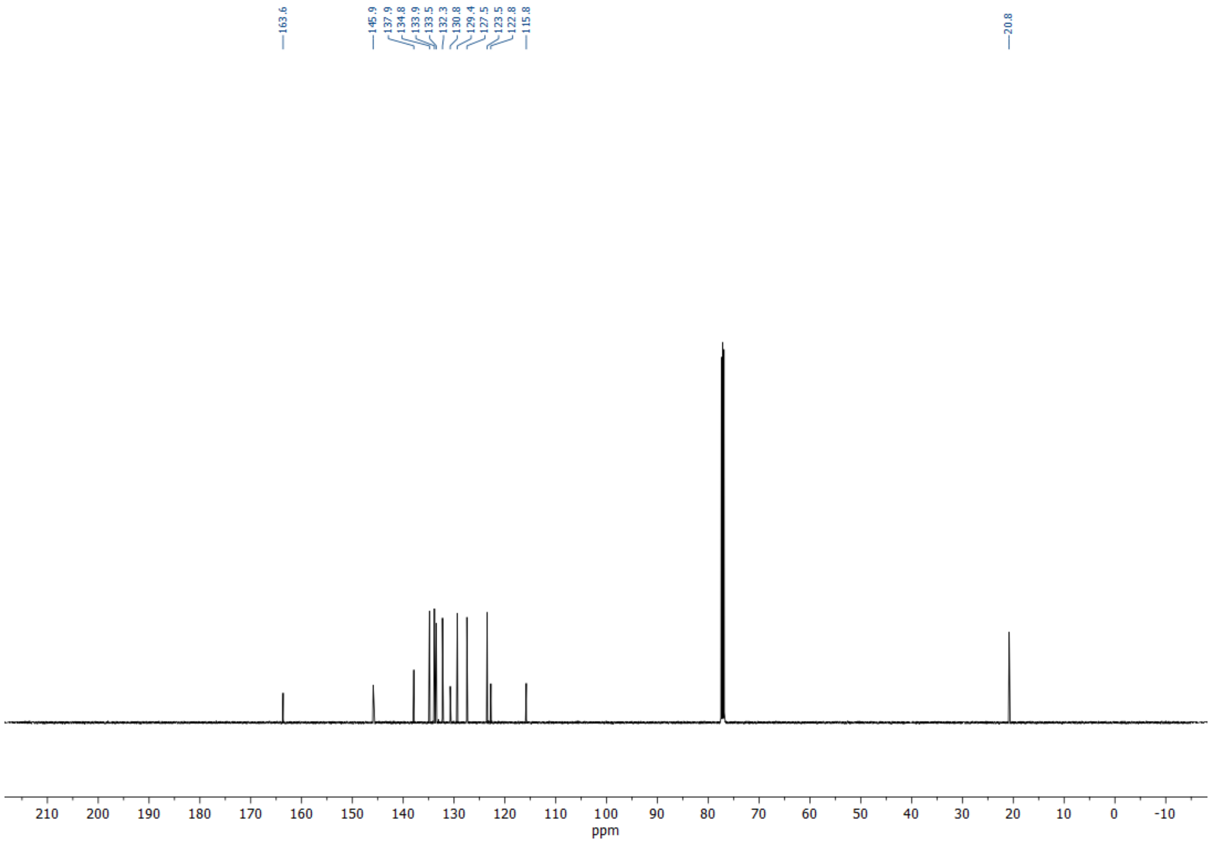

4-Bromo-2-methyl-9*H*-xanthene, S3:

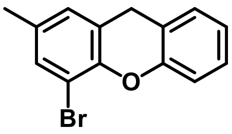

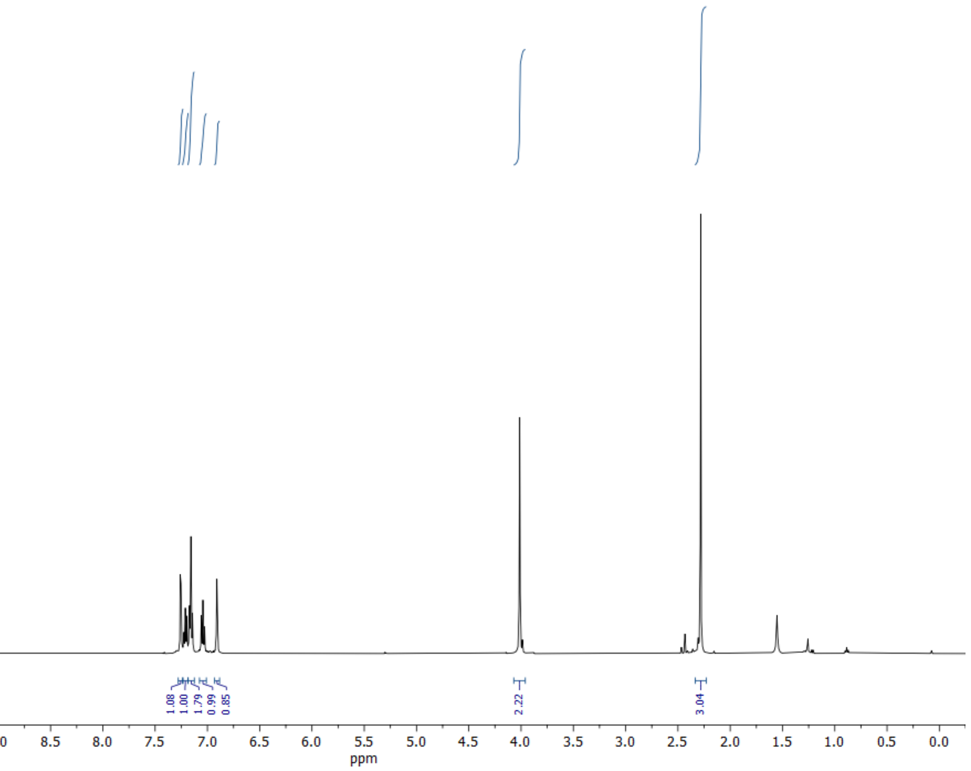

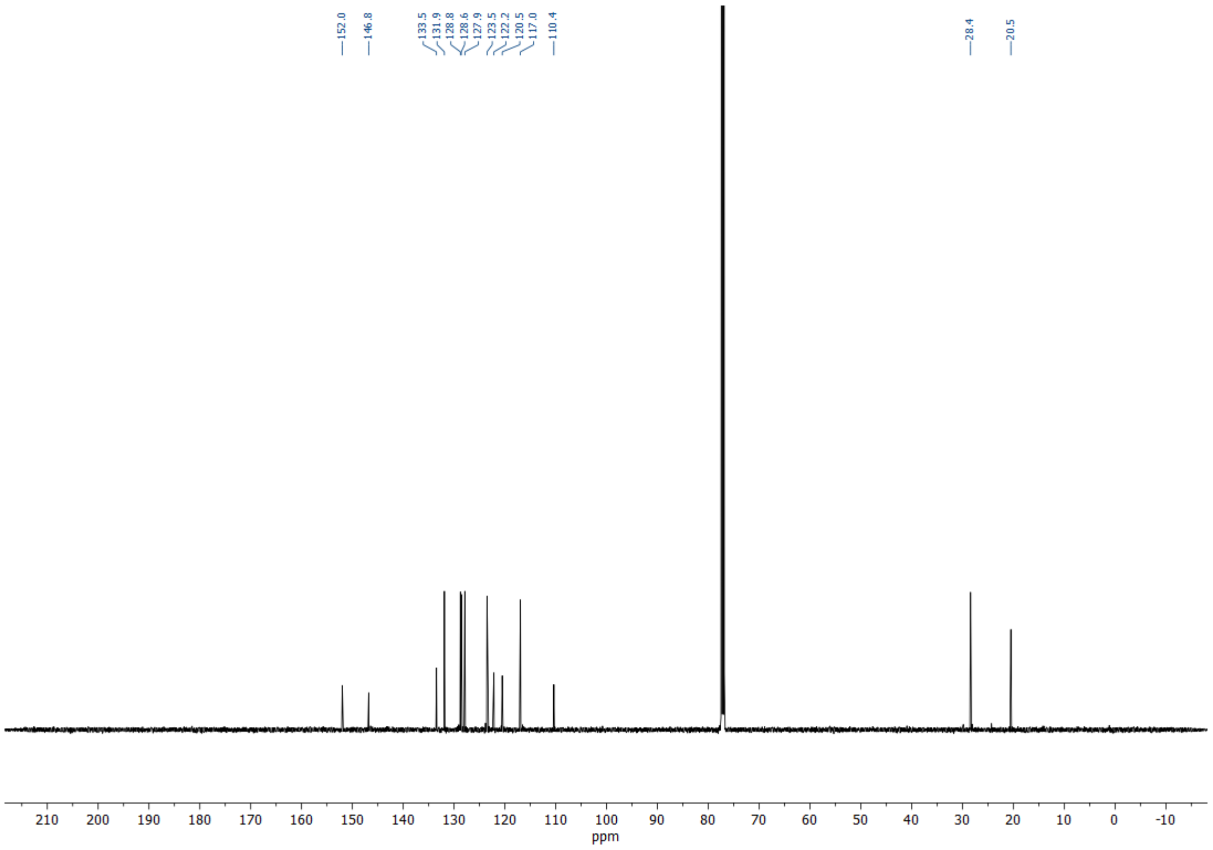

2,4-Dimethyl-6-(2-methyl-9*H*-xanthen-4-yl)aniline, 8:

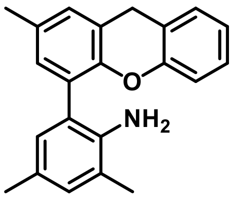

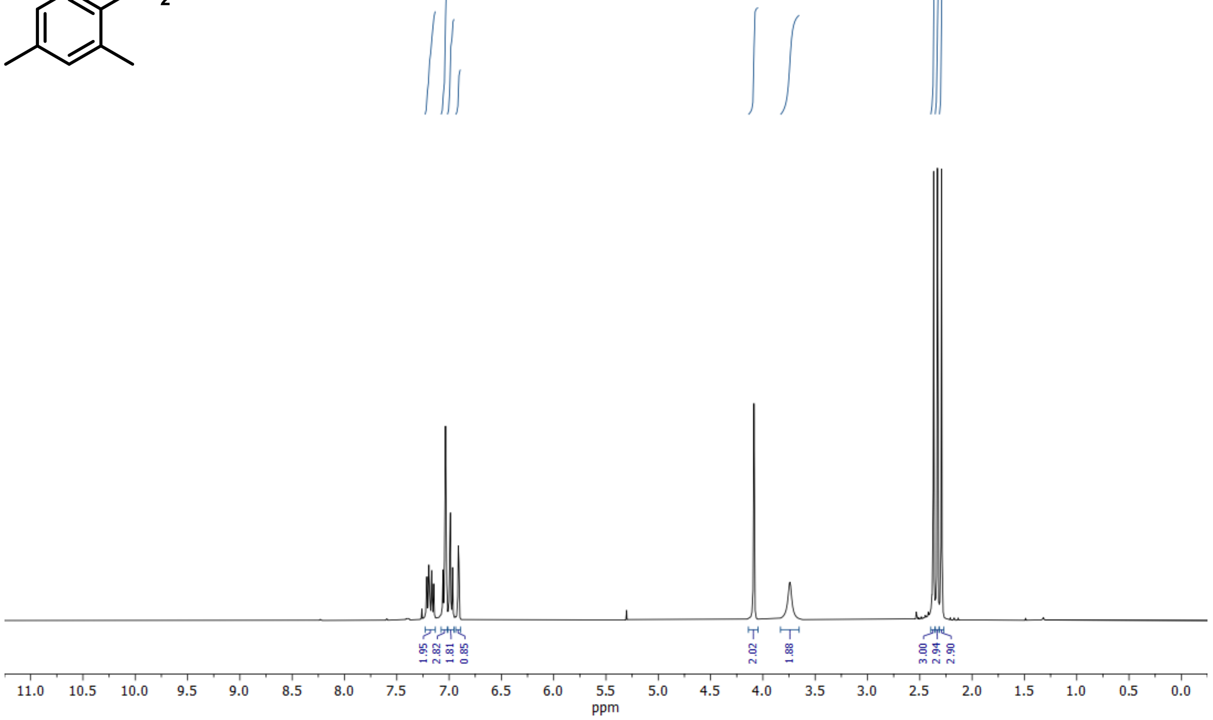

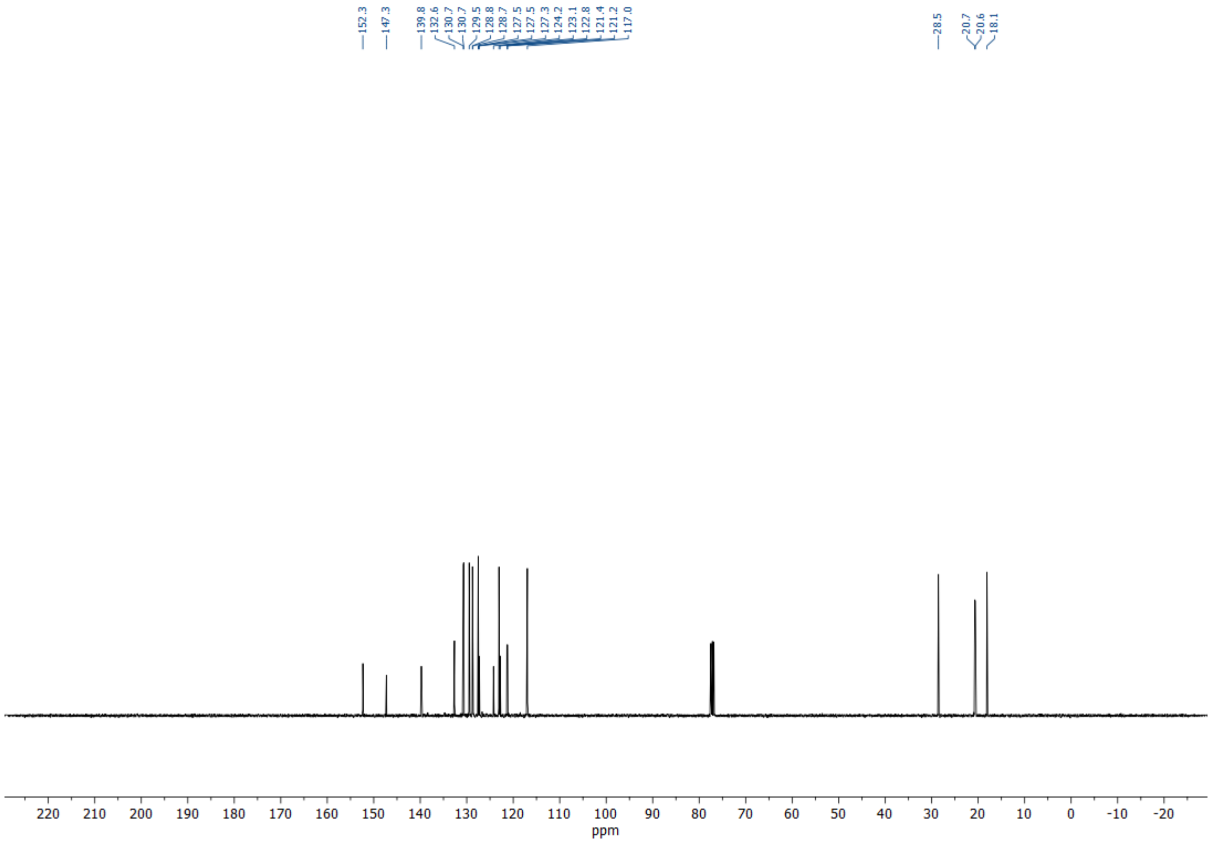

1,3,6-Trimethyl-8*H*-benzofuro[3,2,1-*de*]xanthen-13-ium tetrafluoroborate, 10:

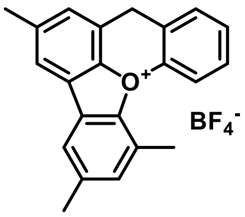

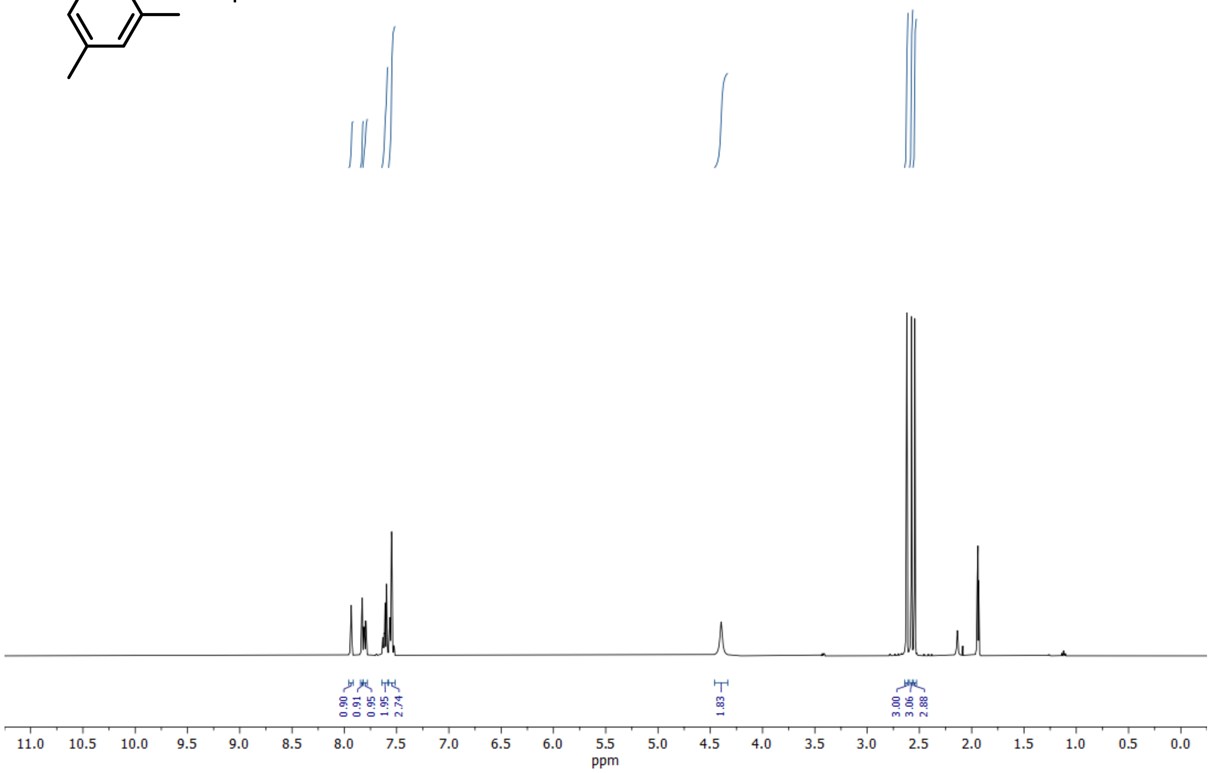

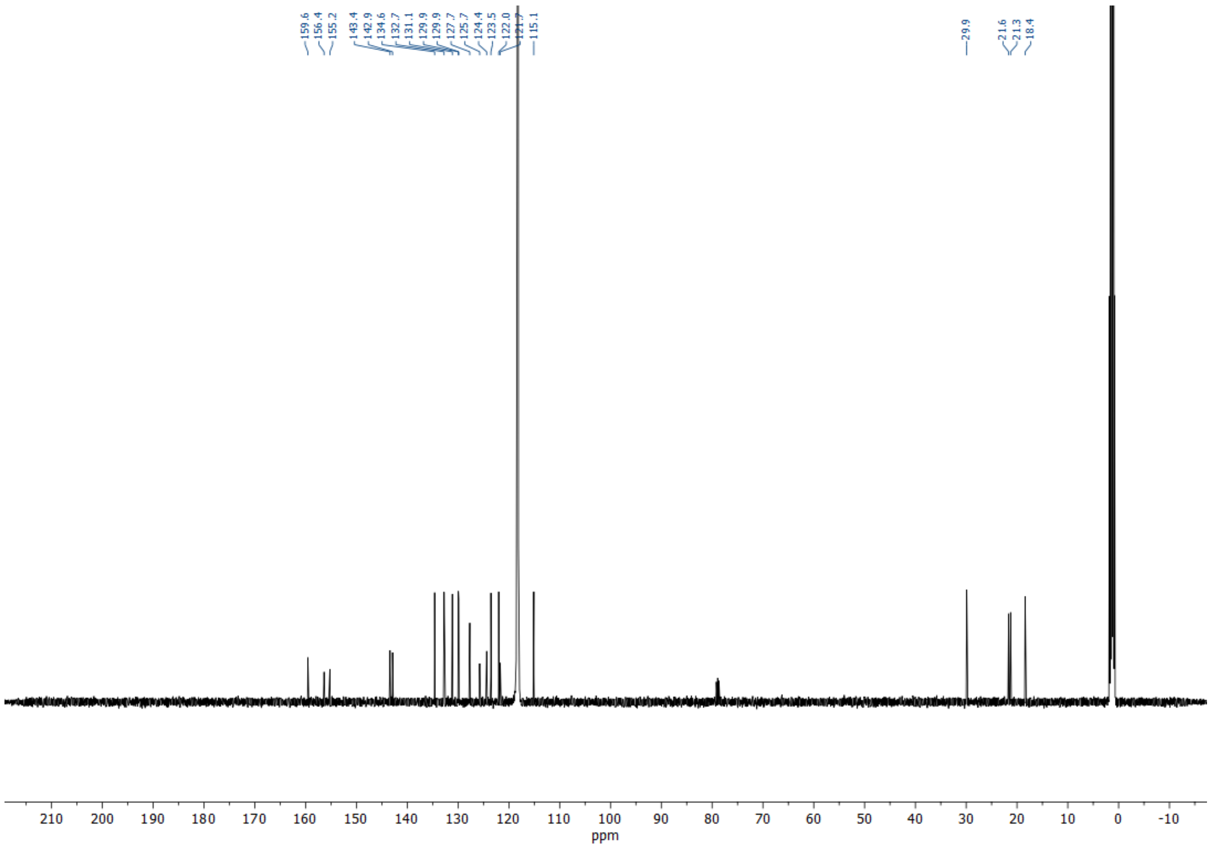

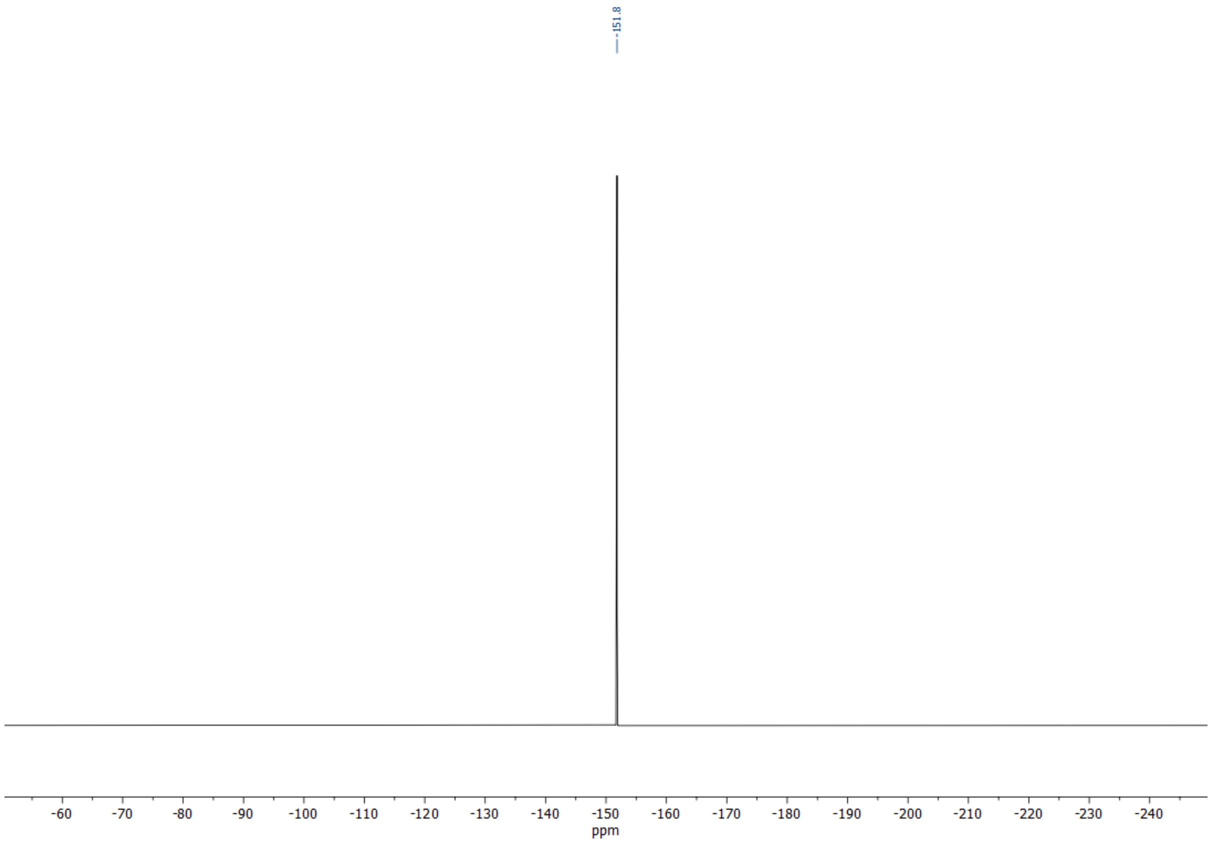

-151.8
-60 -70 -80 -90 -100 -110 -120 -130 -140 -150 -160 -170 -180 -190 -200 -210 -220 -230 -240
ppm

2-Bromo-4-methylphenyl 2,3-dichlorobenzoate, S4:

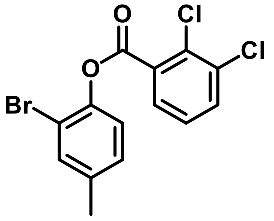

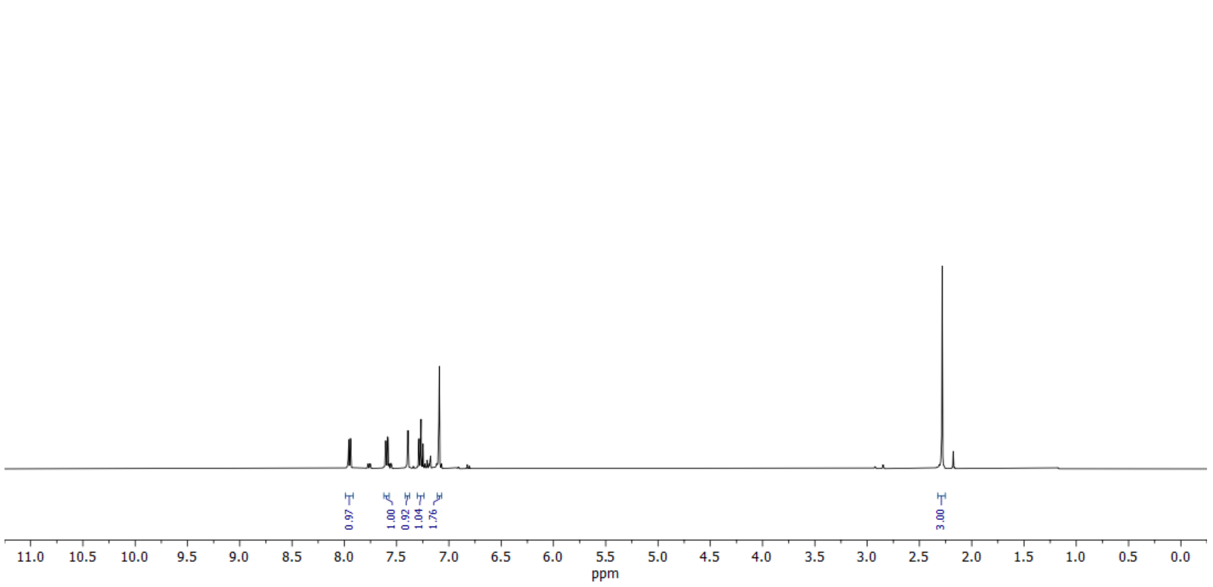

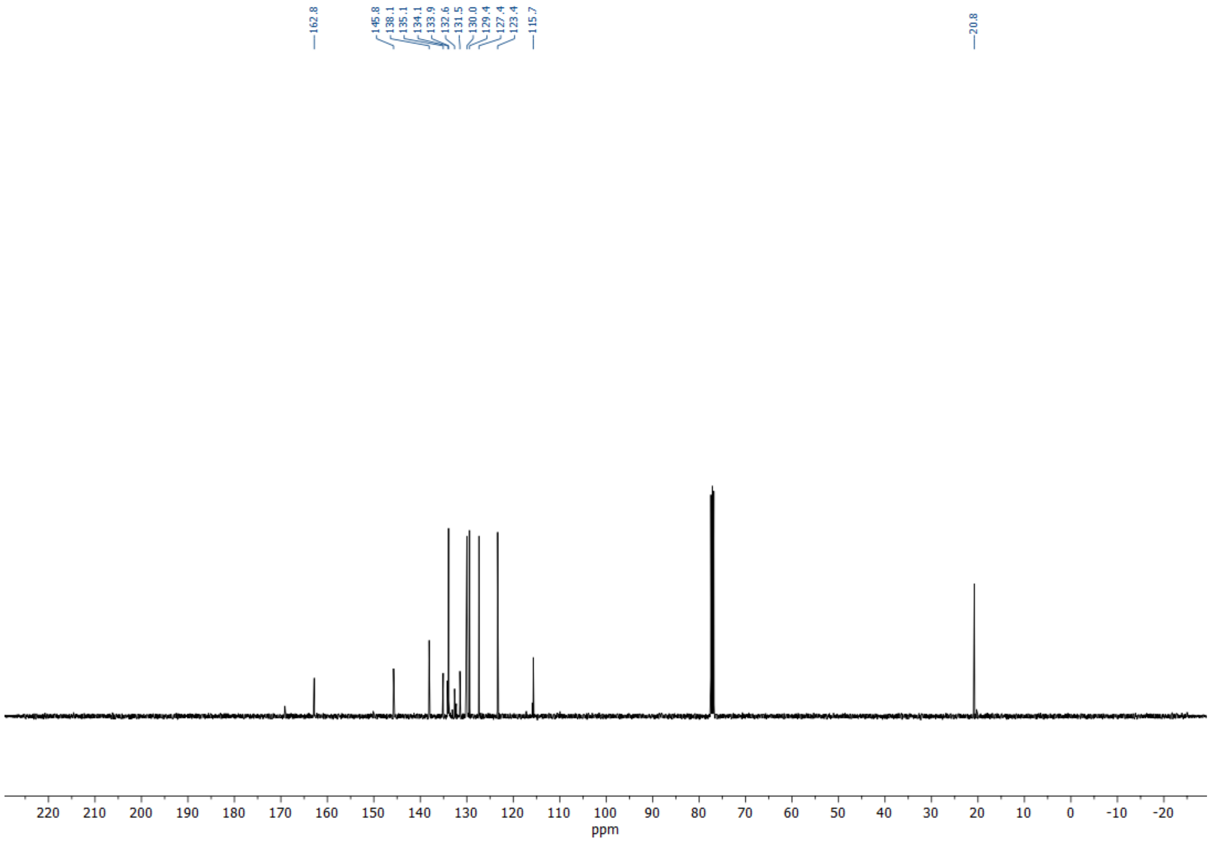

4-Bromo-5-chloro-2-methyl-9*H*-xanthene, S5:

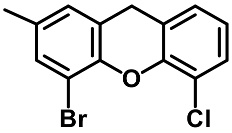

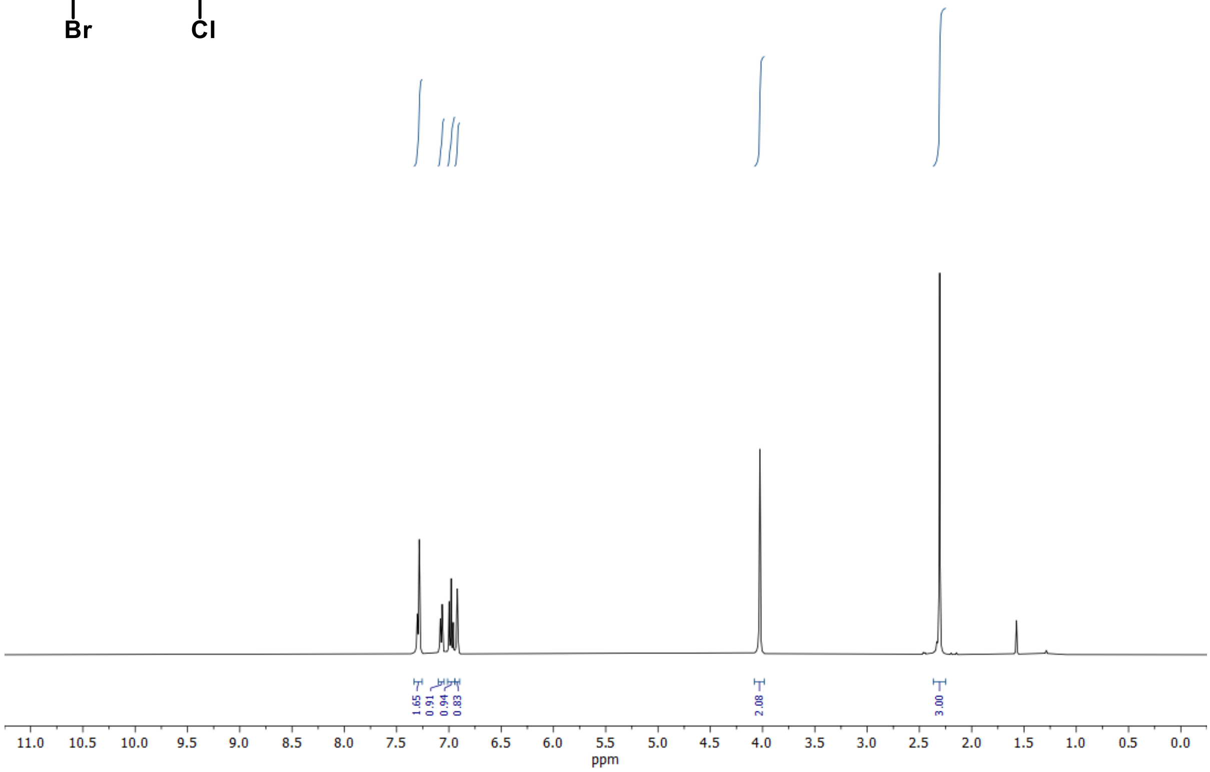

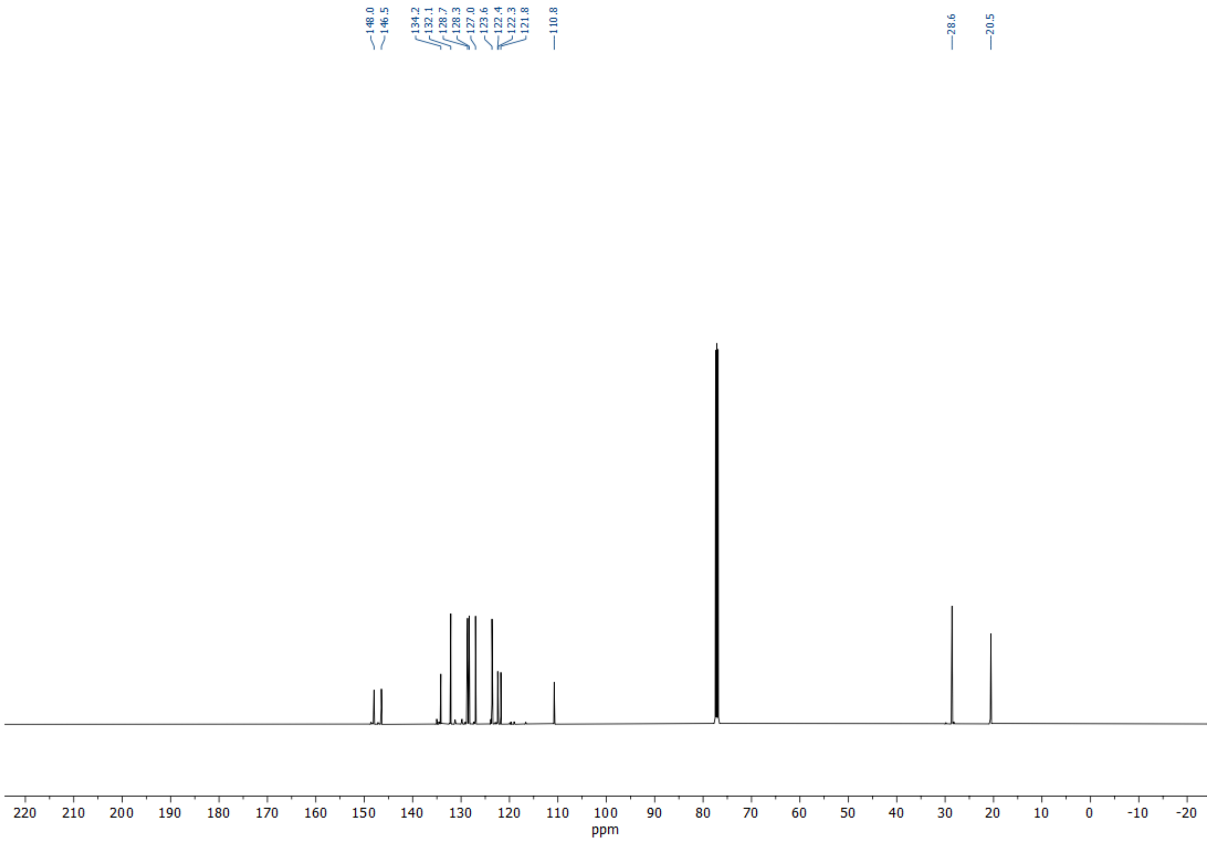

2-(5-Chloro-2-methyl-9*H*-xanthen-4-yl)-4,6-dimethylaniline, 9:

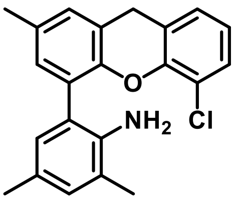

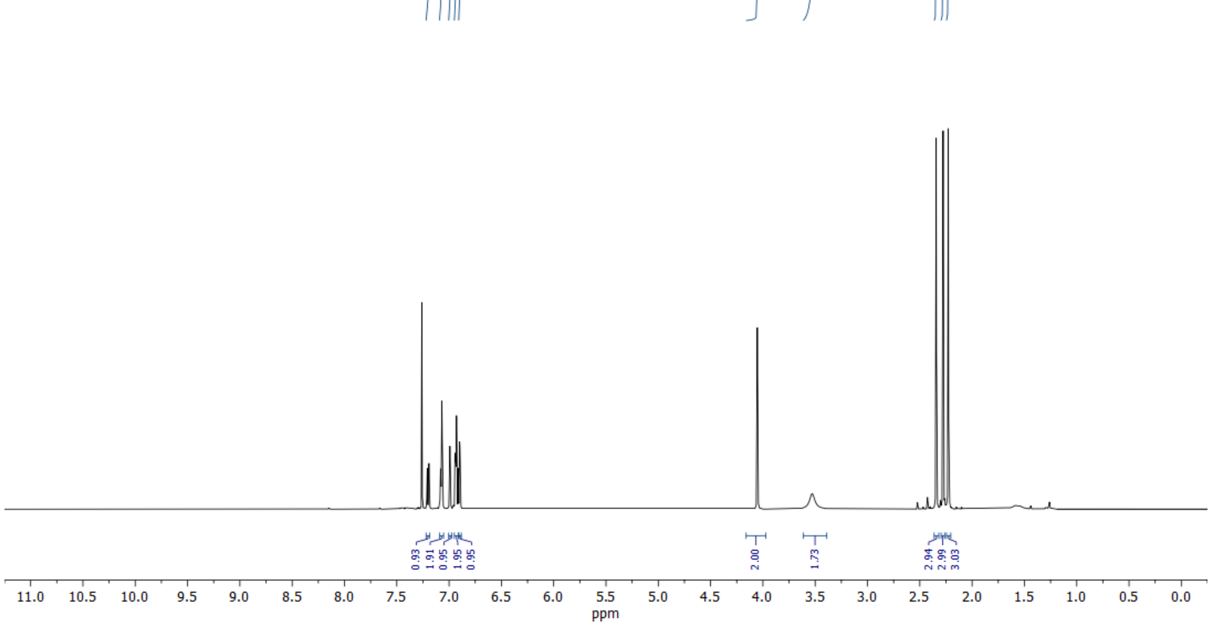

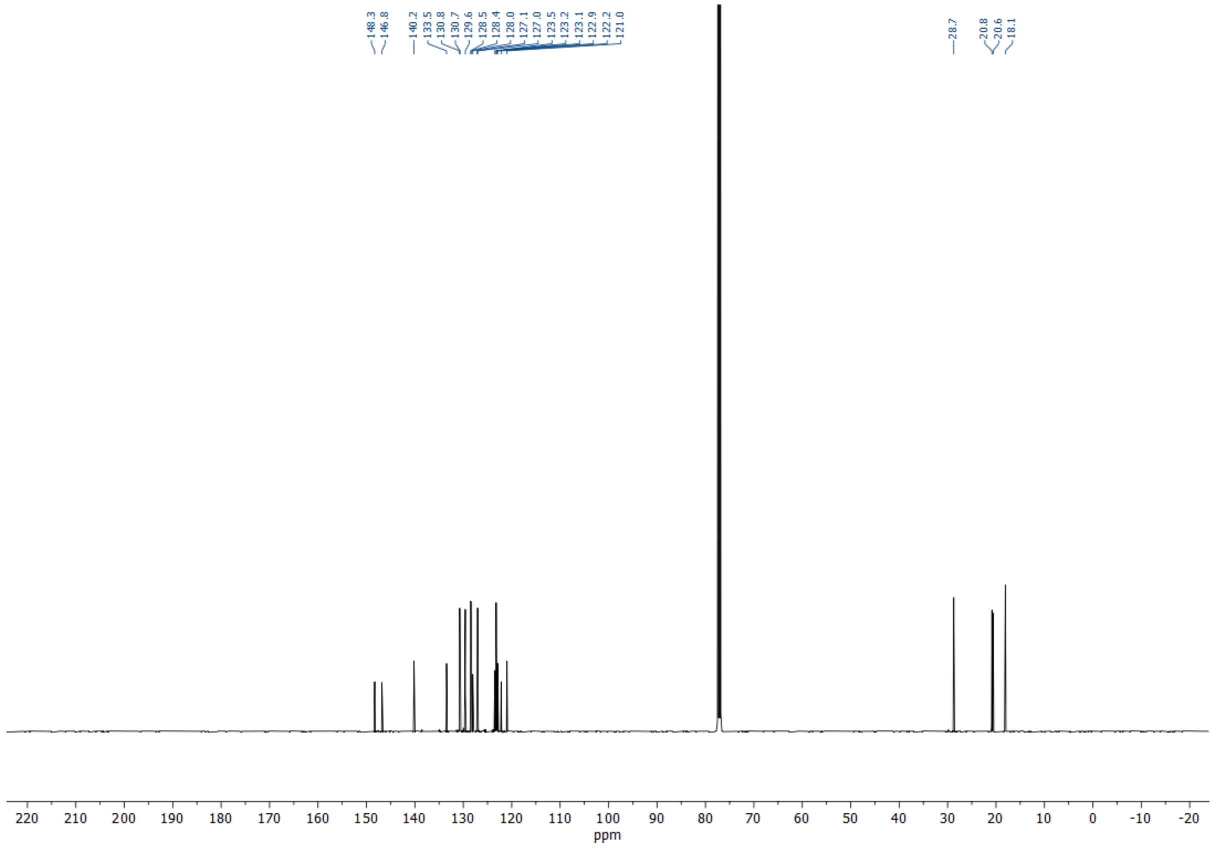

12-Chloro-1,3,6-trimethyl-8*H*-benzofuro[3,2,1-*de*]xanthen-13-ium tetrakis(perfluorophenyl)borate, 12:

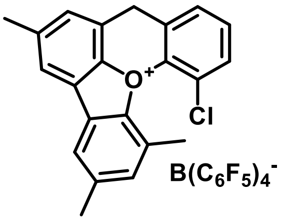

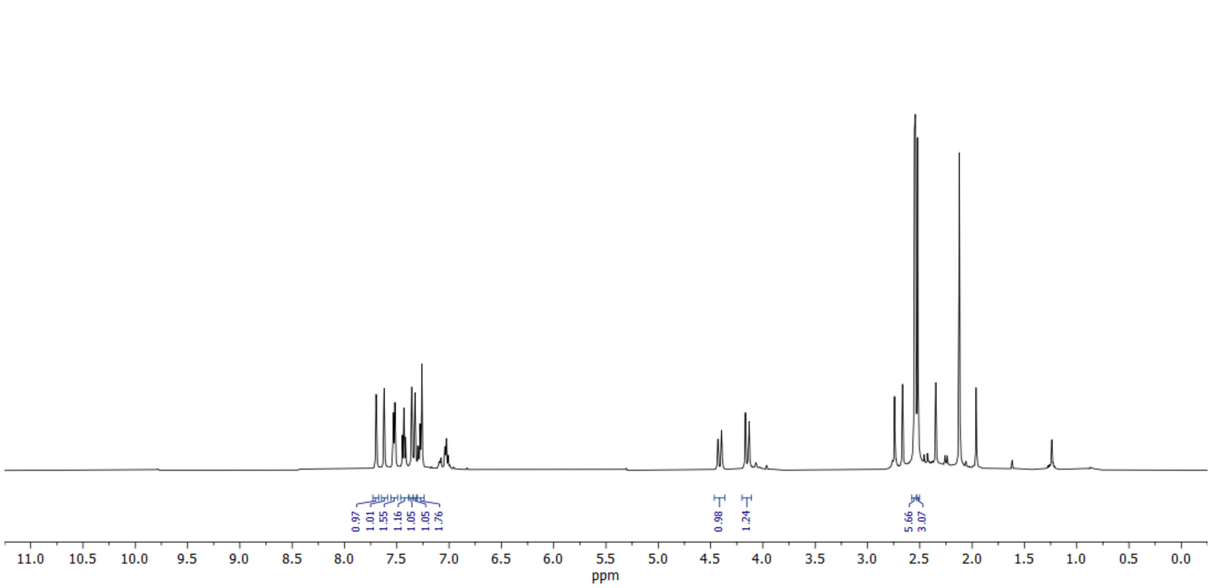

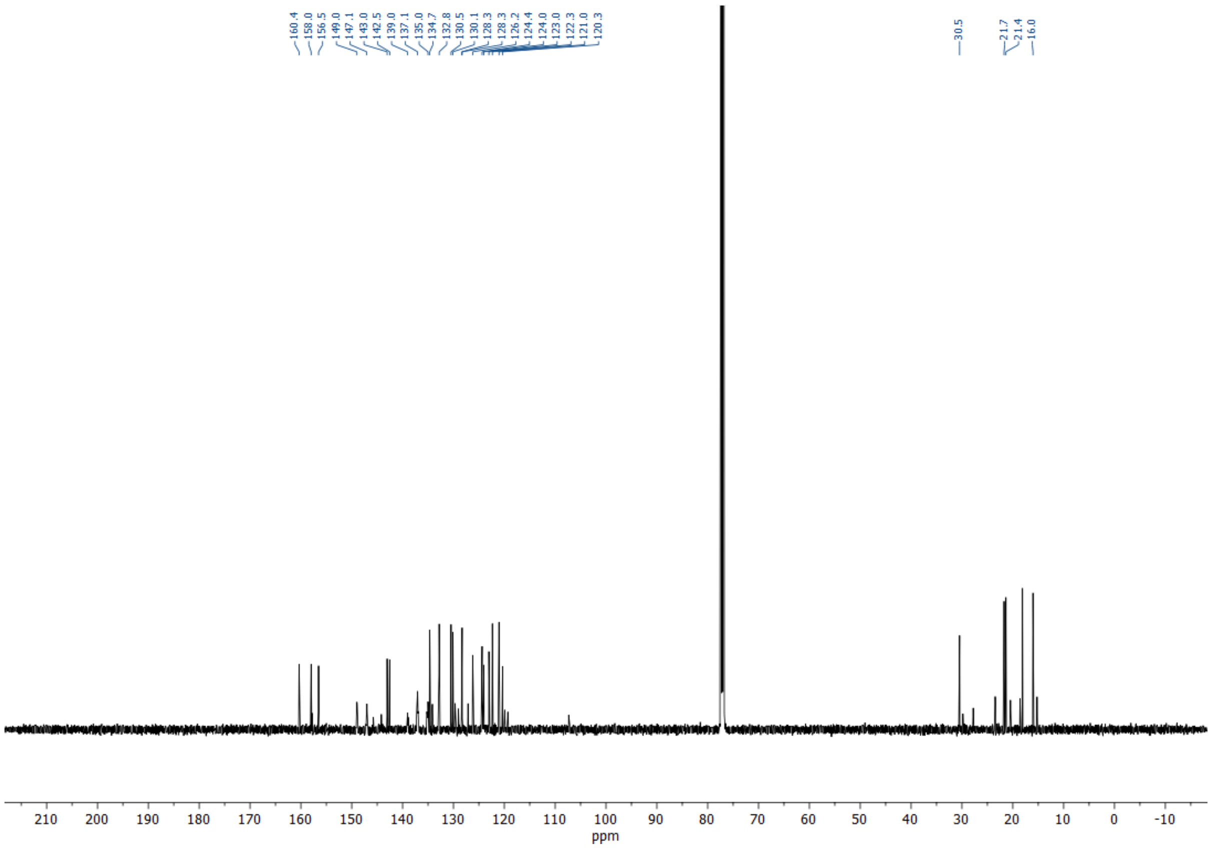

3-bromo-2-fluorobenzaldehyde, S6:

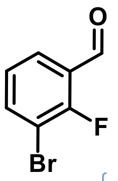

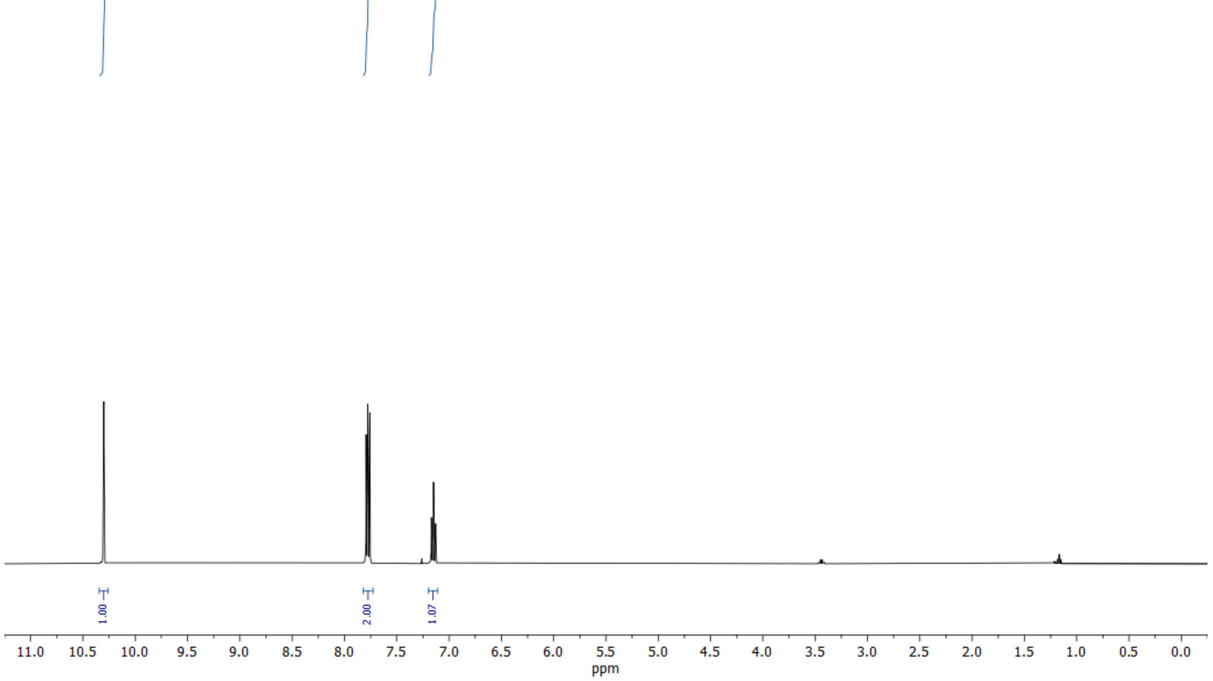

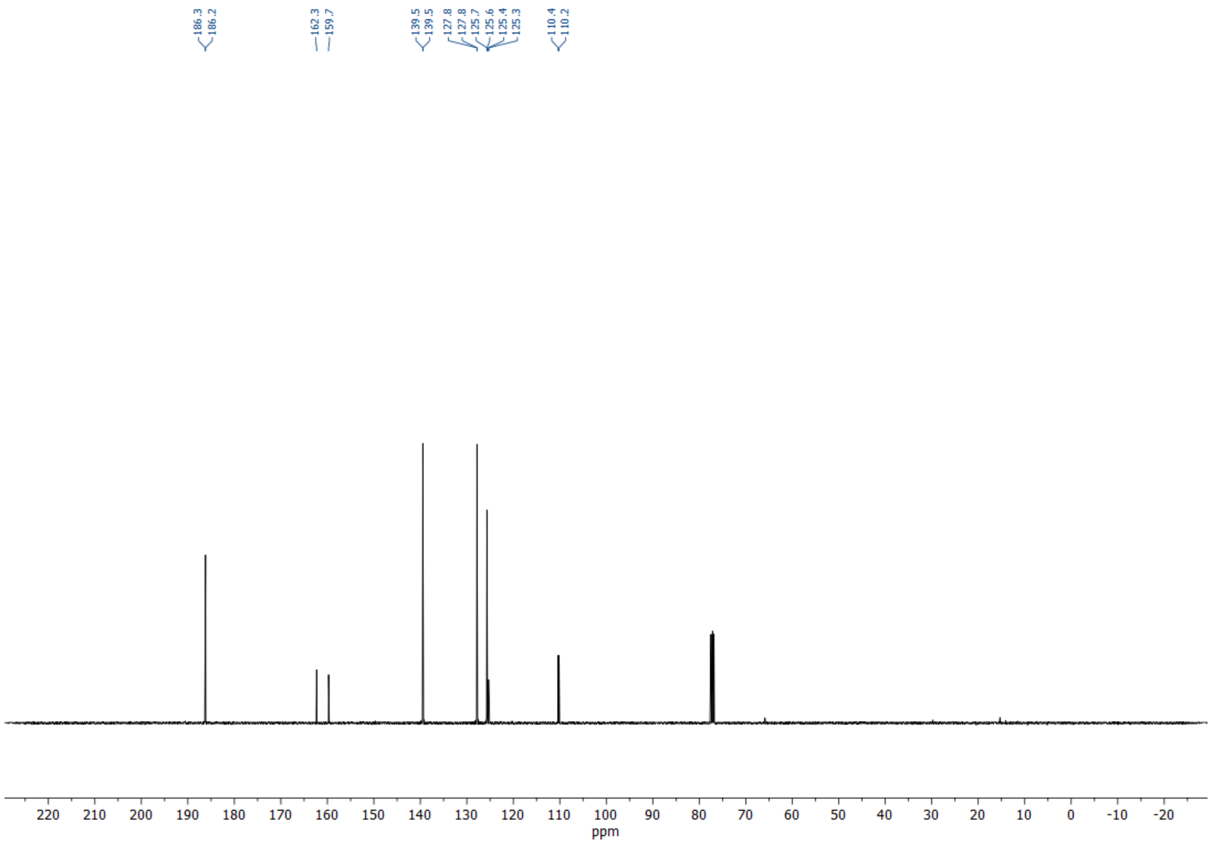

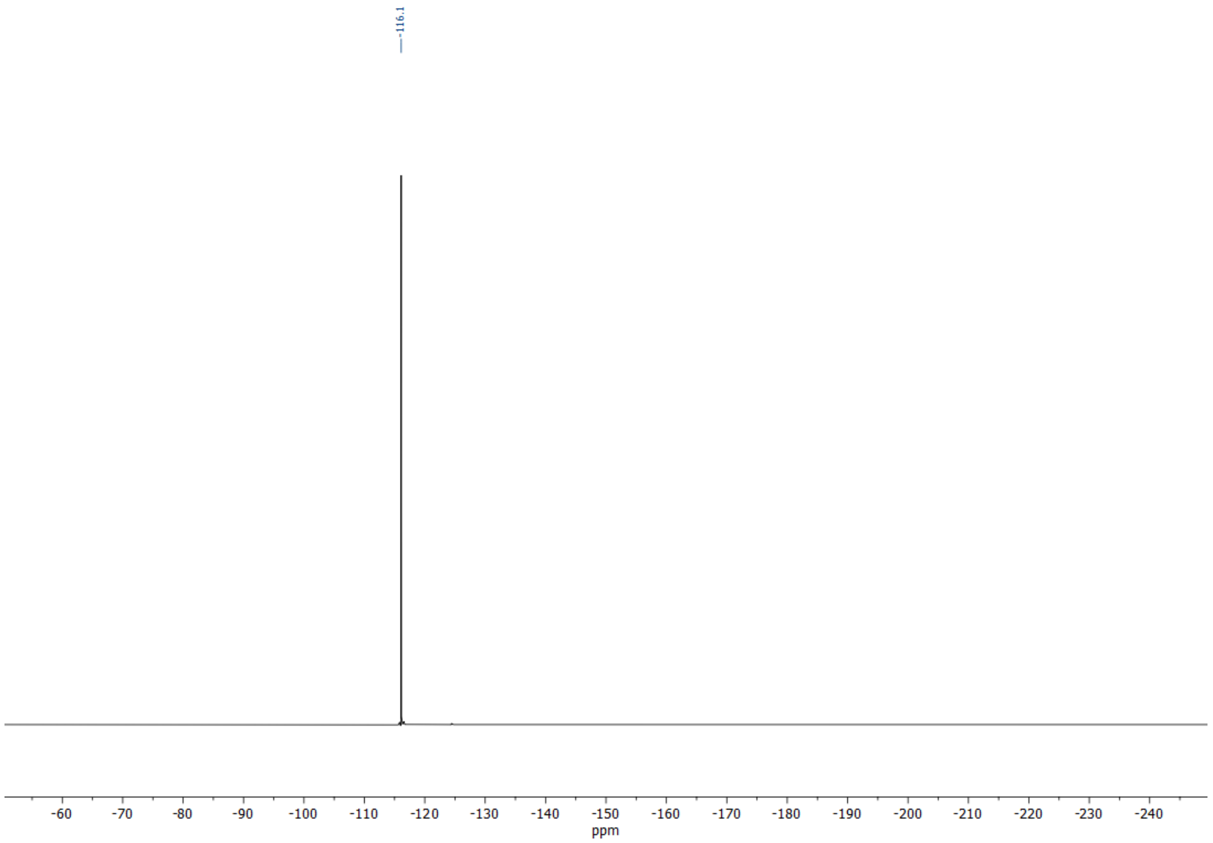
-116.1
-60
-70
-80
-90
-100
-110
-120
-130
-140
-150
-160
-170
-180
-190
-200
-210
-220
-230
-240
ppm

3-Bromo-2-(2-formylphenoxy)benzaldehyde, S7:

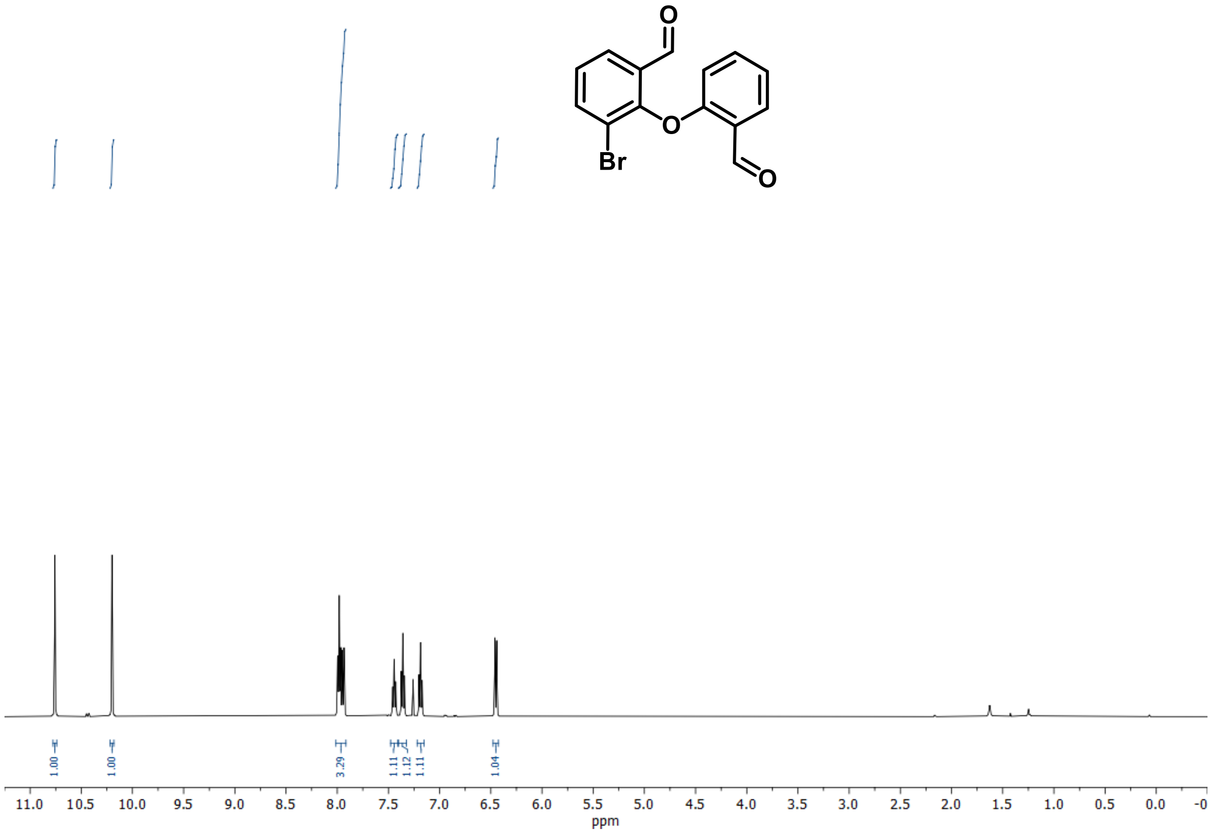

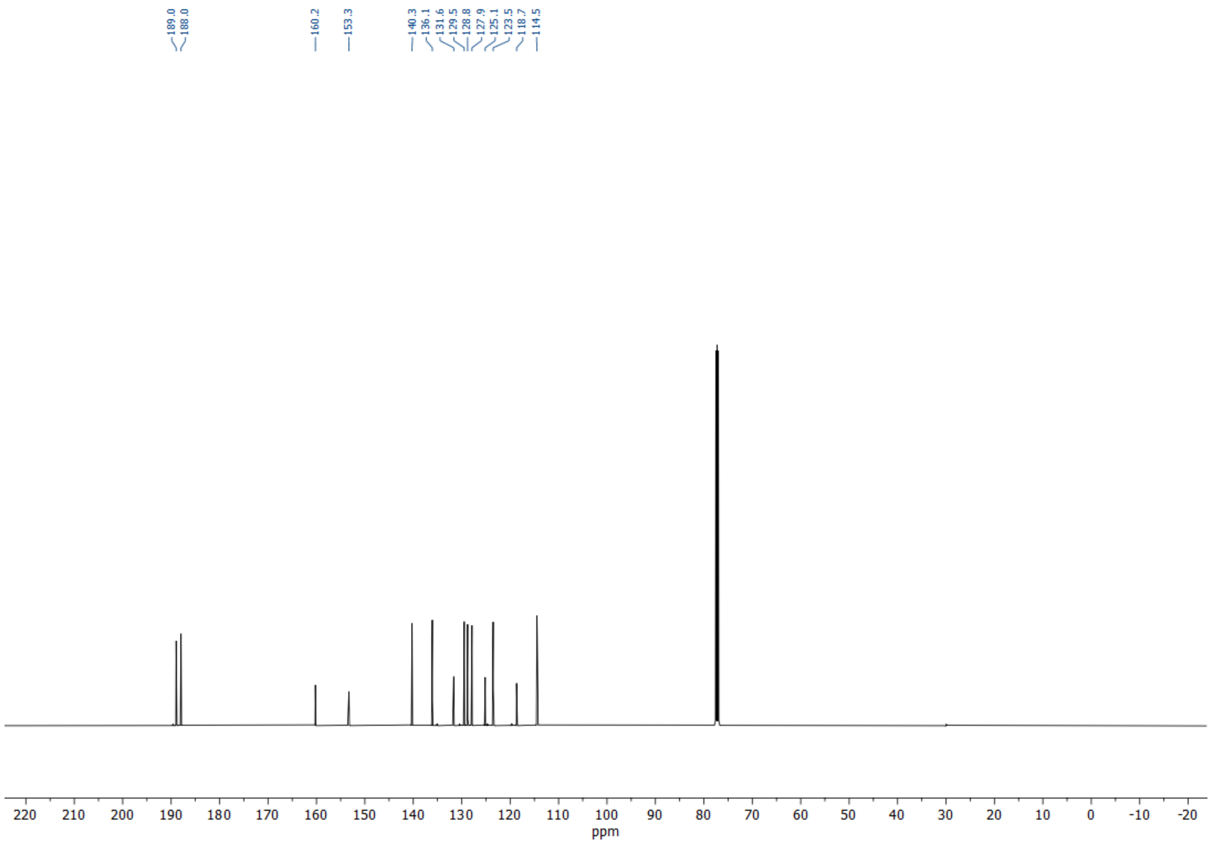

1-Bromo-3-vinyl-2-(2-vinylphenoxy)benzene, S8:

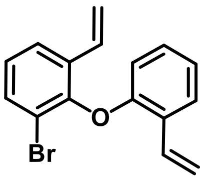

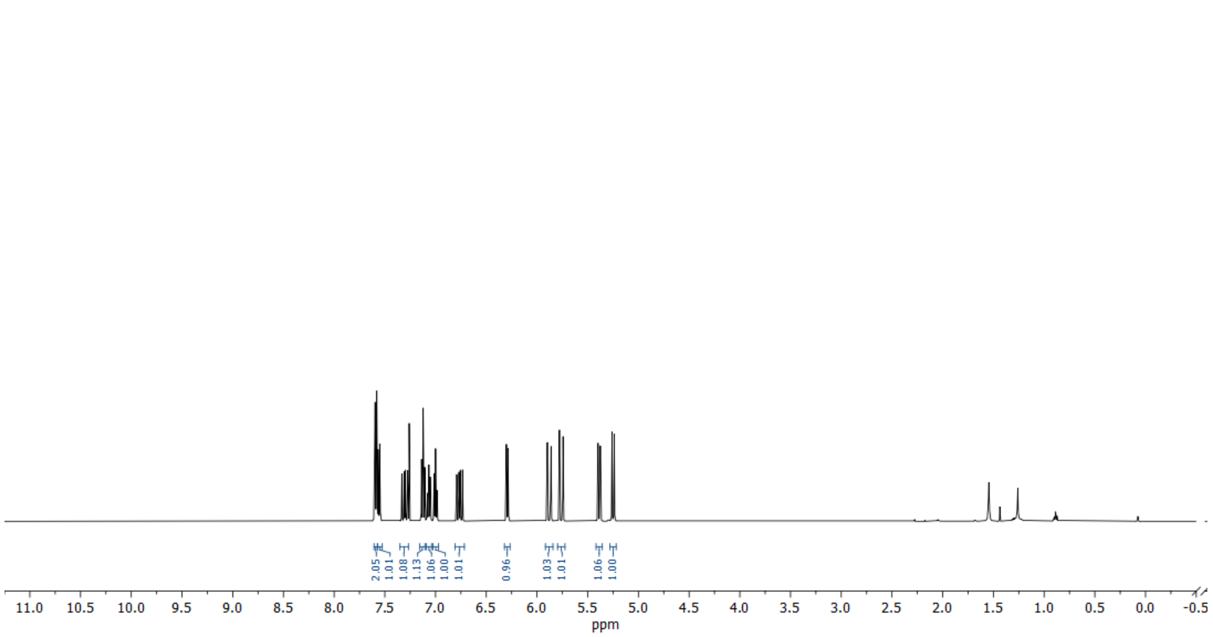

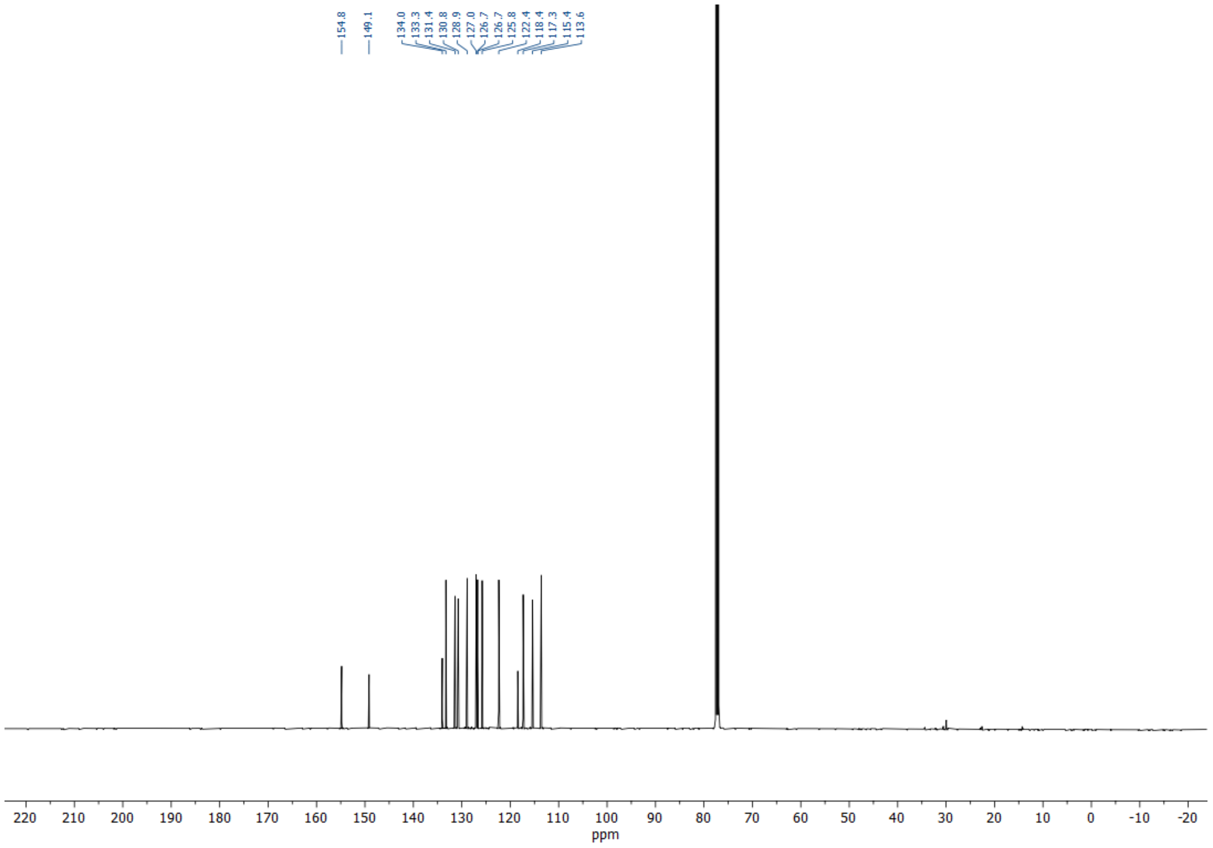

4-Bromodibenzo[*b*,*f*]oxepine, 13:

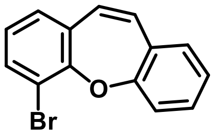

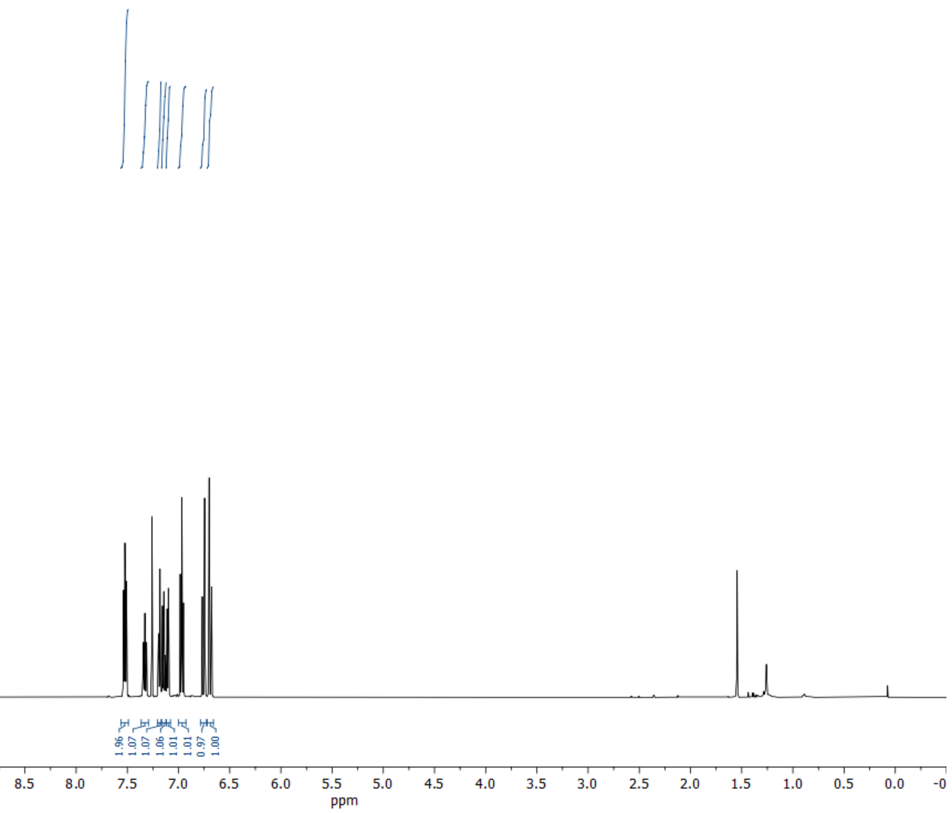

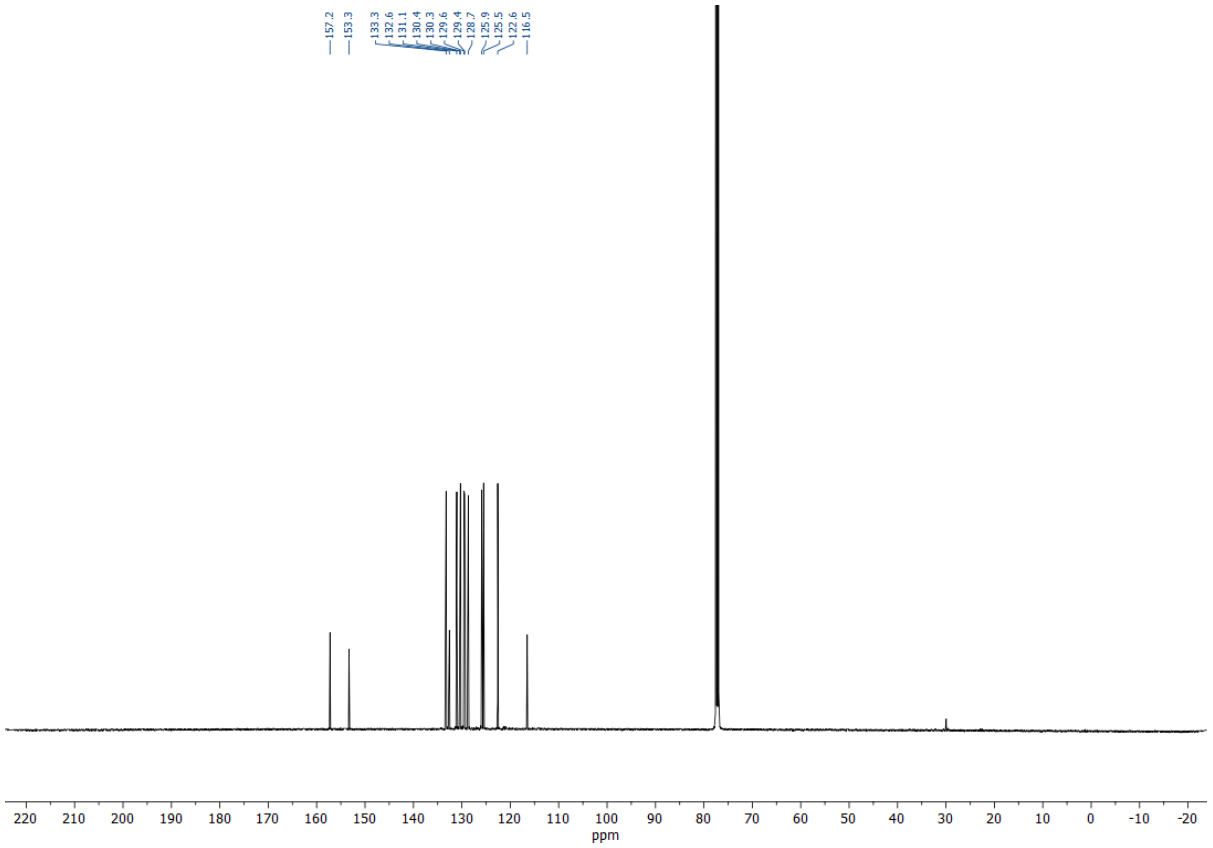

2-(Dibenzo[*b*,*f*]oxepin-4-yl)-4,6-dimethylaniline, S9:

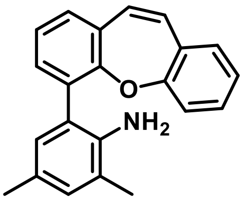

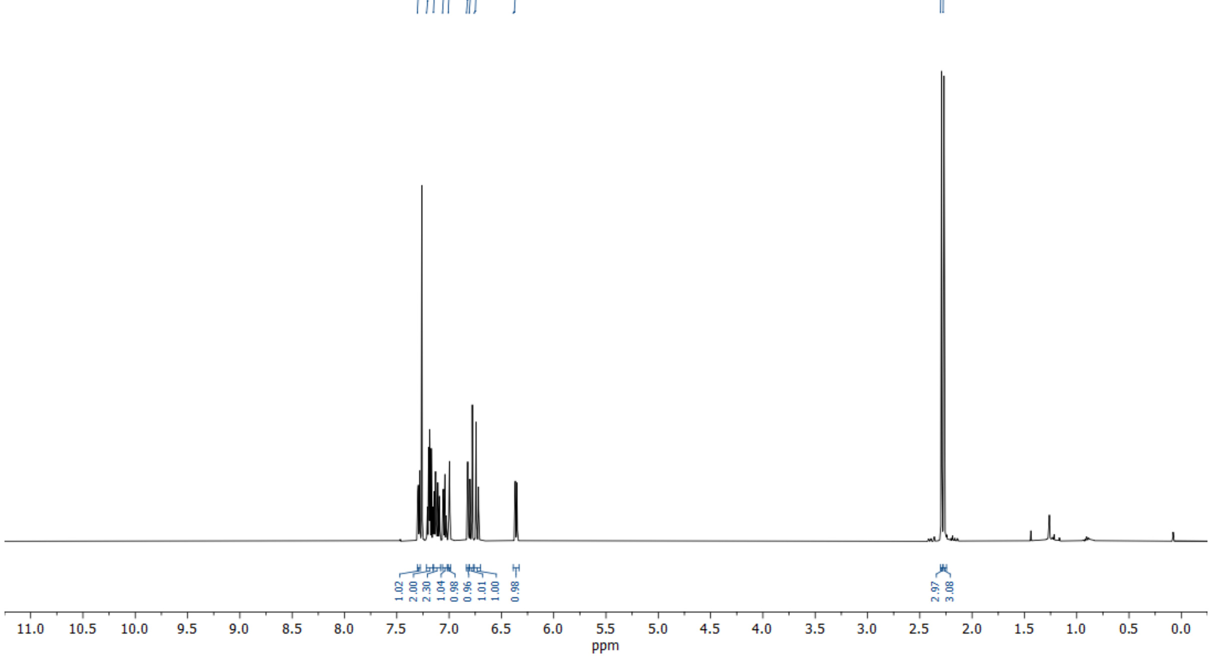

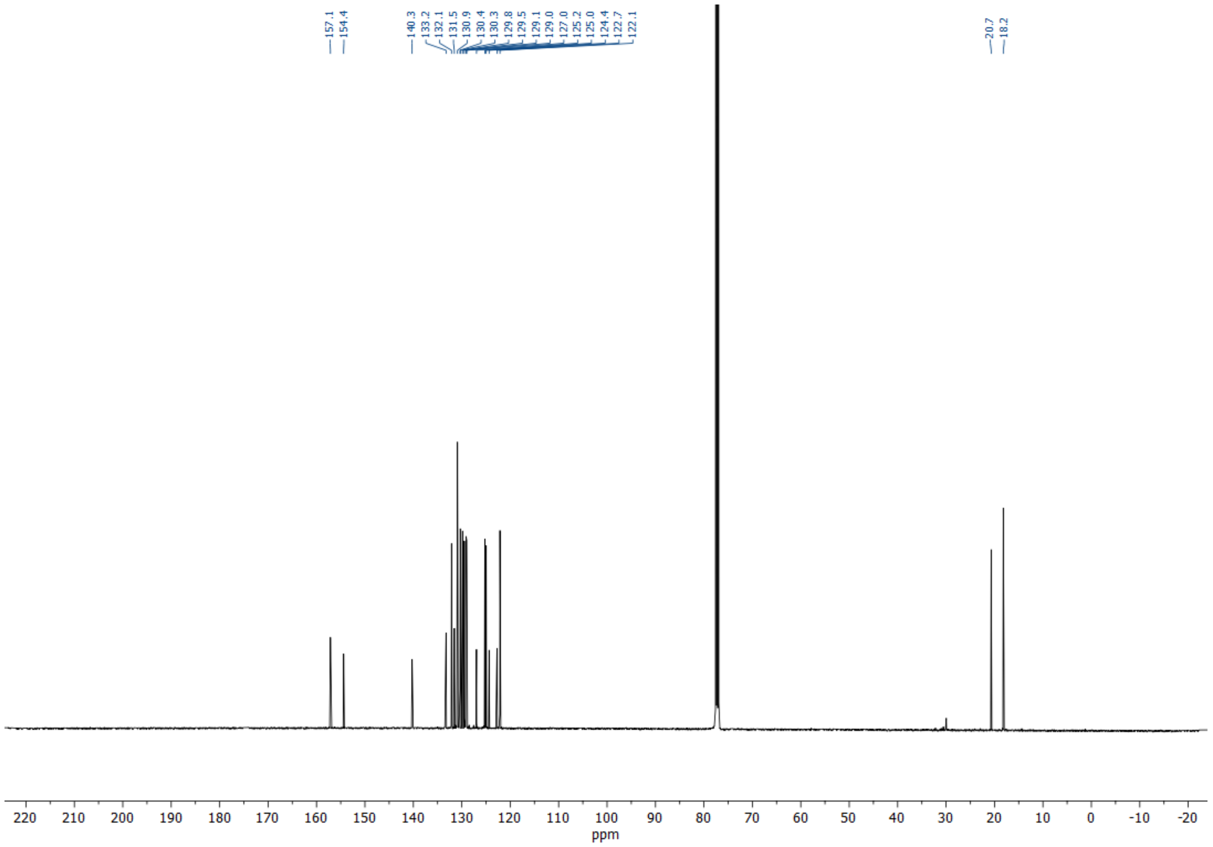

2-(10,11-Dihydrodibenzo[*b*,*f*]oxepin-4-yl)-4,6-dimethylaniline, 15:

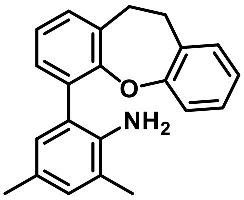

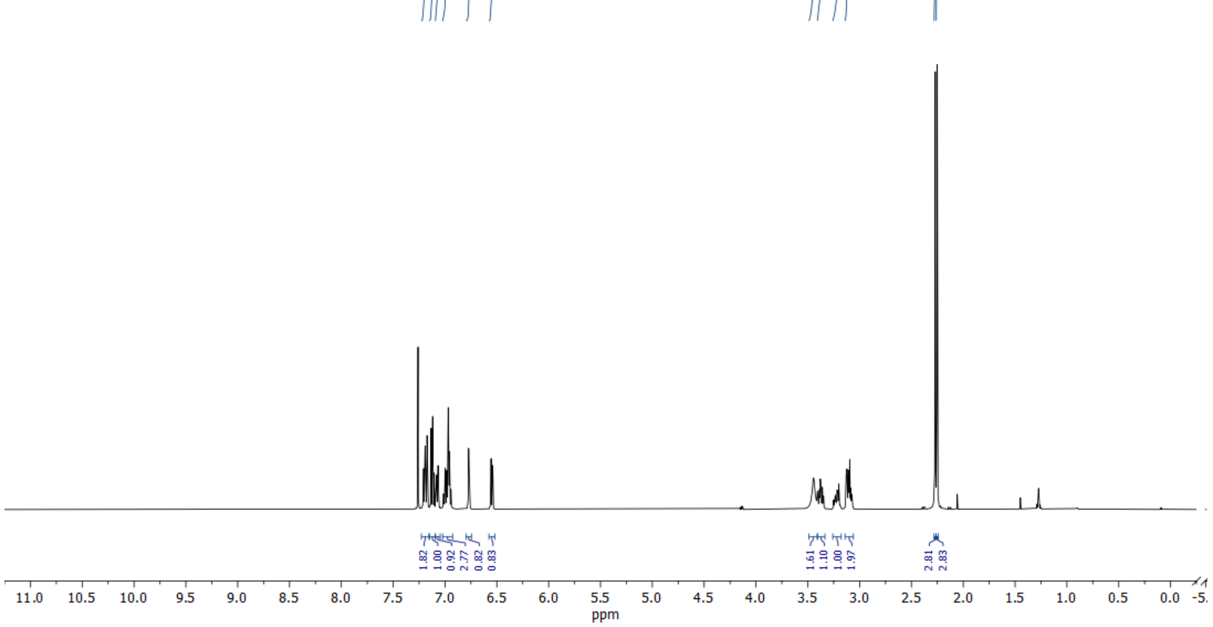

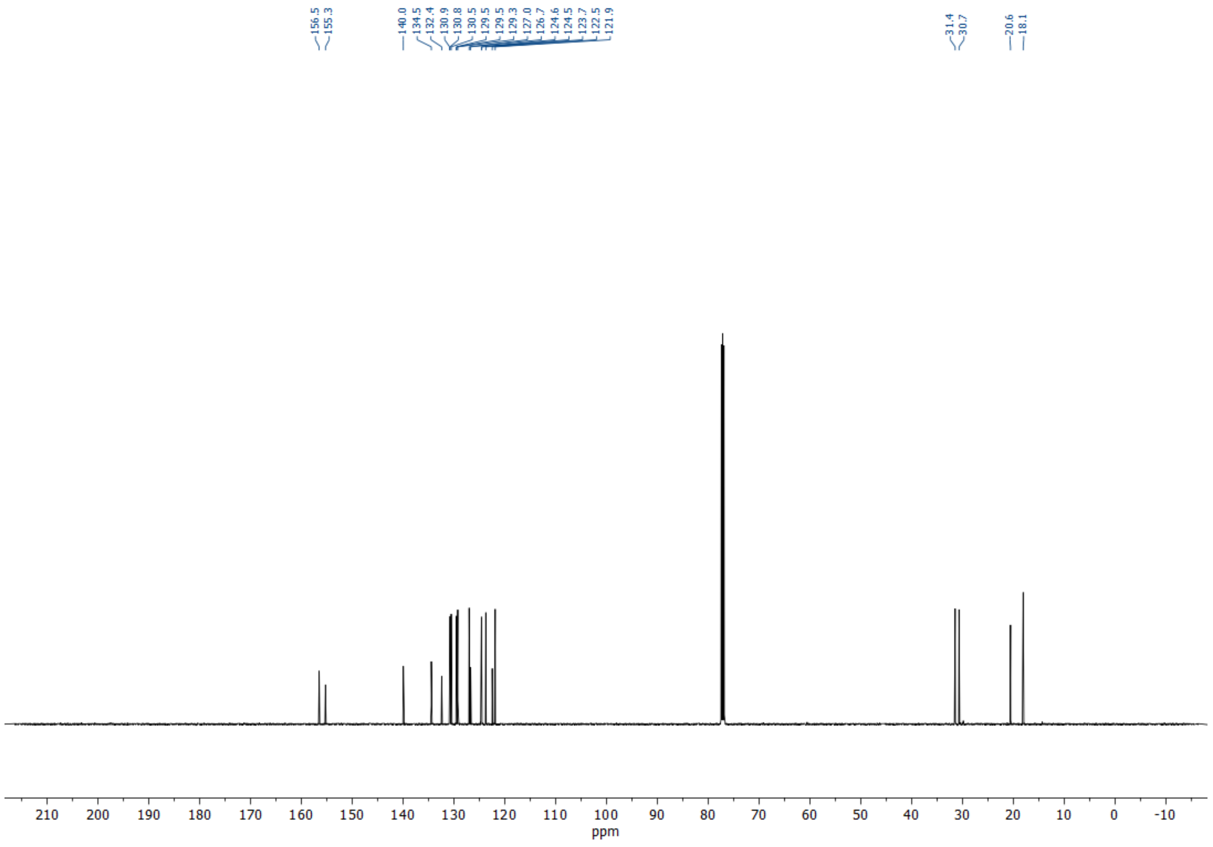

11,13-Dimethyl-4,5-dihydrobenzo[*b*]benzo[6,7]oxepino[3,2,1-*hi*]benzofuran-10-ium tetrafluoroborate, *rac*-16:

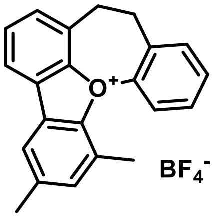

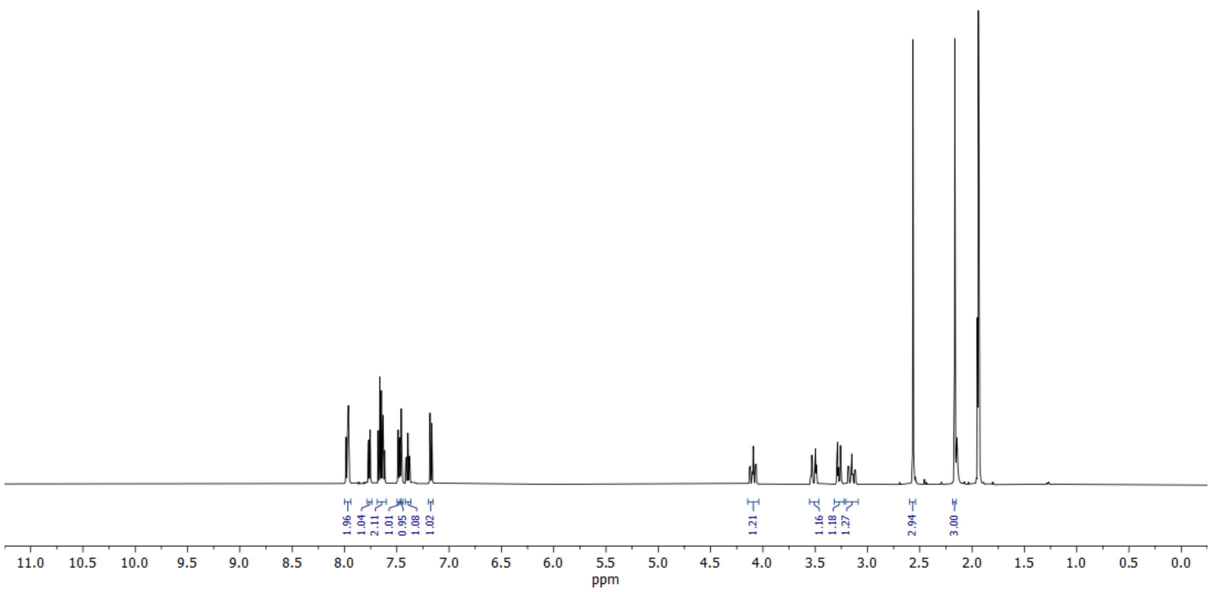

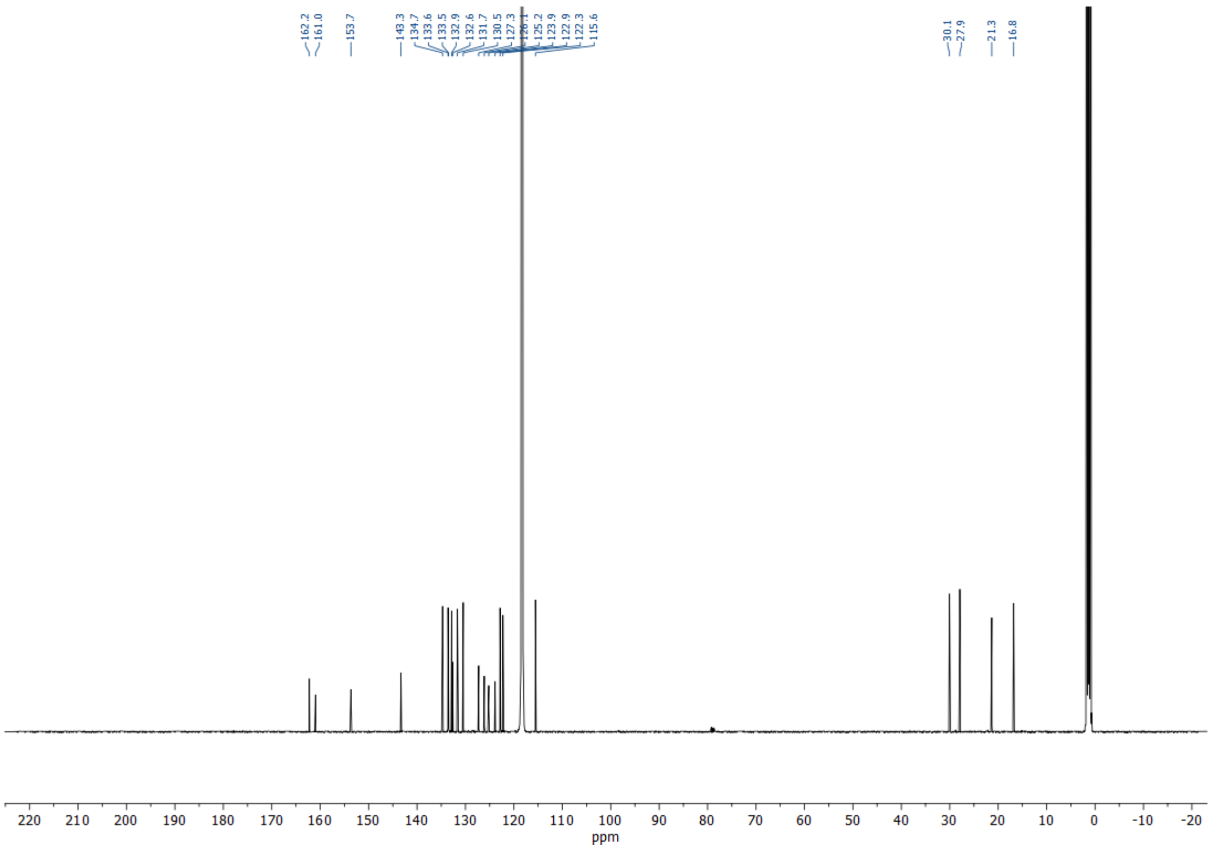

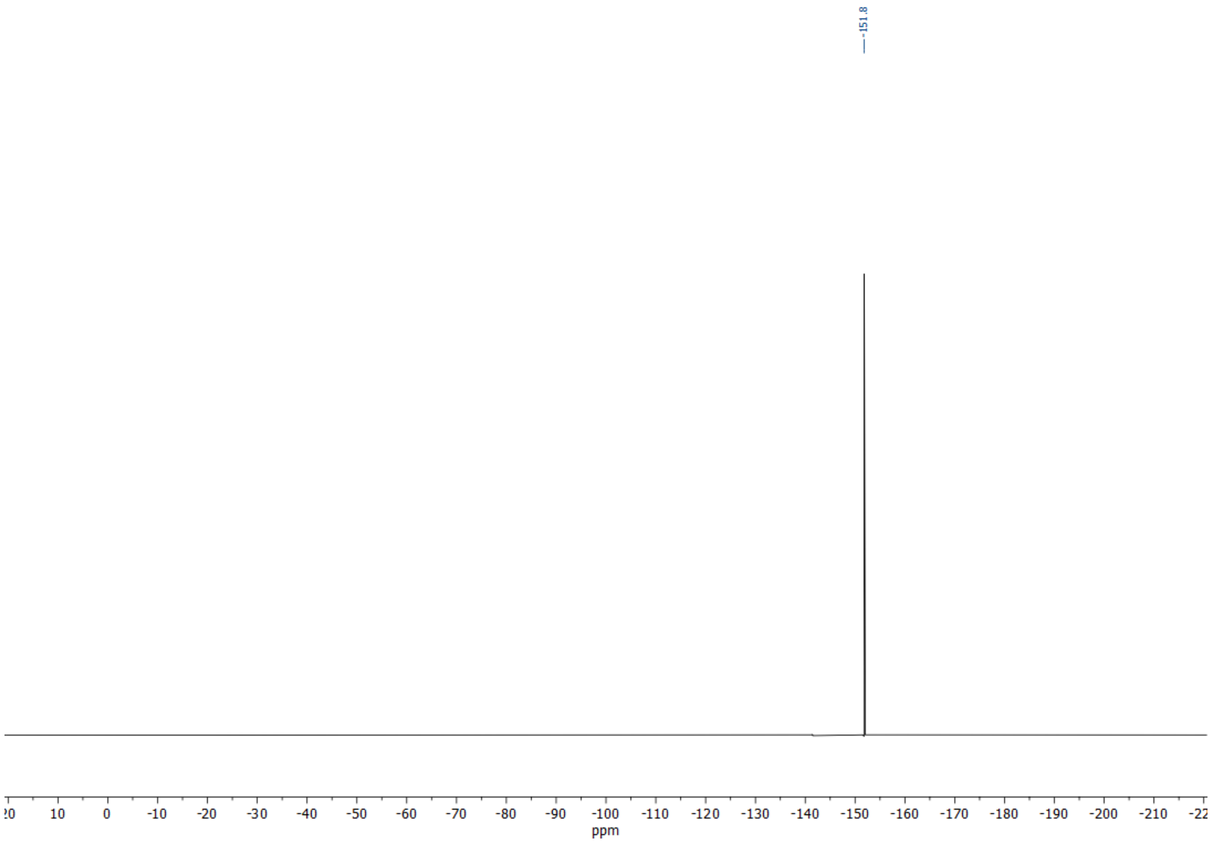
-151.8
20 10 0 -10 -20 -30 -40 -50 -60 -70 -80 -90 -100 -110 -120 -130 -140 -150 -160 -170 -180 -190 -200 -210 -22
ppm

11,13-Dimethyl-4,5-dihydrobenzo[*b*]benzo[6,7]oxepino[3,2,1-*hi*]benzofuran-10-ium hexafluorophosphate, *rac*-17:

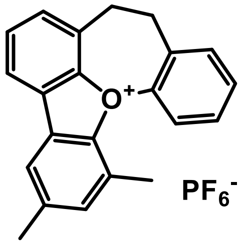

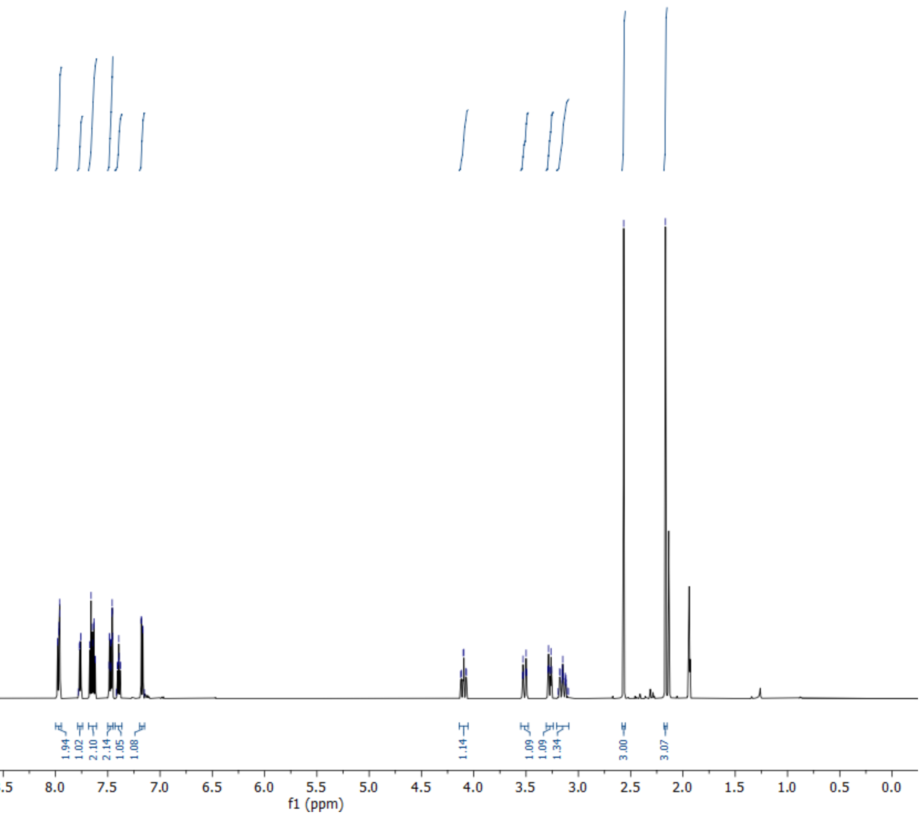

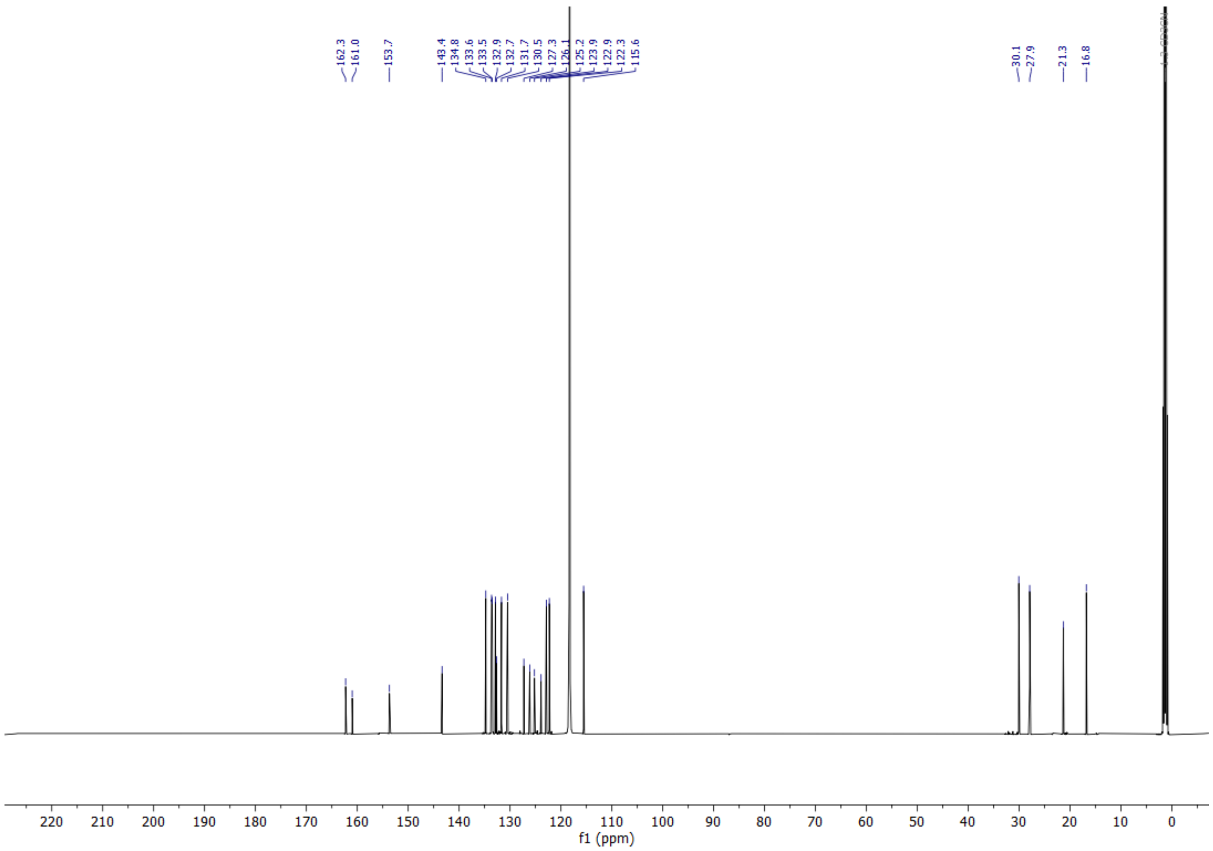

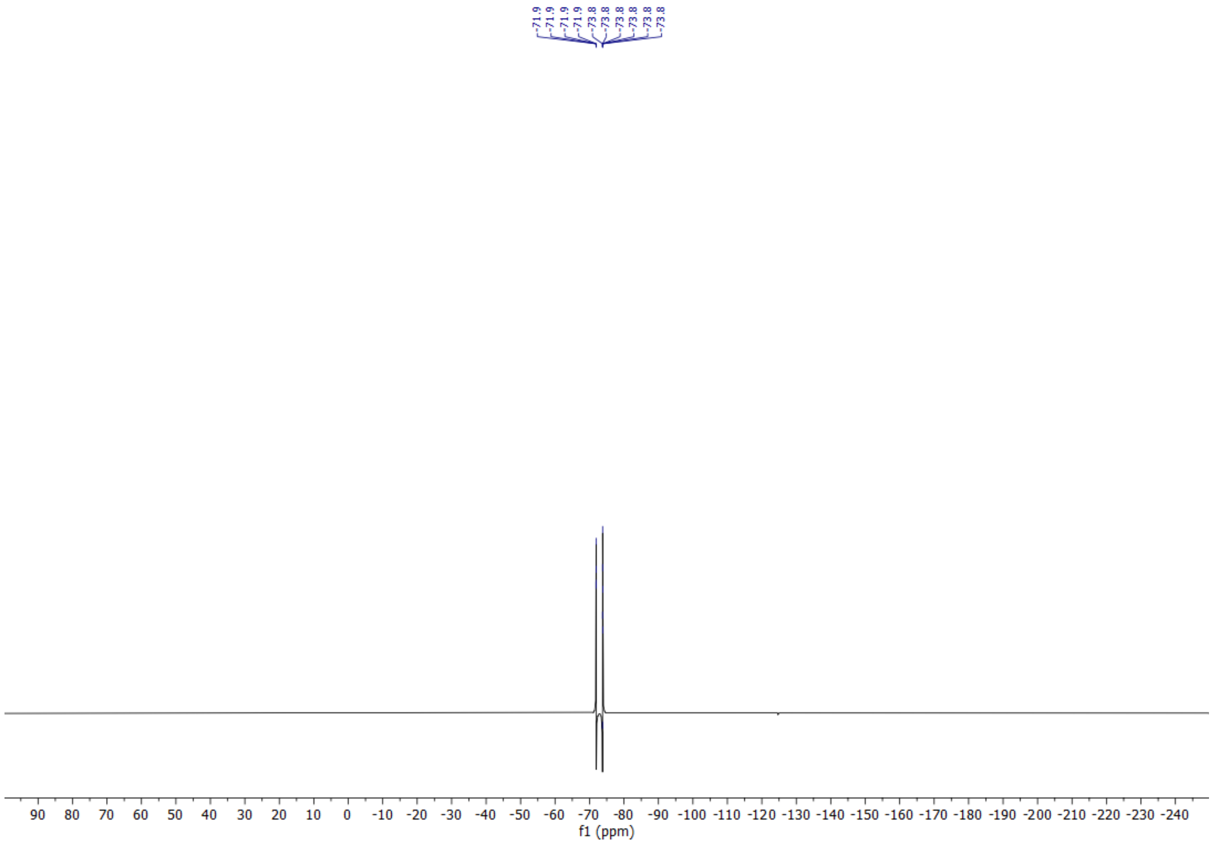
-71.9
-71.9
-71.9
-71.9
-73.8
-73.8
-73.8
-73.8
-73.8
-73.8
90 80 70 60 50 40 30 20 10 0 -10 -20 -30 -40 -50 -60 -70 -80 -90 -100 -110 -120 -130 -140 -150 -160 -170 -180 -190 -200 -210 -220 -230 -240
f1 (ppm)

Sodium bis-(*R*)-BINOL borate, S10:

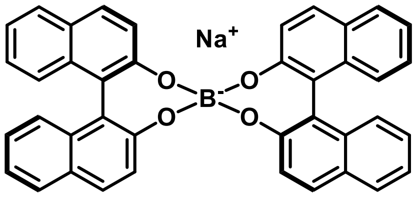

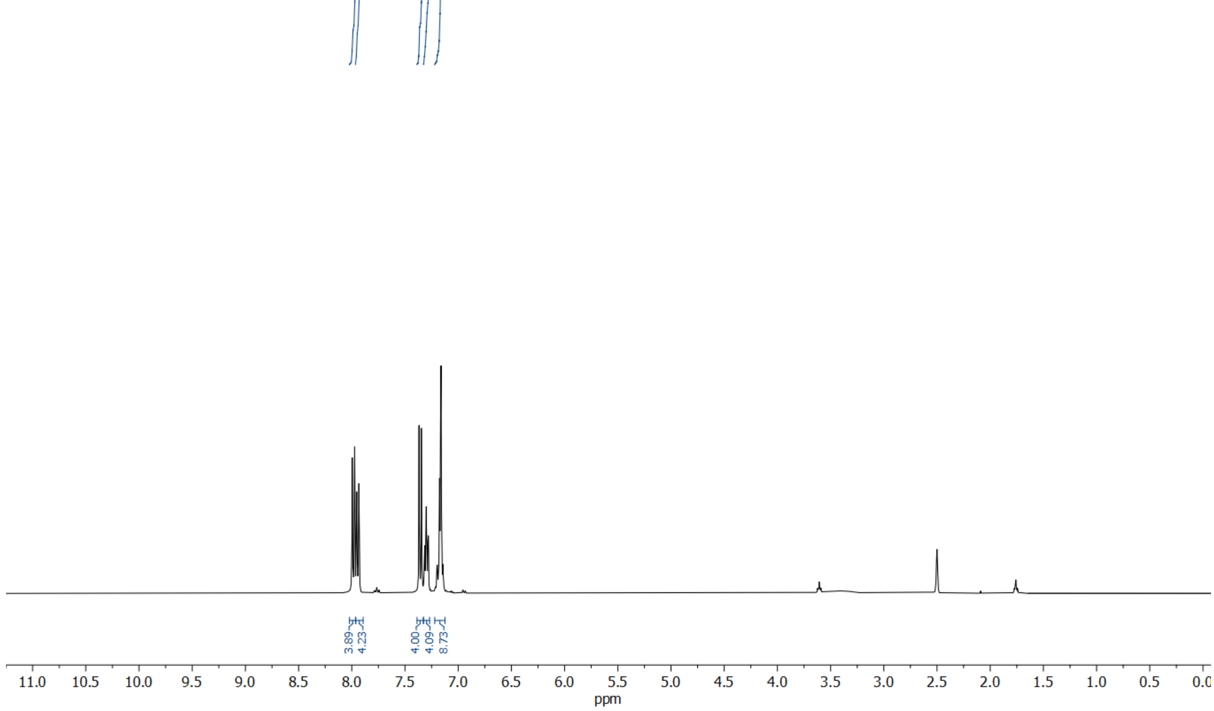

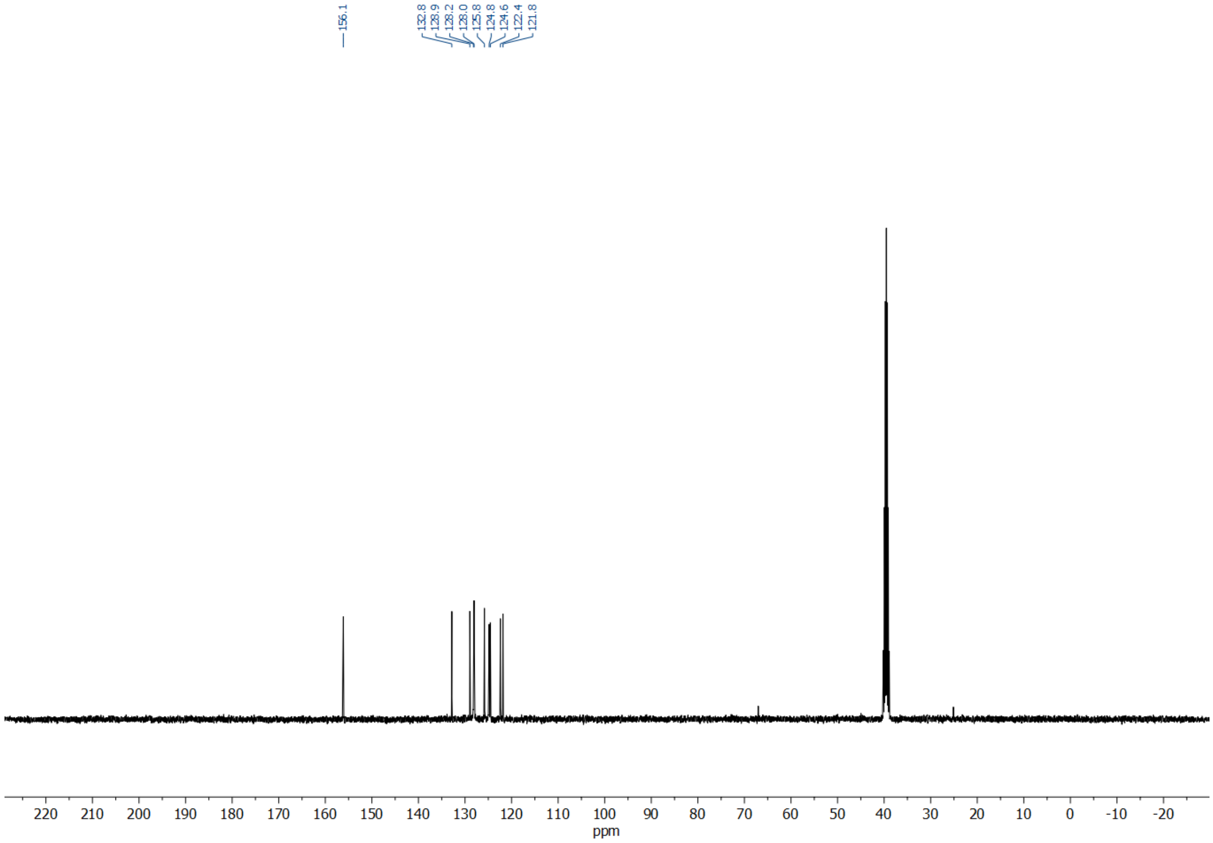

11,13-Dimethyl-4,5-dihydrobenzo[*b*]benzo[6,7]oxepino[3,2,1-*hi*]benzofuran-10-ium, bis-(*R*)-BINOL borate, 18 [(*P*, R_O)-(*R*)] and 19 [(*M*, S_O)-(*R*)] (1:1 mixture of diastereoisomers):

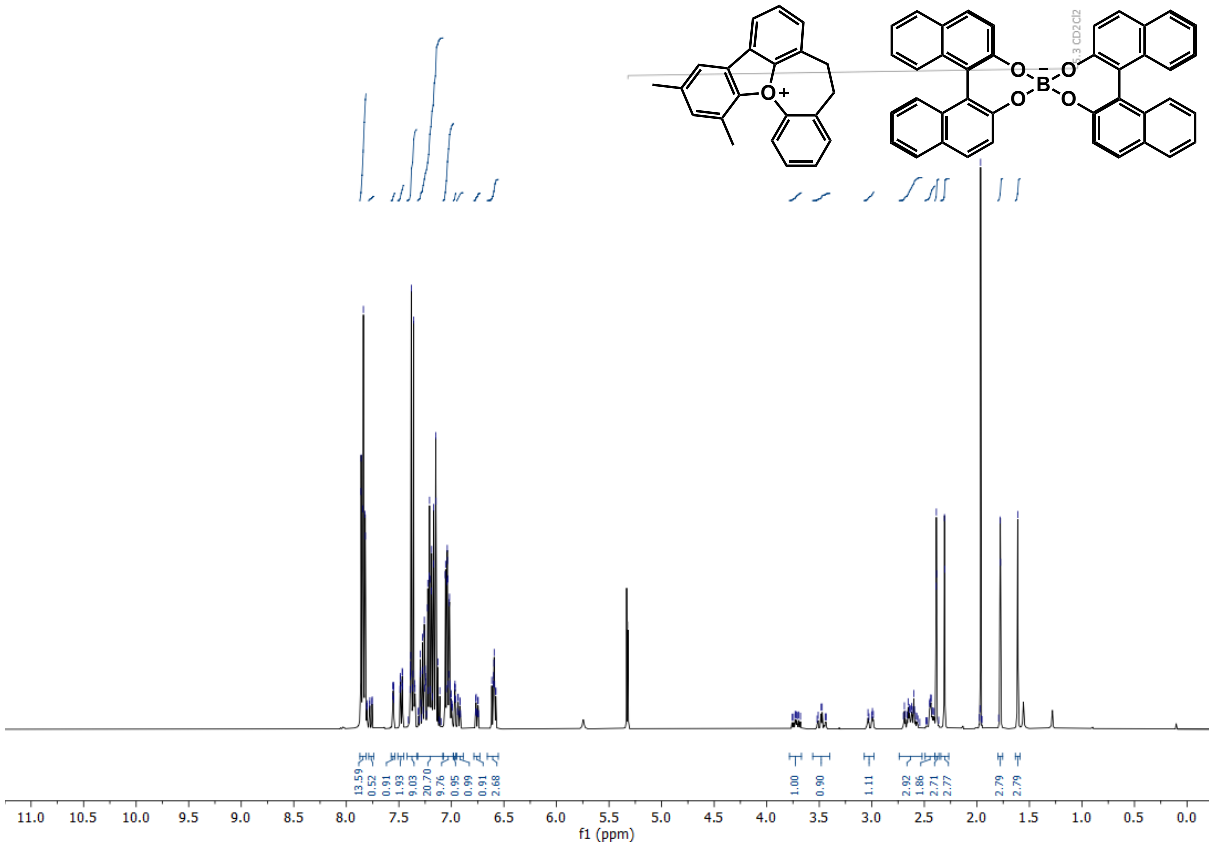

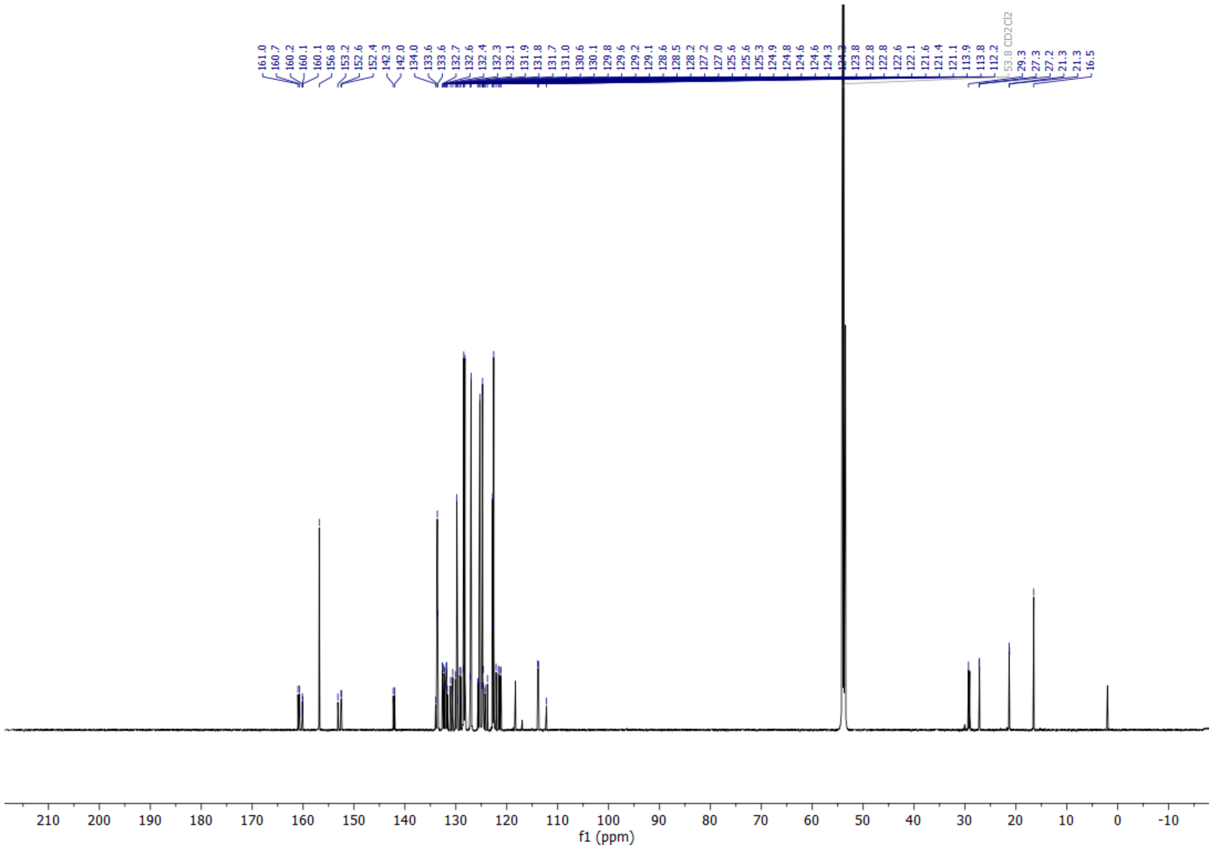

(*P*, *R*$_O$)-11,13-Dimethyl-4,5-dihydrobenzo[*b*]benzo[6,7]oxepino[3,2,1-*hi*]benzofuran-10-ium, bis-(*R*) - BINOL borate, 18:

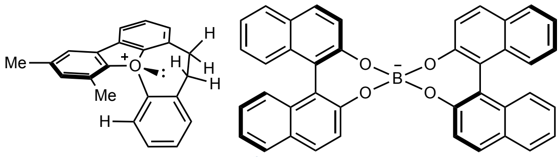

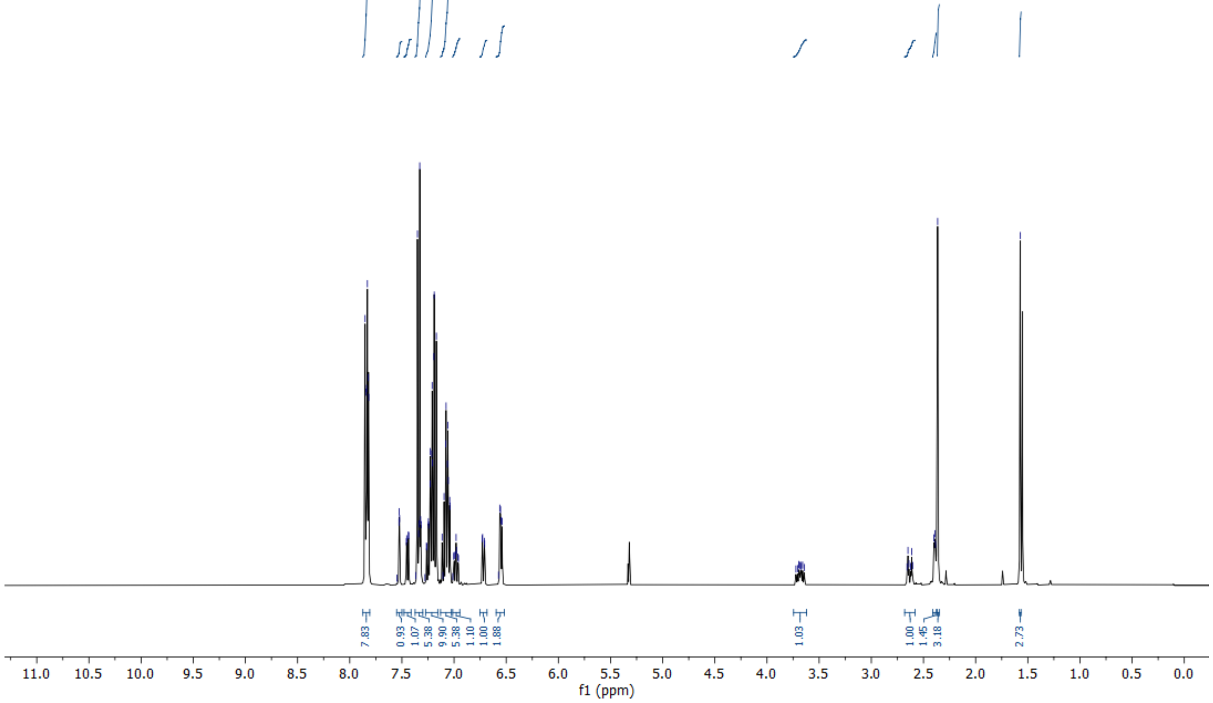

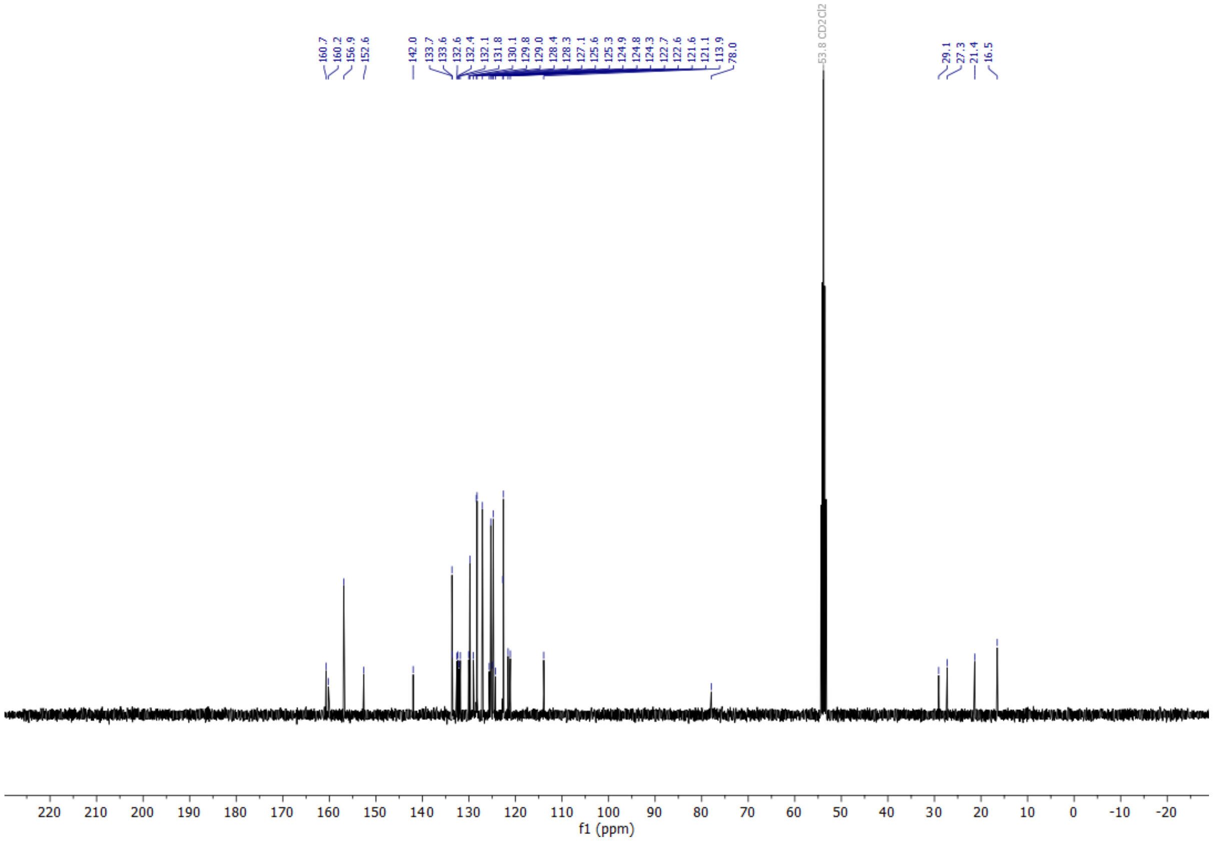

8 References

1. S. Song, X. Sun, X. Li, Y. Yuan, N. Jiao, *Org. Lett.* **2015**, *17*, 2886–2889.
2. E. Vieira, J. Huwyler, S. Jolidon, F. Knoflach, V. Mutel, J. Wichmann, *Bioorg. Med. Chem. Lett.* **2009**, *19*, 1666–1669.
3. T. Ishiyama, M. Murata, N. Miyaura, *J. Org. Chem.* **1995**, *60*, 7508–7510.
4. F. Mongin, M. Schlosser, *Tetrahedron Lett.* **1996**, *37*, 6551–6554.
5. W. Schmidt, R. Wingen, B. Hornung, US Patent, 2005/255258, 7.
6. T. Matsuda, S. Sato, *J. Org. Chem.* **2013**, *78*, 3329–3335.
7. J. A. Raskatov, J. M. Brown, A. L. Thompson, *CrystEngComm*, **2011**, *13*, 2923–2929.
8. J. Cosier and A. M. Glazer, *J. Appl. Crystallogr.* **1986**, *19*, 105–107.
9. L. Palatinus, G. Chapuis, *J. Appl. Crystallogr.* **2007**, *40,* 786–790.
10. P. Parois, R. I. Cooper, A. L. Thompson, *Chem. Cent. J.* **2015**, *9*, 30.
11. R. I. Cooper, A. L. Thompson, D. J. Watkin, *J. Appl. Crystallogr.* **2010**, *43*, 1100–1107.
12. H. D. Flack, G. Bernardinelli, D. A. Clemente, A. Linden, A. L. Spek, *Acta Crystallogr. Sect. B Struct. Sci.* **2006**, *62*, 695–701.
13. H. D. Flack, *Acta Cryst. A*, **1983**, *39*, 876–881.
14. S. Parsons, H. D. Flack, T. Wagner, *Acta Cryst. B* **2013**, *69*, 249–259.
15. R. W. W. Hooft, L. H. Straver, A. L. Spek, *J. Appl. Crystallogr.* **2008**, *41*, 96–103.
16. A. Ahmed, R. A. Bragg, J. Clayden, L. W. Lai, C. McCarthy, J. H. Pink, N. Westlund, S. A. Yasin, *Tetrahedron*, **1998**, *54*, 13277–13294.
17. M. Reist, B. Testa, P. A. Carrupt, M. Jung, V. Schurig. *Chirality* **1995**, *7*, 396−400
18. Gaussian 16, Revision C.01, M. J. Frisch, G. W. Trucks, H. B. Schlegel, G. E. Scuseria, M. A. Robb, J. R. Cheeseman, G. Scalmani, V. Barone, G. A. Petersson, H. Nakatsuji, X. Li, M. Caricato, A. V. Marenich, J. Bloino, B. G. Janesko, R. Gomperts, B. Mennucci, H. P. Hratchian, J. V. Ortiz, A. F. Izmaylov, J. L. Sonnenberg, D. Williams-Young, F. Ding, F. Lipparini, F. Egidi, J. Goings, B. Peng, A. Petrone, T. Henderson, D. Ranasinghe, V. G. Zakrzewski, J. Gao, N. Rega, G. Zheng, W. Liang, M. Hada, M. Ehara, K. Toyota, R. Fukuda, J. Hasegawa, M. Ishida, T. Nakajima, Y. Honda, O. Kitao, H. Nakai, T. Vreven, K. Throssell, J. A. Jr. Montgomery, J. E. Peralta, F. Ogliaro, M. J. Bearpark, J. J. Heyd, E. N. Brothers, K. N. Kudin, V. N. Staroverov, T. A. Keith, R. Kobayashi, J. Normand, K. Raghavachari, A. P. Rendell, J. C. Burant, S. S. Iyengar, J. Tomasi, M. Cossi, J. M. Millam, M. Klene, C. Adamo, R. Cammi, J. W. Ochterski, R. L. Martin, K. Morokuma, O. Farkas, J. B. Foresman, D. J. Fox, Gaussian, Inc., Wallingford CT, **2016**.
19. K. Fukui, *Acc. Chem. Res.* **1981**, *14*, 363−368.
20. S. Grimme, *Chem. Eur. J.* **2012**, 18, 9955−9964.
21. G. Luchini, J. V. Alegre-Requena, I. Funes-Ardoiz, R. S. Paton, *F1000Research*, **2020**, *9*, 291.
22. CYLview, 1.0b; C. Y. Legault, Université de Sherbrooke, 2009 (http://www.cylview.org)
23 a) A. D. Becke, *J. Chem. Phys.* **1993**, *98*, 5648–5652; b) C. Lee, W. Yang, R. G. Parr, *Phys. Rev. B*, **1988**, *37*, 785–789; c) S. H. Vosko, L. Wilk, M. Nusair, *Can. J. Phys.* **1980**, *58*, 1200–1211; d) P. J. Stephens, F. J. Devlin, C. F. Chabalowshi, M. J. Frisch, *J. Phys. Chem.* **1994**, *98*, 11623–11627.
24. S. Grimme, S. Ehrlich, L. Goerigk, *J. Comp Chem.* **2011**, *32*, 1456–1465.
25. a) W. J. Hehre, R. Ditchfield, J. A. Pople, *J. Chem. Phys.* **1972**, *56*, 2257–2261; b) P. C. Hariharan, J. A. Pople, *Theor. Chim. Acta.* **1973**, *28*, 213–222; c) R. Krishnan, J. S. Binkley, R. Seeger, J. A. Pople, *J. Chem. Phys.* **1980**, *72*, 650–654; d) A. D. McLean, G. S. Chandler, *J. Chem. Phys.* **1980**, *72*, 5639–5648; e) M. M. Francl, W. J. Pietro, W. J. Hehre, J. S. Binkley, M. S. Gordon, D. J. DeFrees, J. A. Pople, *J. Chem. Phys.* **1982**, *77*, 3654–3665.
26. G. Scalmani, M. J. Frisch, *J. Chem. Phys.* **2010**, *132*, 114110-114115.
27. V. Barone, M. Cossi, *J. Phys. Chem. A* **1998**, *102*, 1995-2001.
28. A. V. Marenich, C. J. Cramer, D. G. Truhlar, *J. Phys. Chem. B*, **2009**, *113*, 6378–6396.
29. a) P. Pracht, F. Bohle, S. Grimme, *Phys. Chem. Chem. Phys.* **2020**, *22*, 7169-7192; b) S. Grimme, *J. Chem. Theory Comput.* **2019**, *15*, 2847-2862.
30. a) Y. Zhao, D. G. Truhlar, *Theor. Chem. Acc*. **2008**, *120*, 215–241; b) S. Grimme, J. Antony, S. Ehrlich, H. Krieg, *J. Chem. Phys.* **2010**, *132*, 154104–154119.
31. a) F. Weigend, R. Ahlrichs, *Phys. Chem. Chem. Phys.* **2005**, *7*, 3297–3305; b) F. Weigend, *Phys. Chem. Chem. Phys.* **2006**, *8*, 1057–1065.
32. a) J. -D. Chai, M. Head-Gordon, *Phys. Chem. Chem. Phys.* **2008**, *10*, 6615-6620; b) J. -D. Chai, M. Head-Gordon, *J. Chem. Phys.* **2008**, *128*, 084106.
33. The PyMol Molecular Graphics System, Version 2.5.4., Schrödinger, LLC.
34. NBO 7.0, E. D. Glendening, J, K. Badenhoop, A. E. Reed, J. E. Carpenter, J. A. Bohmann, C. M. Morales, P. Karafiloglou, C. R. Landis, and F. Weinhold, Theoretical Chemistry Institute, University of Wisconsin, Madison, **2018**.
